# Extracellular Vesicles for Clinical Diagnostics: From Bulk Measurements to Single-Vesicle Analysis

**DOI:** 10.1021/acsnano.5c00706

**Published:** 2025-07-28

**Authors:** Hai Linh Tran, Wenshu Zheng, David A. Issadore, Hyungsoon Im, Yoon-Kyoung Cho, Yuanqing Zhang, Dingbin Liu, Yang Liu, Bo Li, Fei Liu, David Tai Wai Wong, Jiashu Sun, Kun Qian, Mei He, Meihua Wan, Yong Zeng, Ke Cheng, Tony Jun Huang, Daniel T. Chiu, Luke P. Lee, Lei Zheng, Andrew K. Godwin, Raghu Kalluri, Steven A. Soper, Tony Y. Hu

**Affiliations:** † Center for Cellular and Molecular Diagnostics and Department of Biochemistry and Molecular Biology, 12255 Tulane University School of Medicine, New Orleans, Louisiana 70112, United States; ‡ Department of Bioengineering, University of Pennsylvania, Philadelphia, Pennsylvania 19104, United States; § Department of Radiology, Massachusetts General Hospital, 1811 Harvard Medical School, Boston, Massachusetts 02114, United States; ∥ Department of Biomedical Engineering, Ulsan National Institute of Science and Technology (UNIST), Ulsan 44919, Republic of Korea; ⊥ School of Pharmaceutical Sciences, 26469 Sun Yat-Sen University, Guangzhou, Guangdong 510006, China; # State Key Laboratory of Medicinal Chemical Biology, Research Center for Analytical Sciences, Tianjin Key Laboratory of Molecular Recognition and Biosensing, College of Chemistry, 12538 Nankai University, Tianjin 300071, China; ∇ Department of Hepatobiliary Cancer, Liver Cancer Center, Tianjin Medical University Cancer Institute & Hospital, National Clinical Research Center for Cancer, Key Laboratory of Cancer Prevention and Therapy, 74675 Tianjin’s Clinical Research Center for Cancer, Tianjin 300060, China; ○ Department of Laboratory Medicine, Guangdong Provincial Key Laboratory of Precision Medical Diagnostics, Guangdong Engineering and Technology Research Center for Rapid Diagnostic Biosensors, Guangdong Provincial Key Laboratory of Single Cell Technology and Application, Nanfang Hospital, 198153 Southern Medical University, Guangzhou 510515, P. R. China; ◆ Department of Medicine, Brigham and Women’s Hospital, 1811 Harvard Medical School, Boston, Massachusetts 02115, United States; ¶ School of Dentistry, 8783 University of California, Los Angeles, California 90095, United States; & Beijing Engineering Research Center for BioNanotechnology, CAS Key Laboratory of Standardization and Measurement for Nanotechnology, National Center for Nanoscience and Technology, Beijing 100190, China; ● School of Future Technology, University of Chinese Academy of Sciences, Beijing 100049, China; ◊ State Key Laboratory of Systems Medicine for Cancer, School of Biomedical Engineering and Institute of Medical Robotics, Division of Cardiology, Renji Hospital, School of Medicine, 12474 Shanghai Jiao Tong University, Shanghai 200030, PR China; ▲ Shanghai Jiao Tong University Sichuan Research Institute, Chengdu 610213, PR China; □ Department of Pharmaceutics, College of Pharmacy; 3463 University of Florida, Gainesville, Florida 32611, United States; ∧ Department of Integrated Traditional Chinese and Western Medicine, West China Hospital, 617912 Sichuan University, Chengdu, Sichuan 61004, China; ¢ Department of Chemistry, 3463 University of Florida, Gainesville, Florida 32611, United States; + University of Florida Health Cancer Center, Gainesville, Florida 32610, United States; $ J. Crayton Pruitt Family Department of Biomedical Engineering, 3463 University of Florida, Gainesville, Florida 32611, United States; ∠ Department of Biomedical Engineering, 5798 Columbia University, New York, New York 10027, United States; € Herbert Irving Comprehensive Cancer Center, 5798 Columbia University, New York, New York 10027, United States; ¤ Department of Mechanical Engineering and Materials Science, 3065 Duke University, Durham, North Carolina 27708, United States; ¥ Departments of Chemistry and Bioengineering, 7284 University of Washington, Seattle, Washington 98195, United States; ☼ Institute of Quantum Biophysics, Sungkyunkwan University, Suwon 16419, Republic of Korea; ± Department of Medicine, Brigham and Women’s Hospital, Harvard Medical School, Harvard University, Boston, Massachusetts 02115, United States; ∞ Department of Chemistry & Nanoscience, Ewha Womans University, Seoul 03760, Republic of Korea; †† Guangzhou Key Laboratory of Chinese Medicine Research on Prevention and Treatment of Osteoporosis, The Third Affiliated Hospital of Guangzhou University of Chinese Medicine, Guangzhou, Guangdong 510378, China; ‡‡ The Third Clinical Medical School of Guangzhou University of Chinese Medicine, Guangzhou, Guangdong 510378, China; §§ Department of Pathology and Laboratory Medicine, 21638 University of Kansas Medical Center, Kansas City, Kansas 66160, United States; ∥∥ Kansas Institute for Precision Medicine, 21638 University of Kansas Medical Center, Kansas City, Kansas 66160, United States; ⊥⊥ Department of Cancer Biology, Metastasis Research Center, 4002 University of Texas MD Anderson Cancer Center, Houston, Texas 77054, United States; ## Department of Bioengineering, Rice University, Houston, Texas 77054, United States; ∇∇ Department of Molecular and Cellular Biology, Baylor College of Medicine, Houston, Texas 77054, United States; ○○ Center of BioModular Multiscale Systems for Precision Medicine (CBM2), University of Kansas, Lawrence, Kansas 66045, United States; ◆◆ Department of Chemistry, University of Kansas, Lawrence, Kansas 66045, United States; ¶¶ Department of Mechanical Engineering, University of Kansas, Lawrence, Kansas 66045, United States; && Bioengineering Program, University of Kansas, Lawrence, Kansas 66045, United States; ●● KU Comprehensive Cancer Center and Kansas Institute for Precision Medicine, 21638 University of Kansas Medical Center, Kansas City, Kansas 66160, United States

**Keywords:** extracellular vesicles (EVs), single EV analysis, biomarkers, diagnostics, analytical techniques

## Abstract

Extracellular vesicles (EVs) play a crucial role in intercellular communication, signaling pathways, and disease pathogenesis by transporting biomolecules such as DNA, RNA, proteins, and lipids derived from their cells of origin, and they have demonstrated substantial potential in clinical applications. Their clinical significance underscores the need for sensitive methods to fully harness their diagnostic potential. In this comprehensive review, we explore EV heterogeneity related to biogenesis, structure, content, origin, sample type, and function roles; the use of EVs as disease biomarkers; and the evolving landscape of EV measurement for clinical diagnostics, highlighting the progression from bulk measurement to single vesicle analysis. This review covers emerging technologies such as single-particle tracking microscopy, single-vesicle RNA sequencing, and various nanopore-, nanoplasmonic-, immuno-digital droplet–, microfluidic-, and nanomaterial-based techniques. Unlike traditional bulk analysis methods, these methods contribute uniquely to EV characterization. Techniques like droplet-based single EV-counting enzyme-linked immunosorbent assays (ELISA), proximity-dependent barcoding assays, and surface-enhanced Raman spectroscopy further enhance our ability to precisely identify biomarkers, detect diseases earlier, and significantly improve clinical outcomes. These innovations provide access to intricate molecular details that expand our understanding of EV composition, with profound diagnostic implications. This review also examines key research challenges in the field, including the complexities of sample analysis, technique sensitivity and specificity, the level of detail provided by analytical methods, and practical applications, and we identify directions for future research. This review underscores the value of advanced EV analysis methods, which contribute to deep insights into EV-mediated pathological diversity and enhanced clinical diagnostics.

## Graphical abstract

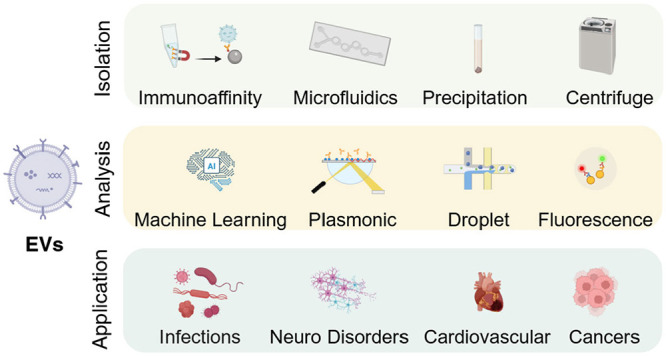

Extracellular vesicles (EVs) are membrane-enclosed particles expelled by cells into the extracellular milieu. They encompass exosomes (∼40 to ∼160 nm in diameter), originating from the endosomal pathway; ectosomes (∼50 nm to ∼1 μm in diameter), shed directly from the plasma membrane; and apoptotic bodies (∼50 nm to ∼5 μm), generated during programmed cell death. EVs carry a diverse collection of biomolecules, including proteins, glycoproteins, lipids, RNAs, DNA, enzymes, and metabolites, which can reflect the molecular composition of their cells of origin (e.g., parent cells).^1^

After being expelled into the extracellular milieu, EVs can transport their biomolecules to recipient cells. The recipient cells can internalize EVs through mechanisms including phagocytosis, micropinocytosis, receptor-mediated endocytosis, or direct fusion with the cell membrane.^2^ This uptake is influenced by factors such as EV origin, size, and content, as well as the structure of recipient cell membranes.^3^ The biomolecular cargo of EVs includes transcription factors, signaling molecules, or other regulatory components, influencing the behavior of recipient cells and impacting gene expression, cell signaling, and cellular functions. Additionally, EVs can modify recipient cell functions through an alternative mechanism that involves binding to surface receptors or interacting with lipid rafts on the cell membrane, which can modify recipient cell functions without the direct transfer of biomolecules.^4^ Regardless of the mechanism, their ability to facilitate communication between adjacent cells or between distant cells (via systemic transfer in bodily fluids like blood) supports their critical role in diverse physiological and pathological processes, such as immune response, tissue regeneration, vascular health, tumor growth, metastasis, and neurodegenerative disorders.^5^

With their unique features, EVs are a promising source of biomarkers for clinical diagnostics. Unlike traditional biomarkers, which provide limited information and can degrade rapidly, EVs provide a wealth of information that is encapsulated and protected from degradation.^6^ EV-derived biomarkers have the advantage of remarkable stability in bodily fluids such as blood, plasma, urine, saliva, sweat, and breast milk, as well as cerebrospinal, amniotic, seminal, and bronchoalveolar lavage fluids. With this stability, EVs are reliable indicators of disease states and physiological changes in their originating cells and tissues. The potential of EVs for clinical diagnostics is considerable, covering a wide range of diseases such as chronic, degenerative, and infectious diseases, in addition to cancer. This broad applicability highlights their potential as diagnostic tools.^7^

Despite their promise, EVs are difficult to analyze because of their diverse nature, small size, and the complexity of their biological functions. Over the past decade, EV research has expanded rapidly, initially focusing on bulk EV (BuEV) measurements, which assess the collective characteristics of a heterogeneous EV population. Traditional BuEV measurement techniques have elucidated the biogenesis, cargo composition, and functional roles of EVs, and some, like flow cytometry, dynamic light scattering (DLS), and nanoparticle tracking analysis (NTA), allow for the quantification and size characterization of EV populations. However, BuEV measurements fall short in providing detailed molecular information about single EVs (SiEVs), concealing subtle distinctions between subpopulations with unique biological functions that may prove essential for accurate diagnostics. In contrast, SiEV analysis offers the ability to examine individual vesicles. Recent techniques, including single-particle tracking microscopy, single-particle RNA sequencing, nanoflow cytometry, digital droplet polymerase chain reaction (ddPCR), immuno-digital droplet PCR (iddPCR), droplet-based single exosome-counting enzyme-linked immunosorbent assays (droplet digital ExoELISA), nanoplasmon-enhanced scattering (nPES), resistive pulse sensing (RPS), and nanomaterial-integrated SiEV isolation techniques,^8,9^ have been developed to allow for a more precise characterization of EV size, composition, and functional properties, and have enabled the detection of specific biomarkers or molecular signatures associated with distinct disease states. By providing higher resolution, SiEV analysis can uncover diagnostic insights that BuEV methods might miss. The increasing focus on SiEV analysis reflects its promise for enhancing diagnostic precision and deepening our understanding of disease mechanisms and progression.

This review seeks to delve into the constraints of conventional EV analysis methodologies and highlight the rise of SiEV analysis techniques and their considerable potential and benefits for clinical diagnostics. By examining the intricacies of SiEV analysis, we seek to elucidate its substantial impact on our understanding of EV biology and its translation into enhanced diagnostic strategies for various diseases. From the initial stages of diagnosis to the intricate realm of precision medicine applications, EVs emerge as indispensable tools, holding substantial promise for early detection and precise diagnosis. Because of the increasing number of studies on EVs for clinical medicine, it is vital to understand the evolution of EV analysis techniques and articulate the nuanced advantages and drawbacks associated with each technique. This exploration is not merely an academic pursuit, but a crucial stride toward unlocking the full potential of these promising tools in the health care sector. In this review, we provide an overview of EVs, followed by a comprehensive exploration of their heterogeneity and their role as a source of biomarkers. We then discuss advancements in isolation and analytical techniques, tracing the transition from BuEV measurements to SiEV interrogation and elucidating the promise and challenges inherent in each methodology. Finally, we discuss clinical applications, including clinical trials involving EVs, and provide perspectives and directions for future research. With this review, we aspire to unveil novel avenues for precision diagnostics and personalized medicine.

## 1 Overview of EVs

EVs, enclosed by a lipid bilayer membrane and containing cytoplasmic molecules, are formed by a broad spectrum of organisms, ranging from microbes to mammals, underscoring their fundamental role in biological systems. EVs are also ubiquitous, released into the extracellular milieu by all examined cell types, regardless of their physiological state,^10,11^ and present in all tissues and physiological fluids, including blood, plasma, urine, saliva, sweat, breast milk, cerebrospinal fluid, amniotic fluid, seminal fluid, and bronchoalveolar lavage fluid.^12^ Their cargo can be transferred to recipient cells, influencing cellular behavior and orchestrating diverse physiological responses.^13^ EVs have also been implicated in numerous pathologies, ranging from cancer and neurodegenerative disorders to infectious diseases.^14^ Such ubiquity not only emphasizes the interconnectedness of life but also underscores the pivotal role of EVs in diverse biological functions, with direct implications extending to clinical contexts.^15^

The International Society for Extracellular Vesicles defines EVs, in its guidance titled *Minimal Information for Studies of Extracellular Vesicles (MISEV2023)*, as “particles that are released from cells, are delimited by a lipid bilayer, and cannot replicate on their own (i.e., do not contain a functional nucleus).” Within this definition, EVs are a heterogeneous family, often classified by their size, origin, surface markers, and cargo. Generally, EVs < 200 nm in diameter are defined as small (sEVs), while EVs with diameters exceeding this number are considered large EVs (lEVs). Terms such as “exosomes,” “ectosomes” (also known as microvesicles), and “apoptotic bodies” classify EVs on the basis of their cellular origin. EVs are also classified by their surface markers, including membrane proteins and lipids, and cargo, including molecules such as nucleic acids (DNA, RNAs), metabolites, enzymes, lipids, glycans, and proteins.^10^ While EVs are often described as reflective of their parent cells, given that their lipid bilayer membranes resemble the plasma membrane of the originating cells, and their cargo selectively represents components of the originating cells, this mirroring is not absolute. For example, sEVs do not carry full-length mRNA transcripts, but instead, selectively package truncated mRNAs (mRNAs) or specific RNA subsets. This selective cargo packaging underscores the regulated nature of EV biogenesis and highlights the utility of EVs in understanding the physiological state, health, and function of their cells of origin, offering a window into the intricate landscape of pathological diversity. EV studies promise to transform our understanding of cell-to-cell communication, offer new insights into disease mechanisms, and open avenues for innovative diagnostic and therapeutic interventions.^16,17^

## 2 Heterogeneity of EVs

EVs collected from different sample sources exhibit considerable heterogeneity, arising from variations in their cellular origin, biogenesis pathways, and complex microenvironments.^18,19^ This diversity manifests in several ways, including differences in size, shape, density, cargo content, and functional traits. Understanding this heterogeneity is crucial for understanding the role of EVs in disease and unlocking their potential as diagnostic tools. Variations in EV characteristics can impact their utility as noninvasive biomarkers, influencing their effectiveness in disease detection and monitoring. Therefore, in this section, we will explore the various dimensions of EV heterogeneity in detail, focusing on their biogenesis and secretion, size, shape, content, source, and function (Figure 1). This detailed examination will provide a clear and organized understanding of EV diversity and its implications for disease diagnostics.

**Figure 1:**
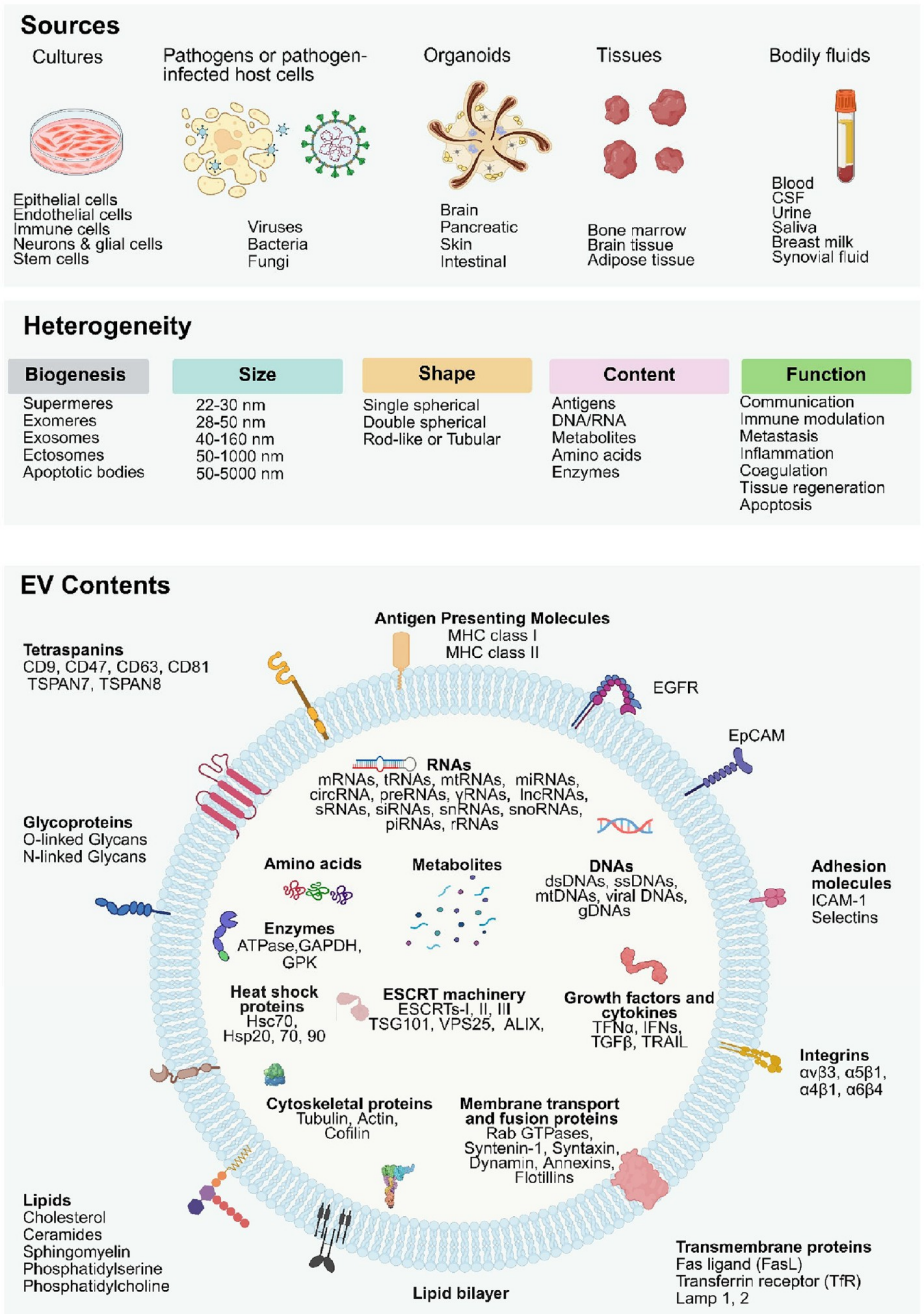
Heterogeneity of Extracellular Vesicles. EVs collected from different sources (cell cultures, pathogens or pathogen-infected host cells, organoids, tissues, or bodily fluids; top) exhibit significant heterogeneity in their biogenesis, size, shape, content, and function (middle), and their molecular content (bottom), reflecting the characteristics of the cells from which they originated. The contents of EVs are varied, encompassing both surface components (such as membrane proteins, glycoproteins, and lipids) and internal cargo (including RNAs, amino acids, metabolites, DNAs, enzymes, and proteins). These biomolecules contribute to EVs’ roles in intercellular communication, immune modulation, metastasis, inflammation, coagulation, tissue regeneration, and apoptosis. The figure emphasizes the complexity of individual EVs and their ability to transport biologically active molecules, influencing various biological processes across different tissues and organs. Figure created with Biorender.com.

### 2.1 Biogenesis and Secretion

EV biogenesis involves intricate processes that can be broadly categorized into endosomal sorting complex required for transport (ESCRT)-dependent and ESCRT-independent pathways, each contributing to the formation of different EV subtypes and influencing cargo loading mechanisms. EVs are formed via distinct cellular mechanisms (Figure 2).^16,20^ For example, exosomes are formed via the endosomal pathway, which is initiated by inward budding of the plasma membrane to generate early endosomes. When these endosomes reach a more advanced stage (late endosomes), they start to produce intraluminal vesicles (ILVs) by inward budding of the endosomal membrane, resulting in the creation of multivesicular bodies (MVBs). MVBs can either fuse with lysosomes for degradation or fuse with the plasma membrane to release ILVs into the extracellular space, generating exosomes. In contrast to exosomes, ectosomes are produced by outward budding of the plasma membrane, a phenomenon referred to as exocytosis. Actin and myosin are involved in promoting membrane protrusion and the release of ectosomes.^21^ In apoptosis, a programmed cell death process, cells undergo morphological changes leading to fragmentation and the release of apoptotic bodies into the external environment.^22^ In this section, we provide more details about EV biogenesis and secretion.

**Figure 2:**
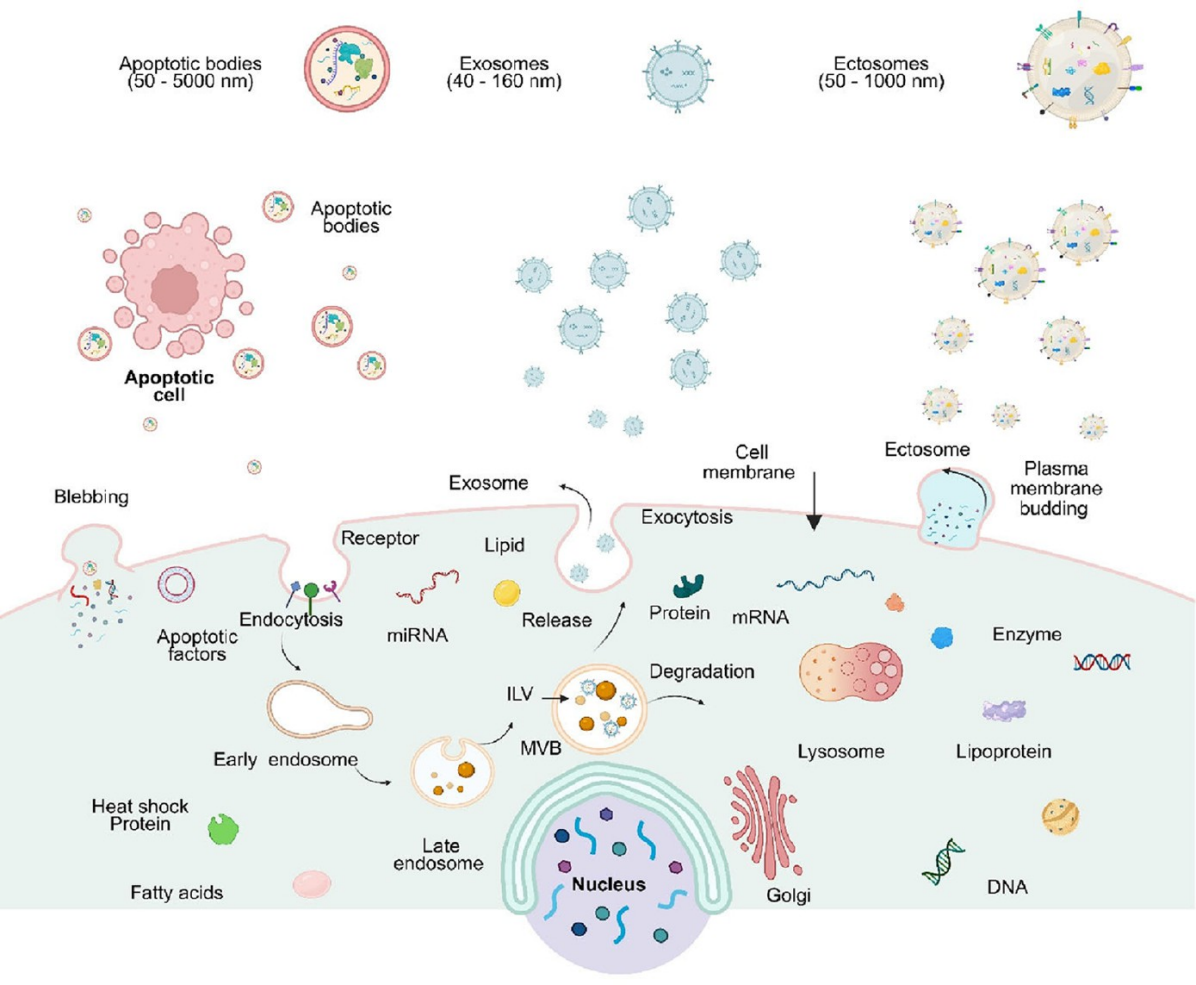
Biogenesis and secretion of EVs. EVs can be broadly classified into three major subtypes: exosomes, ectosomes (also known as microvesicles), and apoptotic bodies. Exosomes originate from the endosomal pathway, in which early endosomes mature into late endosomes. Late endosomes develop intraluminal vesicles (ILVs), becoming multivesicular bodies (MVBs), which then follow either a degradative pathway or a secretory pathway. In the latter, MVBs fuse with the plasma membrane and release their ILVs as exosomes. Ectosomes are generated by budding from the cell membrane. Apoptotic bodies are released during apoptosis, when cells undergo programmed cell death and fragmentation. Figure created with Biorender.com.

#### 2.1.1 Exosomes

The ESCRT-dependent pathway is the canonical mechanism for exosome biogenesis, primarily involving the inward budding of the membrane within early endosomes and the formation of ILVs. This process begins with the internalization of membrane proteins and lipids into early endosomes through endocytosis. Once formed, these early endosomes mature into MVBs, which contain ILVs that house the cargo to be secreted. The formation and secretion of exosomes are driven by a series of multisubunit protein complexes, collectively known as the ESCRT complexes. These protein complexes include ESCRT-0, -I, -II, and -III, which work alongside associated proteins such as ALIX (apoptosis-linked gene 2–interacting protein X), tetraspanins (TSPANs), and sphingomyelinases, and alongside processes such as phospholipid relocalization and actin cytoskeleton rearrangement, which are integral to exosome formation and release.^23^ ESCRT-0 is crucial in the early stages of exosome biogenesis. It recognizes and binds ubiquitinated cargo proteins on the endosomal membrane and recruits ESCRT-I to the sites of budding. This step is essential for the initial clustering of ubiquitinated proteins as the inward budding of the endosomal membrane begins. ESCRT-I plays a pivotal role in the organization of cargo during the formation of ILVs. It facilitates the arrangement of ubiquitinated proteins and directs them into the maturing ILVs. ESCRT-I adopts an elongated structure, with one of its components, tumor susceptibility gene 101 (TSG101), shaping membrane protrusions to encapsulate cargo.^24^ In addition, ESCRT-I recruits other ESCRT complexes, orchestrating the assembly of the entire ESCRT machinery. ESCRT-II acts as a crucial bridge between ESCRT-I and ESCRT-III, further organizing and concentrating cargo proteins within specific regions of the endosomal membrane, and initiates ESCRT-III assembly. ESCRT-III functions in the final stages of ILV formation and release. It forms spiral filaments that deform and sever the membrane, allowing ILVs to be released into the MVB lumen.^22^ ESCRT-III also mediates fusion of the MVB with the plasma membrane, enabling mature MVBs to release their contents, including exosomes.^23^

Exosomes are also formed by ESCRT-independent pathways, which involve diverse proteins, lipids, and cellular processes that contribute significantly to exosome biogenesis.^25^ TSPAN proteins such as CD9, CD63, CD81, and CD82 arrange membrane microdomains to support ILV development without needing ESCRT proteins.^26^ Cholesterol-rich lipid rafts containing sphingolipids, phosphatidylserine, and ceramide also help organize proteins and start membrane budding.^27,28^ Integral components of lipid rafts, such as flotillins and caveolins, are essential for ESCRT-independent pathways. Flotillins organize lipid rafts and form membrane microdomains that promote membrane curvature, which is critical for ILV budding and exosome biogenesis. They also stabilize these domains, aiding in the clustering of cargo proteins into exosomes. Caveolins, key structural proteins of caveolae (specialized lipid raft domains), regulate membrane invagination, facilitating cargo sorting and membrane budding. In addition, caveolins contribute to signal transduction processes that influence exosome release. The activities of Rab GTPases (Rab27a and Rab27b), along with lipid metabolism involving ceramide production, regulate MVB docking and fusion with the plasma membrane, thereby influencing exosome release.^27^ Proteins like ADP-ribosylation factor 6 (ARF6) and Rho GTPases regulate actin dynamics, crucial for membrane remodeling and exosome release.^29^

#### 2.1.2 Ectosomes

Ectosomes are vesicles formed through direct outward budding of the plasma membrane. The outward budding captures cytosolic material, including proteins and nucleic acids. Unlike exosomes, ectosomes closely resemble the composition of the plasma membrane and can arise through various mechanisms, some overlapping with exosome biogenesis pathways. As a result, ectosomes and exosomes can contain overlapping biomolecules, such as specific proteins like TSPANs and lipids characteristic of the plasma membrane. This similarity in molecular content complicates their differentiation based solely on components. Furthermore, ectosomes can form from membrane regions involved in endocytic processes, such as areas previously engaged in clathrin-mediated endocytosis. These overlaps in size, surface composition, and biogenesis pathways further complicate isolating, distinguishing vesicles of endosomal origin from those derived from the plasma membrane, making it more challenging to explore their specific roles in disease and physiology and to assess their unique potential as biomarkers for diagnostic purposes.

Specific factors are pivotal for ectosome generation. Calcium initiates cytoskeletal remodeling mechanisms, with elevated levels activating cytosolic proteases like calpain and caspase, which disrupt the cytoskeleton and induce ectosome production.^30^ Cholesterol-rich lipid rafts facilitate ectosome biogenesis by sorting specific lipid and protein cargo via anchors on the inner leaflet of the cell membrane.^31^ ARF6, ceramide, and phospholipase D1 (PLD1) are crucial for ectosome formation, with ARF6 selectively loading proteins such as integrin β1 and histocompatibility complex (MHC)-1 and inducing actin-myosin–based contraction, leading to ectosome shedding.^32^ Ectosomes often express markers akin to those on the parent cell’s plasma membrane, such as integrins, selectins, and phosphatidylserine (PS).^10,30^ Recent studies underscore the importance of calcium influx, cytoskeleton reorganization, and the enzymatic functions of proteins like floppases and scramblases in ectosome biogenesis.^32,33^ TSPANs (CD9, CD63, CD81), commonly associated with exosomes, are also found on ectosomes.^34^ Across various physiological cell stages and cell types, ectosomes exhibit diverse biogenesis pathways and surface compositions.

#### 2.1.3 Apoptotic Bodies

Distinct from other ectosomes, apoptotic bodies are formed exclusively during apoptosis and arise from unique mechanisms such as phospholipid reorganization, which induces membrane blebbing and organelle inclusion. During apoptosis, cells fragment and release apoptotic bodies, which are characterized by their distinct morphology and composition. These bodies are formed after the cell has selectively excluded nuclear content, contributing to their unique features as a subpopulation of ectosomes.^18,30^ Apoptotic bodies contain a diverse array of cellular components, including intact organelles like mitochondria, the endoplasmic reticulum, and the Golgi apparatus. Additionally, they contain fragments of chromatin, DNA, and RNA, reflecting the process of programmed cell death. Alongside these nucleic acids, apoptotic bodies also carry various proteins that serve as markers of apoptosis, such as caspases and histones.^3,20,35^ Apoptotic bodies exhibit PS on their external membrane surface and undergo clearance by macrophages. The heterogeneity of apoptotic bodies encompasses variations in size, composition, and functional characteristics, despite their shared origin.^36^ This diversity may stem from factors such as the type of cell undergoing apoptosis, the signaling pathways involved, and the surrounding microenvironment.

#### 2.1.4 Other EV Subtypes

Recent advancements in isolation techniques and analytical methods have led to the identification of a growing variety of EV subtypes, which have been found to be prevalent components of the extracellular space and bodily fluids. Among these newly identified EV-like structures are exomeres and supermeres, which differ significantly from well-established EV subtypes.^37^ Unlike other EVs, exomeres and supermeres lack the lipid bilayer membrane that typically defines these vesicles. They are enriched with a diverse range of biomolecules, including proteins and nucleic acids, suggesting potential roles in intercellular communication and other biological functions. Exomeres, typically ranging from 28 to 50 nm in size, have been shown to contain notable proteins such as HSP90AB1, Hsp90-β, FASN, and ACLY, which could serve as biomarkers for their identification and further characterization.^38,39^ Supermeres, smaller in size (22–30 nm), are characterized by the presence of biomarkers like TGFBI, HSPA13, and ENO2. The precise molecular mechanisms that govern the formation, secretion, and functional diversity of exomeres and supermeres remain poorly understood, with their origins and release pathways still to be clarified.^40^ The distinct structural and molecular characteristics of these newly discovered entities highlight the need for further research to unravel their biological functions and explore their potential in clinical diagnostics and therapeutic applications.

### 2.2 Size

EV subtypes, such as supermeres, exomeres, exosomes, ectosomes, and apoptotic bodies, are heterogeneous in size and size overlap (Figure 3, top), which presents substantial challenges in their classification and isolation. Although these vesicles originate from distinct cellular processes, their overlapping size ranges often hinder differentiation based solely on size. Detailed analysis of each subtype highlights the complexity and limitations of size-based classification.

**Figure 3:**
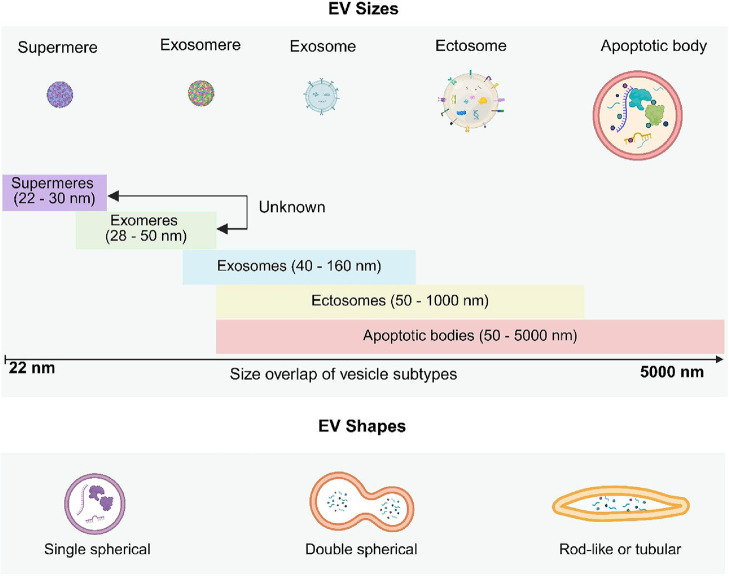
EVs range in size according to their subtype and exhibit different morphologies. Figure created with Biorender.com.

As detailed in the previous section, supermeres, typically ranging from 22 to 30 nm, represent the smallest EVs and exhibit relatively low size heterogeneity within this narrow range. Despite this apparent uniformity, their molecular composition, including small RNAs, proteins, and lipids, can introduce variability. Supermeres overlap with exomeres at the lower end of their size spectrum (approximately 30 nm). Although their cargo is primarily small RNAs, the mechanisms of supermere formation remain less understood compared to other EV types, further complicating their characterization.

Exomeres, with sizes ranging from 28 to 50 nm, display moderate size heterogeneity, primarily driven by differences in their molecular cargo, which includes proteins, lipids, and small RNAs. This subtype overlaps significantly with supermeres at smaller sizes (∼30 nm) and with exosomes at larger sizes (∼40 nm).^38,39^ Such size overlaps challenge clear distinctions between exomeres and other EV types, especially exosomes, as they also share molecular contents and functional roles.

Exosomes, ranging broadly from 40 to 160 nm, exhibit substantial size heterogeneity. Their diverse biogenesis pathways influence not only their size but also their molecular content, which may include proteins, lipids, mRNAs, and miRNAs. Their broad size range leads to significant overlap with exomeres, ectosomes, and apoptotic bodies. Moreover, the variability within exosome subpopulations reflects their functional diversity in intercellular communication, immune modulation, and disease progression. Such heterogeneity underscores the limitations of relying on size for exosome classification and highlights the importance of complementary molecular analyses.

Ectosomes, also referred to as microvesicles, are larger EVs with sizes ranging from 50 nm to 1 μm and exhibit greater size heterogeneity. This variability is indicative of the complex mechanisms underlying their formation, including direct membrane shedding. Ectosomes carry a wide array of molecular cargo and provide a substantial surface area for ligand–receptor interactions, suggesting their prominent roles in cell-to-cell signaling and immune responses.^41^ The considerable size overlap of ectosomes with exomeres, exosomes, and apoptotic bodies further complicates size-based classification efforts.

Apoptotic bodies, the largest EVs, range from 50 to 5000 nm. Their size heterogeneity arises from the fragmentation of cellular components during apoptosis, leading to significant variability in vesicle size. While apoptotic bodies often contain fragmented DNA, organelles, proteins, and other cellular debris, their size overlaps with exomeres, exosomes, and ectosomes at the lower end of their size spectrum. Despite their shared size ranges, apoptotic bodies are distinct in origin and content, which differentiates them from other EV subtypes.^20^ The pervasive size heterogeneity and overlap across EV subtypes emphasize the challenges of accurate classification and isolation based on size alone. Furthermore, these difficulties are exacerbated by variations in molecular content, which not only affect the biological functions and interactions of EVs but also necessitate the integration of size-independent markers and advanced characterization techniques for precise EV analysis.

Exosomes and other types of sEVs demonstrate remarkable efficacy in transporting cargo molecules such as microRNAs (miRNAs or miRs) and proteins to target cells, thereby modulating cellular processes including gene expression, cellular proliferation, and cellular differentiation. In contrast, lEVs, such as ectosomes, can carry more cargo and have a greater surface area for ligand–receptor interactions, suggesting their potential involvement in cell-to-cell signaling and immune responses.^41^ In essence, the heterogeneity in EV size underscores its significance in dictating a range of biological functions and interactions with recipient cells. Understanding the functional implications of EV size heterogeneity is crucial for deciphering the role of EVs in physiological and pathological processes, given that the size of EVs may reflect their cellular origin and biogenesis pathways.^42^

### 2.3 Shape

EV morphology can vary significantly depending on factors such as the source of the EVs, isolation methods, and imaging techniques (Figure 3, bottom). Typically, single spherical EVs are the most prevalent, comprising approximately 60–95% of observed vesicles, with these vesicles often being exosomes or ectosomes, characterized by their bilayer membranes. Double spherical vesicles are less common, representing 5–20% of the population, and may result from EV aggregation or fusion during isolation or storage. Additionally, rod-like or tubular EVs are seen in about 0.5–10% of cases, associated with vesicle fusion events, lipid composition, or specialized functions such as cargo transport.^43^ These morphological distributions can vary across different diseases, bodily fluids, and experimental conditions. The shape of EVs may impact their functionality and interaction with recipient cells. For example, double spherical EVs have the potential to simultaneously deliver multiple cargo types or interact with multiple receptors on target cells.^44^ Rod-like or tubular EVs, with their elongated structure, facilitate more extensive interaction with cell membranes, potentially enhancing their targeting capabilities. Understanding EV shape heterogeneity is crucial for optimizing isolation techniques, developing targeted surface modifications, and improving cargo-loading methodologies. Further exploration of the functional implications of EV shape heterogeneity promises insights that could inform the development of tailored diagnostics.

### 2.4 Content

EVs demonstrate substantial content diversity. In this section, we describe different categories of molecular cargo found within EVs and provide specific examples. It is important to recognize that not all EVs will carry the same combination of these components, as their content, which depends not only on their biogenesis and cellular origin but also on their functional roles, can vary greatly. For example, EVs carry receptors that recognize and bind specific ligands, including growth factors, hormones, and immune cell receptors, facilitating diverse cellular responses. Understanding the molecular diversity within EVs is crucial, providing insight into their functions in cellular communication and disease processes, as well as their potential for diagnostic applications. EVs can serve as disease biomarkers, as they can carry unique proteins that reflect specific patient conditions. These bioactive molecules have the potential to influence surrounding cells and can be targeted for therapeutic and diagnostic applications.^45^ Proteins generally retain their core functions when present on EVs, but their roles may differ because of the unique microenvironment of EVs. On the surface of cells, these proteins are primarily involved in direct signaling and cellular interactions.^46^ However, when associated with EVs, they play key roles in cargo sorting, targeting, uptake by recipient cells, and mediating long-range communication. Additionally, the EVs provide a protective environment for these proteins, shielding them from degradation, which helps preserve their functional integrity and enhances their potential as diagnostic biomarkers.^47^ Table 1 highlights the diversity of EV contents, their sources, functions, and potential applications across various pathological conditions. The listed proteins represent key molecular signatures that are increasingly being investigated as noninvasive biomarkers for early disease detection, disease monitoring, and treatment evaluation.

**Table 1:** EV Proteins Discussed in this Review^a^

content	source	function	pathological condition	refs
CD37	tumor, cell lines (peripheral blood mononuclear cells, hematopoietic stem cells)	diagnosis, prognosis, immune response, therapy, drug delivery	acute myeloid leukemia, colorectal cancer	^48–51^
CD44	synovial fluid, serum, plasma, cell lines (adenoid cystic carcinoma, pulmonary endothelial)	tumor metastasis, inflammation, progression, diagnosis	lung metastasis, osteoarthritis, rheumatoid arthritis, glioblastoma malignancy	^52–55^
CD47	tumor, bone marrow, tissue, cell lines (mesenchymal stem cells [MSCs], HL-60, KG-1, THP-1, Kasumi-1, MOLM-13)	tumor progression, immune response, diagnosis, therapy, prognosis	ovarian cancer, acute myeloid leukemia	^56–58^
CD53	tumor, cell lines (MSCs, HL-60, KG-1, THP-1, Kasumi-1, MOLM-13)	signaling, development, diagnosis, therapy	acute myeloid leukemia, nonalcoholic steatohepatitis, type 2 diabetes	^56,59^
CD54	serum, plasma, tissues, blood cells, MSCs	tumor progression, immune response, diagnosis, monitoring, apoptosis, inflammation	inflammatory bowel disease, gastric cancer	^60,61^
CD71	serum, plasma, small bowel mucosa, cell lines (H69AR, MRC5),	immune modulation prognosis, diagnosis	small bowel mucosa, lung cancer, spleen in malaria	^62–64^
CD82	serum, plasma, tissue	metastasis, progression, inflammation	COVID-19 progression, breast cancer	^65,66^
CD151	serum, plasma, tissue	progression, therapy	triple-negative breast cancer, lung cancer, gastric cancer	^67–69^
TSPAN6	serum, plasma, tissue, cell lines (U87, U251, A172, HUVEC, HT29, SW480, Colo205, SW620)	immune responses, progression, diagnosis, therapy	glioblastoma, colorectal cancer, lung cancer	^70–72^
TSPAN7	tissue, serum, plasma, MSCs	therapy, regulation, diagnosis	autism spectrum disorder, Huntington disease, Parkinson disease, Alzheimer disease, diabetes	^73,74^
CD9	tumor, serum, plasma, urine, cell lines (PC3, LNCaP, RWPE-1)	diagnosis, therapy, engineering, migration, metastasis	prostate cancer, kidney disease	^75–77^
CD63	serum, plasma, sweat, HIV-1 lymphadenopathy-associated virus (LAV), cell lines (J1.1_LAV_, U1_(LAV)_, Jurkat, U937)	biomarker, diagnosis, monitoring	HIV-1, autoimmune diseases, breast cancer	^75,78–80^
CD81	serum, plasma, sputum, cell lines (Vero E6, HT1080)	biomarker, diagnosis	SARS-CoV-2 infection	^81,82^
Hsc70	tissue, cell lines (MSCs, HEK293T, MCF7, PANC1, U937, 4T1)	development, apoptosis, immune modulation, therapy	renal interstitial fibrosis, Alzheimer disease	^83–85^
Hsp90	tissue, serum, cell lines (HSC-3, HSC-3-M3, Ca9–22, HO-1-u-1, SAS, HSC-2, HSC-4, THP-1, RT7)	tumor microenvironment, metastasis	hepatocellular carcinoma, oral cancer	^86,87^
MHC Class I Molecules	organoids, cell lines (PaTu-8988T, KP4, MiaPaca2, Panc 2.03, PaTu-8902, Panc1, AsPc1, HupT3 and A549, H358, HCT116, BEAS-2B)	regulation, therapy	pancreatic cancer, breast cancer	^88,89^
MHC Class II Molecules	survival, tumor, blood, cell lines (EMT6, LLC1, B16F10, ASPC-1, Capan-1, CFPAC-1, FA6, IMIMPC-2, MDA-Panc-3, MiaPaca-2, Panc-1, PT45, SUIT-2, CTC-76, CTC-102, CTC-139, T2-DP4)	tumor microenvironment, therapy	lung cancer, melanoma, bladder cancer, renal cell carcinoma, pancreatic cancer	^90,91^
αvβ3	tumor, cell lines (A375, HaCaT, MDA-MB-231, MCF 10A)	therapy, drug delivery	glioblastoma, malignant melanoma, rheumatoid arthritis	^92–94^
α5β1	tumors, fibronectin, cell lines (IMR90, HUVEC-i670, Lenti-X 293T)	cell migration, angiogenesis, prognosis	fibrotic disorders, head and neck squamous cell carcinoma, hepatocellular carcinoma	^95–97^
α4β1	serum, cell lines (MSCs, TEC, HK-2)	diagnosis, therapy, drug delivery	kidney hypoxia, cardiovascular inflammation	^98,99^
α6β4	blood, tissue, cell lines (HLE, HuH7, LX2, PANC1, Capan-1)	metastasis, progression	lung metastasis, cancer-associated fibroblasts, liver cancer	^97,100–102^
α6β1	blood, tissue, cell lines (HLE, HuH7, LX2)	metastasis, inflammation, progression	lung metastasis, breast cancer inflammation, cancer-associated fibroblasts, liver cancer	^101–103^
αvβ5	cell lines (MDA-MB-231, MDA-MB-468, MCF10A, HT29, TS576)	angiogenesis, infection, therapy	zika virus infection, liver metastasis	^101,104^
TNF-α	serum, plasma, blood cells, BCG, H37Rv, cell lines (H37Ra, RAW264.7)	therapy, inflammatory responses, immune regulation	osteoarthritis, rheumatoid arthritis, infection	^94,105,106^
Fas (CD95)	serum, plasma, blood, MSCs	apoptosis, therapy, inflammation, prognosis	multiple myeloma, Crohn disease	^107,108^
EGFR	tumor, bile, serum, plasma, blood, cell lines (SNU308, SNU478, SNU1196)	diagnosis, progression, metastasis, therapy	glioblastoma, cholangiocarcinoma, nonsmall cell lung cancer	^68,109,110^
HER2	plasma, tumor, cell lines (MDA-MB-231, SH-SY5Y, MCF7, ZR-75–1, BT-474, SK-BR-3)	diagnosis, therapy	breast cancer	^111,112^
ATPase	tissues, serum, CSF, plasma, cells line (AGS),	diagnosis, monitoring, prognosis	infection, gastric cancer, Alzheimer disease	^113,114^
GAPDH	serum, plasma, blood, cell lines (MSCs, HEK293T, SKOVE-3, B16–F10, HeLa)	apoptosis regulation, diagnosis, therapy	neurodegenerative diseases, cancer progression, metabolic disorders	^115,116^
EpCAM	serum, urine, cell lines (PC3, LNCaP, RWPE-1, OVCAR3, HO23)	diagnosis, monitoring	colorectal cancer, prostate cancer, ovarian carcinoma	^77,117^
GPC1	tumors, serum, plasma, blood, cell lines (MSCs, PANC-1)	diagnosis, prognosis, therapy	pancreatic cancer	^117,118^
MUC1	tumors, serum, plasma, bile, tissue, cell lines (MSCs, NU308, SNU478, SNU1196)	diagnosis, prognosis, therapy	breast cancer, cholangiocarcinoma	^110,119^
Rab5	cell lines (A549, EA.hy926, MSCs, human umbilical cord blood)	angiogenesis, signaling, therapy	disorders and infectious diseases	^120,121^
Rab7	tissue, plasma, raw 264.7 macrophages, adenoviruses	regulates intracellular trafficking	neurodegenerative diseases, cancer	^122^
Sytenin-1	tumor tissues, blood, plasma, cell lines (549, NCI-H1975, NCI-H226, HCC827, MCF-7, BEAS-2B, HEK293, SH-SY5Y), lentivirus	migration, invasion, metastasis, progression	metastatic lung cancer, Alzheimer disease	^123,124^
LAMP1	tissues, plasma, serum, cell lines (LN18, LN229, NCH82)	lysosomal biogenesis, monitoring	lung cancer, central nervous system diseases	^125–127^
LAMP2	plasma, fibroblasts, cell lines (induced–pluripotent stem cells (iPSC), neural stem cells (NSC))	regulation, biomarker for chaperone-mediated autophagy (CMA) activity and lysosomal function	huntington disease, Alzheimer disease	^128,129^

a The table indicates where the EV proteins have been found (source), how they have been used clinically (function), and which pathological conditions they are relevant for. The listed proteins represent key molecular signatures that are increasingly being investigated as noninvasive biomarkers for early disease detection, disease monitoring, and treatment evaluation.

#### 2.4.1 Tetraspanins

TSPANs, including many cluster-of-differentiation (CD) proteins, are transmembrane proteins widely expressed across various tissues and abundantly present in EVs, playing critical roles in their formation, stability, and function. These proteins play key roles in cargo trafficking, organizing cargo within developing exosomes, and coordinating the formation of protein complexes on EV membranes, which are essential for signal transduction, cell communication, and immune responses. TSPANs enhance the interaction between EVs and recipient cells by mediating binding, fusion, and adhesion events, which facilitate efficient cargo transfer and uptake.^12^ Through their role in signaling and adhesion as well as cell migration, they also regulate key pathological processes such as metastasis, infection, and viral entry and exit. Because TSPANs are highly abundant in EVs and specific TSPANs are associated with physiological and pathological processes, these proteins are candidates for markers of physiological processes or biomarkers of disease.

CD37, involved in the formation of TSPAN-enriched microdomains, plays a role in the organization of the cell membrane and potentially in the sorting of EV content. It is primarily expressed in immune cells, including T cells and B cells, and is involved in immune cell signaling. EVs carrying CD37 can serve as prognostic biomarkers, and potentially as therapeutic targets, for acute myeloid leukemia (AML).^130^

CD44 functions as a receptor for hyaluronic acid, playing a role in cell adhesion and movement, crucial for the interaction of EVs with target cells in cancer metastasis and inflammation.^53^ In addition, CD44 serves as a marker for cancer stem cells; EVs containing CD44 allow for the identification and tracking of these cells.^55^

CD47 is known for its immune evasion capabilities. It prevents phagocytosis by binding to signal regulatory protein alpha (SIRPα) on macrophages, helping cancer cells evade immune system clearance, prolonging their circulation. Because CD47-expressing EVs aid tumors in avoiding detection by the immune system, they could be used as markers for cancer. The presence of CD47^+^ EVs could suggest not only the presence of tumors but also their progression.^57^

CD53^+^ and CD54^+^ EVs provide insights into immune-related disorders and inflammatory diseases because of their involvement in immune cell signaling and inflammatory responses. CD53 functions in immune cell signaling and development. It has the potential to impact the creation of EVs and the incorporation of certain protein cargo. CD53 can indicate illnesses that impact the function of immune cells, such as specific leukemias and lymphomas, offering information on the immune health of individuals.^131^ CD54 (ICAM-1) helps attach EVs to target cells by binding with integrins. It is connected to immune reactions and could be involved in transmitting inflammatory signals.^132^ As a biomarker, CD54 is useful in diagnosing and monitoring inflammatory disorders, such as rheumatoid arthritis and inflammatory bowel disease. CD54^+^ EVs may also indicate the presence of cancer cells that have spread to other parts of the body, because CD54 supports the adhesion and movement of tumor cells.^133^

CD71 controls the intake of iron and plays a role in moving iron bound to transferrin.^134^ Its appearance on EVs could impact their function in iron metabolism and the transfer of transferrin to target cells. EVs containing CD71 could therefore hold potential for diagnosing and monitoring iron metabolism disorders, such as anemia and hemochromatosis. However, research specifically focused on EVs carrying CD71 remains limited.^135^ CD71 is predominantly expressed on erythroid cells, where it plays a key role in iron uptake, making it a potential biomarker for assessing iron status.^136,137^ EVs that express CD71 could also suggest elevated cellular growth, which is often seen in cancers of the small bowel mucosa and in leukemia and lymphoma.^134^

CD82 is involved in stabilizing exosomal membranes and can impact the organization and display of EV cargo. It controls cell adhesion, movement, and growth. CD82 also inhibits the spread of cancer to other organs. The number of EVs expressing CD82 can forecast cancer prognosis by disclosing the potential for metastasis; fewer CD82^+^ EVs can point to an increased chance of metastasis in cancers, serving as a useful predictor of outcomes.^138^

CD151 connects with integrins to facilitate cell adhesion and movement. It is essential for arranging membrane microdomains on EVs, impacting their targeting and uptake. It enhances cell movement and invasion, making CD151-expressing EVs potential biomarkers for highly metastatic and angiogenic cancers. Elevated levels of EVs expressing CD151 may suggest aggressive tumor behavior and a poor prognosis, helping with patient stratification for treatment decisions.^139^ Its role in the targeting and interaction of EVs with recipient cells also makes it relevant for inflammation and infectious diseases.

Additional TSPANs play essential roles in EV-mediated communication, cargo sorting, and membrane organization. They impact cellular signaling and control immune responses, having therapeutic implications for cancer, immune regulation, neuroprotection, and tissue repair.^140^ For instance, TSPAN6 controls immune responses, and detecting EVs that are TSPAN6^+^ could be beneficial in the surveillance of immune-related disorders or inflammatory conditions.^141^ TSPAN7 participates in the development of the nervous system and the creation of synapses, assisting in cell communication and plasticity.^142^ Furthermore, the accumulation of TSPAN7, along with TSPAN4 and associated cholesterol, triggers the formation of migrasomes. These specific tetraspanins are involved in the formation, upkeep, and operation of EVs, aiding in communication with target cells and influencing various physiological and pathological processes.

TSPANs have also been exploited as research tools, playing a crucial role in the isolation and analysis of SiEVs. For example, CD9, CD63, and CD81 are commonly used markers for exosomes.^26^ When these and other TSPANs are tagged with fluorescent probes, they enable the investigation of EV subpopulations, allowing for the visualization of multiple markers expressed on SiEVs and the deduction of similarities and dissimilarities among subpopulations.^143^

#### 2.4.2 ESCRT Machinery

EVs also contain ESCRT multisubunit protein complexes (including ESCRT-0, I, II, and III) and their associated proteins and lipids. As detailed in the earlier section on EV biogenesis and secretion, ESCRT-dependent exosome biogenesis relies on ESCRT-0 complexes, essential for recognizing ubiquitinated proteins and facilitating their packaging into nascent ILVs within MVBs. ESCRT-dependent exosome biogenesis also relies on ESCRT-I and ESCRT-II complexes, which begin the process of forming ILVs by triggering budding of the endosomal membrane. The last scission of EVs from the endosomal membrane is facilitated by ESCRT-III, in a process dependent on the interaction between ALIX and lysobisphosphatidic acid (LBPA).^144^ In addition to these, ESCRT complexes require other biomolecules to function, such as proteins TSG101, vacuolar protein sorting 25 homologue (VPS25), syntenin, TSPANs, Rab proteins, and integrins, as well as the lipid phospholipase D2. Thus, ESCRT complexes and their associated biomolecules regulate various aspects of EV biology, including biogenesis, size, cargo loading, trafficking, and cellular uptake.^24^ They have implications for cellular communication, cellular homeostasis, waste management, and disease conditions like neurodegeneration and cancer.

#### 2.4.3 Heat Shock Proteins

EVs may also contain heat shock proteins (HSPs), which act as molecular chaperones, mediating the proper folding and stabilization of proteins within EVs. This function is vital for maintaining protein integrity during the transport of EVs.^145^ But the importance of HSPs extends beyond maintaining protein integrity; Hsp20, Hsp27, Hsp60, Hsp70, Hsp90, and heat shock cognate 71-kDa protein (Hsc70) are essential for EV biogenesis and cargo loading. They facilitate cellular stress responses and regulate cellular processes, including apoptosis and immune modulation.^146^ In particular, Hsp90 modulates cargo protein stability and signaling pathways implicated in cancer progression and neurodegenerative disorders, underscoring its critical role in disease mechanisms.^147^

#### 2.4.4 Antigen Presenting Molecules

Ultimately derived from parent cell membranes, EV membranes can contain MHC class I molecules or, if the parent cell is an antigen-presenting cell (APC), CD8^+^ cytotoxic T cells. As part of the immune system, MHC class I molecules present endogenous antigens (derived from intracellular proteins) to CD8^+^ cytotoxic T cells, a presentation that allows the immune system to monitor and respond to infections or malignancies within cells.^148^ In contrast, MHC class II molecules present exogenous antigens (derived from extracellular proteins that have been engulfed and processed by APCs) to CD4^+^ helper T cells, a presentation crucial for activating helper T cells, which in turn stimulate other immune cells, including B cells and macrophages, to mount an effective immune response.^149^ Thus, MHC molecules are fundamental to immune system function, enabling the detection and elimination of pathogens and abnormal cells. EVs containing complexes of MHC class II molecules and antigens can transfer these complexes to APCs, enhancing T-cell activation and immune surveillance. EVs can also facilitate MHC “cross-dressing,” in which recipient cells obtain MHC molecules from donor cells, playing an important role in alloimmune responses such as those seen in transplantation.^150^

#### 2.4.5 Cell Adhesion Molecules

Integrins, transmembrane receptors crucial for adhesion between cells or between cells and the extracellular matrix (ECM), influence cell signaling and regulation as well as cell migration and invasion. When carried on the surface of EVs, integrins profoundly impact their function, uptake, and targeting abilities.^151^

The EV integrin αvβ3 influences angiogenesis, metastasis, and wound healing by interacting with ECM components. This integrin on the EV surface mediates interactions with ECM proteins such as vitronectin, fibronectin, and osteopontin, which are crucial for EV uptake by recipient cells. This interaction can promote endothelial cell adhesion and proliferation, thereby stimulating new blood vessel formation.^152^ In addition, EVs carrying pro-angiogenic factors can enhance this process by localizing these factors to areas requiring new vessel growth. In the context of cancer, the interaction between integrin αvβ3 and ECM components can facilitate the spread of tumor cells. Tumor-derived EVs (tEVs) can modify the ECM, creating a microenvironment conducive to tumor invasion and dissemination. Furthermore, EVs expressing αvβ3 can specifically target tumor cells and the tumor microenvironment, facilitating the direct delivery of therapeutic agents to tumor sites.^93^ Integrin α5β1 enhances the transfer of pro-migratory signals to recipient cells. By binding to fibronectin, it facilitates the transmission of signals that promote cell migration and invasion, which supports the spread of cancer cells to distant sites in the body, contributing to cancer metastasis. Integrin α5β1 also plays a critical role in wound healing by modulating intracellular signaling pathways such as PI3K/AKT. Through these pathways, integrin α5β1 enhances cell survival, proliferation, and migration, which are essential for effective tissue repair and regeneration following injury.^96^

Integrin α4β1 on EVs interacts with vascular cell adhesion molecule-1 (VCAM-1) on endothelial cells, playing a critical role in immune cell trafficking and inflammation. EVs displaying integrin α4β1 may enhance immune cell adhesion to the endothelial cell surface, akin to the interactions observed during inflammatory responses. This interaction between integrin α4β1 and VCAM-1 facilitates the extravasation of immune cells, which involves their migration from the bloodstream into the surrounding tissues, particularly at sites of inflammation. Thus, the integrin α4β1–VCAM-1 interaction on EVs contributes to the recruitment and accumulation of immune cells in inflamed tissues.^153^ EVs with integrin α4β1 can be engineered to deliver anti-inflammatory agents to sites of inflammation, offering a potential therapy for autoimmune diseases and inflammatory conditions.^154^

EVs expressing integrin α6β4, which primarily interacts with basement membrane laminin to influence epithelial cell behavior, can impact the epithelial-mesenchymal transition, a key process in cancer metastasis. This integrin can also activate signaling pathways such as Ras/MAPK in recipient cells, promoting cell proliferation and survival.

Integrins on EVs play a key role in organotropic metastasis, facilitating EV homing and premetastatic niche formation in target organs.^155^ For instance, integrins α6β4 and α6β1 on EVs are linked to lung metastasis; αvβ5, to liver metastasis; and α4β1, to brain metastasis. These integrin–EV signatures serve as potential biomarkers for predicting metastatic spread and could be integrated into diagnostic assays to assess metastatic potential. Furthermore, profiling EV integrins may inform personalized treatment strategies, enabling targeted therapies based on predicted metastatic patterns.^156,157^

EVs also carry selectins, transmembrane proteins that play an essential role in cell adhesion, signaling, and immune modulation. These proteins help EVs bind to target cells, thereby improving the efficiency of EV uptake.^158^ They can also mediate intercellular communication in a range of physiological and pathological situations.^159^ EVs can carry P- and/or L-selectins, which have different roles; P-selectin plays a key role in the rolling and initial attachment of platelets and leukocytes at sites of inflammation and injury, whereas L-selectin has been implicated in lymphocyte homing and adhesion to endothelial cells in peripheral lymph nodes. P-selectin has also been studied as a potential imaging biomarker and molecular target for therapeutic interventions, especially in the context of inflammatory conditions.^160^

#### 2.4.6 Growth Factors, Cytokines, and Their Receptors

EVs carry signaling molecules, including cytokines, growth factors, and their receptors to modulate the activity of recipient cells and affect physiological and pathological processes.^2,32^ For instance, EVs can carry interferons (IFNs) involved in antiviral responses and immune modulation, influencing immune cell activation and infection responses.^161^ EV receptors can also sequester cytokines and growth factors, affecting inflammation, immune reactions, and tissue regeneration. For example, EVs with receptors for tumor necrosis factor-α (TNF-α) can sequester this pro-inflammatory cytokine, modulating inflammatory responses in nearby cells.^162^ These EV-derived signaling molecules can serve as disease biomarkers. EV-associated TNF-α levels can reflect inflammatory conditions, providing diagnostic insights. TNF-α has been implicated in diseases like rheumatoid arthritis, Crohn's disease, and psoriasis, and TNF-α and IFNs are key cytokines associated with the severity of diseases like COVID-19.^163^ Other signaling molecules on EVs, such as Fas ligand and TNF receptors, can serve as biomarkers for apoptosis regulation and immune activation, and transferrin receptor can serve as a biomarker for iron metabolism disorders, as it reflects changes in iron metabolism and erythropoietic activity.^107,108,162^ EVs can also carry transforming growth factor-β (TGF-β), which regulates cellular responses and is implicated in fibrosis, tumor progression, and inflammatory diseases.^164^

In addition to cytokines and growth factors, EVs can carry receptors that affect cell signaling. Epidermal growth factor receptor (EGFR) and other receptor tyrosine kinases on EVs interact with ligands on recipient cells, initiating signaling pathways that influence cell proliferation, migration, and survival.^165^ This engagement, particularly involving EGFR, activates signaling cascades crucial for cancer progression and metastasis for cancer progression and metastasis in glioblastoma, cholangiocarcinoma, and nonsmall cell lung cancer.^68,109,110^ Furthermore, human epidermal growth factor receptor 2 (HER2), another receptor tyrosine kinase, is commonly incorporated into the membranes of EVs derived from HER2-positive cancer cells. This incorporation plays a crucial role in both the diagnosis and therapy of breast cancer.^111,112^

#### 2.4.7 Enzymes

EVs can carry a variety of enzymes, thereby contributing to diverse processes. For example, EVs can carry glycosidase enzymes, which play a critical role in carbohydrate digestion and metabolism by hydrolyzing α-glucosidic bonds in glucose polymers and β-galactosidic bonds in galactose-containing compounds like lactose.

EV-derived enzymes can serve as diagnostic markers, as their activity levels or mutations can indicate metabolic disorders, neurodegenerative diseases, or cancer. In particular, enzymes like ATPase, GAPDH, and glycerol kinase within EVs reflect the metabolic state of their parent cells and contribute to disease-associated processes.^166^ ATPases hydrolyze ATP, releasing energy for cellular processes. Pathogenic variants in *ATP1A3*, which encodes the alpha-3 subunit of Na^+^/K^+^-ATPase, can impair ATPase activity and are associated with alternating hemiplegia of childhood (AHC) and related disorders.^167^ GAPDH, while central to glycolysis, also plays a role in apoptosis, DNA repair, and RNA transport. Dysregulation of GAPDH influences cellular responses to metabolic stress and disease and is linked to neurodegenerative diseases and cancer progression.^116^ Glycerol kinase, an enzyme carried by EVs, catalyzes the conversion of glycerol to glycerol-3-phosphate, which is crucial for lipid biosynthesis. Thus, glycerol kinase impacts lipid homeostasis and membrane integrity during EV-mediated processes as well as cellular signaling. Mutations in the glycerol kinase gene can cause glycerol kinase deficiency, a rare X-linked disorder characterized by metabolic disturbances and hyperglycerolemia.^162^ The presence and activity of glycerol kinase in EVs can serve as a biomarker, reflecting metabolic disorders or diseases in the parent cells.

#### 2.4.8 Glycoproteins

Glycoproteins, characterized by covalently attached glycans, are integral components of EVs. They play pivotal roles in EV biogenesis, cargo loading, intercellular communication, and targeting of recipient cells. The glycosylation patterns of EV proteins, encompassing both N-linked and O-linked glycosylation, significantly influence EV stability, uptake, and immunomodulatory properties.^168,169^

Specific glycoproteins enriched in EVs play essential roles in modulating their biological functions and serve as valuable biomarkers for disease detection and monitoring.^170^ For example, epithelial cell adhesion molecule (EpCAM), an N-linked glycoprotein, is highly expressed in EVs derived from epithelial cancers such as colorectal cancer, prostate cancer, and ovarian carcinoma, making it a crucial marker for cancer diagnostics.^77,117^ Similarly, Glypican-1 (GPC1), another N-linked glycoprotein, is abundantly present in pancreatic cancer-derived EVs and has been recognized as a promising biomarker for early cancer detection.^118^ Additionally, Mucin-1 (MUC1), a heavily O-glycosylated protein, is found in EVs from breast cancer and cholangiocarcinoma, serving as a biomarker for noninvasively identifying tEVs.^110,119^

Lectins, a type of glycan-binding protein, are key tools for studying and understanding these glycosylation patterns. Lectins specifically recognize and bind to distinct glycan motifs on EV surfaces, providing valuable insights into the glycosylation profiles of EVs.^171^ These proteins have been employed in research to detect specific glycan signatures, such as those associated with tEVs or viral infections, highlighting their potential as glycan-based biomarkers for clinical diagnostics.^172^ This growing understanding of EV glycosylation has spurred significant interest in using EV glycan signatures as novel biomarkers for clinical diagnostics and therapeutic applications, particularly in cancer.^173^

#### 2.4.9 Cytoskeletal Proteins

Cytoskeleton components such as tubulin, actin, and cofilin are integral to cellular structure, movement, and signaling, and their presence in EVs provides insight into various cellular processes and diseases. Tubulin, a key component of microtubules, is critical for facilitating intracellular transport, maintaining cell structure, and enabling cell division.^162^ Inside EVs, tubulin can reflect cytoskeletal dynamics and cellular health, and abnormal tubulin levels can act as a marker for alterations in cytoskeletal organization associated with cellular stress responses, neurological disorders, or cancer metastasis. Actin, a ubiquitous protein essential for cell motility, structure, and signaling, plays a critical role in EV-related processes, including vesicle biogenesis, cargo sorting, and interactions with recipient cells. Actin dynamics influence the formation and release of EVs from the parent cell, the sorting and packaging of cargo into EVs, and the subsequent binding and uptake of EVs by target cells.^174^ Actin levels in EVs can mirror processes related to cellular migration, cancer invasion, wound healing, or inflammatory responses.^175^ Cofilin, which regulates actin dynamics, can signal cellular stress or injury when detected in EVs. Variations in cofilin expression or activity within EVs may also serve as a biomarker for disease conditions, including neurodegenerative diseases, cardiovascular disorders, or cancer metastasis, highlighting the importance of cytoskeletal proteins in the functional diversity of EVs, as well as their potential clinical applications.^176,177^

#### 2.4.10 Membrane Transport and Fusion Proteins

Membrane transport and fusion proteins contribute significantly to the heterogeneity of EVs, and dysregulation of these proteins is associated with various diseases, highlighting their diagnostic and therapeutic potential. These proteins, including Rab GTPases, sytenin-1, syntaxin, dynamin, annexins, and flotillins, play pivotal roles in cellular processes such as vesicle trafficking, exocytosis, membrane dynamics, and EV formation. Specifically, Rab GTPases (e.g., Rab5, Rab7, Rab11, Rab27a, Rab27b, and Rab35) regulate vesicular transport and membrane dynamics, orchestrating EV secretion and cellular uptake processes. Rab5 dysregulation affects receptor trafficking, contributing to cancer and neurodegenerative disorders, and aberrant Rab7 activity is linked to lysosomal storage disorders and infectious diseases.^178^ Sytenin-1 is implicated in cancer cell migration, invasion, and metastasis. Syntaxin is part of the SNARE complex involved in vesicle fusion and, when dysregulated, is associated with neurodegenerative diseases and insulin secretion defects in diabetes.^123^ Annexins (e.g., annexins A1, A2, and A5) and flotillins (e.g., flotillin-1 and flotillin-2) regulate membrane organization and EV-mediated signaling pathways, influencing cellular responses and disease pathogenesis.^179^

#### 2.4.11 Lysosomal Membrane Proteins

EVs can contain lysosome-associated membrane proteins 1 and 2 (LAMP1 and LAMP2), which are critical for maintaining lysosomal integrity and function.^180^ Specifically, LAMP1 (CD107a) is involved in lysosomal biogenesis, acidification, and fusion, and transformations in LAMP1 appearance or localization can indicate lysosomal dysfunction. LAMP2 (CD107b) is important for chaperone-mediated autophagy (CMA) and plays a role in lysosomal stability and membrane repair, and LAMP2 expression levels can be a marker for CMA activity and lysosomal function. Deficiencies in LAMP2 are linked to Danon disease, characterized by cardiomyopathy, skeletal myopathy, and intellectual disability.^47^ Thus, these proteins are important for cellular homeostasis and can contribute to disease pathology.

#### 2.4.12 Lipids

Lipids are fundamental structural and functional components of EVs, contributing to their biogenesis, stability, cargo transport, and intercellular communication. EV membranes are enriched in various lipids, such as cholesterol, sphingomyelin (SM), PS, phosphatidylcholine (PC), phosphatidylinositol (PI), phosphatidylethanolamine (PE), glycosphingolipids, ceramides, and the membrane lipid composition plays a critical role in EV structure, stability, and function in diagnostics and therapeutic applications.^181,182^

Cholesterol, a key component of cell membranes, is crucial for membrane fluidity, signaling, and lipid raft formation. Investigating cholesterol levels in EVs can offer insights into lipid metabolism disorders, cardiovascular diseases, and neurological conditions.^183^ SM is a critical lipid in EVs' membranes, essential for their biogenesis, structure, stability, and function. SM-enriched EVs also influence immune responses and can promote immune evasion in cancer by altering immune cell signaling.^184^ In addition, sphingomyelin-rich EVs facilitate tumor metastasis by modulating the tumor microenvironment, enhancing cancer cell migration, invasion, and immune suppression. Thus, sphingomyelin in EVs is pivotal in normal cellular functions and disease progression, especially in cancer.

PS plays a distinctive role in EV membranes, differing from its typical localization in cell membranes. EV membranes exhibit a higher concentration of PS compared to cell membranes, which enhances their uptake by recipient cells.^185^ Their composition can also reflect their parent cells, indicating physiological or pathological features.^186^ Under normal conditions, PS is primarily localized to the inner leaflet of the EV membrane; however, in pathological settings, it becomes exposed on the outer surface, acting as an “eat-me” signal for phagocytes. PS, indicating cell activation, apoptosis, and immune regulation, is relevant in cancer and autoimmune diseases.^185,187^ PC is the most abundant phospholipid in biological membranes and is essential for maintaining the structural integrity of EVs. In EVs, observing the levels of PC, an important contributor to membrane integrity and fluidity, can provide insight into liver function, lipid metabolism disorders, and cardiovascular health.^188^ PI and its phosphorylated derivatives further expand the functional spectrum of EVs by regulating cellular signaling and vesicle trafficking. PI lipids participate in cargo sorting, membrane curvature, and vesicle fusion, influencing the capacity of EVs to deliver bioactive molecules to target cells.^181^ In cancer, phosphatidylinositol 3-kinase (PI3K)-Akt signaling activation through PI-enriched EVs promotes tumor progression, implicating PI as a biomarker for cancer detection and therapy response monitoring.^189^ Furthermore, PE plays a crucial role in membrane fusion during EV release and cellular uptake, enhancing the efficiency of cargo delivery in therapeutic applications. The presence of PE in EVs also facilitates the formation of microdomains, contributing to membrane curvature, lipid packing, and vesicle stability.

Glycosphingolipids, such as gangliosides (GM), are commonly present in EVs and contribute to membrane stability and cellular interactions.^190^ GM on the EV membrane can mediate interactions with specific receptors on recipient cells, influencing cellular uptake, immune modulation, and tumor progression. In neurodegenerative diseases, EVs enriched with GM1 and GM3 have been associated with disease progression, suggesting their potential as biomarkers for early diagnosis and prognosis.^191^

Ceramides, a class of sphingolipids derived from sphingosine and fatty acids, are critical for EV biogenesis by regulating membrane curvature and facilitating vesicle formation and fusion. Beyond their structural role, ceramide-enriched EVs participate in key cellular pathways, including apoptosis and stress responses, linking them to various disease states. Elevated ceramide levels in EVs have been associated with metabolic disorders, cancer progression, and neurodegenerative diseases, highlighting their potential as diagnostic biomarkers.^192,193^

#### 2.4.13 Nucleic Acids

EVs encapsulate various RNA species, including small RNAs such as microRNAs (miRNAs), small interfering RNAs (siRNAs), small nuclear RNAs, small nucleolar RNAs, PIWI-interacting RNAs, Y RNAs, and rRNAs. Additionally, EVs contain long noncoding RNAs (lncRNAs), circular RNAs, mRNAs, precursor RNAs, tRNAs, and mitochondrial RNAs. These RNA species reflect the genetic and functional diversity of their cells of origin.^194,195^ RNA molecules play crucial roles in gene regulation, cellular signaling, and disease pathogenesis, making them valuable biomarkers for early diagnosis and therapeutic targets in clinical settings.^45^ For example, miRNAs regulate gene expression post-transcriptionally and are implicated in cancer, cardiovascular diseases, and neurodegenerative disorders.^196^ The siRNAs have therapeutic potential via RNA interference mechanisms, targeting specific genes in viral infections and genetic diseases.^197^ Circular RNAs act as miRNA sponges, influencing cancer progression and neurodegeneration.^198^ Modulating chromatin dynamics and gene expression, lncRNAs contribute to cancer biology and cardiovascular disorders.^199^ Profiles of EV mRNAs reflect the gene expression status of the parent cells, providing insights into disease progression and treatment response.^200,201^ Transfer and mitochondrial RNAs in EVs are indicators of cellular metabolism and mitochondrial dysfunction, relevant to diseases such as cancer and neurodegenerative disorders.^202^ Short noncoding RNAs (small RNA, small nuclear RNA, Y RNA, PIWI-interacting RNA, rRNA, and small nucleolar RNA) play crucial roles in RNA processing and gene regulation. These molecules are essential for protein synthesis, reflect cellular health, and have significant implications for genetic disorders and cancer biology. In addition, they may serve as biomarkers for various disease states.^203^

The RNA content of EVs differs from that of parental cells, both in terms of the types of RNA present and their relative quantities.^204^ In general, EV RNA is enriched in small RNA species, such as miRNAs, while longer RNA molecules, such as mRNAs, are often fragmented or truncated. In contrast, the RNA profile of parental cells predominantly contains intact and full-length mRNAs. In mammalian cells, the total RNA (TRNA) content spans a broad size range, from 20 to 12,000 nucleotides (nt). The majority of this RNA is composed of rRNA (rRNA), with the 18S and 28S rRNA subunits accounting for 80% to 90% of the total RNA.^205^ The presence of rRNA subunits, such as 18S and 28S, in EVs is often observed, but these rRNA molecules are frequently truncated compared to their full-length counterparts in parental cells. A key reason for these differences is the limited size capacity of sEVs, which typically accommodate around 10,000 nt of nucleic acid content. Despite this, certain EV subtypes, such as lEVs, can occasionally contain full-length mRNAs, though this is less common.^206,207^ These differences in RNA composition and size distribution underscore the importance of selecting appropriate analytical techniques for studying EV RNA content. Because of the small size and low RNA yield of individual EVs, specialized methods, such as ddPCR and next-generation sequencing technologies, are required for accurate characterization and quantification. A clear understanding of these RNA profiles is essential for deciphering the functional and diagnostic potential of EV RNAs.

Along with RNAs, EVs encapsulate DNA fragments of various types, including double-stranded DNA, single-stranded DNA, mitochondrial DNA, viral DNA, and genomic DNA. Each DNA type serves a distinct function, emphasizing the multifaceted roles of EVs in intercellular communication, physiological responses, and disease pathogenesis.^208^ Double-stranded DNA facilitates genetic exchange between cells and disease propagation, while single-stranded DNA aids in viral infection and genetic maintenance.^208^ Mitochondrial DNA signals cellular stress responses and modulates immune functions, viral DNA facilitates infection spread and immune evasion, and genomic DNA influences cellular behavior.^209^ Understanding the functional roles of DNA within EVs is crucial for elucidating their impact on health and disease and offers insight into potential diagnostic and therapeutic strategies.

### 2.5 Origin

EVs are derived from a variety of sources (Figure 4). They can be collected from a wide range of human bodily fluids, each containing a variety of cell types. These cells can be further classified into subtypes, each potentially contributing distinct functions to the EVs they release, thereby increasing the complexity of EV populations.^162^ Furthermore, human bodily fluids not only contain EVs from human cells but also those derived from pathogens or pathogen-infected host cells, adding another layer of variability.^165^ In this section, we will examine variations in EV content and function between different cell types and within specific cell types, providing examples from immune, stem, and neural cells and citing studies using in vitro and in vivo models, tissue models, or patient samples. We will also address the diversity introduced by EVs derived from pathogens, highlighting the additional complexity in analyzing EVs from human bodily fluid sources. Understanding this variability is critical for research and clinical applications.^177^

**Figure 4:**
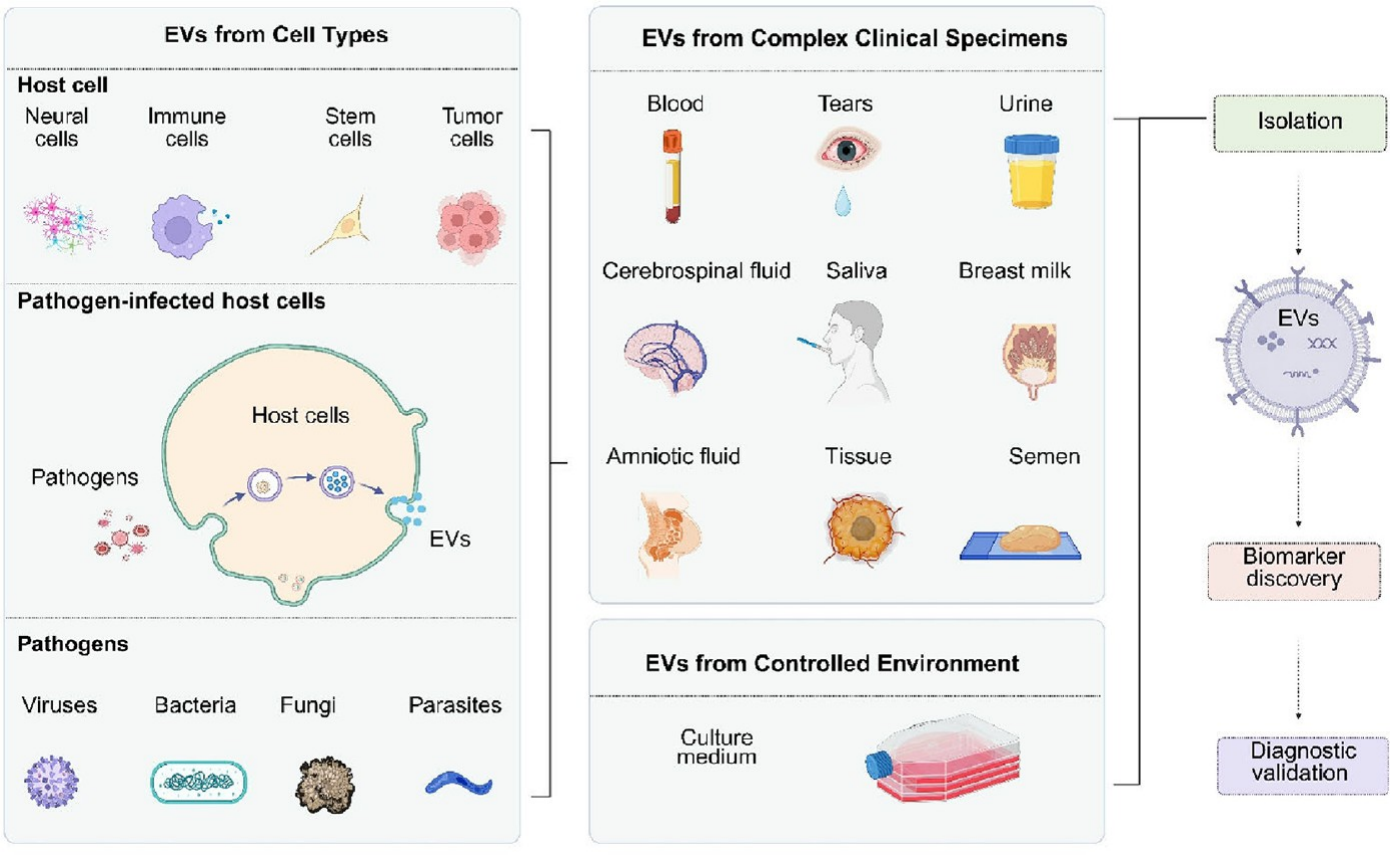
Diverse sources of EVs. EVs originate from various sources, contributing to their heterogeneity in composition and function. EVs can be isolated from cell culture media, which provide a controlled environment for studying EVs from specific cells, pathogen-infected host cells, or pathogens; or from human bodily fluids, where they represent a mixture of EVs derived from multiple cell types. This variability in EV sources plays a crucial role in their isolation, characterization, and potential clinical applications. Figure created with Biorender.com.

#### 2.5.1 EVs Derived from Cell Types

##### 2.5.1.1 EVs Derived from Immune Cells

Even when derived from one cell type, EVs display great heterogeneity, with immune cell–derived EVs being a prominent example. The great heterogeneity displayed by immune cell–derived EVs reflects the diverse functions and phenotypes of immune cells and the dynamic nature of immune responses. Immune cells encompass various subtypes, including macrophages, dendritic cells, T cells, B cells, natural killer cells, and myeloid-derived suppressor cells, each playing distinct roles crucial for immune regulation and response.^10,210^

In the form of EVs, immune cells release customized cargo, comprising proteins, lipids, nucleic acids, and other bioactive molecules, and this cargo plays critical roles in the human body. The composition of this cargo reflects the dynamic nature of cells, changing with their activation state, differentiation status, and environmental cues.^17,41^ The cargo also reflects the functions of specific immune cells, which may include immune regulation, antigen presentation, and intercellular signaling.^168,211^ For example, dendritic cell–derived EVs are enriched with MHC and costimulatory molecules, facilitating T-cell activation and subsequent immune responses.^212^ Macrophage-derived EVs transport cytokines, chemokines, and inflammatory mediators, thereby modulating immune responses and inflammation.^213^ Sometimes EVs are involved in opposing processes. For instance, EVs released by regulatory T cells suppress immune responses and promote immune tolerance, while those from APCs (e.g., dendritic cells) stimulate T-cell activation and differentiation.^10,212^ Importantly, immune cell–derived EVs have the capacity to modulate the activity and function of target cells both within and beyond the immune system, affecting not only immune cells-for example, by modulating immune cell function, promoting or inhibiting immune activation, regulating immune cell differentiation and polarization, or facilitating immune cell crosstalk-but also other types of cells in various tissues. Thus, immune cell–derived EVs can influence diverse processes, such as homeostasis, repair, infection, and inflammation, as well as the tumor microenvironment.

Given their pivotal functions, including their role in various diseases, including cancer, infectious diseases, autoimmune disorders, and inflammatory conditions, immune cell–derived EVs have important implications for diagnosis and therapy. Characterizing their heterogeneity may provide insights into disease mechanisms and identify potential biomarkers and therapeutic targets.^10^

##### 2.5.1.2 EVs Derived from Neuronal Cells

Like EVs derived from immune cells, EVs derived from neural cells display great heterogeneity. This great heterogeneity underscores the diverse functions and phenotypes of neural cells and highlights the intricate nature of neural processes and the intricate interactions that occur within the nervous system. Neural cells include neurons and glial cells, with examples of the latter being astrocytes, oligodendrocytes, microglia, and Schwann cells. Each of these cell types contributes uniquely to neural functions.^214^ For instance, neurons are central to synaptic signaling and neural communication, while glial cells are essential for maintaining neuronal homeostasis, providing structural support, regulating synaptic function, modulating immune responses in the brain, and facilitating myelination.^215^ Neurons’ interactions with glial cells, as well as their communication with other cell types in the nervous system, are critical for regulating synaptic plasticity, maintaining neural circuit stability, and responding to injury or disease.^216^

Neural cell–derived EVs serve as essential mediators of intercellular communication within the nervous system and beyond, facilitating diverse physiological and pathological processes.^217^ Neuron-derived EVs are enriched with neurotransmitters, neuropeptides, and synaptic proteins, enabling them to modulate synaptic transmission and plasticity.^7^ In contrast, glial cell–derived EVs contribute to functions such as myelination, neuroinflammation, and neuroprotection. These EVs are often enriched with proteins and molecules such as myelin basic protein (MBP), which is crucial for myelination, and various cytokines and growth factors like brain-derived neurotrophic factor (BDNF) and TGF-β, which play roles in neuroinflammation and neuroprotection.^218^ This diversity in cargo and function underscores the intricate communication network within the nervous system and the role of EVs in mediating these interactions.

Neural cell–derived EVs carry a variety of biomolecules, including proteins, lipids, nucleic acids, and other bioactive molecules, which are reflective of the physiological state and specific functions of their parent cells.^214^ The cargo of neural cell–derived EVs includes tetraspanin proteins, including CD9, CD63, and CD81, which are commonly found on the surface of EVs and involved in EV biogenesis, cargo sorting, and intercellular signaling.^217^ Also identified on the surface of neural cell–derived EVs are integrin proteins, including αvβ3 and α6β4, which facilitate EV binding and uptake by target cells;^219^ MHC proteins, including MHC class I and II molecules, which play roles in immune modulation and antigen presentation within the central nervous system;^220^ and various glycoproteins, including neural cell adhesion molecule (NCAM), L1 cell adhesion molecule (L1CAM), and EGFR, which may mediate EV binding, signaling, and uptake by recipient cells.^7^ The use of L1CAM as a biomarker for neurodegenerative and psychiatric diseases is contentious, as studies have shown it is not associated with EVs in human plasma or cerebrospinal fluid, challenging its validity as a neural cell–derived EV marker.^221^ A recent study employing proteomic profiling of EVs from human neural cells identified ATP1A3 as a robust neuron-specific marker. ATP1A3^+^ EVs isolated from plasma from individuals with Alzheimer's disease demonstrated amyloid-β positivity, providing superior diagnostic accuracy compared to conventional plasma biomarkers. These findings highlight ATP1A3^+^ EVs as a promising alternative for neurodegenerative disease diagnostics.^114^ Neural cell–derived EVs hold significant diagnostic value, with biomarkers such as Aβ42, total tau, p-T181-tau, p-S396-tau, NRGN, synaptotagmin, GAP43, SNAP25, cathepsin D, REST, and α-synuclein demonstrating consistent reliability for clinical disease diagnosis.^222^

Astrocyte-derived EVs play dual roles in central nervous system disorders, contributing to neuroinflammation and neurotoxicity while also providing neuroprotection in certain contexts. For example, neural cell–derived EVs mitigate astrocyte-induced neurotoxicity, with miR-124–3p overexpression shown to reduce neural damage effectively. The distinct protein and miRNA profiles of astrocyte-derived EVs across various physiological and pathological conditions further highlight their potential as valuable diagnostic biomarkers and therapeutic targets.^223^

Given their pivotal functions, including their role in neural communication, synaptic plasticity, neuroprotection, and modulation of neuronal activity,^224,225^ neural cell–derived EVs hold clinical relevance as potential biomarkers for neurological disorders and neurodegenerative diseases and can provide valuable insights into disease pathogenesis and progression.^226^ Notably, brain-derived EVs can cross the blood–brain barrier, as opposed to cells, and thus appear early in the pathogenesis of neurological diseases, with implications for diagnostics. Furthermore, they possess therapeutic potential as carriers for targeted drug delivery and agents for neural repair and regeneration in various neurological conditions.^7,227^

##### 2.5.1.3 EVs Derived from Stem Cells

EVs derived from stem cells also display great heterogeneity, reflecting their diverse origins and functions. Stem cell–derived EVs include those from MSCs, embryonic stem cells, induced pluripotent stem cells, and neural stem cells, and they possess a variety of regenerative and immunomodulatory properties.^217,228,229^ Additionally, even EVs from the same type of parent stem cell have heterogeneous cargo. For example, different MSC subpopulations produce EVs with distinct surface markers and protein profiles that reflect their specialized biological roles.^230,231^ Surface markers found on MSC-derived EVs include CD9, CD37, CD53, CD63, CD81, and CD82, along with specific markers like CD29, CD44, CD73, CD90, CD105, CD166, and KIT (CD117).^232^ Importantly, MSC-derived EVs lack hematopoietic antigens (CD14, CD19, CD34, CD45, and HLA-DR), reflecting their mesenchymal origin.^233^ Within MSC-derived EVs are trophic factors, cytokines, and miRNAs, PIWI-interacting RNAs, and siRNAs, which are delivered to target cells, altering their activity and function.^234,235^ EVs derived from induced pluripotent stem cells carry inhibitory miRNAs, such as those from the miR-125 family, as well as miR-126a, miR-146a, miR-199a, and miR-223, suppressing T-cell proliferation and pro-inflammatory cytokine expression.^236^ Notably, cancer cell–derived EVs may carry tumor-specific biomarkers, such as mutated proteins or oncogenic miRNAs, which can be detected in bodily fluids like blood or urine. These biomarkers are valuable for early cancer detection, disease monitoring, and therapeutic response assessment.^47^ For example, specific protein or RNA signatures in EVs can help differentiate lung cancer subtypes, providing critical insights for targeted therapies.^237^ And MSC-EVs have shown the ability to promote tissue repair and modulate immune responses, making them promising candidates for treating inflammatory diseases and tissue injuries. Furthermore, the capability of EVs to cross biological barriers and deliver therapeutic molecules positions them as potential EVs for drug delivery systems.^12,165^ EVs derived from neural stem cells also exert their effects through specific miRNAs, regulating cell growth and apoptosis.^229^ Their miRNAs can also contribute to neuroprotection. For example, miR-16 is known to contribute to the therapeutic effects of selective serotonin reuptake inhibitor (SSRI) antidepressants.^238^ Moreover, insulin-like growth factor influences the loading of miR-219a-2–3p into exosomes derived from rat neural stem cells, which suppresses YY1 expression and partially mitigates neuroinflammation, thereby enhancing neuroprotective effects following spinal cord injury (SCI).^239^ Thus, stem cell–derived EVs, through their cargo of bioactive molecules such as growth factors and miRNAs, play crucial roles in tissue repair, regeneration, and immune modulation, having relevance for tissue injury, inflammatory disorders, and autoimmune diseases.^228,240^ Continuing to build our understanding of stem cell–derived EVs in health and disease is paramount for the advancement of EV-based therapeutics and diagnostic approaches.^241^

#### 2.5.2 EVs Derived from Pathogen-Infected Host Cells or Pathogens

EV heterogeneity also arises from the presence of pathogens in the human body, with bodily fluids containing both human-derived EVs and pathogen-derived EVs. Moreover, within pathogen-derived EVs, there is notable heterogeneity; they exhibit diversity in both composition and function, playing pivotal roles in various aspects of pathogenesis, including immune evasion, intercellular communication, and establishment of infection within the host milieu.^242^ This diversity reflects the intricate strategies pathogens employ to interact with the host and manipulate its immune response, thereby shaping the progression of infection and disease.^168^ Pathogen-derived EVs come from a wide range of microbial entities, including bacteria, viruses, fungi, and parasites, and EV heterogeneity exists among these entities and also within each one.^243,244^

For example, heterogeneity exists within bacterial EVs. Bacterial EVs differ in structure, size, density, and molecular cargo composition, and these differences are due to a variety of factors: diverse biogenesis routes, including membrane blebbing, endosomal sorting, and other mechanisms; unique membrane envelope structures; and different strain-related genetic backgrounds.^245^ Bacterial EVs also differ in their production and distribution, which are dependent on bacterial species and physiological state.^18,246^ EVs also differ with respect to bacterial type; EVs are generated by both Gram-negative and Gram-positive bacteria, with EVs from each type exhibiting distinct characteristics. EVs from Gram-negative bacteria are characterized by an interior leaflet of phospholipids and an exterior leaflet of lipopolysaccharides, activating immune cells through Toll-like receptors. In contrast, EVs from Gram-positive bacteria display surface lipoteichoic acid, stimulating immune cells via Toll-like receptor 2 (TLR2).^26,127^ Bacterial EVs also have diverse functions. They are involved in bacterial survival, dissemination, and evasion of host immune responses, and they participate in crucial processes such as biofilm formation, antibiotic resistance, and manipulation of host cellular functions.^247,248^ EVs derived from pathogenic bacteria like , , and have been observed to induce mitochondrial dysfunction and immune responses in macrophages, highlighting their significance in pathological initiation.^249^ However, it should be noted that bacterial EVs can be engulfed by macrophages via several different pathways, which can influence both the macrophage immune response as well as the availability of bacterial EVs in circulation.^243,244^ Despite the complexities involved, elucidating the roles of EVs in infectious disease pathogenesis is essential. While pathogen-derived EVs in human bodily fluids have received less attention, possibly due to methodological challenges, understanding their origins and functions remains paramount. For example, the mechanisms governing bacterial EV export are poorly understood, and universal markers for bacterial cargo remain elusive. Such insights and many others will be instrumental in the development of diagnostic strategies and effective therapeutic interventions for combating various infectious diseases.^242,246,250^

In relation to this, bacterial EVs are promising diagnostic markers for bacterial infections, and accurately identifying EVs of diverse bacterial origins could provide early diagnosis, facilitate intervention, and guide treatment.^249,251^ The biomolecular cargo within bacterial EVs also holds significant potential for cancer diagnosis and therapy,^18,252^ as specific proteins, nucleic acids, and lipids encapsulated within bacterial EVs can serve as valuable biomarkers for cancer detection and monitoring, offering insights into cancer progression and characteristics.^245,246^ Furthermore, bacterial EVs can be engineered as delivery vehicles for targeted drug delivery or immunotherapy for cancer treatment, presenting opportunities for personalized and effective therapeutic interventions.^249,252^

Viral EVs are somewhat different than bacterial EVs. These EVs, often referred to as virosomes, are released during viral replication from cells infected with various viruses, such as human immunodeficiency virus (HIV), severe acute respiratory syndrome coronavirus 2 (SARS-CoV-2), herpes simplex virus, or hepatitis viruses.^253,254^ As viruses exit host cells, virosomes form during the budding process, acquiring a lipid bilayer membrane. This budding process involves the viral particles pushing through the host cell membrane, which wraps around them to form a new lipid bilayer. Viral EVs carry a cargo consisting of viral proteins, nucleic acids, enzymes, toxins, and host cell-derived components. They play pivotal roles in modulating host immune responses and viral pathogenesis.^246,251^

Fungal EVs are produced via mechanisms involving endosomal sorting and plasma membrane budding. As observed in , they share a size range similar to bacteria-derived EVs. They carry a diverse cargo, including proteins, lipids, nucleic acids, polysaccharides, and specific virulence factors, contributing significantly to intercellular communication, virulence, host–pathogen interactions, and environmental adaptation.^255^

Parasitic EVs, from helminths, protozoans, malaria parasites, and trypanosomes, are likely produced by similar processes to those of bacterial and fungal EVs, including endosomal sorting and membrane shedding, and they also fall within a comparable size range.^256^ Their cargo includes proteins, lipids, nucleic acids, and parasite-specific molecules, including virulence factors and antigens. Their roles in host cell invasion, dissemination, and modulation of host immune responses underscore their importance in infectious diseases.^250,257^

### 2.6 Sample Type

EVs can be collected from human bodily fluids, which may present important differences compared to those collected from cells cultured in media.^258^ In this section, we discuss important considerations related to working with EVs collected from human bodily fluids and EVs collected from cells cultured in media.

#### 2.6.1 EVs Collected from Specimens

EVs collected from human bodily fluids display remarkable diversity in content and function, mirroring the diversity of their originating cells. EVs can be collected from different bodily fluids, including blood, urine, saliva, solid tissues, cerebrospinal fluid, and breast milk, and the EVs collected from each of these can originate from a variety of cell types.^7,183,259^ For example, blood alone contains EVs originating from various cellular sources, including red blood cells, platelets, endothelial cells, leukocytes, and stem cells. And these EVs have different functions depending on their cell of origin. For instance, platelet-derived EVs actively participate in hemostasis and thrombosis, contributing to the delicate equilibrium required for blood clotting, while EVs derived from endothelial cells play pivotal roles in angiogenesis and vascular repair.^260^ But these are just two examples; EVs in human bodily fluids play indispensable roles in numerous biological processes.^261^ From modulating immune responses to orchestrating tissue repair and regeneration, from contributing to coagulation processes to influencing cancer progression, EVs exert significant influence over cellular behavior and gene expression.^262^ Their involvement in diverse signaling pathways associated with both health and disease underscores their importance in maintaining physiological homeostasis and driving pathological conditions.^7^

EVs collected from human bodily fluids also display diversity in their concentration, reflecting their physiological origins and disease-specific utility. Plasma typically contains the highest EV concentration, ranging from 10^7^ to 10^12^ particles/mL, making it suitable for diagnostics for systemic diseases, including cancers, cardiovascular, and neurological disorders.^206^ In contrast, urine has a lower EV concentration, ranging from 10^4^ to 10^7^ particles/mL, but provides a valuable, noninvasive source for diagnosing renal and urological conditions.^263^ Cerebrospinal fluid holds 10^6^–10^8^ particles/mL of EVs, offering critical biomarkers for neurological diseases such as Alzheimer's and Parkinson's disease.^264^ Saliva contains 10^4^–10^7^ particles/mL of EVs, serving as a convenient, noninvasive option for oral health and systemic disease monitoring.^265^ Breast milk contains 10^8^–10^9^ particles/mL of EVs, important for infant development and maternal health.^266^ Semen contains 10^4^–10^7^ particles/mL of EVs, which are involved in reproductive processes, such as sperm motility and fertilization. EVs from semen are also studied in the context of prostate cancer diagnostics.^267^ Sweat, containing 10^4^–10^6^ particles/mL of EVs, offers a lower concentration of EVs but shows potential for diagnosing skin-related diseases and conditions like cystic fibrosis.^268^ These variations in EV concentration highlight the diverse utility of different bodily fluids, with plasma and breast milk offering higher concentrations for systemic disease detection, while fluids like urine, cerebrospinal fluid, and saliva provide more localized, noninvasive diagnostic options.

The extensive heterogeneity among EVs in human bodily fluids presents both opportunities and challenges for biomedical research and clinical applications.^269^ EVs from bodily fluids provide a dynamic reflection of the physiological and pathological states of their originating cells and tissues, offering valuable insights into various health conditions.^13^ Their diverse content and functions hold the potential to reveal novel diagnostic biomarkers, drive innovative therapeutic interventions, and enable targeted drug delivery strategies, ultimately improving diagnostic accuracy and patient outcomes. However, the complexity of EV populations can make it difficult to isolate and characterize specific subtypes, and the variability in EV content across different individuals and conditions can complicate the interpretation of results. In addition, the presence of a broad range of EVs from various sources within the same bodily fluid can lead to overlapping signals and potential interference, complicating the development of precise diagnostic and therapeutic applications. Understanding these complexities and overcoming these challenges are crucial for effectively leveraging EVs from human bodily fluids for clinical applications. In contrast, EVs derived from cultured cells, as discussed in the next section, may offer more controlled and consistent profiles, which can help clarify specific cellular mechanisms and streamline the development of targeted interventions in clinical applications.

#### 2.6.2 EVs Collected from Cells Cultured in Media

EVs are released by cultured cells and collected from the cell culture medium. Their heterogeneity is influenced by factors such as cell type, culture medium composition, and environmental conditions during cell growth. These factors can impact the characteristics and contents of the EVs, making it essential to consider them when studying EVs from cell culture models.^258,270^ For instance, MSC-derived EVs vary depending on whether the cells are grown in 2D (two-dimensional) or 3D (three-dimensional) environments, with 3D cultures often enhancing both EV yield and functionality.^271^

When working with EVs secreted by cells and isolated from culture medium, one must consider several factors. Supplements used for cell growth can introduce non-EV particles (NEVs), and these particles must be removed to accurately study EV content and function.^258,271^ In addition, culture medium composition significantly influences EV size and properties.^272^ The identity of the parent cell also impacts EV yield, phenotype, and function, as cells from different sources produce EVs at varying rates and with different functional profiles. For example, in comparison to healthy cell lines, cancer cell lines tend to release more EVs and EVs with higher densities. Finally, EVs isolated from the cell culture medium generally display higher purity than those from bodily fluids.

### 2.7 Function and Applications of EVs

The heterogeneity of EVs is fundamental to their diverse functions. The cargo carried by EVs can influence recipient cell behavior in several ways, making EVs critical mediators of intercellular communication, essential for maintaining physiological homeostasis and contributing to pathological conditions. Among other functions highlighted elsewhere in this review, EVs can modulate immune responses, promote repair and regeneration, and mediate tumor progression and drug resistance. They also play an increasing role in medicine, with clinical applications such as diagnostics, prognostics, therapy, and drug delivery (Figure 5).^254,273^

**Figure 5:**
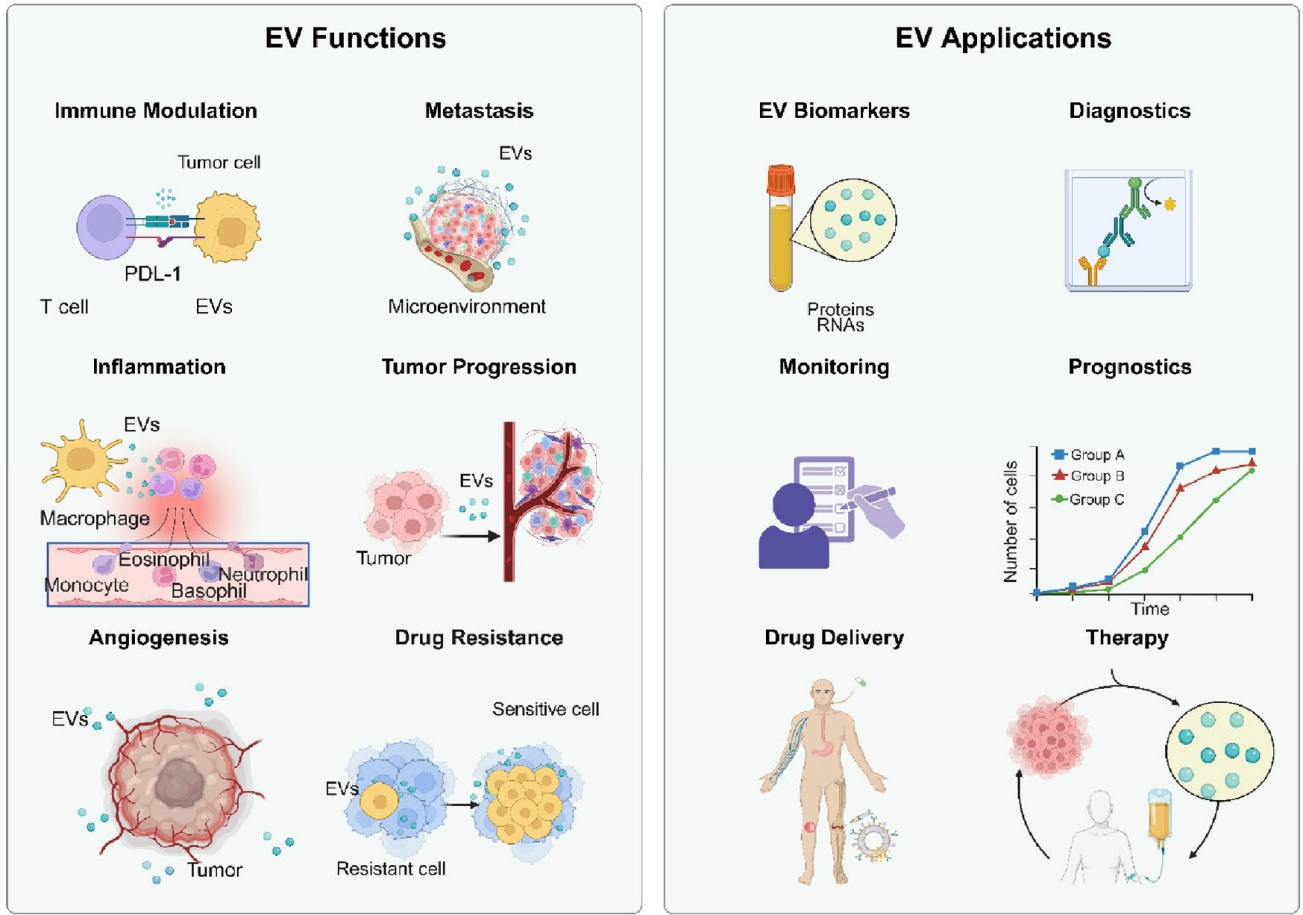
Diversity of EV functions in clinical applications. Figure created with Biorender.com.

#### 2.7.1 EVs in Immune Modulation

EVs play a crucial role in modulating immune responses through various mechanisms, influencing both the activation and regulation of immune cells.^274^ EVs can facilitate immune modulation by delivering antigens to APCs, such as dendritic cells and macrophages. APCs recognize and process “non-self” antigens through their surface receptors and then present these antigens to other cells via MHC molecules. For instance, antigens presented on MHC class II molecules are recognized by B cells, which process and display the antigens, leading to B-cell activation.^275^ Activated B cells present the antigens to helper T cells (CD4^+^ T cells), which become activated and release cytokines. These cytokines stimulate other immune cells to produce antibodies and recruit macrophages, neutrophils, and additional lymphocytes to the infection site.^276^ In parallel, cytotoxic T cells (CD8^+^ T cells) recognize and bind to antigens presented on MHC class I molecules on infected or altered cells. This interaction leads to the activation of cytotoxic T cells, which then target and destroy cells harboring intracellular pathogens. This dual mechanism—both MHC class I- and MHC class II-mediated activation ensures a comprehensive and targeted immune response against various pathogens.^277^

Recent studies highlight the immunosuppressive potential of MSCs and their sEVs, particularly in treating acute graft-versus-host disease (aGvHD). Wharton’s Jelly-derived MSCs are especially promising, secreting sEVs enriched in programmed death-ligand 1 (PD-L1), a key immune checkpoint molecule.^278^ WJMSC-derived sEVs suppress T cell receptor (TCR)–mediated activation through PD-L1, as demonstrated by in vitro experiments where blocking PD-L1 restored T-cell activity. Moreover, sEVs lacking PD-L1 failed to suppress T-cell activation, confirming its essential role. These findings position WJMSC-derived sEVs as a promising tool for immunotherapy, offering potential for both cell-based and cell-independent treatments for immune-related disorders like aGvHD. Their PD-L1–enriched vesicles provide a targeted mechanism for immune modulation, supporting their advancement in therapeutic strategies.^312^

In addition to antigen presentation, EVs can modulate immune responses through direct antimicrobial actions. Immune cell–derived EVs can carry antimicrobial peptides, enzymes, and other molecules that inhibit or kill pathogens. For example, neutrophil-derived EVs can carry enzymes like myeloperoxidase and neutrophil elastase, which degrade bacterial cell walls and neutralize pathogens. This direct antimicrobial activity helps control infections and limit pathogen spread.^277,279^

EVs also play a role in enhancing immune responses by transferring cytokines and chemokines, which promote the recruitment and activation of additional immune cells at infection sites. By transferring these signaling molecules, EVs help in orchestrating a more effective and localized immune response.^17^ Moreover, EVs can influence bacterial behavior by disrupting quorum sensing, which is a mechanism used by bacteria to coordinate gene expression on the basis of cell density. By interfering with quorum sensing, EVs can hinder bacterial biofilm formation and pathogenicity, further contributing to the host’s defense against bacterial infections.^10^ EVs are integral to immune modulation through their roles in antigen presentation, direct pathogen inhibition, and the enhancement of immune cell recruitment and activation. Their diverse functions highlight their potential as therapeutic targets and tools in managing immune-related diseases and infections.

#### 2.7.2 EVs in Repair and Regeneration

EVs promote tissue repair and regeneration by supporting cell proliferation and survival.^280^ For instance, EVs derived from MSCs contain growth factors and cytokines, such as vascular endothelial growth factor, hepatocyte growth factor, and insulin-like growth factor, which enhance the proliferation and survival of recipient cells.^281^ These factors can activate signaling pathways such as PI3K/Akt and MAPK, leading to increased cell proliferation and reduced apoptosis.^282^

EVs also play a crucial role in angiogenesis, the formation of new blood vessels from existing vasculature, essential for tissue repair and regeneration.^283^ EVs deliver pro-angiogenic factors to endothelial cells, including miRNAs, like miR-126 and miR-210, and proteins, like vascular endothelial growth factor and angiopoietin, directly stimulating endothelial cell migration, proliferation, and tube formation.^194^

EVs can also modulate the inflammatory response to create a favorable environment for tissue repair.^230,284^ For example, EVs from MSCs have been shown to carry anti-inflammatory cytokines, such as interleukin-10 and TGF-β, which can reduce the infiltration of pro-inflammatory cells and the production of inflammatory cytokines. This modulation helps to resolve inflammation and promote tissue regeneration.^285^

#### 2.7.3 EVs in Tumor Progression and Cancer Drug Resistance

EVs play pivotal roles in fueling tumor progression by modulating the tumor microenvironment and facilitating key oncogenic processes.^286^ They enhance cancer cell proliferation, induce angiogenesis, and drive epithelial-mesenchymal transitions, all of which contribute to tumor growth, invasion, and metastasis. By transferring bioactive molecules such as oncogenic proteins, RNA species, and lipids, EVs create a pro-tumorigenic niche that supports sustained cancer cell expansion and immune evasion.^287^ Beyond their role in tumor progression, EVs are also key mediators of cancer drug resistance. EVs originating from drug-resistant cells carry drug efflux pumps, receptor tyrosine kinases, and pro-survival factors, which can be transferred to recipient cells.^288^ This transfer reduces drug levels in recipient cells, triggering survival pathways, vascular restructuring, and avoidance of cell death mechanisms,^289^ facilitating the propagation of drug resistance within tumors and complicating treatment strategies.^284,290^ Consequently, EV-mediated drug resistance not only diminishes the efficacy of chemotherapy and targeted therapies but also fosters the emergence of treatment-resistant tumor populations, complicating therapeutic strategies and contributing to disease recurrence.

#### 2.7.4 EVs in Disease Detection and Monitoring

EVs are extremely valuable for disease detection and monitoring. They are abundant in human bodily fluids, such as blood, urine, saliva, and cerebrospinal fluid, and the noninvasive to minimally invasive nature of EV sampling allows for repeated measurements over time, critical for monitoring disease dynamics, such as response to therapy.^291^ EVs mediate numerous biological phenomena, significantly influencing pathological processes by eliciting responses within target cells, and they correlate with specific pathophysiological states.^292^ They mirror their parent cells and carry disease-specific markers.^273^ For all these reasons, EVs are great potential sources of biomarkers for managing a variety of diseases. For instance, tEVs carrying oncogenic proteins and miRNAs may serve as biomarkers for cancer, while immune cell-derived EVs may serve as biomarkers for inflammation or autoimmune activity. This specificity enables early disease detection, often before clinical symptoms manifest themselves.^17,293^ For example, Atay and colleagues reported that gastrointestinal stromal tumor (GIST) cells invade the interstitial stroma through the release of exosomes containing the oncogenic protein tyrosine kinase (KIT) (i.e., “oncosomes”). In these oncosomes, KIT is constitutively activated, which triggers the phenotypic conversion of progenitor smooth muscle cells to tumor-promoting cells.^294^ Furthermore, the researchers could use these oncosomes and associated exosome proteins, discovered through the first high-quality proteomic study of GIST-derived exosomes, to track disease burden in patients receiving imatinib mesylate.^295^

EV-derived biomarkers can be used to detect cancer, and these biomarkers include tumor-specific proteins, oncogenic RNA signatures, and mutated DNA. Different proteins are associated with specific types of cancer: for example, EGFR is linked to lung cancer; prostate-specific antigen (PSA), to prostate cancer; and HER2, to breast cancer, to name a few. The existence of these proteins in EVs obtained from bodily fluids can aid in the early detection of cancer. Due to the high abundance of cancer-derived EVs secreted by cells, they hold significant potential for early-stage cancer detection.^296^ In addition to distinct proteins, tEVs can carry distinct RNA molecules that have been recognized as possible biomarkers for different types of cancer, such as mRNAs, miRNAs, and lncRNAs.^297^ EVs can also contain cancer-associated DNA fragments, such as altered *KRAS*, *PIK3CA*, *PD-L1*, and *TP53* genes.

EV-derived biomarkers can also aid in the diagnosis of cardiovascular disease. EVs contribute significantly to cardiovascular disease via their molecular cargo. EVs derived from cardiac cells contain proteins indicative of myocardial injury and heart failure, including troponin and natriuretic peptides. These are traditional biomarkers for these conditions, but detecting them in EVs can enhance diagnostic accuracy. Cardiac cell–derived EVs can also contain RNAs relevant to diagnostics. Like proteins, EV RNAs encapsulated in EVs can be indicative of myocardial injury and heart failure, and EVs from patients with cardiovascular disease often contain specific miRNAs, such as miR-126, miR-133a, miR-146a, miR-155, and miR-223, which are involved in vascular and cardiac function. These miRNAs can serve as noninvasive biomarkers for cardiovascular conditions. Thus, monitoring cardiac-specific EV biomarkers in circulation provides insights into cardiovascular health and disease progression.^42^

EVs can also provide biomarkers for diagnosing infectious diseases. During infection, EVs carry pathogen-derived proteins and nucleic acids, which can serve as diagnostic markers for diseases such as hepatitis, HIV, and COVID-19.^242,243^ During viral infection of hepatitis C, EVs in the serum carry viral RNA capable of replication, along with factors like Ago2, miR122, and HSP90, which support viral replication and can also transmit the virus to other cells to facilitate infection.^298^ Viral RNA sequences have been used to help detect and monitor infections of hepatitis B and C.^299,300^ EVs from HIV-infected individuals can carry viral RNA and proteins, and detecting these components in EVs can aid in diagnosing HIV and monitoring the disease.^301^ EVs in the blood of tuberculosis (TB) patients can contain mycobacterial components, which can be used to diagnose TB and monitor treatment efficacy.^302^ In addition to pathogen-derived markers, EVs from infected individuals can carry proteins and RNAs reflecting the host immune response, such as interferons and cytokines. These can also help diagnose and monitor the progression of infections.^243^

EV-derived biomarkers can also be used to detect neurodegenerative, autoimmune, metabolic, and liver disease. In neurodegenerative diseases such as Alzheimer's and Parkinson's disease, EVs from neural cells carry disease-specific proteins (e.g., tau, α-synuclein) and miRNAs (e.g., miR-125b, miR-146a) associated with disease pathology. EVs and the cargo they carry can serve as important markers for the detection of acute ischemic stroke as well.^206^ Detection of these biomarkers in EVs holds promise for early diagnosis and monitoring of disease progression. In autoimmune diseases, EVs provide insights into disease mechanisms and serve as diagnostic tools.^303^ In type 1 diabetes, an autoimmune disease targeting the insulin-producing pancreatic β cells, EVs are crucial in transporting autoantigen peptides from β cells to APCs. This transfer can initiate or exacerbate immune responses by activating T cells, contributing to the autoimmune attack on β cells.^304^ EVs can also aid in diagnosing metabolic disorders, such as obesity and type 2 diabetes. EVs from individuals with metabolic disorders may carry specific proteins involved in metabolic pathways.^305^ For example, adipocyte-derived EVs can carry adipokines, indicative of metabolic health. Specific miRNAs in EVs, such as miR-122 and miR-192, have been associated with insulin resistance and liver function, providing potential biomarkers for metabolic conditions.^306^ Finally, in nonalcoholic fatty liver disease (NAFLD), miRNAs derived from hepatocyte vesicles are better biomarkers than miRNAs directly from hepatocytes. The reduced levels of miR-135a-3p in vesicles are linked to the progression of NAFLD and are thus regarded as a biomarker for the condition.^307^

#### 2.7.5 EVs in Therapeutics

EVs contain a phospholipid bilayer membrane, which maintains structural integrity for carrying molecular cargos and protects the endogenous or exogenous proteins and RNAs from enzymatic degradation. Therefore, EVs are excellent natural biomaterials for drug delivery.^308^ Compared to synthetic nanoparticles, EVs, with their intrinsic properties, exhibit longer circulation times and tissue penetration ability. Several factors from EVs, including size, shape, surface charge, and surface receptors, play important roles in determining their in vivo biodistribution and drug delivery performance. A few early clinical trials have evaluated the effects of autologous dendritic cell–derived EVs for cancer immunotherapy and allogeneic mesenchymal stem cell–derived EVs for regenerative and anti-inflammatory applications.^309,310^ To increase the therapeutic potency, molecularly engineered EVs have been popular for developing specific disease treatment agents.^309,311^ The broadly reported minimal side effects from the administration of EVs derived from various specific cell sources are also highly promising for utilizing EVs as safe and well-tolerated therapeutic agents.^312^ As a cell-free therapy, EVs provide a cost-effective approach with good stability and transportation, and straightforward handling and therapeutic administration, which is more advanced than their parent cells. EVs are opening new opportunities in developing therapeutic applications and clinical translation.

The therapeutic potential of EVs has garnered significant interest for diverse medical applications. One application is cancer treatment. Approaches targeting EV biogenesis, secretion, or uptake aim to disrupt tumor-promoting signaling pathways and inhibit cancer progression.^256^ EVs offer a promising approach for targeted cancer therapy. These vesicles can be engineered to deliver therapeutic agents directly to cancer cells, thereby enhancing treatment specificity and minimizing off-target effects.^313^ For instance, EVs can be modified to carry chemotherapeutic drugs or immune checkpoint inhibitors, such as PD-L1 inhibitors, which help modulate the tumor microenvironment and enhance the immune response against cancer cells.^314^ Another application is cardiovascular treatment. EVs derived from MSCs show considerable promise for cardiac repair and regeneration. Following myocardial infarction, MSC-derived EVs can promote angiogenesis, reduce inflammation, and prevent cardiomyocyte apoptosis. Moreover, these EVs exhibit anti-inflammatory effects that may benefit patients with atherosclerosis and other inflammatory cardiovascular conditions. Yet another application is treatment for neurodegenerative diseases such as Alzheimer's and Parkinson's disease, in which EVs offer a potential means of delivering neuroprotective agents and modulating disease processes.^214,218^ For instance, EVs can be engineered to carry therapeutic molecules like β-amyloid–clearing enzymes for Alzheimer's disease or neuroprotective agents for Parkinson's disease, aiming to reduce neurotoxicity and support neural regeneration.^315^ EVs are also being explored for their potential to modulate immune responses in autoimmune diseases. EVs derived from regulatory T cells (Tregs) can transport anti-inflammatory cytokines and miRs that influence immune system activity. This approach holds promise for treating conditions such as rheumatoid arthritis and systemic lupus erythematosus (SLE).^231,316^ In the realm of antiviral therapy, EVs can be engineered to deliver antiviral agents or RNA-based therapies that inhibit viral replication.^317^ For example, EVs carrying siRNA targeting HIV or SARS-CoV-2, or carrying antiviral compounds for hepatitis, have demonstrated potential in reducing viral loads and improving treatment outcomes.^318^ In metabolic disorders such as type 1 and type 2 diabetes, stem cell–derived EVs are being investigated for their ability to modulate insulin sensitivity and support pancreatic β-cell regeneration. These EVs offer promise for improving metabolic regulation and mitigating complications associated with diabetes.^319,320^

The unique attributes of EVs, such as low immunogenicity, biocompatibility, and the ability to traverse biological barriers, position them as promising candidates for a wide range of therapeutic applications. Despite the potential of EVs for therapeutics, the mechanisms underlying their therapeutic effects remain only partially understood, largely due to the inherent complexity and heterogeneity of EV populations.^187,316^ Continued research is essential to fully elucidate their mechanisms of action and to optimize their therapeutic potential.

## 3 EV Biomarkers

EVs have emerged as a promising source of biomarkers for clinical applications, offering distinct advantages over free proteins present in biofluids such as plasma and urine. Free proteins arise from various cellular processes, including secretion, enzymatic activity, and cell lysis, and include cytokines, enzymes, and growth factors, vital for physiological functions. Although free proteins play critical roles in clinical diagnostics, they are susceptible to rapid degradation by proteases, dilution in circulation, and nonspecific interactions, which limit their stability and reliability as biomarkers. Additionally, the wide dynamic range of serum proteins can obscure disease-specific changes, making detection difficult. While quantifying free proteins offers valuable insights into systemic physiological changes, it often lacks the specificity required to trace cellular origin or identify disease-specific molecular signatures.^321^ In contrast to free proteins, EV surface proteins, embedded in or attached to the lipid bilayer of EVs, offer several unique benefits that enhance their potential as biomarkers. These proteins serve as markers of their parent cells and are protected within the vesicular structure, preserving disease-specific molecular signatures and enhancing their stability compared to free proteins. EV surface proteins facilitate intercellular communication and can indicate pathological conditions, such as cancer progression or neurodegeneration. Furthermore, EVs carry additional biomolecules, including RNAs and DNAs, which further amplify their diagnostic potential.^322^ Because EVs represent specific cellular subpopulations, their protein content offers greater diagnostic specificity, making them a more reliable and informative source of biomarkers for clinical and research applications.^4^

EVs display remarkable heterogeneity, especially regarding their content, and this diverse molecular makeup offers a vast array of potential biomarkers for various diseases. These diseases include various types of cancer, as well as various types of chronic and infectious diseases (Figure 6). Knowing which EV-associated biomolecules are linked to a specific disease is critical for developing tests for early diagnosis, prognosis, and treatment response monitoring. Therefore, in this section, we discuss EV-derived biomarkers that have been discovered for various diseases. This information is also summarized in Table 2.

**Figure 6:**
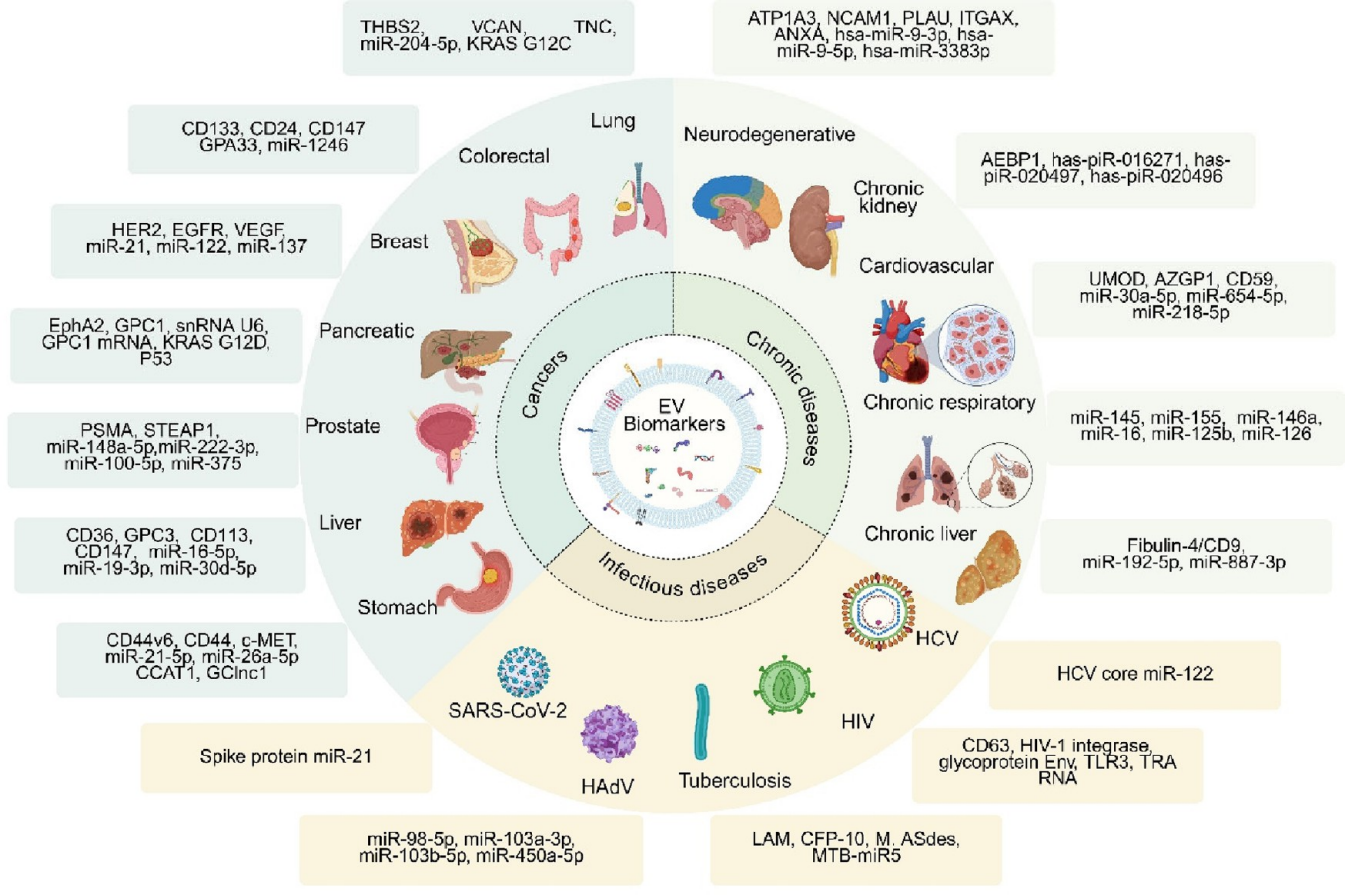
Potential EV biomarkers for disease diagnosis in clinical applications for different diseases. This figure provides an overview and reported examples of EV biomarkers linked to various diseases, including cancer types, chronic conditions, and infectious diseases. It emphasizes the clinical significance of these biomarkers in disease diagnosis, prognosis, and monitoring of treatment responses. The diversity of EV-derived biomarkers, including proteins and nucleic acids, highlights their utility in improving early detection, achieving disease staging, and predicting therapeutic outcomes across a broad spectrum of pathologies. This figure underscores the emerging role of EVs as a noninvasive diagnostic tool in precision medicine. Figure created with Biorender.com.

**Table 2:** EV-Derived Early Diagnostic Biomarkers for Various Diseases, Recently Discovered via BuEV- and SiEV-Analysis Techniques

disease type	disease subtype	biomarker category	specific biomarkers	BuEV/SiEV analysis	matrix	isolation method	detection method	function	refs
cancer	lung cancer	proteins	TNC, CD63, THSB2, VCAN	SiEV	serum, plasma, blood	DECODE chip	digital SERS (DECODE)	screening, diagnosis, prognosis, therapy	^458^
		RNAs	miR-21–5p, miR-486–5p, PD-1 mRNA, PD-L1 mRNA	BuEV/SiEV	plasma	DMF chip, Magnetic beads	RT-qPCR	diagnosis	^459,460^
		lncRNAs	H19, AL139294.1, MALAT1, ROLLCSC	BuEV	tissue culture, urine, plasma	exo kit, ultracentrifugation (UC)	qPCR	diagnosis, therapy, monitoring	^342,461^
cancer	colorectal cancer	proteins	EGFR, EpCAM, CD24, GPA33, FIBG, PDGF-β, TGF-β	SiEV/BuEV	blood, plasma, serum	magnetic beads, UC	electrochemical, CRC-EV array	screening, diagnosis	^349,462^
		miRNAs	miR-10–5p, miR-21–5p, miR-182–5p, miR-196b-5p, miR-429	BuEV	plasma	magnetic beads, UC	RT-qPCR	diagnosis	^463,464^
cancer	breast cancer	proteins	CD63, GPC1, CA 15–3, CA 125, CEA, HER2, EGFR, PSMA, EpCAM, VEGF	BuEV/SiEV	serum, plasma	UC	ELISA, TAS	diagnosis	^465^
		miRNAs	miR-21, miR-122, miR-137	SiEV	blood, plasma	UC	TIRF	diagnosis	^466^
		lncRNAs	*MALAT1/POSTN*	BuEV	blood, plasma	UC	RT-qPCR	prognosis	^467^
cancer	pancreatic cancer	proteins	MUC1, EGFR, EPCAM, CD63, GPC1, HER2, ApoA2, ITGαv, ITGβ5	SiEV	Serum, Plasma, Tumor	UC, magnetic beads	Co-PAR	diagnosis, monitoring	^374,378^
	pancreatic cancer	RNAs	miR-10b, miR-141–3p, miR-200a-3p, miR-200b-3p, miR-200c-3p, miR-429, snRNA U6, GPC1 mRNA	BuEV	serum, plasma	UC, TEI kit	RT-qPCR/TIRF, Co-PAR	diagnosis, prognosis	^118,378,379^
		gene mutations	*KRAS*, *P53*	BuEV/SiEV	plasma	UC, SEC	fluorescence microscope, simoa	diagnosis	^369,370^
cancer	prostate cancer	proteins	STEAP1, EpCAM, CD9	BuEV	serum, plasma, urine	UC, SEC	nFC, CLIA	diagnosis	^77,386^
		RNAs	miR-148a-5p, miR-21–5p, miR-181a-5p, miR-222–3p, miR-100–5p, miR-375, PSA mRNA	BuEV	serum, urine, blood	UC, magnetic beads	DSA, DTTA, NGS, RT-qPCR	screening, diagnosis	^389,468,469^
cancer	liver cancer	proteins	CD36, CD63, GPC3, EpCAM, CD113, CD147, ASGPR1, TENM2, ITGA1, DSC1, TIMP2, MUC1	BuEV/SiEV	plasma, blood	UC, magnetic beads	RT-qPCR, PBA	diagnosis	^392,395^
		miRNAs	miR-16–5p, miR-19–3p, miR-30d-5p, miR-223–3p, miR-451a	BuEV	plasma	ExoQuick kit	RT-ddPCR	diagnosis, prognosis	^394^
		lncRNAs	SFTA1P, HOTTIP, HAGLROS, LINC01419, HAGLR, CRNDE, LINC00853	BuEV	serum	UC	RT-qPCR	diagnosis	^395^
cancer	gastric cancer	miRNAs	miR-21–5p, miR-26a-5p	BuEV	serum	UC	RT-qPCR	diagnosis, prognosis	^396^
		lncRNAs	CCAT1, GClnc1	BuEV	plasma, serum	UC	RT-qPCR	diagnosis	^397,470^
cancer	bladder cancer	proteins	MUC-1, CCDC25, GLUT1	BuEV	urine, tissue	UC	ELISA, immunoassays	diagnosis	^188^
cancer	melanoma	proteins	MCSP, MCAM, LNGFR, ErbB3, PlLD, PD-L1	BuEV	plasma	UC	FCM, SERS nanotag	diagnosis, monitoring	^409,471^
		miRNAs	miR-1180–3p	BuEV	plasma	ExoRNeasy kit	qPCR	diagnosis	^410^
cancer	ovarian cancer	proteins	EpCAM, CD24, VCAN, HE4, TNC	BuEV/SiEV	serum, plasma		ELISA, SAViA	diagnosis, prognosis	^400^
		miRNAs	miR-141–3p, miR-200c-3p	BuEV	serum	UC	RT-qPCR	diagnosis, prognosis	^401^
chronic diseases	coronary artery disease	proteins	UMOD	BuEV	urine	UC	ELISA, MS	diagnosis	^412^
	vesicular vardiovascular valcification	lipoproteins	lipoprotein(a)	SiEV	serum	UC	fluorescence imaging	progression, therapy	^419^
	cardiac fibrosis	miRNAs	miR-218–5p	BuEV	plasma	N/A	RT-qPCR	diagnosis, therapy	^418^
neurodegenerative diseases	Alzheimer disease	proteins	ATP1A3, NCAM1, L1CAM, PLAU, ITGAX, ANXA1	BuEV/SiEV	human neurons, brain tissue, CSF, plasma	UC, ExoQuick kit	ELISA, MS, simoa, PBA	diagnosis	^114,424^
	Alzheimer disease	miRNAs	hsa-miR-9–3p, hsa-miR-9–5p, hsa-miR-338–3p		plasma	ExoQuick kit	RT-qPCR	diagnosis, monitoring	^423^
neurodegenerative diseases	frontotemporal dementia	proteins	TDP-43, 3R to 4R tau ratio	BuEV/SiEV	CSF, plasma	UC	ELISA, SIMOA	diagnosis	^429^
	neurodegenerative and other brain diseases	proteins	L1CAM/β-III-tubulin, GAP43, VAMP2	SiEV	blood	UC	FCM	diagnosis	^472^
chronic liver diseases	cirrhosis	proteins	Fibulin-4/CD9	BuEV	serum	MagCapture exosome isolation kit	ELISA, MS	prognosis	^473^
infectious diseases	hepatitis C virus	proteins miRNAs	HCV core miR-122	BuEV/SiEV	serum, plasma	UC	ELISA, RT-qPCR	diagnosis, monitoring	^447,474^
infectious diseases	SARS-CoV-2	proteins miRNAs	spike protein miR-21	BuEV/SiEV	serum, plasma	magnetic beads, UC	ELISA, RT-qPCR	diagnosis, monitoring	^451,452^
infectious diseases	tuberculosis	proteins	LAM, CFP-10	BuEV	serum, urine	UC	ELISA, RT-qPCR	diagnosis	^454^
infectious diseases	tuberculosis	sRNAs	M. ASdes, MTB-miR5	BuEV	serum	UC	RT-qPCR	diagnosis	^203^

### 3.1 EV Biomarkers in Cancer

EVs are a significant source of potential biomarkers for a wide range of cancers, given their ability to carry specific molecules reflecting the molecular signature of tumor cells.^323^ Characterized by runaway cellular proliferation and distribution, cancer encompasses over 200 different subtypes.^324,325^ EVs released by cancer cells contain various molecules, such as proteins, mutated genes, miRNAs, and lncRNAs, that are critical for pathogenesis, promoting tumor growth, angiogenesis, or metastasis.^259^ EVs collected from blood, urine, or other bodily fluids that contain these biomolecules can serve as biomarkers for early cancer diagnosis.^196,234,273^ Detecting cancer at an early stage generally provides more effective treatment options and leads to better outcomes, including a better quality of life and higher survival rates.^326^ Cancer therapeutics may also result from targeting EVs; therapeutics could inhibit EV release, or alternatively, EV cargo could be modified to deliver therapeutic agents directly to cancer cells. Knowing which biomarkers are relevant for which types of cancer is critical for designing early cancer diagnostics and perhaps treatments. Therefore, in the following sections, we describe EV biomolecules associated with various types of cancer: lung, colorectal, breast, pancreatic, prostate, and liver cancer, as well as stomach, bladder, ovarian, and skin cancer.

#### 3.1.1 Lung Cancer

Biomarkers are crucial for multiple aspects of lung cancer care, for initially diagnosing the cancer, predicting the prognosis, assessing the disease progression, metastasis, and monitoring the treatment response.^183,327^ EVs could be a source of these biomarkers, as EVs originating from tumor cells possess unique biomolecules that reflect the characteristics of the tumor and also contribute to pathogenesis.^328^ As one example of the latter, EVs are thought to transfer biomolecules from tumor cells to healthy cells, facilitating metastasis and the establishment of metastatic niches.^329^

Specific proteins carried by EVs are associated with lung cancer and may serve as biomarkers. Proteins carried by SiEV, as well as mRNA cargo, have shown exceptional diagnostic utility in identifying non-small cell lung cancer (NSCLC) and predicting patient responses to immunotherapy. Surface proteins programmed cell death–protein 1 (PD-1) and programmed death–ligand 1 (PD-L1) on EVs, along with PD-1 and PD-L1 mRNA cargo within EVs, were detected with SiEV resolution, surpassing the sensitivities achieved by conventional BuEV analysis methods.^200^ Proteins like carcinoembryonic antigen (CEA) and PD-L1 are specific to lung adenocarcinoma, a type of NSCLC, and are linked to either the presence of tumors or the evasion of the immune system.^200,330^ Another potential protein biomarker is EGFR, frequently found in high levels or with mutations in lung cancer cells. EGFR mutations were detected in tissue or plasma samples from 87.3% of patients with stage IIIB lung cancer and 89.7% with stage IV, and EGFR has been identified on EV surfaces.^331^ Molecular profiles of SiEVs, including CD63, THBS2, VCAN, and TNC, correctly classified individuals with early-stage malignant lung lesions (stages I and II), benign lung lesions, and healthy participants. However, challenges include SiEV heterogeneity and the low abundance of cancer-specific SiEVs in circulation, necessitating highly sensitive technologies for multibiomarker detection to advance SiEV analysis in lung cancer screening.^332^ Mutated genes can also serve as biomarkers for various types of cancer, examples being mutated forms of *MET*, *BRAF*, *ROS*, *ALK*, and *KRAS*.^333^ *KRAS* mutations are frequent in lung adenocarcinoma, especially among smokers. EVs containing *KRAS G12C* mutations serve as important biomarkers for diagnosing and monitoring *KRAS* mutant lung cancers.^333,334^ EVs containing lymphoma kinase (ALK) fusion transcripts can indicate ALK^+^ lung cancer, which means enhanced therapeutic efficacy for a subset of NSCLC patients.^335^ In lung cancer, mutations such as *EGFR* (e.g., L858R, T790M) and *KRAS* (e.g., G12C) have been detected in both cancer cells and tEVs, with the primary exosomal source being mRNA, which reflects active gene expression and tumor dynamics. While exosomal gDNA may also harbor these mutations, mRNA is more commonly studied because of its diagnostic and prognostic relevance.^336,337^ For example, mutations in exosomal *EGFR* mRNA are widely used to monitor treatment responses to tyrosine kinase inhibitors (TKIs) and identify resistance mutations such as T790M, while G12C mutations in exosomal *KRAS* mRNA are indicative of treatment resistance and disease progression.^338^

Specific EV miRNAs and lncRNAs are associated with lung cancer and may serve as biomarkers. EVs from patients with NSCLC overexpress specific miRNAs, namely miR-21, miR-29, miR-146a, miR-155, miR-210, and miR-598, which predict poor prognosis.^339^ And miR-210 TIMP-mediated ephrin-A3 reduction in endothelial cells stimulates angiogenesis and can be regulated by cancer-derived EVs. EV-derived miR-21 can distinguish patients with early-stage lung cancer from healthy individuals, facilitating early diagnosis.^340^ In addition to miRNAs, some EV-derived lncRNAs have been identified as potential diagnostic and prognostic indicators for lung cancer, specifically for NSCLC, because of their association with aggressive traits, metastasis, and tumor progression. These lncRNAs include metastasis-associated lung adenocarcinoma transcript 1 (MALAT1), HOX transcript antisense intergenic RNA (HOTAIR), and SOX2 overlapping transcript (SOX2OT).^341^ Distinguishing patients with NSCLC from healthy individuals may also be achieved by detecting simultaneous changes in miRNAs and lncRNAs, as more aggressive NSCLC characteristics have been associated with reduced levels of EV miR-204–5p and elevated levels of EV lncRNA AL139294.1.^342^

HCC metastasis, the lncR-TSPAN12, and the lncR-TSPAN12-EIF3I/SENP1 axis have been identified as a novel therapeutic target for HCC. The lncRNA H19 is involved in tumorigenesis, metastasis, and chemoresistance. NSUN2 can modify H19 lncRNA through m5C methylation, and H19 has been proposed as a potential gene therapeutic target for adjuvant therapy in chemotherapy-treated patients. Additionally, the discovery of the LCAT3-FUBP1-MYC axis provides a new potential therapeutic target for lung adenocarcinoma.^343^

#### 3.1.2 Colorectal Cancer

Many EV-derived biomarkers for colorectal cancer have been identified, relevant for the metastasis, chemoresistance, and recurrence of colorectal cancer and aiding its diagnosis, prognosis, and treatment.^344^

EVs originating from colorectal cancer cells commonly have proteins such as CD133, CD24, EpCAM, and CD147, which indicate cancer progression when identified in blood samples.^345^ CD133-expressing EVs have been found to enhance tumor development and spread in colorectal cancer,^346^ and CD24 and EpCAM are found in EVs and are specifically associated with the early stages of the disease.^347^ Recent studies have identified chemokine (C-X-C motif) ligand 4 (CXCL4) as a crucial factor in the diagnosis and prediction of colorectal cancer, and an analysis of serum from 749 colorectal cancer patients showed EV-derived CXCL4 to be a potential tumor.^348^ Moreover, surface proteins on EVs, including FIBG, PDGF-β, and TGF-β, have emerged as potential diagnostic biomarkers for noninvasive detection of colorectal cancer. To validate these markers, a colorectal cancer–EV array model was developed, employing a machine learning algorithm to detect colorectal cancer with high diagnostic accuracy. The model demonstrated superior performance, achieving an area under the receiver operating characteristic (ROC) curve (AUC) of 0.88 in the training set and 0.94 in the test set. Furthermore, the expression levels of these EV surface proteins were assessed in a multicenter study involving 404 individuals, providing robust evidence for their clinical relevance as biomarkers.^349^

EVs extracted from colorectal cancer cells found in blood also contain fragments of DNA or RNA that reflect mutations in genes such as *KRAS*, *APC*, *p53*, *PIK3CA*, and *BRAF*, which can potentially act as biomarkers for specific forms of this type of cancer.^344,350,351^ One form of colorectal cancer is due to the *KRAS* G12C mutation, reported in around 40–50% of cases and linked to a lack of response to anti-EGFR treatments like cetuximab and panitumumab.^352^ Recent research demonstrated that Rab13 plays a part in the secretion of EVs from colorectal cancer cells with a mutated *KRAS* gene.^353^

In addition to proteins and mutated gene fragments, EV-derived miRNAs, examined in samples of colorectal tissue or blood, have significant potential for the early diagnosis of colorectal cancer. Key miRNAs, such as miR-21, miR-23a, miR-150, miR-223, miR-1229, and miR-1246, are markedly overexpressed in patients with colorectal cancer compared to healthy individuals. Among these, miR-21, miR-23a, and miR-1246 have shown particularly high diagnostic accuracy, with AUC values approaching 1, and EV-miR-1246, enhanced by a dominant *P53* mutation in colorectal cancer, contributes to ECM degradation, a critical factor in cancer malignancy.^350,354^ In addition to miR-21, miR-23a, and miR-1246; miR-150–5p also shows promise as a diagnostic and prognostic marker, with an AUC value of 0.87. Other miRNAs relevant to colorectal cancer have been identified. In a comprehensive study involving 452 patients with colon cancer (stages I through III), tissue microarrays were constructed from tumor tissues. This investigation revealed that elevated levels of EV-derived miR-17–5p and miR-20a-5p were associated with favorable disease-specific survival outcomes.^355^ In addition, miR-221 in EVs derived from patient plasma, when detected along with miR-21, demonstrated 100% sensitivity and specificity for colorectal cancer detection.^356^ Another study identified a 10-miRNA biomarker panel from the tissue and serum of 77 colorectal cancer patients. Notably, EVs from patients with stage II and III colorectal cancer exhibited high levels of miR-92a-3p and miR-221–3p, with AUC values of 0.83 and 0.79, respectively.^357^ These studies highlight the potential of EV-derived miRNAs to enhance diagnostic accuracy and improve patient outcomes through early detection and targeted treatment strategies.

Finally, EV-associated lncRNAs such as HOTAIR and MALAT1 may serve as biomarkers for the diagnosis and prediction of colorectal cancer.^358^ SNHG3 may be another option, as this EV-derived lncRNA was found to promote metastasis in colorectal cancer by enhancing the RNA stability of β-catenin through HNRNPC-mediated mechanisms.^359^

#### 3.1.3 Breast Cancer

Breast cancer is the most common carcinoma among women and the second leading cause of cancer-related death worldwide.^360^ Recently, EVs have gained attention as potential sources of biomarkers for breast cancer. These vesicles carry molecular cargo, including proteins, RNA, and lipids, from their host cells, and higher levels of breast cancer-associated molecules have been identified in the bloodstream of breast cancer patients, suggesting their potential as valuable biomarkers for detection.^361^

EVs derived from breast cancer cells contain elevated levels of proteins such as HER2, EGFR, CA 125, CA15–3, PSMA, EpCAM, and MUC1, which are commonly found in breast cancer patients and may serve as EV-derived biomarkers. Another possibility is the EV-derived protein integrin α6β4, which could detect early progression of breast cancer in plasma with 85.7% sensitivity and 83.3% specificity.^362^

EVs can also contain mutated breast cancer genes, which may serve as helpful biomarkers. Mutations in genes such as *TP53*, *PIK3CA*, *BRCA1*, and *BRCA2* can be identified in EV DNA from breast cancer patients, and breast cancers caused by mutations in *BRCA1* and *TP53* may be targeted through strategies aimed at reactivating mutant p53. Thus, EV-derived DNA fragments may be helpful for treatment decisions.^363^

Exosomal mRNA has emerged as a promising biomarker for molecular subtyping of breast cancer, offering a noninvasive method.^364^ miRNAs such as miR-19a, miR-21, miR-24, miR-105, miR-155, miR-181b, and miR-210 have been recognized as promising biomarkers for breast cancer diagnosis.^365^ The use of miR-375 and PD-L1 mRNA as endogenous biomarkers has demonstrated high accuracy and selectivity in diagnosing breast cancer.^366^ Another potential biomarker for breast cancer is miR-660, which originates from breast cancer tumor–associated macrophages within the breast cancer microenvironment. This miRNA is encapsulated in EVs, which are involved in promoting metastasis by enhancing the invasive capacity of breast cancer cells. EVs encapsulating miRNAs can be expelled into bodily fluids and identified in the early stages of breast cancer in patient circulation, serving as prognostic indicators.^367^

Finally, current studies are investigating lncRNAs such as HOTAIR, SNHG14, and H19 found in EVs as potential indicators for breast cancer diagnosis. HOTAIR is upregulated in breast cancer cells, present in EVs, and associated with cancer progression and metastasis. Exploring the presence of HOTAIR and other lncRNAs in EVs from bodily fluids may lead to noninvasive diagnostic and prognostic tools for breast cancer.^368^

#### 3.1.4 Pancreatic Cancer

Pancreatic cancer is a serious disease that occurs when malignant cells form in the pancreatic tissues and typically shows no symptoms until it reaches an advanced stage, which makes it challenging to treat. CA19–9 serum cancer antigen is often used as a marker for detecting pancreatic cancer, but its limited ability to accurately detect early-stage disease or to differentiate pancreatic cancer from benign conditions makes it unsuitable for widespread screening of asymptomatic patients.^369–371^ Therefore, research is being conducted on EVs from pancreatic tumor cells to find biomarkers for the early detection of pancreatic cancer.^372^ EVs derived from these cells carry biomarkers such as proteins, mutated genes, and miRNAs that mirror the aggressive characteristics of the disease.^373^

EVs contain proteins that may serve as biomarkers for pancreatic cancer. Several proteins derived from pancreatic cancer cells, such as GPC1, EpCAM, Ephrin type-A receptor 2 (EphA2), MUC1, and EGFR, are frequently detected in EVs from pancreatic cancer patients and are being investigated as potential diagnostic biomarkers in clinical studies.^361,374–376^ In EVs isolated from patients with pancreatic ductal adenocarcinoma (PDAC), the expression levels of GPC1 and EpCAM, as well as ITGαv and ITGβ5, show a strong correlation with the disease. EV-derived apolipoproteins may also make good biomarkers for the early detection of pancreatic cancer, as a clinical study of a blood biomarker composed of apolipoprotein A2 isoforms has shown promising results. The point estimate of the AUC to distinguish pancreatic cancer (*n* = 106) from healthy controls (*n* = 106) was higher for apoA2-ATQ/AT (0.879; 95% confidence interval [CI], 0.832–0.925) than for CA19–9 (0.849; 95% CI, 0.793–0.905), meeting the primary end point of the study.^377^ EV proteins can also be analyzed in combination with EV RNAs for diagnosis. A recent study analyzed 3 protein markers (GPC1, CD63, and HER2) and 3 RNA markers (snRNA U6, GPC1 mRNA, and miR-10b) in EVs from 30 human plasma samples. The combination of these 6 biomarkers achieved a diagnostic accuracy of 92.9%, significantly outperforming both 3-biomarker combinations and individual biomarkers, suggesting that this multibiomarker approach holds great promise for early disease diagnosis.^378^

The analysis of EV-associated DNA can reveal genetic alterations associated with pancreatic cancer and aid in diagnosis and monitoring. Studies indicate that approximately 40% of EVs derived from PDAC cells contain mutations in *KRAS* and/or p53. Mutant RAS can impact proteins related to EV release, whereas RAS downstream targets regulate EV secretion in mammary tumor cells. SiEV can be used to detect frequent *KRAS* mutations in PDAC through the analysis of EVs. Mutant *KRAS* and p53 proteins are associated with the release of EVs and can be identified in SiEVs, allowing for the detection of stage 1 pancreatic cancer.^370^ Furthermore, the examination of multiple EVs simultaneously improves the accuracy of diagnosis for PDAC. Evaluating disparities in EV mutations, in conjunction with the expression profiles of proteins including CDKN2A, SMAD4, and GNAS, has shown promise in the context of clinical trials focusing on patients undergoing targeted inhibitor therapies.

Furthermore, miRNAs may also serve as biomarkers for pancreatic cancer, as they are commonly dysregulated in the disease.^370^ Plasma sEV miR-664a-3p has emerged as a highly accurate biomarker for predicting PDAC when used in conjunction with CA19–9. Elevated levels of plasma sEV miR-664a-3p are significantly correlated with vascular invasion, lower surgical success rates, and poor differentiation in PDAC patients. This biomarker holds strong potential for improving the accuracy of PDAC diagnosis and offers valuable insights into disease progression and prognosis.^199^ EV miRNA families have also been used for PDAC detection. One study showcases significant progress in PDAC diagnostics by identifying and validating EV-miR-200 family members (miR-141-3p, miR-200a-3p, miR-200b-3p, miR-200c-3p, and miR-429) as reliable biomarkers. Utilizing small RNA sequencing and reverse transcription–quantitative real-time PCR (RT-qPCR), the researchers observed substantial upregulation of these markers in PDAC compared to benign conditions, with combined EV-miR-200 family expression achieving an AUC of 0.823. Independent validation confirmed strong diagnostic performance, achieving 100% sensitivity, 88% specificity, and an AUC of 0.97 for PDAC detection. The small RNA sequencing employed to identify PDAC-specific markers, further validated across diverse patient cohorts using RT-qPCR and logistic regression models, ensured robustness across 95 patients, including patients with cholangiocarcinoma. This approach highlights its potential as a noninvasive diagnostic tool with broad clinical applicability.^379^ EV-derived miRNA has also been used to differentiate PDAC from benign pancreaticobiliary disease. This signature includes miR-141-3p, miR-200a-3p, miR-200b-3p, miR-200c-3p, and miR-429, which can serve as novel biomarkers in the plasma EVs of patients with PDAC and cholangiocarcinoma (CCA). The EV-miR-200 family members (miR-200a, miR-200b, miR-200c, miR-141, and miR-429) produced a diagnostic miRNA signature with an AUC of 0.823 (95% CI, 0.717–0.928). The addition of CA19–9 improved the diagnostic accuracy to 0.997 (95% CI, 0.989–1.000), although this result was affected by data collection limitations. The EV-miR-200 family model was tested in an independent clinical validation cohort (*n* = 32 PDAC vs *n* = 30 benign), and it predicted PDAC with a sensitivity of 100%, a specificity of 88.2%, a negative predictive value (NPV) of 100%, a positive predictive value (PPV) of 88.7%, and an AUC of 0.97 (95% CI, 0.925 to 1.000; *P* < 0.0001). This model has been identified in EVs extracted from plasma samples of pancreatic cancer patients and has been associated with the prognosis of the pancreatic cancer.^379^

Finally, a recent study investigated the potential of combining different EV biomarkers for the diagnosis and staging of PDAC. Key tumor-associated biomarkers analyzed included circulating cell-free DNA (ccfDNA) concentration and *KRAS* mutations (G12D, G12V, and G12R), which occur in approximately 90% of PDAC cases.^380^ In addition, EV-associated protein markers such as CD63, CK18, GAPDH, H3F3A, KRAS, and ODC1 demonstrated strong utility in differentiating patients with metastatic PDAC from healthy controls.^381^ The diagnostic performance of these EV-based markers was compared to that of CA19–9, a commonly used human serum protein. The findings highlight the potential of EV biomarkers to enhance cancer diagnostics, suggesting their role in a multimodal approach that enables more accurate detection and characterization of PDAC across different stages.^367^

#### 3.1.5 Prostate Cancer

Prostate cancer, a complicated disease distinguished by abnormal cell growth in the prostate gland, is the most common cancer in men. Although it frequently remains asymptomatic in its early stages, it can advance to more severe stages with symptoms such as urinary difficulties, pelvic discomfort, and erectile dysfunction, and is ranked as the second highest cause of cancer-related death.^382,383^ Thus, prostate cancer remains a significant global health concern. Screening and monitoring have been accomplished by testing for PSA, but this biomarker cannot distinguish between less aggressive forms of the disease and more aggressive forms, leading to the overdiagnosis of nonaggressive cases and highlighting the need for additional biomarkers.

Recent research has identified certain EV-related proteins as possible diagnostic and prognostic markers for prostate cancer, namely, prostate-specific membrane antigen (PSMA), EpCAM, CD9, and STEAP1.^384,385^ In one study, researchers found elevated levels of PSMA in EVs derived from prostate cancer cells compared to those derived from benign prostatic tissue. The increase in PSMA levels within EVs correlated with disease progression, suggesting its potential as a biomarker for early detection and for monitoring therapeutic responses. Moreover, the study highlighted the dual role of PSMA, positioning it not only as a diagnostic marker but also as a promising target for therapeutic interventions, especially in the development of prostate cancer–specific treatments.^385^ In another study, efforts were made to enhance prostate cancer diagnosis by developing and validating a multivariate diagnostic model centered on urinary EVs positive for EpCAM and CD9. The study involved urine samples from 193 participants, comprising 112 pancreatic cancer patients, and the diagnostic model had a high AUC of 0.952.^77^ Yet another study examined STEAP1, known for its enrichment in prostate cells and particularly in prostate cancer. STEAP1^+^ EVs in the plasma of healthy males and prostate cancer patients were characterized and evaluated for their diagnostic and prognostic significance.^386^ The findings suggest that liquid biopsy for the detection of STEAP1^+^ EVs could be a noninvasive diagnostic strategy for prostate cancer.

EV RNAs have also shown promise as prostate cancer biomarkers. Urinary exosome miR-375 showed a significant correlation with clinical T stage and bone metastasis in prostate cancer patients (*P* < 0.05). And ROC curves demonstrated that levels of urinary exosome miR-375, miR-451a, miR-486-3p, and miR-486-5p could differentiate prostate cancer patients from healthy controls. Notably, urinary exosome miR-375 excelled in distinguishing localized prostate cancer from metastatic cancer with an AUC of 0.806. And additionally, combining urinary exosome miR-375 with miR-451a distinguished from prostate cancer patients with benign prostatic hyperplasia.^386^ In addition to miRNAs, other EV RNAs may serve as diagnostic biomarkers for prostate cancer. These include sncRNAs and specifically, piRNAs, which were identified in urinary sEVs via RNA sequencing. In an initial discovery cohort of 10 men, including 5 with prostate cancer, promising biomarker targets were identified and subsequently validated by using RT-qPCR in a larger cohort comprising 40 patients, including 30 prostate cancer patients, with varying degrees of success.^387^

The pursuit of viable biomarkers such as PSA and CD81 for detecting and monitoring prostate cancer remains a subject of ongoing investigation, with EVs emerging as promising candidates. PSA contained in CD81^+^ exosomes scored an AUC of 0.98.^388^ Additionally, mRNAs, including ACP3, FOLH1, HOXB13, KLK2, KLK3, KLK4, MSMB, RLN1, SLC45A3, STEAP2, and TMPRSS2, have been identified as key markers. These biomarkers can noninvasively identify metastatic prostate cancer and monitor dynamic disease states, complementing imaging tools and blood-based tests for the timely detection of metastatic progression, ultimately enhancing patient care.^389^

#### 3.1.6 Liver Cancer

Liver cancer, also referred to as hepatocellular carcinoma (HCC), ranks as the sixth most common cancer worldwide and stands as the third leading cause of cancer-related mortality. Regrettably, over 90% of patients receive their diagnosis at a late stage, leading to a poor prognosis, with survival rates ranging from 40 to 70%, necessitating a need for early detection.^344^ However, the detection of HCC in its nascent stages is challenging.

However, EVs derived from HCC cells have emerged as sources of promising biomarkers for HCC detection. The identification of specific biomarkers within EVs not only signals the presence of HCC but also provides crucial insights into disease progression.^390^ SiEV, numerous EV surface proteins, including TENM2, ITGA1, CD36, DSC1, TIMP2, and MUC1, showed good diagnostic performance for liver cancer, with the highest AUC value being 0.988.^391^ In addition, CD63, GPC3, EpCAM, CD113, and CD147 have been analyzed as potential protein biomarkers in EVs for HCC detection. Furthermore, combining these specific biomarkers with CD63 has exhibited a strong correlation with early HCC diagnosis, boasting an AUC of 0.95 (95% CI, 0.90–0.99), a sensitivity of 91%, and a specificity of 90% in clinical samples.^392^ Mutated genes carried within EVs could also be biomarkers for HCC, with candidates being mutated versions of *TERT* and *CTNNB1* (β-catenin), the most commonly altered genes in HCC.^393^ In addition, EV-associated miRNAs and lncRNAs have been extensively explored as potential early biomarkers for HCC diagnosis and prognosis.^394^ These include EV miRNAs miR-16-5p, miR-19-3p, miR-30d-5p, miR-223-3p, and miR-451a, and EV lncRNAs SFTA1P, HOTTIP, HAGLROS, LINC01419, HAGLR, and CRNDE. EV-derived LINC00853 is another potential diagnostic biomarker for early HCC, especially for AFP-negative HCC.^395^

#### 3.1.7 Other Types of Cancer

EVs are currently under investigation for their clinical utility as biomarkers for the detection of other types of cancer, including cancer of the stomach (also known as gastric cancer), bladder, ovaries, and skin.

EVs derived from stomach cancer cells encapsulate proteins such as claudin-7, CD44v6, CD44, c-MET, EGFR, EpCAM, and GPC1, all of which are closely associated with the progression of gastric cancer. And EV DNA isolated from gastric cancer patients contains mutations in genes such as *TP53*, *KRAS*, and *HER2* (*ERBB2*). These genetic alterations represent additional avenues for detecting and monitoring gastric cancer through EV-based analysis, potentially enhancing the ability to diagnose and manage this disease. In addition, miRNAs such as miR-21-5p, miR-26a-5p, and miR-27a-3p are significantly elevated in EVs derived from the serum of gastric cancer patients, and lncRNAs like CCAT1 and HOTAIR have displayed dysregulation in EVs derived from these patients.^396,397^ These molecular signatures offer valuable insights into both the diagnosis and prognosis of gastric cancer.

EVs derived from bladder cancer cells are packed with specific biomarkers crucial for diagnosis and prognosis. These biomarkers offer targeted insights that can advance clinical approaches to this type of cancer. EV protein biomarkers have been identified for bladder cancer, especially MUC-1, CCDC25, and GLUT1. These biomarkers effectively distinguished bladder cancer patients with high clinical sensitivity and specificity, with an AUC of 0.98, advancing the discovery and clinical application of EV-based biomarkers.^188^

EVs derived from ovarian cancer cells harbor specific biomarkers critical for the diagnosis and monitoring of ovarian cancer.^398^ One of these biomarkers is human epididymis protein 4 (HE4), a protein elevated in the blood of patients with ovarian cancer.^399^ HE4 and other EV proteins, namely EpCAM, CD24, VCAN, and TNC, can distinguish high-grade serous ovarian cancer (HGSOC) from noncancer cases with 89% sensitivity and 93% specificity, effectively classifying patients into noncancer, early-stage HGSOC, and late-stage HGSOC groups.^400^ EV-encapsulated miRNAs may also be used as biomarkers for ovarian cancer. Possible EV miRNAs include miR-141-3p and miR-200c-3p.^401^ Trinidad and colleagues were the first to identify and employ lineage-specific exosome protein biomarkers focused on the early detection of HGSOCs.^398^ Since most HGSOCs typically arise from the fallopian tubes, the researchers’ EV-related biomarker search focused on proteins found on the surface of EVs released by both fallopian tube (FT) and HGSOC tissue explants and representative cell lines. Using these lineage-specific exosome protein biomarkers, they could achieve a PPV of 15.3% with a sensitivity of 0.90 at a specificity of 99.8%, far exceeding the diagnostic value of CA125. Importantly, these exosome protein biomarkers can accurately discriminate between ovarian cancer and 12 types of cancer commonly diagnosed in women.^402^ In fact, the FDA granted breakthrough designation in 2024 for an EV-diagnostic assay based on these biomarkers for ovarian cancer screening. These EV-derived biomarkers offer a comprehensive and promising approach for diagnosing and monitoring ovarian cancer as well as developing personalized treatment strategies for the disease, highlighting their importance in clinical applications.

Ewing sarcoma (EWS) is a rare cancer that starts in the bones of kids and teenagers and is characterized by chromosomal translocation between the *EWS* gene (chromosome 22) and members of the ETS family of transcription factors (such as FLI1, ERG, ETV1, E1AF, etc.) resulting in fusion oncoprotein/transcription factor that drive tumor development.^403^ The clinical presentation of EWS is quite often nonspecific, with the most common symptoms at presentation consisting of pain, swelling, or general discomfort. Despite the majority of patients presenting with localized disease, approximately 30% succumb to relapse and die despite salvage therapies. Therefore, the discovery of novel EWS biomarkers for diagnosis and monitoring disease progression and recurrence is imperative in the management of this disease. Samuel and colleagues performed the first high-quality proteomic study of EWS-derived EVs, identifying 2 membrane-bound proteins with biomarker potential, CD99/MIC2 and NGFR. CD99/NGFR.^404^ Using an immuno-enrichment strategy, they successfully isolated CD99/NGFR-positive sEVs, which carried EWS-ETS fusion transcripts. This approach demonstrated strong diagnostic performance, with an AUC of 0.92 (*P* = 0.001) for sEV numeration, a positive predictive value of 1 (95% CI, 0.63–1), and a negative predictive value of 0.67 (95% CI, 0.30–0.93). Subsequently, the researchers quantitatively measured *EWS-FLI1* mRNA copy numbers in EWS-derived EVs, further enhancing diagnostic capabilities.^405^ Building on these findings, Turaga and colleagues expanded these EWS proteomic studies and defined a panel of exosome protein biomarkers, i.e., CD99, NGFR, EZR, ENO2, UGT3A2, and SLC52A1, that could accurately diagnose EWS (AUC of 0.98 when combining UGT3A2 with Ezrin).^406^ Finally, Crow and colleagues developed an EV-based miRNA signature that could also accurately diagnose EWS patients via a liquid biopsy with high sensitivity and specificity, and clinical validation of an assay to measure minimal residual disease.^407^

EVs can also be a source of biomarkers for melanoma, a type of skin cancer that starts in melanocytes. Despite its high mortality rate, melanoma is highly curable if diagnosed and treated early.^408^ EV biomarkers for melanoma include proteins such as MCSP, MCAM, LNGFR, and ErbB3, which show great potential for improving early diagnosis and prognosis.^409^ In addition, EV miRNA profiling has identified miR-1180–3p as a promising diagnostic marker for melanoma. In a study involving plasma samples, miR-1180-3p expression was significantly decreased in melanoma patients. The diagnostic potential of miR-1180–3p was further validated in a cohort of melanoma patients (*n* = 28) and healthy controls (*n* = 28), confirming its effectiveness as a novel biomarker. This discovery provides new insights into melanoma development and highlights miR-1180–3p as a potential early biomarker for skin cancer diagnosis.^410^

### 3.2 EV Biomarkers in Chronic Diseases

#### 3.2.1 Cardiovascular Diseases

Cardiovascular diseases are major causes of morbidity and mortality globally, and the complex interaction between diabetes and cardiovascular health poses significant challenges in clinical management. EVs have emerged as valuable biomarkers for diagnosing cardiovascular diseases as well as elucidating the pathophysiology of these diseases and their diabetes-related complications.^411^

One cardiovascular disease that EVs are helping to diagnose is coronary artery disease, a chronic inflammatory condition that often remains asymptomatic until it leads to severe outcomes such as angina, myocardial infarction, or death. Urinary sEVs are emerging as valuable biomarkers for this disease. One study found that proteins such as AZGP1, SEMG1/2, ORM1, IGL, SERPINA5, HSPG2, prosaposin, gelsolin, and CD59 were upregulated in patients with coronary artery disease, while UMOD, KNG1, AMBP, prothrombin, and TF were downregulated. Notably, compared to healthy controls, patients with stable coronary artery disease had lower levels of the protein UMOD, and for patients who recently suffered a myocardial infarction, the levels were even lower, suggesting that UMOD could serve as an early diagnostic biomarker for coronary artery disease.^412^

EVs are also helping to diagnose heart failure, a leading cause of death exacerbated by aging that necessitates early detection for effective risk reduction. EV miRNAs, namely miR-30a-5p and miR-654-5p, are key biomarkers for this condition. In plasma samples from heart failure patients, miR-30a-5p was upregulated while miR-654-5p was downregulated, compared to levels in healthy controls. A diagnostic model based on these miRNAs demonstrated 98.9% sensitivity and 95% specificity in an independent cohort of 50 patients with heart failure and 30 controls, surpassing NT-pro BNP in accuracy. miR-30a-5p and miR-654-5p, as novel biomarkers, and present a promising 2-miRNA model for heart failure diagnosis and prognosis and for monitoring treatment responses.^413^

In addition to providing biomarkers, EVs can also help elucidate the pathophysiology of cardiovascular diseases and conditions. For example, in aortic aneurysms, the expression levels of circulating EV miRNAs, specifically miR-34a, miR-133a, and miR-320a, vary according to the aortic valve morphotype. This variation in miRNA expression provides valuable insights into the relationship between aortic valve morphology and aneurysm development.^414^

Stroke is the fourth leading cause of death, with ∼800,000 people experiencing a new or recurrent stroke each year in the United States.^415^ Worldwide, stroke is responsible for ∼12% of fatalities, which makes it the second leading global cause of death, after heart disease. Between the 2 types of stroke, hemorrhagic stroke (bleeding into the brain) and acute ischemic stroke (blockage of a blood vessel), acute ischemic stroke is much more common (85% of patients), and rapid diagnosis is essential for treatment. Analysis of mRNA revealed that the expression of acute ischemic stroke-specific genes in CD8^+^ EVs was correlated with the expression in their parental T cells, in both cell lines and healthy donors. In a blinded study, 80% test positivity for acute ischemic stroke patients and controls was revealed.^416^ Also, EV miRNAs following stroke were assessed for diagnosing stroke. The identified miRNA signatures demonstrated a high degree of accuracy in the diagnosis of acute ischemic stroke with an AUC of 0.83–0.93.^206^

EVs have also helped elucidate the mechanism underlying diabetic retinopathy, a prevalent and severe complication of diabetes that significantly impacts vision in working-age adults. Although the mechanisms driving diabetic retinopathy remain unclear, emerging research underscores the vital role of circRNAs in its development, particularly through the competing endogenous RNA model, which regulates gene expression. Deep sequencing of EV RNAs from the serum of patients at different stages of diabetes has identified circMKLN1 as a key player in autophagy regulation. This circRNA acts as a molecular sponge for miR-26a-5p, modulating Rab11a-mediated autophagy and thereby affecting the chronic inflammation and microvascular dysfunction associated with diabetic retinopathy. CircMKLN1’s interaction with miR-26a-5p in regulating Rab11a highlights the potential of this circRNA as a new biomarker for this diabetes complication.^417^

EVs have also shed light on cardiac fibrosis, a critical feature of late-stage familial dilated cardiomyopathy that has been challenging to elucidate. In a recent study, injecting EVs secreted from familial dilated cardiomyocytes into mouse hearts significantly exacerbated cardiac fibrosis and impaired cardiac function, suggesting that these EVs contribute to fibrogenesis. The EVs carried upregulated miR-218-5p, which promotes fibrogenesis by activating the TGF-β signaling pathway and inhibiting TNFAIP3, a crucial inflammation suppressor. These results underscore the profibrotic role of cardiomyocyte-derived EVs and identify miR-218-5p as a key factor, providing new insights and potential therapeutic targets for mitigating cardiac fibrosis in dilated cardiomyopathy.^418^

As these and other studies indicate, EVs have emerged as significant players in the study of cardiovascular diseases. While their roles in prevalent conditions such as atherosclerosis and myocardial infarction have been well studied, their roles and diagnostic potential for rarer cardiovascular diseases remain largely unexplored.

SiEV analysis is pivotal for understanding cardiovascular diseases by providing detailed insights into the role of EVs in disease mechanisms, such as Lp­(a)-mediated calcification. It allows for the identification of changes in EV subpopulations, such as the conversion of exosomes into microvesicles under Lp­(a) stimulation. This precision in analyzing EVs enhances diagnostic accuracy, supports the development of targeted therapies for vesicular cardiovascular calcification, and enables effective monitoring of disease progression and therapeutic responses.^419^ Recent studies have revealed that annexin A1 (ANXA1) is predominantly associated with microvesicles that aggregate and contribute to calcification. Notably, ANXA1-enriched EVs serve as biomarkers of microcalcifications, particularly in vulnerable plaque regions. Furthermore, ANXA1-neutralizing antibodies effectively prevented vesicle aggregation, highlighting its critical role in microvesicle aggregation and calcification. These findings suggest that ANXA1-mediated EV processes may be relevant not only to vascular calcifications but also to other diseases involving EV biomarkers, such as autoimmune disorders, neurodegenerative conditions, and cancer.^420^

#### 3.2.2 Neurodegenerative Diseases

Because of their progressive nature and limited treatment options, neurodegenerative diseases, including Alzheimer's disease and Parkinson's disease, represent major challenges to global health. One key challenge in managing these diseases is the lack of effective single-analyte biomarker tests to accurately track disease progression. EVs isolated from patients with these diseases carry relevant biomolecules, which may prove to be crucial biomarkers for early diagnostics and treatment monitoring.

The role of EVs in Alzheimer's disease has received considerable attention, with research indicating that EVs contain several proteins that are relevant for the disease and may make valuable biomarkers. EVs derived from neurons and glial cells can carry pathological forms of tau protein, which aggregates in Alzheimer's disease as well as in other tauopathies, and elevated levels of tau-containing EVs in cerebrospinal fluid and blood are associated with disease severity and progression. Astrocyte-specific EVs from brains of patients with Alzheimer's disease show significantly elevated integrin-β1, which is strongly associated with Alzheimer's disease pathology and cognitive impairment and correlates with brain β-amyloid and tau loads.^129^ Additionally, EVs derived from glial cells carry neuroinflammatory proteins, such as interleukin-1β, TNF-α, CCL2, CXCL10, and enzymes that generate reactive oxygen species. And since neuroinflammation plays a crucial role in Alzheimer's disease, with the activation of microglia and astrocytes and the release of pro-inflammatory cytokines and chemokines contributing to neuronal damage and disease progression, these proteins may be helpful biomarkers for the disease.^421^ EVs can also carry other proteins central to the pathogenesis of Alzheimer's disease, including cathepsin B, pS396 tau, Aβ1–42, ANXA5, VGF, GPM6A, and ACTZ.^422^ PLAU, ITGAX, and ANXA1 proteins have also been identified as key biomarkers for disease diagnosis. Along with proteins, EVs carry miRNAs that are relevant for Alzheimer's disease. EV miRNAs reveal a network module that plays a critical role in neural function and is significantly linked to Alzheimer's disease diagnosis and cognitive impairment.^423^ Central miRNAs within this module, including hsa-miR-9-3p, hsa-miR-9-5p, and hsa-miR-3383p, are involved in key pathways related to Alzheimer's disease neuropathology, such as the modulation of beta-secretase 1 and growth hormone signaling. The reduced expression of these miRNAs in patients with Alzheimer's disease and patients with mild cognitive impairment highlights their potential as early biomarkers for cognitive decline and Alzheimer's disease progression.

Analysis of surface proteins on EVs has revealed distinct patterns associated with Alzheimer's disease, with urinary EVs showing particularly strong correlations. These findings highlight the diagnostic potential of EV subpopulations, which demonstrated high accuracy in distinguishing AD samples. Notably, a urinary EV subpopulation enriched with the signature proteins PLAU, ITGAX, and ANXA1 achieved an 88% diagnostic accuracy for Alzheimer's disease. These protein findings underscore the promise of EV biomarkers, particularly those in bodily fluids like urine, for the noninvasive detection of Alzheimer's disease.^424^ In addition, ATPase Na^+^/K^+^ transporting subunit alpha 3 (ATP1A3) is abundantly expressed in EVs isolated from induced human neurons, brain tissue, cerebrospinal fluid, and plasma.^114,167^ This expression is significantly higher than the expression of the presumed neuron-derived EV markers NCAM1 and L1CAM. Proteomic analysis of immunoprecipitated ATP1A3^+^ brain-derived EVs reveals a greater enrichment of synaptic markers and Alzheimer-disease-related cargo proteins than that of NCAM1^+^ or L1CAM^+^ EVs. Furthermore, single particle analysis of plasma from patients with Alzheimer's disease indicates higher amyloid-β positivity in ATP1A3^+^ EVs, suggesting they provide better diagnostic prediction than that of other plasma biomarkers.^114^

The transmembrane protein L1CAM has emerged as a promising EV biomarker in human serum for Parkinson's disease, highlighting its clinical potential. However, the low abundance of neuronal-derived EVs in circulation and the high levels of soluble L1CAM (sL1CAM) in plasma present challenges for analysis.^425^ Beyond Parkinson's disease, L1CAM-expressing neuronal-derived EVs have been implicated in long COVID, where they contain proteins linked to Alzheimer's disease. Notably, 14 proteins elevated in long COVID were also associated with AD, with six shared proteins (MIF, ENO1, MESD, NUDT5, TNFSF14, and FYB1) specifically linked to cognitive impairment. While no common proteins were found between HIV and AD, one shared protein (BST1) was identified between HIV and long COVID. These findings underscore the diagnostic potential of EV biomarkers in neurodegenerative and postviral syndromes.^426^

EVs are also being investigated as a source of biomarkers for Parkinson's disease, with EV-derived proteins and miRNAs showing promise for diagnosis and treatment monitoring. Various liquid biopsies have detected EVs derived from neurons and glial cells that carry α-synuclein aggregates, characteristic of Parkinson's disease and a possible biomarker for disease diagnosis and progression.^427^ Circulating brain-enriched miRNAs can distinguish between idiopathic and genetic forms of Parkinson's disease. EV-derived miRNAs can also contribute to a minimally invasive test for the early detection and monitoring of Parkinson's disease and REM sleep behavior disorder, crucial for drug development and patient care planning. Research has identified several upregulated miRNAs (including miR-27b-3p, miR-151a-3p, and miR-199a-5p) and downregulated miRNAs (such as miR-96-5p and miR-155-5p) in patients with Parkinson's disease. A diagnostic model based on these miRNAs achieved 97.1% sensitivity, 87.5% specificity, and 92.5% accuracy in the training set, and 92% sensitivity, 85.7% specificity, and 89.1% accuracy in the validation set. Notably, miR-27b-3p was upregulated in the group with Parkinson's disease and the sleep disorder, while miR-182-5p and miR-7-5p were downregulated. The diagnostic performance of the training set showed 97.1% sensitivity, 88.2% specificity, and 92.8% accuracy, with the validation set confirming 96% sensitivity, 86.4% specificity, and 91.5% accuracy.^428^

EVs can also serve as a source of biomarkers for other neurodegenerative diseases. EVs carry measurable levels of TAR DNA-binding protein 43 (TDP-43) and tau, including 3-repeat (3R) and 4-repeat (4R) tau isoforms. EV TDP-43 levels are elevated in amyotrophic lateral sclerosis and in frontotemporal dementia.^429^ Both biomarkers show high diagnostic accuracy, with an AUC of 0.9, and are strongly correlated with neurodegeneration and clinical severity markers. Thus, the combination of EV TDP-43 levels and EV 3R to 4R tau ratios presents a promising approach for molecularly diagnosing amyotrophic lateral sclerosis, frontotemporal dementia, and frontotemporal dementia spectrum disorders. EVs can also carry CD44 and CD133, biomarkers of glioblastoma, a fatal brain tumor characterized by its aggressive nature and poor prognosis. Glioblastoma cell–derived exosomes carrying these markers were isolated from both the blood and cerebrospinal fluid of a mouse model, possibly representing a minimally invasive approach for diagnosing glioblastoma, reducing the need for surgical biopsies and enabling easier and more frequent monitoring of the disease.^430^ CD44 and CD133 were sensitively detected in immunocaptured glioblastoma cell-derived exosomes, highlighting their potential as diagnostic markers. Similarly, EVs derived from cells affected by Huntington disease contain specific proteins associated with the disease’s pathology, underscoring their role in disease monitoring and progression analysis.^225^ Huntington's disease stems from a mutation in the *HTT* gene marked by CAG repeat expansion. EVs can carry mutated forms of the *HTT* gene and genetic material from other genes involved in the pathogenesis of Huntington disease.^197,431^

#### 3.2.3 Chronic Kidney Disease

Early detection and management of chronic kidney disease are vital to mitigate its impact and improve patient outcomes. EVs have emerged as a pivotal factor in understanding the pathogenesis and progression of chronic kidney disease, and research into EVs as biomarkers for the disease is rapidly advancing, as they offer several diagnostic advantages. Unlike traditional biomarkers, such as urinary protein or microprotein levels, EVs do not require specific collection times, thereby enhancing their diagnostic utility. And, particularly when derived from plasma, EVs are stable in circulation, facilitating longitudinal monitoring of disease progression and treatment response.

Several promising EV-derived biomolecules have been identified. For instance, miRNAs such as miR-21, miR-142-3p, and miR-221 are associated with high fibrosis scores in renal histology, highlighting their potential as indicators of renal damage. Among these, miR-21 derived from plasma EVs (but not whole plasma) also correlates with high-grade interstitial fibrosis and tubular atrophy, potentially offering an alternative to renal biopsy for more frequent and earlier monitoring. Recently, novel biomarkers have been identified by kidney tissue microarrays of EV-derived mRNAs. By using this approach, AEBP1 levels in plasma EVs have been confirmed as a promising biomarker for chronic kidney disease with strong diagnostic efficacy.^432^ Moreover, positive for CD9 and α8 integrin, originating from renal mesangial and glomerular endothelial cells, have shown significant potential for diagnosing chronic kidney disease when detected in plasma. This was validated in clinical samples from kidney transplant recipients and healthy controls. Further insights have emerged from studies on urine-derived EVs in autosomal dominant polycystic kidney disease.^433^ Several miRNAs, including miR-146a-5p, miR-199b-3p, miR-320b, miR-320c, miR-671-5p, miR-1246, miR-8485, and miR-3656, as well as piRNAs, like has-piR-016271, has-piR-020496, and has-piR-020497, were found to be significantly upregulated in the urine EVs of these patients, while miR-29c was downregulated. And target genes, like *FBRS*, *EDC3*, *FMNL3*, *CTNNBIP1*, and *KMT2A*, point to new potential biomarkers and drug targets aimed at slowing disease progression.^434^ EV-derived biomarkers offer valuable insights into the early detection of kidney injury, disease progression, and monitoring of treatment responses. Proteins and nucleic acids transferred by EVs hold considerable promise as novel biomarkers for chronic kidney disease, providing a noninvasive means to enhance diagnosis and tailor therapeutic strategies.

#### 3.2.4 Chronic Liver Diseases

EVs are also a promising source of biomarkers for diagnosing chronic liver diseases, including metabolic dysfunction–associated steatotic liver disease (MASLD), previously referred to as nonalcoholic fatty liver disease (NAFLD).^435^ The miRNA miR-135a-3p in circulating serum EVs shows potential as a noninvasive marker for NAFLD, reflecting disease presence and progression. Hepatocyte-derived EV miR-192–5p, which modulates the Rictor/Akt/FoxO1 signaling pathway, also plays a critical role in disease progression and can serve as a biomarker for monitoring the disease. Hepatocyte-specific EVs carrying asialo-glycoprotein receptor 2 (ASGR2) and cytochrome P450 family 2 subfamily E member 1 (CYP2E1) have been associated with NASH detection. These proteins are present at higher levels in the early stages of the condition and decrease as the disease resolves, particularly after interventions such as weight loss surgery.^435^

Alcoholic liver disease spans a range of liver conditions, including liver steatosis, alcoholic hepatitis, fibrosis, and cirrhosis, and miRNAs have emerged as potential biomarkers for these conditions. In the serum of individuals using alcohol and patients with alcoholic liver disease, miRNA-122 and miRNA-155 are upregulated, and miRNA-146a is downregulated, compared to levels in individuals who do not use alcohol or in healthy controls. Despite miRNA-122 upregulation distinguishing patients with alcoholic hepatitis from healthy controls, overall, the diagnostic accuracy of miRNAs for distinguishing between different stages of alcoholic liver disease–related fibrosis and HCC remains inconclusive and requires further investigation. Among the miRNAs studied, miRNA-122 stands out as the most promising biomarker for managing alcoholic liver disease, though more research is needed to refine its diagnostic accuracy.^436^ In another study, analyzing plasma samples before and after the antifibrotic treatment PRI-724 revealed 3 miRNAs, miR-4261, miR-6510-5p, and miR-6772-5p, that predicted the treatment response, while 3 other miRNAs, miR-887-3p, miR-939-3p, and miR-7112-5p correlated with treatment efficacy. Notably, significantly decreased in liver tissue following PRI-724 administration, miR-887-3p was detected in hepatocytes, and its levels in blood may indicate recovery from liver fibrosis. Moreover, miR-887-3p mimics transfection in activated hepatic stellate cells, suggesting that reduced miR-887–3p levels could reflect improvement in liver fibrosis.^437^ Further studies are needed to validate these findings. Finally, studying serum EVs has identified fibulin-3 as a new predictor of liver-related events in metabolic-associated steatotic liver disease.^438^ Overall, EVs offer a dynamic and noninvasive approach to tracking liver disease progression and response to treatment.

#### 3.2.5 Chronic Respiratory Diseases

EVs are increasingly recognized as promising biomarkers for chronic respiratory diseases, encompassing conditions like chronic obstructive pulmonary disease (COPD), asthma, interstitial lung diseases, and pulmonary fibrosis. Airway epithelial cells, pivotal in the pathogenesis of these diseases, release EVs containing miRNAs and proteins indicative of airway inflammation, injury, and repair. Analyzing these markers in EVs isolated from sputum or bronchoalveolar lavage fluid may offer mechanistic insights and aid in diagnosis and monitoring.^439^

Studies have shown that EV miRNAs are involved in both COPD and asthma, offering new avenues for diagnostic and therapeutic strategies. In COPD, the EV miRNA miR-210 has been implicated in regulating autophagic functions and myofibril differentiation in the lungs, suggesting its potential as a diagnostic biomarker. Similarly, miR-21 is also considered a potential biomarker for COPD. In asthma, bronchial stress is associated with the inhibition of EV miR-145, while plasma EV miR-155 is found to be highly expressed in asthma patients. Other miRNAs, such as miR-16, miR-125b, miR-126, miR-133b, miR-206, and miR-299-5p, have been linked to asthmatic responses and hold promise as plasma EV biomarkers for asthma.^440^

Inflammatory cells like neutrophils, macrophages, and lymphocytes play pivotal roles in chronic respiratory disease, releasing pro-inflammatory mediators and reactive oxygen species.^439^ EVs released by these cells carry markers indicative of immune activation and inflammation, including cell surface markers (e.g., CD63, CD81), cytokines (e.g., TNF-α, IL-6), and chemokines (e.g., CXCL8, CCL2), and evaluating this inflammatory cell–derived EV cargo could help assess airway inflammation, predict disease exacerbation, and monitor treatment response.^440,441^ EV-derived miRNAs could also help, as specific miRNAs detected in EVs from blood or respiratory secretions, such as miR-21, miR-146a, and miR-155, are associated with airway inflammation, oxidative stress, and tissue remodeling in chronic respiratory diseases.^442^

### 3.3 EV Biomarkers in Infectious Diseases

By reflecting their cellular origins, EVs can serve as a promising source of biomarkers for various infectious diseases. EVs released by infected cells can encapsulate viral RNA and proteins, making EVs instrumental in detecting viral infections and monitoring disease progression. Similarly, EVs can help differentiate between types of bacterial infections and assess disease severity. Studying EVs can identify specific biomarkers linked to infections, offer insights into mechanisms of infection and related complications, and even help develop vaccines and treatment strategies. EV biomarkers hold significant promise for advancing the clinical diagnosis of infectious diseases.^443^ Their ability to provide noninvasive, sensitive, and specific diagnostic information can lead to earlier detection, better disease monitoring, and improved patient outcomes.^443–445^

#### 3.3.1 HIV and Hepatitis

EVs can be a source of biomarkers for infection with HIV or hepatitis B or C. In patients with these infections, EVs containing viral RNA sequences aid in diagnosing and monitoring infection progression.^300^ EVs can also aid in detecting coinfection with HIV and hepatitis C, as this influences the miRNA cargo of plasma-derived EVs, resulting in a specific miRNA signature linked to inflammation and cancer-related pathways.^446^

Research has also shown that EVs play a role in HIV infection and its complications, information that can lead to the discovery of additional biomarkers or therapeutic interventions. SiEV analysis using direct stochastic optical reconstruction microscopy demonstrated that HIV-1 particles released EVs from chronically infected T cells and that CD63, HIV-1 integrase, and the viral envelope glycoprotein Env colocalized on the same Frac-E particles.^447^ Notably, these Frac-E particles were infectious, and their infectivity significantly decreased when Frac-E was immunodepleted with anti-CD63, indicating the presence of this protein on the surface of the small infectious particles. This study is the first to identify infectious small HIV-1 particles under 50 nm using EV isolation methods. These findings suggest that the interactions between EVs and HIV-1 might be more complex than previously thought, with potential implications for viral pathogenesis. EVs can also play a role in complications of HIV-1 infection, namely HIV-associated neurocognitive disorder, characterized by neurological impairment and persistent inflammation.^301^ During infection, cells release viral products like TAR RNA via EVs, impacting neighboring cells and contributing to disease progression. EVs from HIV-1–infected myeloid cells may cause central nervous system damage through toll-like receptor 3 activation. EVs carrying viral components may also drive chronic central nervous system inflammation, especially in patients on combination antiretroviral therapy, highlighting the need for targeted therapies to alleviate such a complication.

#### 3.3.2 COVID-19

EVs can also be a source of biomarkers for SARS-CoV-2, the virus causing COVID-19. EVs from SARS-CoV-2–infected cells contain viral RNA and proteins, such as the nucleocapsid protein, making them valuable for diagnosing COVID-19 and monitoring viral load. These EVs also carry pro-inflammatory cytokines like IL-6 and TNF-α, markers of the inflammatory response associated with severe disease and cytokine storms.^448,449^

EVs can also provide insight into SARS-CoV-2 pathogenesis and the immune response, possibly providing additional biomarkers or targets for therapeutic intervention. For example, one study showed that the exosome protein TMPRSS2 facilitates viral entry into host cells by cleaving the spike protein via the ACE2 receptor.^450^ And another study revealed that a specific subset of EVs is involved in severe COVID-19. Proteomic profiling of SiEVs showed that SARS-CoV-2 colocalizes with a CD81/integrin-rich subpopulation of EVs in the sputum of patients with severe COVID-19. Using a proximity barcoding assay, researchers examined immune-related proteins in SiEVs and demonstrated that CD81-regulated EV subpopulations play a significant role in pneumonia and the immune response to SARS-CoV-2 infection. These findings indicate that EVs from sputum samples carry both host- and virus-derived proteins, which are altered by infection. This research highlights the involvement of EVs in viral infection and immune responses, offering valuable insights into SARS-CoV-2 pathogenesis and suggesting the potential for developing nanoparticle-based antiviral therapies.^81^

EVs have even been used to prevent or treat COVID-19. The STX-S vaccine contains EVs that deliver the SARS-CoV-2 spike protein, producing a robust humoral immune response with high levels of neutralizing antibodies targeting the Delta and Omicron variants (BA.1 and BA.5), offering broader protection than the current mRNA vaccines.^451^ Additionally, this vaccine significantly enhances CD4^+^ and CD8^+^ T-cell responses. Another innovative approach to combat COVID-19 involves heparin-conjugated ACE2-bearing EVs, which effectively neutralize the Omicron variant by interacting with the viral spike protein. In vitro studies have shown that these EVs bind to the SARS-CoV-2 pseudovirus, preventing its infection of host cells, while in vivo experiments demonstrated that an inhalable version of these EVs can safely block pseudovirus infection in lung tissue. This dual-decoy strategy holds promise for both preventive and therapeutic applications.^452^

#### 3.3.3 Human Adenovirus

Human adenovirus infection can lead to severe pneumonia in children, with high morbidity and mortality rates, and currently, specific diagnostic biomarkers for human adenovirus–associated pneumonia are lacking. However, miRNA sequencing of serum exosomes has identified miRNAs that can differentiate adenovirus-infected patients from healthy controls.^453^ These miRNAs, namely miR-98-5p, miR-103a-3p, miR-103b-5p, and miR-450a-5p, may improve the diagnosis of pneumonia in children with adenovirus infections.

#### 3.3.4 Tuberculosis

Finally, EVs can be a source of biomarkers for TB. EVs from the urine of TB patients contain a range of biomarkers, including lipoarabinomannan (LAM) and CFP-10 (Rv3874), which are present in both pulmonary and extrapulmonary TB. Detection of LAM in urinary EVs offers a promising adjunct test for the rapid diagnosis of TB, and an immuno-polymerase chain reaction assay targeting LAM in these EVs could further enhance diagnostic speed and accuracy.^454,455^ In addition to LAM and CFP-10, –encoded ASdes and miR5, unique to TB patients, show potential as sensitive and precise diagnostic tools. These biomarkers, found in plasma-derived EVs from individuals with active pulmonary TB, could provide a minimally invasive approach for TB screening and diagnosis.^203,456^ Moreover, using an automated nanoparticle-enhanced immunoassay combined with dark-field microscopy and machine learning enables convenient smartphone-based, point-of-care detection of virulence factors on circulating EV surfaces. Advances in SiEV analysis will further enhance our understanding of pathogen physiology and improve diagnostic and therapeutic strategies.^250,457^

CD9 or CD81 indicates a plasma membrane origin, and CD63 suggests an endosomal origin. Analysis of tetraspanin expressions on virus-like particles (VLPs) showed that HIV-Gag–induced VLPs resemble EVs more closely than SARS-CoV-2-NP/M/E–induced VLPs. HIV-Gag–green fluorescent protein (GFP) VLPs highly colocalized with CD9, CD63, and CD81, while SARS-CoV-2-NP-GFP VLPs did not. This suggests that tetraspanin-expressing EVs may be produced similarly to HIV.^81^

## 4 Analyzing EVs

EV-based diagnostics offer many promises, but several remaining challenges hinder their widespread adoption (Figure 7). In this section, we examine the promises, challenges, traditional methods, limitations, and biases associated with analyzing EVs for EV-based diagnostics.

**Figure 7:**
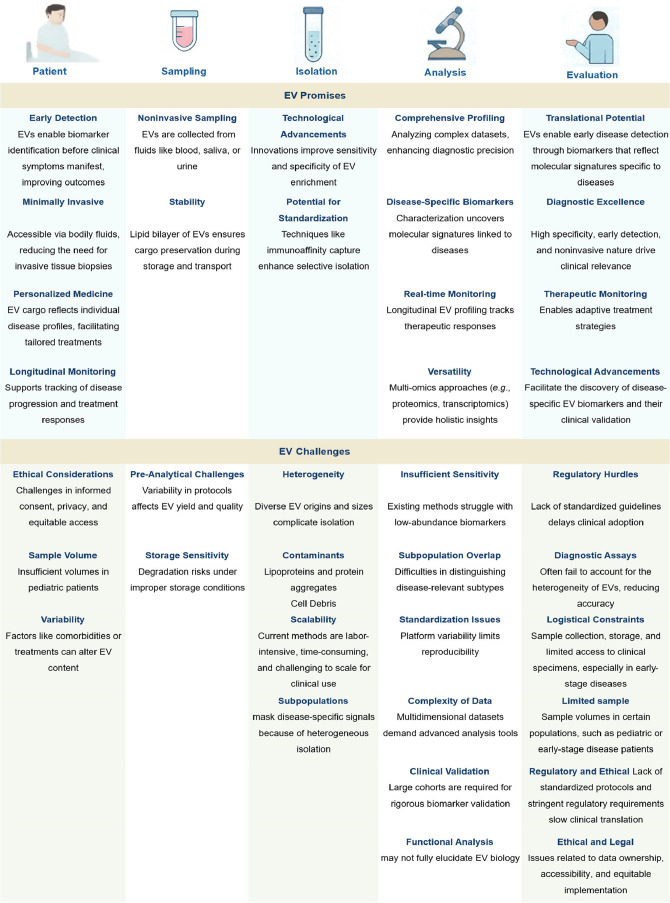
Overview of the promises and challenges of EV-based diagnostics across key stages: patient benefits, sample collection, isolation, characterization, and clinical applications. The upper section highlights advantages such as early detection, noninvasive sampling, technological advancements, comprehensive profiling, and translational potential. The lower section outlines challenges, including ethical considerations, preanalytical variability, heterogeneity, insufficient sensitivity, regulatory hurdles, and logistical constraints, which impact clinical applications. Figure created with Biorender.com.

### 4.1 Promise of Analyzing EVs

EVs possess remarkable potential, promising to improve medicine. One of the key advantages of EVs lies in their unparalleled capability for early disease detection.^325^ EVs can carry biomarkers for various diseases, allowing for early diagnosis and potentially improving patient outcomes. The specificity of EV biomarkers further enhances their utility, as they reflect the molecular signatures specific to different origins and diseases. By analyzing the molecular content of EVs, clinicians can differentiate between various disease states with precision, facilitating accurate diagnosis.^475^ Importantly, EV cargo composition often changes before clinical symptoms manifest, providing a critical window for timely interventions, when treatments are most effective. And EVs offer other advantages. The accessibility of EVs in bodily fluids such as blood, urine, and saliva allows for noninvasive or minimally invasive sample collection, thereby reducing patient discomfort and eliminating the need for invasive tissue biopsies.^476^ Furthermore, the lipid bilayer membrane surrounding EVs acts as a protective barrier, shielding their cargo from degradation by extracellular enzymes. This inherent stability ensures the integrity of biomarkers during sample storage and transportation, thereby enhancing the feasibility of EV-based diagnostics for clinical deployment.^183^

EVs also hold promise for personalized medicine approaches by elucidating individual disease profiles. EVs can provide detailed information about the disease state of a patient, enabling more personalized treatment plans. Through scrutiny of the unique molecular signatures present in EVs, clinicians can tailor treatment strategies to address each patient’s specific needs, thereby optimizing therapeutic outcomes. Additionally, longitudinal monitoring of EV composition provides valuable insights into disease progression and treatment response, empowering clinicians to make informed decisions and promptly adjust therapeutic strategies.^32^

Technological advancements, including refinements in EV isolation techniques and analytical methods, have significantly enhanced the sensitivity and specificity of EV-based diagnostics.^183^ Techniques such as mass spectrometry, flow cytometry, and next-generation sequencing enable comprehensive profiling of EV cargo, facilitating the discovery and validation of disease-specific biomarkers. Furthermore, the integration of machine learning algorithms aids in the analysis of complex data sets generated from EV-profiling experiments, thereby augmenting diagnostic accuracy and efficiency.^477^ While much of the research on EV-based diagnostics remains in the preclinical realm, there is a growing interest in translating these findings into clinical practice. Ongoing clinical trials are rigorously evaluating the performance of EV-based diagnostic assays in diverse patient cohorts, with the aim of validating their clinical utility and establishing standardized protocols for routine adoption.^478^

EVs represent a versatile platform for disease detection, monitoring, and personalized management, highlighting the ongoing need for research and technological advancements to fully exploit their potential and enhance their impact in clinical applications. The potential of EV analysis in clinical diagnostics is expansive and continuously evolving, spanning from BuEV to SiEV analysis to understand pathological diversity. Key benefits include enhanced early disease detection, sensitivity and specificity, minimally invasive sampling, personalized medicine approaches, real-time monitoring, comprehensive profiling, and potential clinical translation.^479^ These factors underscore the role of EV-based diagnostics in improving patient care and advancing precision medicine. Continuous innovation and research efforts in this domain are crucial to harnessing the complete capabilities of EV analysis and ushering in a new era of diagnostic excellence.

### 4.2 Challenges of Analyzing EVs

Analyzing EVs offers remarkable potential, yet also presents challenges. EV populations are highly heterogeneous, derived from various cell types and diverse in composition. This heterogeneity complicates isolation and characterization protocols, making data interpretation and standardization of analysis methods difficult.^480^ Specifically, EVs exhibit significant size overlap with non-EV particles (NEVs), including supermers, exomeres, protein aggregates, lipoproteins (e.g., high-density lipoproteins [HDL] and low-density lipoproteins [LDL]), apoptotic bodies, and cellular debris.^16,35^ This overlap complicates the accurate categorization of EV subpopulations, potentially leading to misclassification and inconsistencies across studies. Additionally, the presence of NEVs, along with contaminants and sample impurities, can obscure experimental outcomes and hinder reliable data interpretation. This challenge highlights the importance of developing more precise methods for distinguishing between EVs and other particles to ensure the reliability of experimental findings.^34^

Mass spectrometry analysis has shown that plasma-derived very low–density lipoprotein (VLDL) and low-density lipoprotein (LDL) particles contain proteins such as LDL-receptor, CD14, protein S100-A8, and HLA class I molecules, which are also found in EVs.^481^ Contaminants from isolation procedures or coisolated particles may skew data analysis, complicating accurate assessment of the biological properties of EVs. DNA, RNA, and proteins are often associated with the surface of EVs, either naturally or as contaminants. Treatments with DNase, RNase, and proteinase are recommended to address these potential contaminants.^482^

EVs released by diseased cells, such as cancer cells, carry specific molecular signatures that can serve as biomarkers. For example, EVs from cancer cells may contain oncogenic proteins like p53 or mutated versions of genes such as *KRAS*, *BRAF*, *BRCA1*, or *EGFR*. These molecular signatures are crucial for cancer diagnosis.^483^ But if the heterogeneity of EVs is not accounted for, diagnostic assays might miss these disease-specific EVs, leading to less accurate diagnoses.^482,484^ EVs from different cellular origins can have varying biological activities. For example, EVs from immune cells might carry different signaling molecules compared to those from tumor cells. Understanding these functional differences is crucial for developing diagnostic tools that can accurately reflect the disease state.

Different subpopulations of EVs can carry distinct sets of EV markers. Analyzing EV profiles can help in identifying the type and stage of cancer. However, if EV heterogeneity is not considered, the diagnostic analysis might not capture the full range of disease-related EVs. Isolating specific EVs from a combined population is challenging, which can affect the sensitivity and specificity of diagnostic assays. Accurately identifying and quantifying specific molecules within EVs remains challenging, as existing cargo analysis techniques often lack the sensitivity or specificity required for comprehensive characterization. This limitation hampers efforts to clarify the functional significance of EVs and identify disease-specific biomarkers.^485^

Functional assays developed to evaluate the biological functions of EVs may not completely capture their intricacies, highlighting the necessity for strong tests that truly represent the complex roles of EVs. Modern methods used for analyzing EVs, like nanoparticle tracking analysis (NTA) and flow cytometry, frequently yield overall data that may not differentiate between various subgroups of EVs. This could result in a limited grasp of the diagnostic capabilities of EVs. Variables like the circumstances of blood collection, processing, and storage can greatly affect both the quantity and quality of EVs. And because standardized protocols are lacking for these preanalytical steps, variability in results may occur. Although EVs show potential as diagnostic biomarkers, there are multiple challenges in validating EV-based biomarkers for clinical application. Extensive research and thorough validation procedures must be conducted to confirm dependability and precision.

An absence of standardized tests and differences in sample handling make the validation process more complex. Developing regulations and quality controls for EV diagnostics is a challenging task. Robust guidelines and validation protocols are necessary to guarantee consistency and reproducibility in various laboratories and clinical settings.^408^

Moving from fundamental research to practical clinical use faces considerable obstacles, such as adhering to complex regulations, scaling up isolation techniques, and guaranteeing the effectiveness and safety of EV-driven diagnostics. Overcoming these obstacles is crucial for effectively translating EV research findings into clinical practice. As research on EVs progresses, ethical considerations relating to their use in diagnostics are becoming more significant. Ethical standards in research and clinical applications must address matters like informed consent, privacy, and fair access to EV-based technologies. To tackle these downsides and obstacles in EV research, it is necessary for diverse teams to work together.^486^ Establishing consistent methodologies, improving analysis techniques, verifying biomarkers, addressing translational challenges, and adhering to ethical standards are crucial for fully utilizing EVs in enhancing diagnostics.^486,487^

### 4.3 Challenges in Applying EV-Based Diagnostics

As discussed previously, EVs have garnered attention as promising biomarkers for various diseases, including cancer, owing to their capability to transport molecular cargo reflective of their cellular origin. However, the diverse nature of EVs in terms of size, composition, and cargo poses significant hurdles in standardizing isolation and analysis methods. This heterogeneity not only introduces variability in results but also complicates the establishment of consistent diagnostic criteria across different pathological conditions. Therefore, the application of EV-based diagnostics for pathological conditions presents numerous challenges that must be effectively addressed for successful clinical implementation.^488^ Current methods for isolating EVs from biological fluids often suffer from inefficiency and susceptibility to contamination, which can lead to false-positive results and compromised diagnostic accuracy. Techniques like ultracentrifugation (UC), size exclusion chromatography (SEC), and precipitation methods may inadvertently coisolate NEVs, exacerbating these challenges.^489,490^ In addition, the aforementioned isolation methods enrich EVs irrespective of their cellular source; both disease-associated and nondisease–associated EVs are isolated. As such, subtle molecular changes indicative of a particular disease state may be masked by the coisolation of nondisease–associated EVs.^491^

Biological fluids contain a diverse array of EV populations, derived from different cell types and tissues, posing challenges in discriminating disease-specific EVs from background noise and nonpathological EVs.^492^ Moreover, accurate quantification of EVs is paramount for diagnostic purposes, yet existing quantification methods, such as nanoparticle tracking analysis and flow cytometry, face limitations in sensitivity, specificity, and reproducibility. Addressing these limitations necessitates the standardization of quantification protocols and the development of more robust techniques.^493^

Preanalytical factors, such as sample collection, storage conditions, and processing methods, can significantly influence EV isolation and analysis. Standardizing preanalytical procedures becomes imperative to minimize variability and ensure consistency across studies. Additionally, identifying reliable disease-specific biomarkers within the complex cargo of EVs remains a formidable challenge. Comprehensive profiling of EV cargo and validation in large, well-characterized patient cohorts are essential steps to identifying robust biomarkers. Furthermore, the limited volume of clinical samples presents logistical challenges for EV-based diagnostics, particularly for certain patient populations. For example, techniques requiring large sample volumes may not be feasible for pediatric patients, and techniques requiring high EV concentrations may not be feasible for patients with early-stage disease. Moreover, EVs are sensitive to storage conditions, and ensuring their stability during sample storage is critical to maintaining the integrity of EVs and the accuracy of diagnostic results. In addition, the lack of standardized protocols, regulatory guidelines, and quality control measures for EV-based diagnostics impedes their clinical translation.^494,495^ Collaboration among stakeholders, including researchers, clinicians, regulatory agencies, and industry partners, is essential to address these challenges and facilitate the development of reliable EV-based diagnostic assays. Addressing these formidable hurdles requires continued research and innovation, crucial for realizing the full clinical utility of EV-based diagnostics for pathological conditions.

### 4.4 Traditional Methods for Analyzing EVs

Traditional methods for analyzing EVs in clinical samples are vital for the development of diagnostics, as they elucidate the role of EVs in various pathological conditions and identify disease-specific biomarkers. These methods, which characterize EVs from bodily fluids like blood, urine, saliva, and cerebrospinal fluid, include NTA, ELISA, electron microscopy, and flow cytometry.^496^ NTA enables real-time, high-resolution analysis of EV size and concentration, providing insights into their heterogeneity.^497^ ELISA quantifies specific proteins in EV samples for diagnostic purposes. Electron microscopy techniques, such as transmission electron microscopy (TEM) and scanning electron microscopy (SEM), visualize EV morphology and structure at high resolution. Flow cytometry analyzes EVs on the basis of size and surface protein expression, allowing for quantitative analysis of subpopulations and disease-specific biomarker detection.^498^

While these traditional methods offer valuable insights into EV characteristics, composition, and cargo, they come with several limitations. First, traditional isolation methods fail to provide pure EV samples. Biological fluids, such as blood or urine, are complex matrices, containing lipoproteins, protein aggregates, and cell debris, which may co-isolate with EVs. Such contaminants interfere with analysis, complicating result interpretation and introducing biases.^499^ UC in particular, while effective in isolating EVs, can coprecipitate contaminants and compromise EV sample purity. And immunocapture-based methods may inadvertently trap NEVs bearing similar surface markers, leading to false-positive results.^500^ Second, traditional methods may preferentially isolate certain types of EVs, which can also introduce bias. UC and density gradient centrifugation can yield low EV recovery rates, especially for smaller EVs, leading to incomplete characterization of an EV population.^501^ Traditional methods may also be biased toward isolating highly abundant EVs, overlooking less abundant EV populations that could be biologically significant.^502^ Third, traditional methods provide limited quantitative information about EVs, such as size distribution, concentration, and cargo content. While techniques like NTA quantify EV size and concentration, they do not offer comprehensive insights into EV cargo composition. Fourth, some traditional methods, UC in particular, can damage EVs. Mechanical stress or damage from high-speed centrifugation can alter EV properties, affecting downstream analyses and interpretation of EV function and cargo. And finally, traditional methods can be time-consuming and require expensive, specialized equipment. UC requires specialized equipment and long processing times.^503^ TEM and SEM require specialized equipment and sample preparation. As research in the field of EVs progresses, newer techniques are emerging to address these limitations.^504^

### 4.5 Analyzing EVs from Single Cells

Current approaches primarily reflect population-level data, obscuring cell-to-cell variability in EV secretion and function. SiEV analysis using state-of-the-art imaging approaches enables detailed profiling of molecular colocalization within individual EVs, yet still does not reveal which cell type or state the vesicle originated from.^143^ Recent advances have enabled direct detection and profiling of EVs secreted by individual cells. By integrating microfluidics, single-cell isolation, and high-sensitivity EV capture, researchers can now monitor EV secretion dynamics at the single-cell level. This allows precise mapping of cell-type-specific EV output and uncovers functional heterogeneity obscured in bulk analyses. Linking vesicle cargo to distinct cellular states such as activation, transformation, or therapeutic resistance offers powerful tools for cell-level diagnostics and mechanistic insights into intercellular communication.^502^ For example, Ji et al. created a high-throughput microchip-based platform capable of multiplexed surface marker profiling of EVs secreted by thousands of single cells. By integrating cytokine profiling, the study delineated functionally distinct cellular subgroups with dominant EV or cytokine secretion behaviors, offering a multidimensional view of intercellular communication at single-cell resolution.^505^ In addition, Nikoloff et al. introduced a nanoliter-scale microfluidic platform functionalized with monoclonal antibodies to capture EVs secreted from single cells. Using multicolor fluorescence imaging, they classified 15 distinct EV phenotypes and tracked secretion dynamics influenced by pathways such as neutral sphingomyelinase signaling. This technology provides a scalable, time-resolved framework to study EV secretion and uptake in real-time.^506^ Fathi et al. addressed another critical limitation in EV biology by tracing EV secretion to individual breast cancer cell clones. Their high-throughput single-cell cloning assay established that specific subsets of EVs linked to nonmetastatic phenotypes can be stably inherited, and their secretion dynamics mapped to distinct transcriptional states via single-cell RNA sequencing. Their single-cell RNA sequencing and EV profiling approach established a connection between transcriptional states and EV output, identifying inheritable secretion programs and providing insights into tumor heterogeneity and progression.^507^

Similarly, Zhou et al. investigated esophageal squamous cell carcinoma (ESCC) by combining single-cell RNA sequencing with exosomal RNA profiling to map the genetic origin and cellular diversity of EVs within the tumor microenvironment. Their analysis revealed that epithelial cells predominantly release EVs in malignant tissues, while endothelial cells and fibroblasts are the main contributors in nonmalignant regions. Notably, the study identified specific EV signatures associated with poor patient prognosis and developed a prognostic model based on exosomal RNA markers, highlighting the clinical relevance of understanding EVs' cell origins and content.^508^ Further emphasizing the clinical potential, Forte et al. integrated single-cell metabolic profiling with EV characterization in acute myeloid leukemia. By mapping the metabolic landscape of leukemic stem cells and immune subsets, and linking these to EV-derived surface markers, they identified metabolically vulnerable acute myeloid leukemia subtypes. Their approach unveiled prognostic EV signatures and opened avenues for personalized therapy using EV-based metabolic stratification.^509^ Recently, a functional analysis platform for EVs secreted from individual cells has been developed. By capturing and profiling EVs at the single-cell level, this technology enables high-throughput identification of cells with superior EV secretion capacity and highlights cell-to-cell heterogeneity that traditional bulk methods overlook. This approach not only deepens our understanding of EV biology but also facilitates the selection of potent therapeutic cell subpopulations for regenerative applications.^510^

Altogether, by characterizing EVs at the single-particle level, researchers can potentially uncover patient-specific biomarkers, monitor treatment responses, and stratify patients for personalized therapy. This precision approach enhances diagnostic performance, improves therapeutic targeting, and ultimately contributes to more effective and individualized healthcare.^502^

### 4.6 Importance and Advantages of SiEV Analysis

SiEV analysis plays a crucial role in advancing our understanding of the complex nature of EV populations. The heterogeneous characteristics of EVs, including size, cargo composition, and cellular origin, present significant challenges for conventional BuEV analysis methods.

SiEV analysis provides a nuanced approach that delves into the characteristics of an SiEV within a population, offering detailed insights that are often obscured in aggregate measurements. By examining SiEVs, researchers can uncover rare or unique vesicle subtypes with distinct functional roles in intercellular communication, disease pathogenesis, or therapeutic response. This level of granularity allows for the identification of specific biomolecules or signaling molecules enriched within certain EV subpopulations that could serve as crucial disease markers or targets for therapeutic intervention. Moreover, SiEV analysis enhances the sensitivity and specificity of disease diagnosis, prognosis, and monitoring therapeutic responses, as it enables the detection of rare biomarkers such as specific proteins, RNA species, and lipids from individual EVs.

Furthermore, SiEV analysis elucidates the dynamic processes underlying EV biogenesis, release, and uptake at the SiEV level.^511^ By tracking SiEVs, researchers can observe the kinetics of their secretion, their interactions with recipient cells, and the mechanisms involved in cargo delivery and uptake. This detailed understanding of EV dynamics provides valuable insights into the molecular mechanisms governing intercellular communication and signaling pathways.

SiEV analysis also enables functional profiling of SiEVs, offering a deeper understanding of their biological activities and effects on recipient cells.^5^ Researchers can study how SiEVs influence cellular signaling pathways, gene expression profiles, or phenotypic changes, providing critical insights into their functional consequences in both physiological and pathological contexts.^512^

In addition to its scientific applications, SiEV analysis holds significant promise for clinical applications. By enabling the characterization of SiEV profiles from patient samples, this approach offers the potential for personalized medicine. Researchers can identify patient-specific biomarkers, monitor disease progression, and tailor therapeutic interventions targeting specific EV subpopulations or cellular pathways. This personalized approach enhances diagnostic accuracy, treatment efficacy, and patient outcomes across various diseases, making SiEV analysis a powerful tool for precision medicine.^513,514^

While the potential of SiEV analysis is clear, several technological challenges remain. These challenges include ensuring scalability, cost-effectiveness, and the development of high-throughput platforms suitable for routine clinical use. As SiEV analysis technology continues to evolve, it is expected to become an invaluable tool in clinical diagnostics, therapeutic monitoring, and personalized medicine. The importance of SiEV analysis lies in its ability to uncover the intricate biological properties of EVs, providing invaluable insights into their heterogeneity, functional diversity, and interactions with recipient cells. Ultimately, SiEV analysis holds the key to unlocking the full diagnostic, prognostic, and therapeutic potential of EVs in a wide range of biomedical applications.

## 5 Recent Advances in EV and SiEV Isolation Techniques

For both BuEV and SiEV analyses, a crucial first step is isolating a substantial quantity of EVs from clinical samples, but the objectives of EV isolation vary between the types of analysis. Isolation techniques for BuEVs aim to obtain diverse EV samples for comprehensive analysis, crucial for biomarker discovery, therapeutic applications, and ensuring reproducibility in research. On the other hand, isolation techniques for SiEVs aim for intact structure, high bioactivity, and high purity, allowing for detailed analysis of individual vesicles, possibly identifying rare EV subpopulations and providing high-resolution insights into their specific roles and functions. Both objectives are essential for advancing clinical research and applications.

Traditional methods of isolating EVs, such as UC, density gradient centrifugation, and precipitation-based techniques, pose significant challenges for clinical translation. In recent years, and as mentioned previously, there has been a surge in the development of advanced techniques aimed at overcoming challenges associated with UC and improving EV isolation from clinical samples. These advanced techniques offer enhanced efficiency, purity, and scalability, making them more suitable for clinical applications, including personalized medicine and early disease diagnosis. This section provides an overview of recent advances in EV isolation techniques.

### 5.1 Integrated Techniques in Ultracentrifugation

Since its use in pioneering EV studies, UC has been a fundamental technique for isolating EVs because of its simplicity and widespread availability. By applying high centrifugal forces and leveraging the principles of sedimentation and buoyancy, UC progressively separates particles on the basis of their size and density, removing debris and larger vesicles while concentrating EVs. However, as mentioned previously, the technique has notable drawbacks. Advances aiming to optimize UC include density gradient UC, which separates EVs from other cellular debris and contaminants on the basis of size and density. This technique improves EV yield and purity compared to that of traditional UC, enabling more precise SiEV analysis without the contamination of larger cellular components.^515^ However, despite these improvements, the yield and purity are often still insufficient for downstream analyses, and the technique is time-consuming.^516^

To overcome these limitations, researchers have improved centrifugation-based EV isolation by combining centrifugation with other techniques, such as sucrose gradient ultracentrifugation (SGUC), size-based filtration–UC, or size-exclusion chromatography.^495^ These integrated methods aim to enhance EV purity by separating particles on the basis of both size and density, ultimately improving the efficiency of EV isolation.^517^ SGUC, a form of density gradient UC, uses a continuous density gradient, typically composed of materials like sucrose or iodixanol, to achieve greater resolution in EV separation, resulting in highly pure exosome populations. Recent improvements in this technique, such as optimizing gradient composition and automation, have led to higher exosome recovery rates and reproducibility.^518^ Improving exosome purity even further, size-based filtration–UC combines SEC with UC and a sucrose density gradient, effectively removing contaminants and isolating EVs on the basis of size and density.^79^ The highly pure exosome populations resulting from this technique are ideal for SiEV analysis. Furthermore, combining size exclusion chromatography with UC and a sucrose density gradient (SBF-UC) results in highly pure exosome populations. By effectively removing contaminants and isolating EVs on the basis of size and density, this method results in highly pure exosome populations, ideal for SiEV analysis.^79^ UC has also been improved by combining it with asymmetric flow field-flow fractionation (AF4). While UC often involves multiple centrifugation steps, which can prolong the separation, the AF4 technique enhances the separation process through continuous flow fractionation, effectively minimizing processing delays. In the integrated approach, UC is used first to concentrate EVs from biological fluids, followed by AF4, which further fractionates the EVs into distinct subpopulations. This combined approach allows for efficient EV isolation, minimizing the contamination from NEVs and resulting in high yield and purity, facilitating more accurate downstream analyses.^519^

### 5.2 Size Exclusion Chromatography

SEC is a fundamental technique for isolating EVs from biological samples, and recent advancements in this and associated techniques have substantially improved the efficiency, scalability, and purity of EV isolation, particularly in clinical applications.^269^ SEC operates on the principle of separating particles by size. In SEC, smaller molecules, such as free DNAs, RNAs, proteins, and small lipoproteins, penetrate the porous matrix of the stationary phase, while larger components, such as cellular debris and aggregated proteins, are excluded or slowed down. This allows EVs, which are larger than small proteins but smaller than cellular debris, to elute in a distinct fraction, enhancing the purity of the isolated EVs.^520^

Several advanced techniques built upon SEC principles have emerged to enhance EV isolation from clinical samples. In one advanced technique, density gradient UC is first used to deplete lipoproteins, including high-density lipoproteins, from plasma before isolating EVs via SEC. The initial density gradient UC step significantly reduces contaminants, improving the efficiency of subsequent SEC by further eliminating residual lipoproteins and plasma proteins. The combination of density gradient UC and SEC not only enhances the purity of the isolated EVs but also enables rapid and automated isolation from small clinical samples. This streamlined process is particularly advantageous for developing biomarker assays, as it facilitates the swift and accurate detection of disease-specific EV signatures, critical for diagnostic and therapeutic applications.^521^ In another advanced technique, immunocapture based on SEC utilizes antibodies specific to common EV surface markers, such as CD63, CD9, CD81, or cancer markers, to capture EVs. Initially, EVs are enriched using immunocapture beads targeting the desired markers. Subsequently, SEC is employed for further purification and separation from captured contaminants, ensuring high specificity and purity.^522^

Another advanced technique integrates dead-end filtration with SEC to isolate EVs from complex samples. Initially, dead-end filtration removes large particles and debris, and the filtrate undergoes SEC to separate EVs based on size and elution profile, providing high-purity EV isolates suitable for downstream applications. This integrated approach improves the efficiency of EV isolation by reducing non-EV contaminants and enhancing purity compared to the traditional SEC method.^503^

Another technique integrates multiangle light scattering with SEC, providing detailed information on EV composition and structure and enhancing data analysis capabilities. This integration surpasses the capabilities of traditional methods, offering a deeper understanding of EV properties through precise characterization of size distribution and molecular weight, which are crucial for SiEV analysis.^523^

Finally, isolating EVs by integrating microfluidic devices with SEC is gaining traction, owing to the capability of these devices to precisely control fluid flow and separation processes, leading to more consistent and reliable results.^524^ These microfluidic SEC devices enable rapid, automated, and high-throughput isolation of EVs from small sample volumes. Compared to traditional SEC methods, these devices also reduce sample loss and provide rapid and more consistent and reliable results.

Compared to the SEC traditional methods, all the advanced techniques based on SEC enhance the efficiency, purity, and scalability of EV isolation from clinical samples. They facilitate the utilization of EVs in various biomedical applications by providing higher-quality isolates suitable for detailed SiEV analysis.

### 5.3 Immunoaffinity-Based Isolation

Immunoaffinity-based isolation of EVs is invaluable for selectively extracting EV subpopulations from complex biological samples. This method utilizes antibodies targeting specific EV surface markers (CD63, CD9, or CD81), the tetraspanins expressed on both disease-associated and nondisease–associated EVs, and specific markers, such as cancer biomarkers (EpCAM, PD-L1, or EGFR) or other biomolecules that ensure high purity and efficiency in EV capture.^525^ In addition, alternative bioaffinity agents can be used, such as aptamers.^207^ For example, He and colleagues reported novel 3D-structured nanographene immunomagnetic particles (NanoPoms) with unique flower pom-pom morphology and photoclick chemistry, enabling the specific marker–defined capture and release of intact exosomes.^526^

Various platforms are utilized for effective immunoaffinity-based isolation, including beads, nanoparticles, microfluidic chips, membranes, and surfaces, all coated with antibodies targeting antigens,^527^ including EV surface markers.^269^ For example, in antibody-coated magnetic bead isolation, antibodies specific to EV surface markers are conjugated onto magnetic beads, which are typically larger than the EVs. When mixed with a sample containing EVs, the smaller EVs bind to the larger beads via the specific antibodies (Figure 8A,B).^528,529^ After binding is allowed to occur, a magnetic field is applied to separate the EV-bound beads from the sample matrix, and nontarget components are washed away.^530^ This technique ensures high specificity and efficiency, enabling the isolation of EVs expressing specific surface markers. Compared to traditional isolation methods, it improves the purity and yield of specific EV subpopulations.

**Figure 8:**
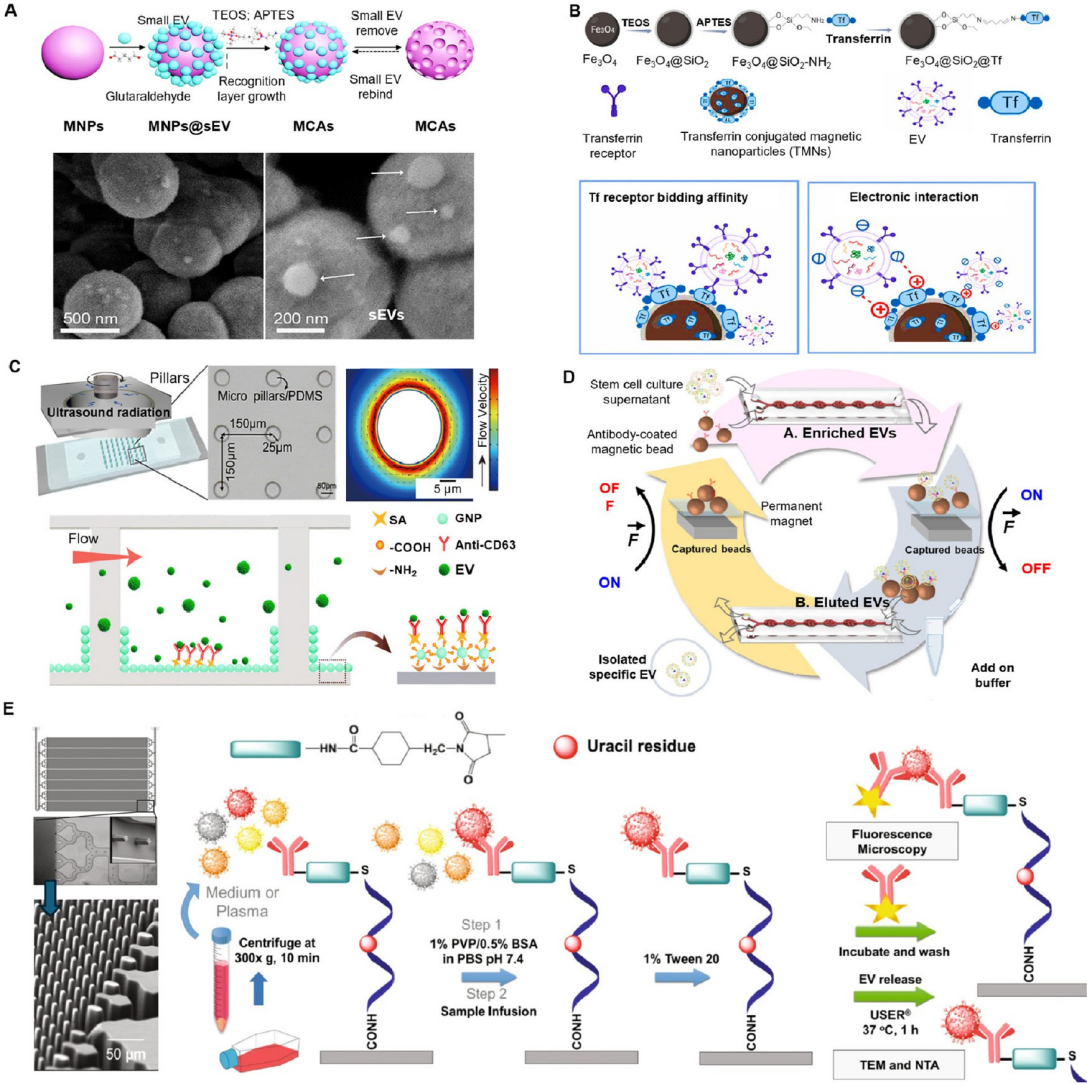
Integrated techniques for immunoaffinity-based EV isolation. (A) Magnetic colloid antibodies (MCAs) accelerate the isolation of small extracellular vesicles (sEVs). This system integrates V-Chip technology with an MCA-based sandwich enzyme-linked immunosorbent assay (ELISA), allowing for the sensitive detection and quantification of tumor markers from sEVs in plasma, facilitating rapid, point-of-care cancer diagnostics. Copyright 2021, American Chemical Society. Reprinted with permission from ref 528. (B) Transferrin-conjugated magnetic nanoparticles (TMNs) are used to isolate brain-derived exosomal microRNAs from the plasma of patients with neurological disorders. These TMNs target transferrin receptors, enriching brain-derived exosomes for more precise molecular diagnostics. Copyright 2024, Elsevier. Reprinted with permission from ref 529. (C) Enhanced immune capture of EVs using gelatin nanoparticles (GNPs) and acoustic mixing within an acoustofluidic device. The device directly isolates antibody-treated EVs in a microfluidic channel, where acoustic streaming enhances the binding between antibodies and vesicles. GNPs functionalized with specific CD63 antibodies are introduced to the channel surface, creating a rough texture that increases EV capture efficiency. Copyright 2021, Royal Society of Chemistry. Reproduced with permission from ref 531. (D) Dual-mode horseshoe-shaped orifice micromixer (DM-HOMM) microfluidic chip–based strategy enables continuous isolation of stem cell–derived extracellular vesicles (SC-EVs). Recycled magnetic beads within the microfluidic channels enrich and elute specific SC-EVs, allowing for efficient, continuous separation. Copyright 2023, Springer Nature. Reproduced with permission from ref 534. (E) EV microfluidic affinity purification chip for the affinity selection of EVs (schematic on left). The chip consists of 1.5 million micropillars (10 μm effective diameter) with a spacing of 10 μm that are packed into 7 separate beds placed in a parallel arrangement, which allows for high-speed processing and a large surface area to produce a large analytical dynamic range. The accompanying images of the chip (middle) were obtained using rapid scanning confocal microscopy. The chip was made via injection molding of the plastic, cyclic olefin polymer. Catch and release of EVs affinity selected by using the EV-MAP chip (schematic on right). The antibodies are attached to the plastic surface with a uracil-containing ssDNA molecule and the captured EVs are released enzymatically Copyright 2020, Springer Nature. Reproduced with permission from ref 206.

In another example, nanoparticle-based immune isolation utilizes nanoparticles coated with antibodies to capture EVs. These antibody-coated nanoparticles, typically smaller than EVs, are introduced to samples containing EVs, and the nanoparticles selectively bind to target EVs. After binding is allowed to occur, EV-bound nanoparticles are separated from the sample matrix by using centrifugation or filtration techniques. This approach offers high sensitivity and is adaptable for isolating EV subpopulations present in low abundance. It is valuable for detecting disease-specific EV biomarkers and studying EV heterogeneity, offering a level of specificity and purity that traditional isolation methods cannot achieve (Figure 8C).^531^

In yet another example, microfluidic chip platforms integrate immunoaffinity capture with microfluidic technology. Antibodies targeting EV surface markers are immobilized within microfluidic channels, enabling selective capture of EVs.^525^ Microfluidic systems allow for rapid isolation, reduced sample volumes, and improved reproducibility.^532^ They are suitable for point-of-care diagnostics and high-throughput screening, offering precise and automated EV isolation that surpasses traditional immunoaffinity isolation methods in terms of speed and efficiency.^533^ The dual-mode horseshoe-shaped orifice micromixer (DM-HOMM) chip provides an advanced immunoaffinity-based method for the efficient isolation of specific stem cell–derived extracellular vesicles (SC-EVs). The enrichment phase of the chip is optimized to achieve high binding efficiency by adjusting the size and concentration of magnetic beads within the micromixer. In the elution phase, the antigen–antibody bonds are disrupted, allowing the selective separation of SC-EVs. The isolated SC-EVs retain their intact morphology and exhibit uniform size and concentration. Additionally, these vesicles demonstrate wound-healing properties. This continuous isolation process using the DM-HOMM chip offers a reliable approach for producing standardized EVs (Figure 8D).^534^ Other microfluidic chip platforms integrate nanomaterial-based immunoaffinity capture with microfluidic technology for isolating specific subtypes of EVs. The exosomes track-etched magnetic nanopore (exosomes TENPO) chip integrates nanomaterials with microfluidic technology for efficient, sensitive, and scalable isolation of exosomes directly from unprocessed serum or plasma. The chip uses superparamagnetic nanoparticles coated with biotinylated antibodies to target exosomal markers such as tetraspanins (CD9, CD81) or tumor-specific markers like EpCAM. Exosomes are magnetically captured via a disc magnet, and the device features a track-etched polycarbonate membrane with engineered nanopores that exclude contaminants while allowing exosome capture. A multilabeling approach ensures efficient exosome capture by overcoming drag forces, enhancing sensitivity. After capture, exosomes are lysed on-chip, and their mRNA cargo is analyzed using quantitative PCR. The exosomes TENPO chip represents a significant advancement in EV isolation and analysis, offering a sensitive, efficient, and scalable platform for the capture and downstream molecular profiling of tumor-derived exosomes. By integrating immunomagnetic labeling with innovative nanopore technology, this platform holds great promise for clinical applications in cancer diagnostics and beyond.^381^

Immunoaffinity-based techniques are often combined with other methods to further enhance isolation efficiency.^518^ For instance, they can be combined with the SEC for further purification. In this approach, EVs are first enriched using immunocapture beads targeting specific markers, and then SEC is employed to separate EVs from contaminants. This ensures high specificity and purity, enhancing isolation efficiency and enabling detailed SiEV analysis. As another example, L1CAM-expressing neuronal EVs were isolated by using a combination of SEC and a microfluidic chip comprised of pillars (1.5 million pillars) to which anti-L1CAM antibodies were surface-immobilized.^221^ Because L1CAM protein can exist in plasma in its free form, SEC was used first to remove free L1CAM protein from a plasma sample, followed by affinity selection of the L1CAM-expressing EVs. In this case, the EV-selection microfluidic chip (EV-MAP) was made from plastic via injection molding to allow for high-scale production at low cost, making it appropriate for clinical applications. The capture antibodies could be linked to the surface of the pillars using an ssDNA molecule that could be cleaved enzymatically through the presence of the uracil residue in the ssDNA (Figure 8E).^206^

Overall, compared to traditional methods, these immunoaffinity-based techniques offer significant improvements in the specificity, purity, and yield of EV isolation, making them invaluable for SiEV analysis in various biomedical applications.

### 5.4 Polymer-Based Precipitation

Polymer-based precipitation is widely used for isolating EVs from complex biological samples, including patient blood samples in clinical applications.^535^ In this method, a polymer solution containing polyethylene glycol and polyvinylpyrrolidone induces EVs to aggregate and subsequently settle, allowing for their separation from other sample components.^536^

Polymer-based precipitation offers advantages such as simplicity, scalability, and rapid isolation without requiring UC, but this method also faces several challenges. Specifically, the particles used in the precipitation process (often made from polymers) can exhibit uneven sizes, especially due to the formation of EV agglomerates, which may compromise the reproducibility of the isolation process.^490^ In addition, removing these polymer particles can be challenging, and the mechanical forces involved in the technique may damage the EVs or hinder their surfaces due to chemical additives. Concerns also exist regarding low purity and recovery rates, as well as the presence of contaminants such as proteins and non-EVs, which can adversely affect analytical accuracy in clinical applications.^537^ To address these challenges, researchers have developed modified precipitation protocols, using other polymers like polyethylenimines, for example, to optimize EV recovery and minimize sample contamination. One notable advancement involves using specialized polymers with tailored properties to enhance EV recovery and purity. These polymers selectively interact with EVs while minimizing nonspecific binding to other sample components, resulting in cleaner EV isolates.^538^ Other adjustments may involve varying factors such as polymer concentration, incubation time, and centrifugation parameters to achieve better isolation outcomes.^539^

In recent years, researchers have explored integrating polymer-based precipitation with other techniques, such as SEC, to enhance EV isolation efficiency. This combination typically involves a sequential approach: first, the sample undergoes polymer-based precipitation to precipitate and concentrate EVs while reducing contaminants, such as proteins and NEVs; and second, the resulting precipitate is run through an SEC column for further purification. This two-step process maximizes the purity and quality of isolated EVs, as SEC effectively separates the concentrated EVs from residual contaminants on the basis of size.^540^

Polymer-based precipitation has also been integrated with nanomaterials, such as nanoparticles and nanofibers, which has been particularly impactful. Nanomaterials offer unique properties that facilitate EV capture and purification while minimizing nonspecific binding. For example, functionalized magnetic nanoparticles coated with specific ligands or antibodies selectively bind to EV surface markers, enhancing EV isolation efficiency in blood samples.^529^ Nanofiber-based matrices, often functionalized with affinity ligands or antibodies, provide a high surface area for EV binding and are compatible with polymer-based precipitation methods. The integration typically involves sequential steps, rather than a single mixture. First, the polymer is dissolved in a suitable solvent to create a precipitation solution. Second, nanomaterials are added to this solution, either prefunctionalized or as part of a subsequent step, enhancing the specificity of EV capture. Third, the biological sample is introduced to the mixture, allowing the polymer to promote EV aggregation while the nanomaterials enhance binding capacity. And fourth, separation techniques, such as magnetic capture for nanoparticles or filtration for nanofibers, are employed to isolate the EVs. This multistep approach leverages the strengths of both polymers and nanomaterials, optimizing EV isolation.^541^ Furthermore, nanomaterials can serve as carriers for EV isolation reagents, delivering the precipitation reagents specifically to EVs and promoting their aggregation and isolation.

Polymer-based precipitation can be effectively integrated with microfluidic devices that incorporate nanomaterials, allowing for the precise manipulation of EVs within microscale channels. In this setup, polymer-based precipitation reagents can be injected into the microfluidic chip, where they interact with the EVs, facilitating their selective precipitation. This integration reduces the overall volume of reagents required and enables rapid mixing and reaction times within the confined microfluidic environment, enhancing the efficiency of EV isolation. In addition, this integration improves the scalability of the process, as multiple channels can be designed to operate in parallel, increasing throughput. These devices enable rapid and automated isolation of EVs from small clinical samples, which is crucial for developing biomarker assays that can quickly and accurately detect disease-specific EV signatures. This capability makes them highly suitable for point-of-care diagnostics and personalized medicine applications.^517^

Thus, polymer-based precipitation represents an advancement in SiEV analysis, especially when integrated with advanced nanomaterials. Such integrated isolation techniques offer improved precision, enhanced purity, and broader applicability in clinical settings. By refining EV isolation processes and enabling detailed molecular profiling of individual vesicles, polymer-based precipitation contributes significantly to advancing our understanding of EV biology and accelerating the development of EV-based diagnostics.

### 5.5 Microfluidic Devices

As already mentioned, microfluidic devices offer significant potential for isolating EVs from clinical samples. These systems use small channels and precise fluid manipulation to efficiently capture and purify EVs.^533^ As a main advantage, microfluidic devices do not require more than a small sample volume, which is crucial for isolating EVs from clinical samples in which volume is limited, such as cerebrospinal fluid, saliva, and tears. A new microfluidic approach to streamline and expedite the exosome analysis pipeline by integrating specific immunoisolation and targeted protein analysis of circulating exosomes. This represents one of the first microfluidic approaches to immuno-isolate and target protein biomarkers within EVs. Using this device, they were able to assess the total expression and phosphorylation levels of IGF-1R in NSCLC patients by probing plasma exosomes as a noninvasive alternative to conventional tissue biopsy.^542^ Microfluidic isolation can also detect low-abundance EVs more effectively, enhancing its diagnostic potential for early disease detection. For example, Zhang and colleagues engineered a microfluidic chip with a self-assembled 3D herringbone nanopattern that detected low levels of tumor-associated exosomes in plasma (10 exosomes/μL) that would be undetectable by standard microfluidic systems for biosensing.^543^ Moreover, microfluidic devices provide rapid EV isolation, reducing processing time compared to that of traditional isolation methods. This is specifically important for point-of-care applications, where timely diagnosis and treatment are critical.

Many variations of microfluidic chips have been developed to improve EV isolation. For example, microfluidic devices with multiplexed capture zones enable the simultaneous isolation of multiple EV subpopulations from a single sample.^544,545^ A novel integrated droplet microfluidic system has been developed, comprising a droplet generator, incubator, and magnetic bead extraction module. This system is specifically designed for efficient EV isolation. Compared to other microfluidic platforms utilizing droplet-based affinity capture, it offers a significantly higher isolation throughput (400 μL/h) and the ability to process larger sample volumes (up to 2 mL). This strategy ensures complete infusion of the sample, eliminating losses in containers or capillaries. This feature is crucial for diagnostic applications, where sample volumes range from hundreds of microliters to a few milliliters (Figure 9A).^546^ Viscoelastic microfluidics allow for the direct isolation of small EVs from human blood. In one example, researchers extracted small EVs from the blood of 20 cancer patients and 20 healthy donors and isolated small EVs with UC or viscoelastic microfluidics. When viscoelastic microfluidics were used for isolation, the concentrations of small EVs were higher, and the size distribution was more uniform, with a greater proportion of smaller vesicles detected. This suggests that viscoelastic microfluidics can provide a more accurate representation of the small EV population in blood samples, potentially improving the sensitivity of downstream analyses for biomarkers in cancer diagnostics (Figure 9B).^547^ Another innovative example is conductive spiral microfluidic chips combining hydrodynamic and dielectrophoretic forces, which enhance separation efficiency. These chips isolate uniformly sized exosomes from complex environments, such as cell culture media, showcasing their rapid, straightforward, and efficient exosome isolation capabilities.^548^ And there are other examples of using external forces or hydrodynamic properties to manipulate EVs in microfluidic chips, including active methods, such as acoustic-, electric field-, and filtration-based techniques, and passive methods that rely on physical constraints within microfluidic channels.^549^ These approaches enable precise control over EV separation and purification. Microfluidic chip–based isolation can also be enhanced by nanotechnology. Chips can incorporate functionalized nanoparticles coated with specific ligands or antibodies that can selectively bind to EVs, improving capture efficiency and specificity during the isolation process.^546^ Nanofilters or nanoporous membranes integrated into microfluidic channels can selectively trap EVs on the basis of their size, with adjustable pore sizes allowing for high-purity isolation.^544,550^

**Figure 9:**
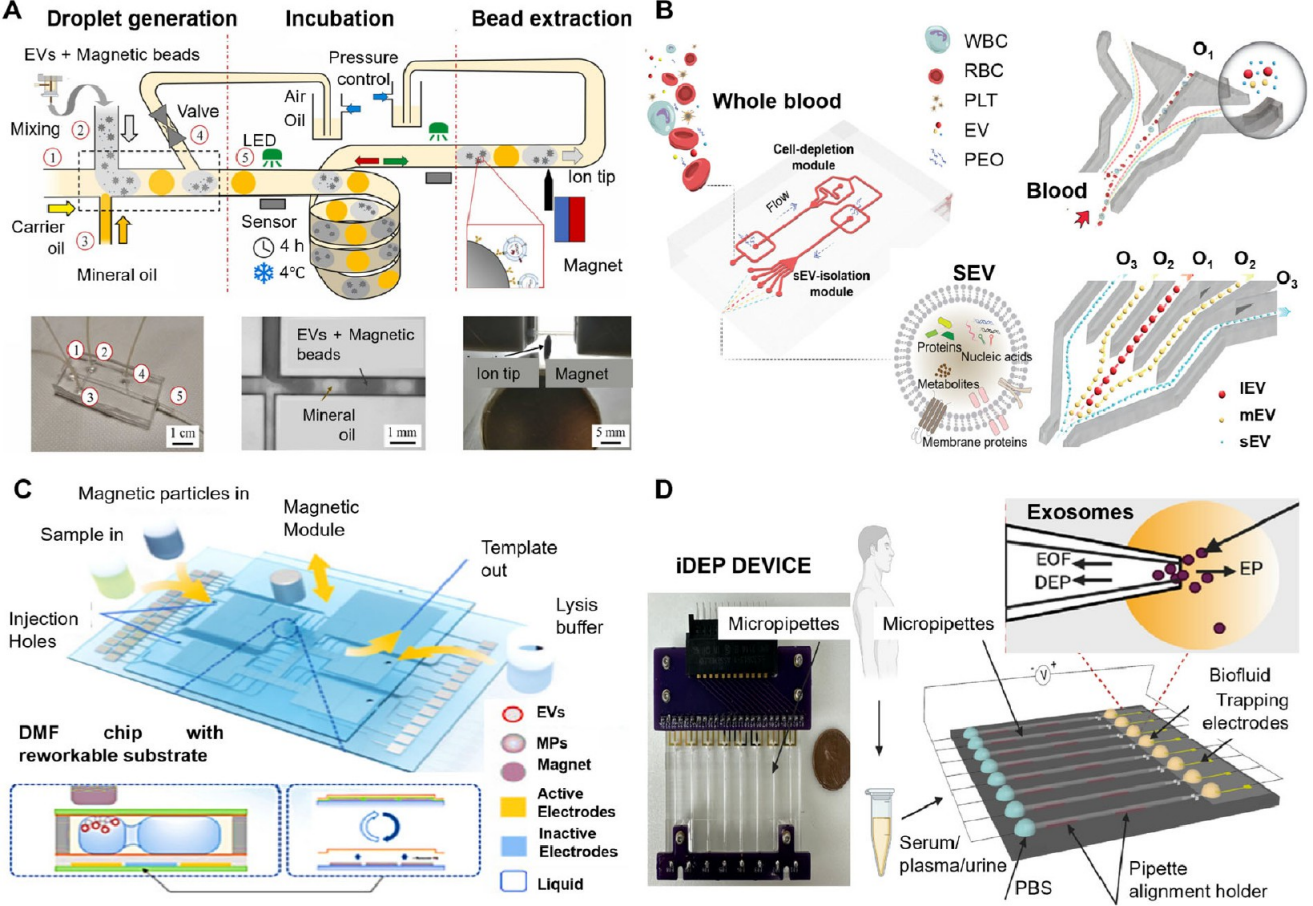
Recent advancements in microfluidic technology for EV isolation. (A) Integrated droplet microfluidic system featuring a droplet generator, incubator, and magnetic bead extraction module. Droplet generation is controlled via three inlets (1, 2, 3), while outlet 5 allows droplets to enter a storage capillary without breakup. Inlet 4 connects to a pressure controller. Droplet generation occurs at a double T-junction, alternating aqueous phases with immiscible fluorinated oil. The bead extraction module utilizes a magnetizable iron tip to facilitate magnetic bead extraction as droplets are transported through the capillary. Copyright 2024, Elsevier. Reprinted with permission from ref 546. (B) Viscoelastic microfluidic system for sEV separation from whole blood. The device comprises two sequential modules: a cell-depletion module, where blood components larger than 1 μm (WBCs, RBCs, PLTs) are removed at outlet O_1_, and an sEV-isolation module, where larger EVs (lEVs) and medium-sized EVs (mEVs) are collected at outlet O_2_, and sEVs are isolated at outlet O_3_. Copyright 2023 AAAS. Reprinted with permission from ref 547. (C) Digital microfluidic (DMF) platform automates the conventional magnetic microparticle (MP) affinity-based EV isolation process, integrating EV isolation, washing, and lysis into a streamlined workflow. Copyright 2024, Elsevier. Reprinted with permission from ref 459. (D) Insulator-based dielectrophoretic (iDEP) device generates a trapping zone at the micropipette tip by balancing the dielectrophoretic (DEP) force with electrokinetic forces such as electroosmosis (EOF) and electrophoresis (EP), enabling sEV purification from serum, plasma, and urine samples. Copyright 2023, Springer Nature. Reproduced with permission from ref 551.

Recent advancements in microfluidic technology have led to the development of digital microfluidic platforms, which automate conventional magnetic microparticle affinity-based EV isolation. This innovation significantly reduces isolation time, from 1 to 3 h to just 20–30 min, achieving over 77% EV isolation efficiency. Digital microfluidic platforms manipulate small liquid volumes by using programmable electric fields, enhancing the precision and automation of fluid handling processes. These systems can be seen as a specialized variation of traditional microfluidic devices (Figure 9C).^459^ Moreover, microfluidic systems can incorporate insulator-based dielectrophoretic devices, which create trapping zones at micropipette tips, facilitating the isolation of small EVs from human biofluids (Figure 9D).^551^ A notable advancement is the integration of a microfluidic chip with an antibody barcode biochip. This setup achieves 82% filtration efficiency, effectively removing vesicles larger than 200 nm. The antibody barcode biochip utilizes antibodies tagged with unique barcodes for the identification and capture of specific EV populations, enhancing both separation and detection capabilities. This integrated approach has successfully separated and characterized EV subgroups from clinical samples, including serum, cerebrospinal fluid, and cell supernatants.^552^

Microfluidic isolation techniques have advantages over BuEV analysis methods by providing selective, sensitive, rapid, and controlled isolation of SiEVs. These capabilities are pivotal for advancing our understanding of the biology of EVs and exploiting their potential in clinical settings.

### 5.6 Membrane-Based Isolation Techniques

Membrane-based isolation techniques use porous membranes to isolate EVs from smaller molecules. Basically, by applying pressure or using tangential flow, a sample is pushed through a membrane.^553^ The membrane acts as a barrier, allowing small molecules and fluid to pass through while catching larger particles like EVs.^554^ The size and surface properties of the membrane pores can be customized to enhance EV capture and minimize nonspecific binding.

One commonly used membrane-based technique is ultrafiltration, which uses pressure differentials to drive a sample through a porous membrane. Ultrafiltration membranes have pore sizes ranging from a few nanometers to hundreds of nanometers, allowing selective retention of EV particles while enabling smaller molecules to pass through the membrane. The result is better EV purity than that of UC, which may coprecipitate contaminants such as lipoproteins and protein aggregates.^555^ Ultrafiltration is advantageous for processing relatively large sample volumes and can be automated. However, traditional ultrafiltration methods often encounter issues like membrane fouling and clogging, which can reduce separation efficiency and result in sample loss.^553^ One variation of ultrafiltration is tangential flow filtration, which allows continuous processing of large sample volumes, making it suitable for industrial-scale EV isolation. But this method requires specialized equipment and may also face clogging problems.^556^

To overcome these and other challenges, researchers have developed advanced membrane-based techniques, focusing on improving the efficiency, speed, and scalability of membrane-based EV isolation. In recent years, novel membrane materials with optimized pore sizes, surface chemistry, and antifouling properties have been explored to enhance EV capture efficiency and reduce the common issue of membrane fouling.^557^ One such approach for isolating bovine milk exosomes combines tangential flow ultrafiltration with electrophoretic oscillation to address fouling. Fouling occurs when larger particles obstruct nanopores, impeding exosome isolation, and it is particularly a problem in microultrafiltration using silicon nitride (SiNx) membranes. This novel approach mitigates fouling by introducing low-voltage, low-frequency electrical field oscillations that induce particle movement within the nanopores, effectively dislodging trapped particles. The tangential flow then transports these loosened particles to a reservoir, enhancing filtration efficiency (Figure 10A).^555^ In another innovation, a nanofluidic device integrates 2 microfluidic chips, forming a dual-function system. A filtration chip efficiently separates EVs via an alumina nanochannel array membrane, and a detection chip captures and analyzes EVs. This cost-effective, highly efficient, and automated device enabled direct separation of EVs from serum or cerebrospinal fluid with an efficiency of 82%, followed by in situ detection. The compact design provides a powerful tool for high-efficiency EV identification and analysis, offering significant potential for clinical diagnostics (Figure 10B).^557^ Another advanced membrane-based technique is a zwitterionic tandem membrane system for exosome isolation. This system features a 200 nm pore poly­(vinylidene fluoride) (PVDF) filter and a 30 nm pore sulfobetaine methacrylate hydrogel-modified cellulose triacetate (CTA) filter, facilitating precise exosome separation. The inclusion of the hydrogel-modified CTA filter enhances the biocompatibility and bioactivity of the materials, meeting critical requirements for biomedical applications. This innovative approach leverages the unique properties of zwitterionic materials to optimize exosome isolation, ensuring high yield and purity (Figure 10C).^558^ Moreover, a novel microfluidic technique, termed “catch and display for liquid biopsy” (CAD-LB), rapidly isolates and characterizes EVs at the single-vesicle level using ultrathin nanoporous silicon nitride (NPN) membranes. In this technique, minimally processed EV-containing samples are pipet-injected into a microfluidic device, where EVs are fluorescently labeled with carboxyfluorescein succinimidyl ester (CFSE) and an antibody targeting a specific biomarker. After labeling, EVs are captured within the nanopores of the NPN membrane, filtering out unreacted antibodies and contaminants. The captured EVs are then analyzed via fluorescence microscopy to detect CFSE and antibody signals, indicating biomarker-positive EVs. This platform significantly enhances the specificity and sensitivity of EV analysis, advancing liquid biopsy applications for personalized medicine (Figure 10D).^559^ Finally, membrane coatings with functional groups can be tailored to selectively bind EVs via surface markers or biochemical properties. This customization allows researchers to isolate specific EV subpopulations, improving the specificity of capture and enabling detailed downstream analysis.^560^ An example is the ultrafast-isolation system EXODUS, which uses negative pressure oscillations and double-coupled harmonic oscillator–enabled membrane vibration for rapid and automated exosome purification for clinical samples.^227^ This approach inhibits fouling effects, enabling clog-free purification of exosomes.

**Figure 10:**
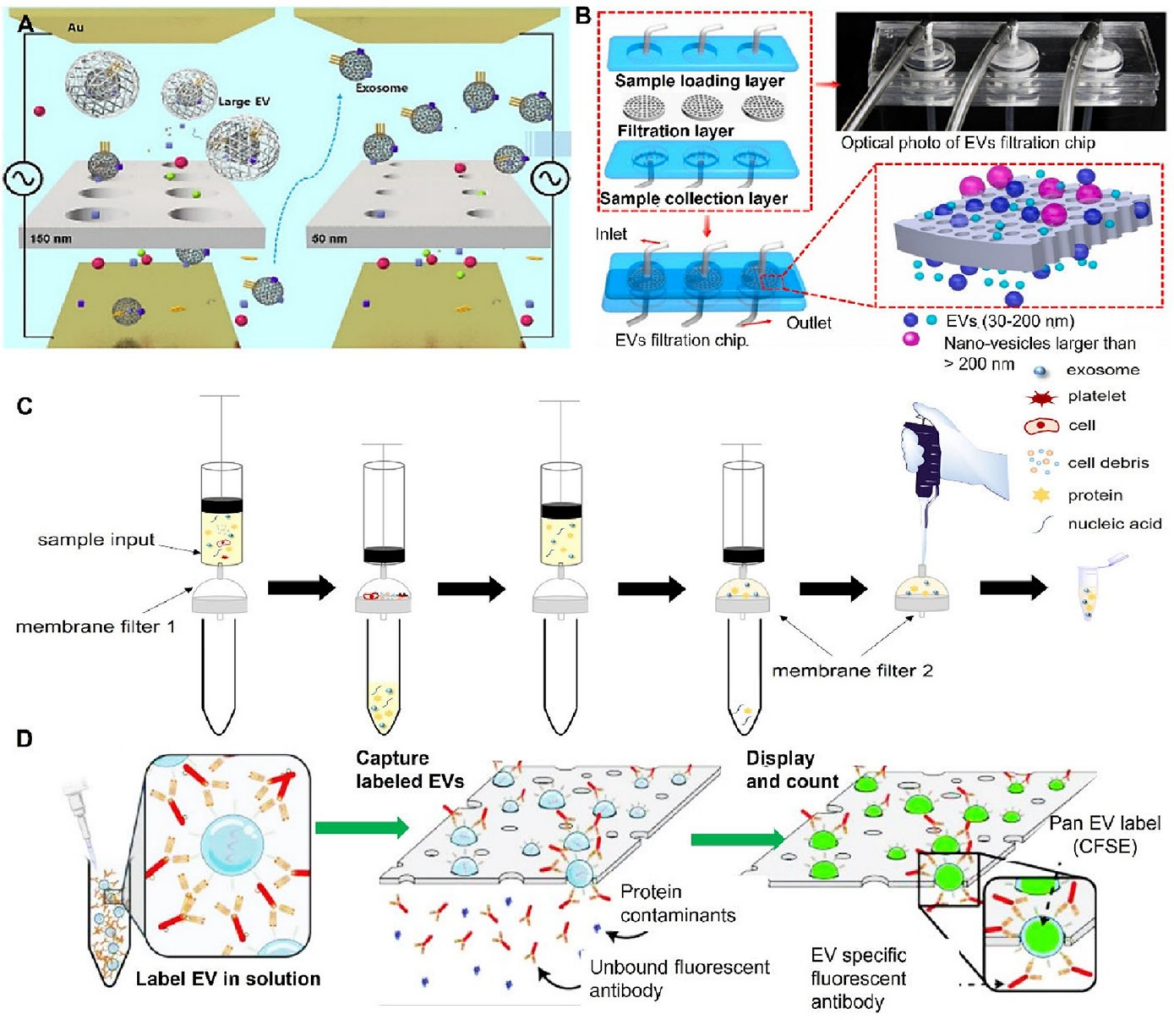
Advanced integrated membrane technologies for EV isolation. (A) Isolation of bovine milk exosomes using electrophoretic oscillation-assisted tangential flow filtration with antifouling microultrafiltration membranes. The oscillations assist in driving exosomes through antifouling silicon nitride (SiNx) microultrafiltration membranes and prevent pore blockage by larger particles. Copyright 2023, American Chemical Society. Reprinted with permission from ref 555. (B) Microfluidic device for precise, size-based EV separation via alumina nanochannel array membranes. The membranes are positioned between the sample loading and collection chambers. The top loading chamber contains an inlet for sample introduction, while the collection chip comprises 5 supporting circular layers (3 mm in diameter) to stabilize the membrane during filtration. Blocked vesicles are periodically flushed by reversing flow with washing buffer through bottom-to-top channels, maintaining membrane efficiency and allowing for continuous isolation. Copyright 2024, Elsevier. Reprinted with permission from ref 557. (C) Sequential filtration of exosomes using a zwitterionized tandem membrane system. The system consists of 2 filters: a 200 nm pore poly­(vinylidene fluoride) (PVDF) filter followed by a 30 nm pore cellulose triacetate (CTA) filter. The zwitterionized hydrogel, sulfobetaine methacrylate, is applied to the CTA filter to mitigate protein fouling and enhance exosome capture, allowing for selective exosome enrichment and improved purity in downstream applications. Copyright 2024, American Chemical Society. Available under a CC-BY 4.0.^558^ (D) Catch and display for liquid biopsy (CAD-LB) for capturing and analyzing SiEVs. Fluorescent labeling of EVs is performed by using carboxyfluorescein succinimidyl ester (CFSE), along with antibody targeting for specific biomarkers. The labeled EVs are introduced into a microfluidic device equipped with an ultrathin nanoporous silicon nitride (NPN) membrane. During filtration, unreacted antibodies and contaminants are removed. Captured EVs are subsequently visualized via fluorescence microscopy, enabling the colocalization of CFSE and antibody signals for precise biomarker detection. Copyright 2024, Wiley-VCH GmbH. Reprinted with permission from ref 559.

Membrane-based techniques for EV isolation offer significant advantages over BuEV analysis methods by providing improved selectivity, purity, efficiency, and scalability. These advancements facilitate SiEV analysis, allowing for precise characterization and potential biomarker discovery in clinical settings.

## 6 Recent Advances in EV Characterization and Quantitative Techniques

Understanding the roles of BuEVs and SiEVs in disease pathogenesis is crucial for developing effective diagnostic approaches. As interest in EV research has continued to grow, researchers have faced significant challenges that have driven the development and application of a variety of innovative techniques for measuring and analyzing these vesicles. However, no single method has yet demonstrated the capability to fully characterize the diverse properties of EVs, as well as size distribution and quantity, across a wide range of biological and clinical samples.

The inherent heterogeneity of EVs, characterized by a wide array of biochemical and physical properties, complicates the interpretation of results derived from bulk analysis alone. Recent studies emphasize the necessity of employing multiple methods for analyzing EVs in clinical samples. For example, TEM is frequently utilized to confirm the structural morphology of EVs, while NTA quantifies the number and size distribution of EVs within a given sample.^561,562^ Additionally, immunoblotting is employed to verify EV origin, and Western blotting is used to detect specific proteins associated with EVs in purified samples. However, even when used in combination, traditional bulk analysis techniques have limitations in differentiating between EV subpopulations or in providing detailed information about their cargo.^537^

To address these limitations, recent advances in analytical techniques have incorporated nanomaterials, micro- or nanofluidic devices, and artificial intelligence (AI) and machine learning (ML) into conventional methods.^248^ This integration marks a significant shift toward SiEV analysis, significantly enhancing the sensitivity and specificity of EV detection and characterization.^535,543^ These innovations facilitate the precise identification of EVs, even in complex biological samples, allowing for the differentiation between BuEV and SiEV populations, a critical step in understanding the functional heterogeneity of EVs and their roles in various pathological conditions.

Moreover, advanced analytical methods now enable the comprehensive characterization of EV cargo, including proteins, nucleic acids, lipids, and metabolites. This detailed cargo analysis aids in defining distinct EV subpopulations and linking them to various diseases, thus deepening our understanding of disease mechanisms. The capability of analyzing EVs from bodily fluids also opens up avenues for noninvasive or minimally invasive diagnostics via liquid biopsy, representing a considerable shift in disease detection, monitoring, and personalized treatment strategies. Although SiEV analysis requires smaller sample volumes, often measured in microliters or nanograms, it also necessitates higher sample quality because of the sensitivity of EVs to handling and processing.^314^

In this section of the review, we will present recent advances in both BuEV and SiEV analysis techniques, organized by their underlying principles. This categorization will provide an overview of the advanced approaches available for characterizing and analyzing BuEVs and SiEVs in clinical diagnostics. The implications of these advancements for personalized medicine are significant, as these advancements offer enhanced sensitivity, greater specificity, and improved patient care. The technological innovations that have improved the characterization and analysis of EVs, from BuEV to SiEV analysis, represent a pivotal evolution with profound implications for clinical applications.

### 6.1 Electron Microscopy

Electron microscopy is a powerful imaging technique that employs electrons, not photons, to achieve high-resolution imaging down to the nanometer scale. SEM and TEM, the main types of electron microscopy, are prominent methods utilized for BuEV and SiEV analysis, offering detailed insights into EV structure and morphology. SEM provides detailed surface topography of EVs, making it useful for assessing the size, shape, and surface morphology of large EV populations. TEM, on the other hand, offers a more detailed view of internal EV structures, such as their membrane and cargo organization, and is commonly used for SiEV analysis because of its higher resolution. SEM requires that biological samples be coated with a conductive material, such as gold, to prevent charging and achieve optimal imaging quality. In contrast, TEM does not require sample conductivity, and ultrathin conductive material coating can be directly observed in transmission mode. While both SEM and TEM are valuable for visualizing EV morphology, they primarily provide structural information. SEM also enables relatively large area analysis, allowing researchers to analyze multiple EVs across broader sample regions, giving a more comprehensive overview of their distribution and diversity.

A more advanced variant of SEM, focused ion beam scanning electron microscopy (FIB-SEM), extends the capabilities of traditional SEM by enabling detailed 3D reconstruction of EVs.^537^ Through a process of milling away layers of material, FIB-SEM allows for visualization not only of surface features but also internal structures, offering richer morphological and structural information. This is particularly valuable for characterizing the complex architectures of EVs, which are often involved in nuanced biological processes. However, FIB-SEM, like SEM, requires that biological samples be coated with a conductive material. While surface imaging and 3D topography are central to these methods, the added value of FIB-SEM lies in its ability to offer precise, layer-by-layer visualization, significantly advancing our understanding of EV structure in both isolated and complex environments.^563^

Several advanced TEM-based techniques have been developed. Cryogenic transmission electron microscopy (Cryo-TEM) rapidly freezes EVs in vitreous ice, preserving their native structure and providing high-resolution images without staining or chemical fixation, thus minimizing artifacts. This represents a significant advantage over traditional TEM, allowing for the high-resolution imaging of EV membranes and lumens, providing detailed information about EV structure, membrane organization, and cargo distribution. This technique has been used to reveal substantial morphological diversity among SiEV.^44,564^ Immunolabeling electron microscopy (immuno-TEM) labels EVs with antibodies conjugated to electron-dense markers like gold nanoparticles, enabling the visualization of specific proteins or markers within EVs and thus indicating the presence and distribution of specific proteins or cargo molecules.^562^ Correlative light and electron microscopy (CLEM) combines TEM’s high-resolution imaging with fluorescence microscopy’s specific labeling, enabling the identification and localization of fluorescently labeled EVs within complex biological samples, thus indicating EV interactions and identifying EV subpopulations on the basis of specific markers.^565^ And electron tomography collects TEM images at different tilt angles and reconstructs them to generate a 3D model of EV structures, providing detailed 3D structural information about SiEVs and facilitating the study of complex EV structures and interactions with other cellular components (Figure 11A).^566^ These advanced TEM techniques provide valuable insights into the structure, composition, and interactions of EVs, enhancing our understanding of their biological roles and guiding the development of diagnostics.

**Figure 11:**
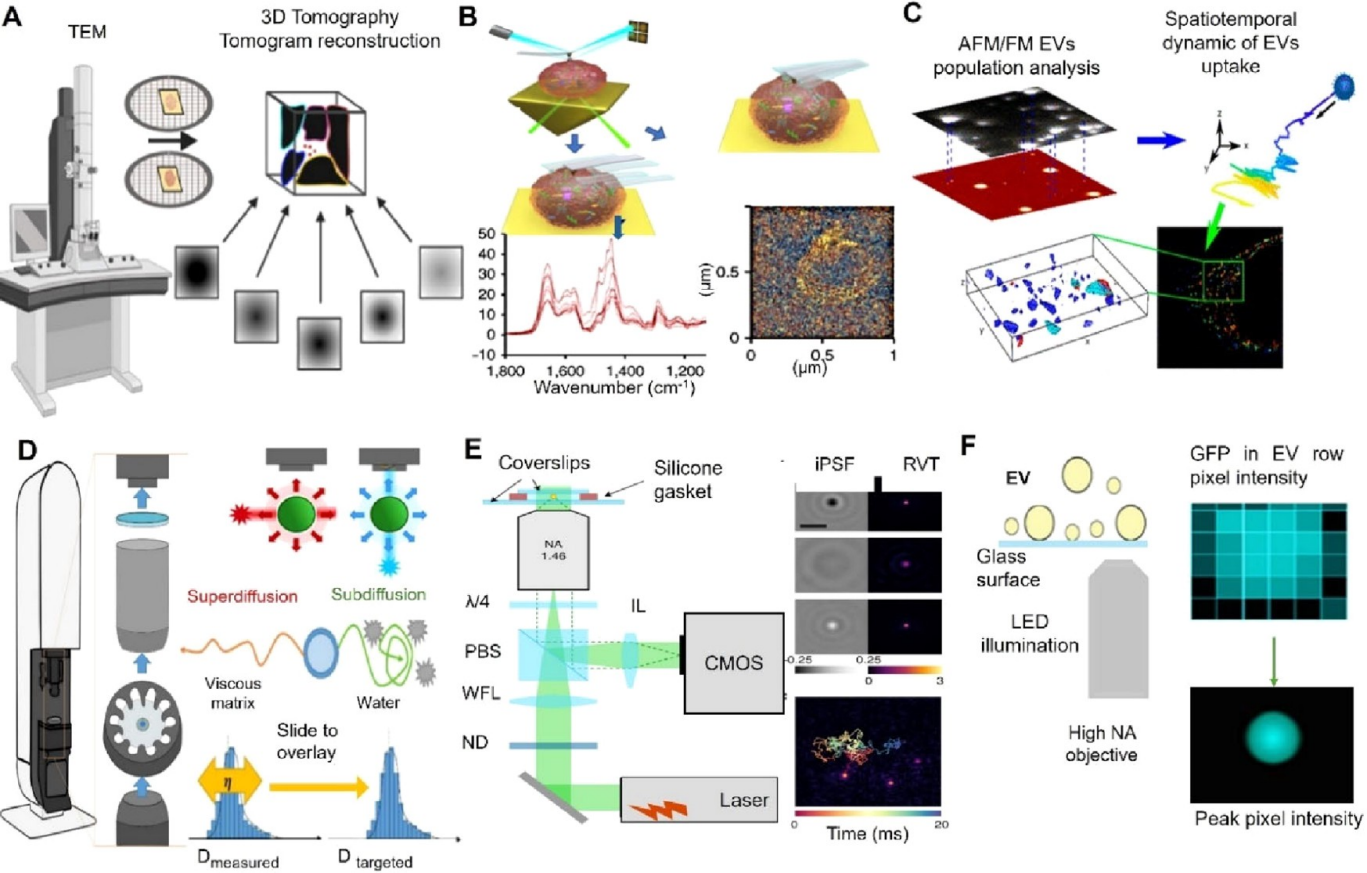
Advanced techniques for BuEV and SiEV characterization. (A) Cryo-TEM and electron tomography. EV samples are applied to a grid and undergo cryo-TEM imaging and processing. Electron tomography is then used for 3D reconstruction. Copyright 2023, John Wiley and Sons. Reprinted with permission from ref 566. (B) AFM-IR spectra and scan mapping image of SiEV in the 1,000 to 1,800 cm^–1^ range, and high-resolution spectra at 1 cm^–1^ resolution. Copyright 2019, Springer Nature. Reproduced with permission from ref 568. (C) Purification analysis, intracellular tracking, and colocalization of EVs using atomic force and 3D single-molecule localization microscopy. Copyright 2023, American Chemical Society. Available under a CC-BY 4.0.^569^ (D) ILM instrument for nanoparticle characterization and the pipeline for analyzing interactions with the environment. Copyright 2024, Wiley-VCH GmbH. Reprinted with permission from ref 573. (E) iNTA utilizes a Wide-field iSCAT setup for tracking freely diffusing particles. Copyright 2022, Springer Nature. Reproduced with permission from ref 576. (F) SMLM determination of the number of green fluorescent protein (GFP) molecules loaded into SiEVs. Copyright 2021, Journal of Extracellular Vesicles published by Wiley Periodicals, LLC on behalf of the International Society for Extracellular Vesicles. Reprinted with permission from ref 76.

### 6.2 Atomic Force Microscopy

AFM is a powerful imaging technique used for characterizing EVs, providing detailed information about their biophysical properties at the nanoscale level. It enables high-resolution imaging and mechanical mapping of entire EVs, allowing for the analysis of both their surface features and internal structure.^493^ AFM provides crucial data on size, surface topography, and mechanical properties, which are essential for studying the heterogeneity of SiEVs. In addition to standard topographic imaging, advanced AFM modes offer deeper insights, such as analyzing the kinetics of EVs at the single-vesicle level, which can reveal how membrane phases and EV origins influence vesicle internalization routes.^567^ AFM can also be combined with other methods. For example, AFM combined with infrared spectroscopy (AFM-IR), a technique that has seen extensive use in various applications, offers simultaneous measurements with enhanced spatial resolution. While its utilization in SiEV studies remains relatively limited, its potential for detailed chemical analysis of EV composition makes it a promising technique for further exploration (Figure 11B).^568^ AFM has also been combined with 2D single-molecule fluorescence microscopy (SMFM) and 3D single-molecule localization microscopy (SMLM) to provide a comprehensive understanding of purified, fluorescently labeled, endosome-derived EVs, particularly in terms of their size and colocalization with a specific protein (Figure 11C).^569^ The researchers employed 2-color 3D SMLM and newly developed software to measure, for the first time, the colocalization of EVs with transferrin, a protein known to be involved in early endosomes and recycling processes within cells.

### 6.3 Fluorescence Microscopy

EVs can be characterized by using fluorescence microscopy techniques, such as total internal reflection (TIRF) microscopy, confocal microscopy, super-resolution microscopy, SMLM, and light-sheet microscopy, which play crucial roles in understanding various EV–cell interactions, including EV release, uptake, and SiEV composition.^118^ TIRF microscopy, in particular, is especially useful for analyzing EV content, as it visualizes molecules within a thin region near the glass coverslip surface where EVs or cells are immobilized. By restricting fluorescence detection to this area, TIRF enhances the signal-to-noise ratio, making it ideal for single-molecule analysis and enabling precise detection of fluorescence signals at the cell membrane surface.^570^ This capability allows detailed insights into the molecular composition of SiEVs. For instance, studies using TIRF and SiEVs with GFP-tagged organelle markers have shown that CD9 and CD81 are primarily associated with the plasma membrane, while CD63 is linked to endosomal organelles.^571^ TIRF microscopy is also often employed in SMLM to provide quantitative and high-resolution details at the SiEV level, including the shape and morphology of SiEVs, which help to elucidate relationships between morphology and function.^570^ Combining TIRF microscopy with microfluidic resistive pulse sensing (MRPS) offers additional avenues for SiEV analysis, enabling precise sizing, quantification, and insights into membrane dynamics and interactions.^572^ One notable assay employing TIRF microscopy for image recording and analysis is the immune lipoplex nanoparticle (ILN) biochip assay. This assay is designed to capture specific subpopulations of EVs and detect GPC1 mRNA and its corresponding protein at the SiEV level. The assay established optical cutoff values for GPC1 mRNA (threshold fluorescence intensity [TFI] = 325,873) and tetraspanin membrane vesicle (tMV) protein (TFI = 32,495), enabling differentiation of PDAC patients from controls with 100% specificity, based on ROC curves. These cutoff values correspond to 6.5 × 10^8^ and 2.0 × 10^8^ PANC1-derived EVs/mL in healthy donor serum, ensuring reproducibility across laboratories. Clinical evaluations demonstrated the assay’s translational potential for early PDAC screening and treatment prognosis. It achieves high AUC values of 0.947 for stage I PDAC and 0.973 for stage III/IV. Additionally, the performance of the EV GPC1 biomarker was compared to CA19-9, the established clinical standard for PDAC diagnosis and prognosis.^118^ To optimize the detection and analysis of EVs within the ILN biochip assay, the researchers employed algorithms for EV detection that generally consider EVs as diffraction-limited objects and approximate their shape to a Gaussian function in confocal, TIRF, and light-sheet microscopy.^466^ In addition, the use of interferometric light microscopy (ILM) enhances this analysis by measuring nanoscale viscosity, providing insights into local viscosity at the vesicle surface and its effects on EV size and concentration (Figure 11D).^573^ This combination of techniques underscores the assay’s ability to accurately identify and characterize SiEVs, further supporting its application in PDAC diagnostics.

### 6.4 Nanoparticle Tracking Analysis

Dynamic light scattering (DLS) and nanoparticle tracking analysis (NTA) are vital techniques for understanding the physical properties of BuEVs and SiEVs in suspension.^574^ DLS is often used for particle sizing, providing a straightforward and accurate assessment of the hydrodynamic size distribution of EVs, crucial for evaluating their stability and aggregation. But DLS has limitations in resolution, especially for heterogeneous samples and aggregates, leading to potential overestimation of EV size, and the technique provides limited insights into morphology and cargo content.^536^ In contrast, NTA offers high resolution and the capability to detect individual EVs, enabling real-time analysis and movement tracking. NTA’s reliance on Brownian motion analysis allows for real-time quantification of particle concentration and determination of hydrodynamic radius, overcoming challenges associated with analysis following common EV isolation methods, where particles can be obscured by similarly sized lipoproteins. NTA directly visualizes SiEVs, effectively overcoming challenges associated with polydispersity and the presence of similarly sized lipoproteins that can complicate traditional EV isolation methods. This capability also facilitates surface protein phenotyping of EVs. While NTA is versatile and effective for a range of EV types, it does have limitations. It requires careful sample preparation and may struggle with detecting very small or very large EVs, leading to incomplete analysis for certain size ranges.^561^ Despite this, NTA is widely used for quantifying EV populations and characterizing their size distribution, which is critical for understanding the heterogeneity of EV samples.

Advanced techniques can couple orthogonal detection strategies with NTA. For example, NTA coupled with fluorescence detection (NTA-FD) can enable simultaneous visualization and quantification of fluorescently labeled SiEVs, while single particle tracking analysis (SPTA) offers high-throughput analysis and detailed morphological information about EV populations.^575^ Moreover, interferometric nanoparticle tracking analysis (iNTA) is a new method that employs interferometric detection of scattering to analyze the trajectories and scattering cross sections of diffusing single nanoparticles. Interferometric detection of scattering (iSCAT), to reach unprecedented sensitivity and precision in determining the size and refractive index distributions of nanoparticles in suspensions. iNTA represents a recent breakthrough in particle analysis, offering precise determination of size and refractive index with exceptional sensitivity. Unlike conventional NTA, iNTA has improved capability to differentiate between EVs and lipoproteins, enabling precise quantification of EVs. Thus, iNTA holds significant promise as a novel standard for label-free characterization of EVs in suspension (Figure 11E).^576^ These advanced techniques enhance the capabilities of NTA, promising significant advancements in studying EVs across various scientific disciplines, including nanomedicine and biotechnology.

### 6.5 Flow Cytometry

Flow cytometry is a commonly used technique for examining and separating cells (i.e., fluorescence or flow-assisted cell sorting [FACS]) on the basis of their physiological and chemical traits. In conventional flow cytometry, cells are labeled with fluorescent markers and then passed through a laser beam. A detector captures scattered and emitted light, providing information on cell size, granularity, and surface markers.^577^ Although helpful for analyzing cells, using flow cytometry to analyze EVs comes with distinct challenges because of their tiny size—most conventional flow cytometers cannot accurately detect particles smaller than 500 nm in diameter, as well as their diversity and relatively low protein content.^578^

To overcome these challenges, researchers have developed advanced techniques. Recently, it was shown that a laboratory-built nanoscale flow cytometer could detect SiEVs as small as 40 nm in diameter as well as single DNA fragments of 200 bp upon SYTO 16 staining, used to study EV DNA at the SiEV level.^579^ Another technique, high-resolution flow cytometry (HRFC), combines traditional flow cytometry with high-resolution imaging to enhance EV detection, sensitivity, and resolution. This allows for the analysis of SiEVs and their cargo, including proteins and nucleic acids, and enables the identification of specific subpopulations with diagnostic relevance.^580^ Innovations like the BD influx flow cytometer and HRFC now enable the detection of smaller EVs, down to about 100 nm, thus improving BuEV analysis. Integrated techniques, such as optimized vesicle flow cytometry, enhance sensitivity and resolution for detecting SiEVs, while nanoscale flow cytometry specifically utilizes nanostructures for improved detection.^581^ The integration of nanoflow cytometry and SMLM provides a rapid and reliable method for analyzing SiEVs within a population of engineered EVs. This advanced approach enabled a detailed examination of the distribution of GFP across various SiEV subpopulations, highlighting differences in their engineering (Figure 11F).^76^ SMLM, in particular, is a highly precise technique that allows for the quantification of GFP molecules present in individual SiEVs. Through comparative analyses, we can evaluate the extent of GFP enrichment when SiEVs are fused with different EV-sorting proteins. This capability is crucial, as it helps identify which sorting proteins enhance the targeting efficiency of engineered EVs toward specific recipient cells.

High-sensitivity flow cytometers, such as CytoFLEX by Beckman Coulter, CellStream by Cytek, and Flow NanoAnalyzer by NanoFCM, can detect EVs as small as ∼60 nm by using a “top-down” approach, in which technologies developed for large entities (cells) have been adapted for the study of EVs. But new technologies are achieving even higher sensitivity by using a “bottom-up” approach, in which technologies developed to study single molecules are being adapted for the study of EVs. For example, a recent comparison study of the performance of a single-molecule sensitive flow cytometry (SMFC) platform with CytoFLEX and CellStream in characterizing sEVs from colorectal cancer cells found that CytoFLEX detected only 5.7% of EVs detected by SMFC and CellStream, only 1.5%, and that median EV diameter and protein biomarker copy numbers were much larger for CytoFLEX and CellStream than for SMFC and as measured by single-molecule microscopy.^582^ This comparison demonstrated that SMFC detects many sEVs that are below the limits of detection of the other systems. Thus, calibrating instruments and determining their limits of detection is important so the subpopulation of detected sEVs can be ascertained for improved rigor and reproducibility.

Another microfluidic approach for the molecular profiling of SiEVs was developed through nanoscale exome protein-based sorting using immunomagnetic-activated cytometry (NanoEPIC). The NanoEPIC platform conducts high-throughput and high-resolution sorting of individual SiEVs on the basis of surface marker expression, offering robust profiling of exosome markers and enabling small EV subpopulation analysis.^583^

Furthermore, fluorescence imaging flow cytometry (IFC) combines flow cytometry with microscopy, allowing visualization and analysis of SiEVs.^584^ Recent research developed a method named “EV fingerprinting,” which discerns distinct vesicle populations using dimensional reduction of multiparametric data collected by quantitative SiEV flow cytometry.^82^

These advancements hold promise for clinical diagnostics, providing deeper insights into EV heterogeneity and cargo for elucidating pathological mechanisms and managing diseases. Nonetheless, challenges in standardization and accessibility persist. Ongoing developments in innovative flow cytometry platforms, including microfluidics-based systems, offer potential solutions to these issues, potentially contributing to EV analysis for clinical diagnostics.

### 6.6 Resistive Pulse Sensing

Resistive pulse sensing (RPS)-based techniques offer robust methods for both BuEVs and SiEVs. In bulk analysis, RPS accurately measures EV size, concentration, and surface charge by detecting changes in electrical resistance as EVs traverse a nanopore.^585^ This technique, also known as tunable resistive pulse sensing, is particularly advantageous for analyzing polydispersed samples, as it allows for the adjustment of nanopore size to enhance sensitivity across a broad range of particle sizes.^586^ RPS finds applications in diverse areas, ranging from the development of EV isolation protocols for miRNA sequencing to the investigation of disease-specific EVs.^587^ In SiEV analysis, RPS provides a detailed examination of individual EVs, uncovering the heterogeneity within EV populations and enabling dynamic studies of EV responses to stimuli or treatments.^588^ A challenge with RPS is its restricted particle concentration detection limit. For example, a typical concentration limit of detection is ∼10^8^ particles/mL for many commercial RPS machines, due to the limited sampling efficiency of the platform. This challenge was addressed by an extended Nano-Coulter Counter, which comprised 5 pores in parallel that increased the sampling efficiency. In addition, the pores were placed within a microfluidic chip, and with the proper fluidic network, which also increased the sampling efficiency. As a result, the team reported a concentration limit of detection of ∼1000 particles/mL.^589^

Variations of RPS have been developed, such as microfluidic resistive pulse sensing, which measures the particle size distribution of EVs. However, for this technique, specific guidelines for measuring EVs together with other biofluid particles are lacking, affecting reproducibility, and to address this issue, a procedure has been developed to reproducibly measure the particle size distribution.^494^ Ongoing advancements in RPS instrumentation and data analysis algorithms promise to further enhance our understanding of the biology of EVs and their clinical relevance.

### 6.7 Mass Spectrometry

Techniques based on mass spectrometry (MS) have become essential tools in the fields of genomics, metabolomics, and proteomics.^590^ In metabolomics, MS provides intricate insights into small molecules (metabolites) present in biological samples.^591^ Liquid chromatography–mass spectrometry (LC-MS) and gas chromatography–mass spectrometry (GC-MS) are commonly used for metabolite profiling. LC-MS is particularly powerful because of its capability to separate and identify complex mixtures of metabolites, facilitating our precise understanding of metabolic pathways and disease mechanisms, leading to biomarker discovery.^592^ In proteomics, MS allows comprehensive analysis of proteins and their modifications, crucial for our understanding of cellular functions and disease processes. Shotgun proteomics, which involves digesting proteins into peptides before MS analysis, provides insights into protein expression, post-translational modifications, and protein interactions.^593^ Targeted proteomics, such as selected reaction monitoring (SRM) and parallel reaction monitoring (PRM), enables precise quantification of specific proteins or post-translational modifications, thus aiding in the discovery of biomarkers and their validation in clinical samples.^594^

Mass spectrometry (MS)-based techniques have been instrumental in characterizing EVs by enabling the precise identification and quantification of EV-associated proteins. Through proteomic analysis, MS has revealed dynamic changes in EV protein expression under various physiological and pathological conditions, aiding in the discovery of new biomarkers.^595,596^ For example, LC-MS was used to analyze serum EVs from patients with biliary tract infections and revealed potential protein biomarkers for this condition, CEACAM1 and CRB3, holding promise for clinical diagnosis.^597^ As another example, ultrafast isolation and MALDI-TOF MS were used for the proteomic analysis of BuEVs from plasma, a technique known as robust acute pancreatitis identification and diagnosis (RAPIDx). This technique involves the proteomic fingerprinting of intact nanoscale EVs from clinical samples. Through quantitative analysis of EV fingerprints using MALDI-TOF MS, specific proteins such as SAA proteins (SAA1-1, desR-SAA1-2, SAA2, SAA1-2) were identified with AUCs ranging from 0.92 to 0.97, enabling the detection of acute pancreatitis within 30 min. Moreover, the combination of SAA1-1 and SAA2, with two specific protein peaks (5290.19, 14032.33 *m*/*z*), achieved an AUC of 0.83 for classifying the severity of the condition. This platform holds promise for facilitating the timely diagnosis and treatment of acute pancreatitis, preventing the development of severe acute pancreatitis and persistent organ failure, and contributing to precision diagnostics and the early detection of pancreatic cancer.^598^

LC coupled with electrospray ionization tandem mass spectrometry (ESI–MS/MS) is a preferred technique for analyzing the molecular content of EVs. Specifically, nano-ESI–MS/MS offers high sensitivity and resolution, allowing for the detection, identification, and quantification of thousands of proteins within a homogeneous EV population. For instance, the content of sEVs from different glioblastoma stem-like cell subtypes has been investigated to identify molecules involved in their plasticity. Profiling the protein, metabolite, and fatty acid content revealed significant differences in biomolecule abundance between proneural and mesenchymal sEVs, suggesting that these distinct subtypes cooperate via sEVs, contributing to the aggressive characteristics of glioblastoma.^599^

The integration of MS with other techniques has further enhanced diagnostic performance. Other advanced MS-based techniques, such as targeted sequential window acquisition of all theoretical fragment ion spectra mass spectrometry (SWATH-MS) proteomic workflows, have shown promise in identifying and quantifying plasma-EV proteins. These advanced MS-based techniques enable rapid and sensitive analysis of both BuEVs and SiEVs, promising to revolutionize clinical diagnosis by providing insights into disease mechanisms and facilitating the development of personalized medicine approaches.^600^ Moreover, the integration of MS with other techniques such as tandem surface-enhanced Raman spectroscopy (SERS), when applied to MS profiling of plasma-derived EV, has shown significant promise in detecting early ovarian cancer biomarkers, demonstrating high diagnostic accuracy. This underscores the effectiveness of combining these techniques for the identification of key biomarkers.^601^

Integrating nanomaterial-based enrichment with advanced mass spectrometry enhances the precision and depth of EV proteome analysis. For example, combining the TiO_2_ nanomaterial enrichment method for EV isolation with high-throughput MS facilitates efficient characterization and proteome profiling of EVs from complex and viscous biofluids. In this example, the high specificity of the interaction between EVs and TiO_2_ beads allows for effective isolation of EVs from plasma, separating them from abundant plasma proteins and lipoproteins. This targeted removal facilitates the identification of EV proteins through LC-MS/MS, improving the accuracy of proteomic analysis.^602^ Another technique is multiplexed gold nanoshell and porous silicon nanowires-assisted laser desorption/ionization (LDI), known as MNALCI, which shows high sensitivity and specificity in distinguishing various cancers from healthy controls using minimum serum samples.^603^ Furthermore, MS can also be integrated with nanoprojectile secondary ion mass spectrometry (NP-SIMS), which assesses the protein content of EVs using tagged antibodies. NP-SIMS determines the relative abundance of EV-associated proteins by bombarding a surface with individual gold nanoprojectiles. Each impact transfers material from the sample to a mass spectrometer, generating MS signals that enable the identification and quantification of specific biomolecules. Despite challenges associated with this technique, such as the need for specialized algorithms and software for data analysis, NP-SIMS has great potential for biomarker discovery in EVs.^604,605^

Recent advancements in elemental and molecular mass spectrometry (MS) techniques have significantly enhanced our ability to analyze biomolecules at the SiEV level. Techniques such as inductively coupled plasma mass spectrometry (ICP-MS), especially when combined with metal-labeled probes, facilitate multiplexed analysis of EVs, providing valuable insights into their heterogeneity, biogenesis, composition, and function. While ICP-MS shows considerable promise in analyzing SiEVs and single cells, challenges related to sensitivity and resolution persist at these smaller scales. Despite these limitations, these techniques offer substantial potential for advancing our understanding of EVs in biological processes.^606^ Furthermore, integrated techniques, such as nanoscale secondary ion mass spectrometry (NanoSIMS) combined with transmission electron microscopy (TEM), have enabled in-depth investigations into the role of vesicle size in partial release events. In a study involving PC12 cells containing dopamine-loaded vesicles via the vesicular monoamine transporter 2 (VMAT2), NanoSIMS facilitated the absolute quantification of dopamine (DA) at the single-vesicle level. The study revealed that partial release was independent of vesicle size, suggesting that this release mode is a characteristic feature of all dense-core vesicles.^607^ Analyzing SiEVs remains challenging due to their low molecular content and the potential for contamination from nonspecific binding of environmental proteins. To address these issues, specialized algorithms and software have been developed to enhance data analysis of SiEVs, improving the accuracy and reliability of results.^237^

### 6.8 Raman Spectroscopy

Raman spectroscopy (RS) is a label-free technique that provides a powerful means of analysis. In RS, laser light interacts with a sample, causing molecular vibrations, which result in inelastic scattering of the light. This scattered light is then detected to analyze the sample.^608^ Innovative techniques derived from RS have been developed for BuEV and SiEV analysis, tailored for both lEVs and sEVs.^609,610^

One such technique is surface-enhanced Raman spectroscopy (SERS), which utilizes localized surface plasmon resonance (LSPR). In a novel nanocavity-based system, the integration of surface-enhanced SERS barcodes with mirror-like, asymmetric gold microelectrodes enables precise sEV analysis. By applying an alternating current across the gold microelectrodes, nanofluidic shear forces are generated, promoting the binding of sEVs and facilitating the efficient assembly of nanoboxes. The binding forms a nanocavity between the nanobox and the gold microelectrode, which significantly amplifies the local electromagnetic field, thereby enhancing the Raman signals from four distinct SERS barcodes. These amplified signals generate patient-specific molecular sEV signatures. This system simultaneously profiled 4 protein markers (CD63, MUC1, EGFR, and TNC) on the sEV surface. When tested on a cohort of clinical samples (*n* = 76) representing various stages of lung cancer, the patient-specific sEV molecular signatures enabled accurate identification, stratification, and treatment monitoring of lung cancer patients, underscoring the system’s potential for clinical application (Figure 12A).^611^ Another system derived from RS is single particle automated Raman trapping analysis (SPARTA), an automated system optimized for SiEV analysis, representing a versatile approach for studying EVs as a source of cancer biomarkers. This technique offers molecular characterization of SiEVs by capturing their Raman spectra, providing detailed insights into their chemical composition (Figure 12B).^610^ Another innovative system is tip-enhanced Raman spectroscopy (TERS), a combination of RS and AFM. This system offers enhanced spatial resolution and sensitivity, providing detailed local information on nanometer-sized EVs.^609^

**Figure 12:**
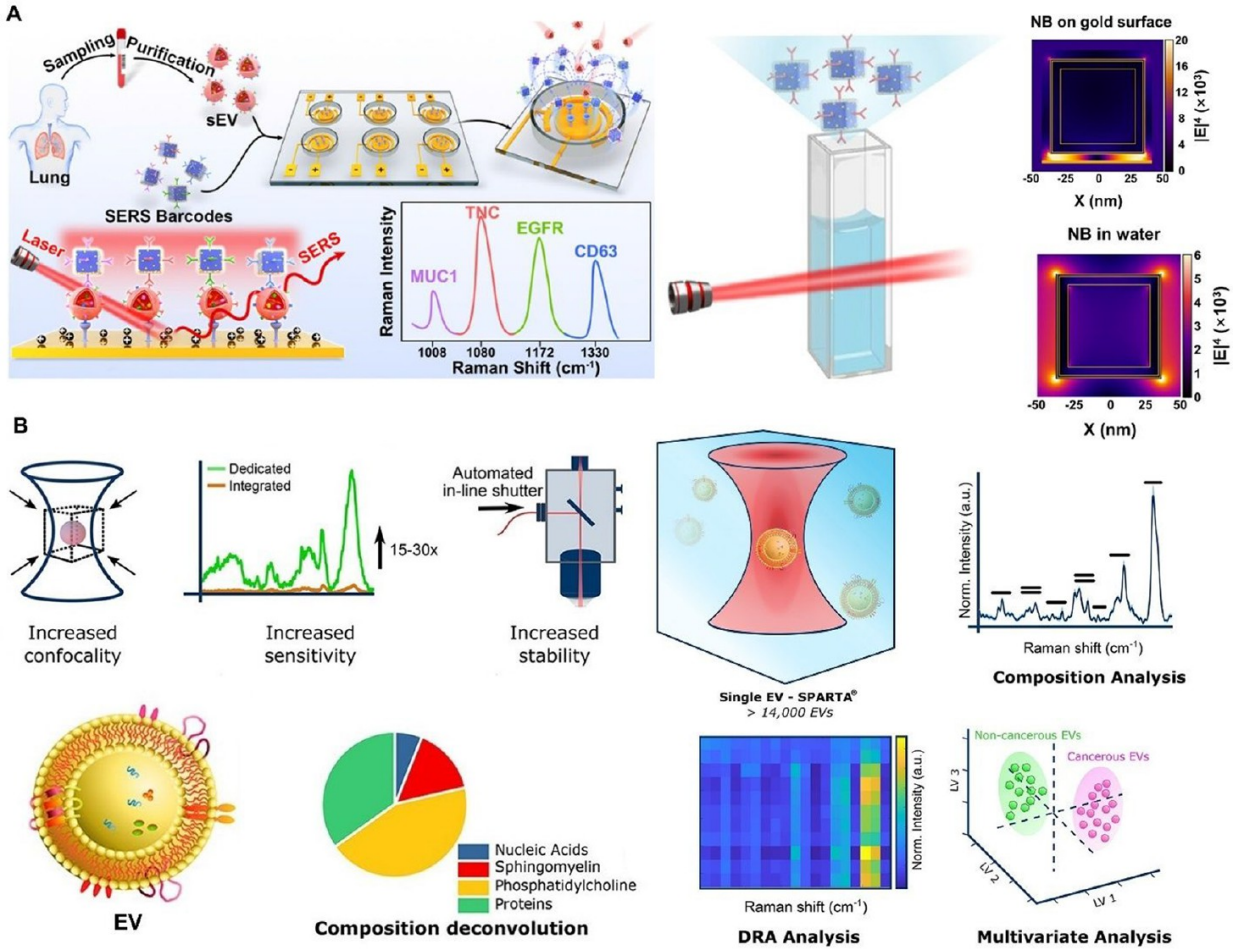
Advanced Raman techniques for BuEV to SiEV analysis. (A) Surface-enhanced Raman scattering (SERS) barcode-based gold microelectrode for BuEV detection. BuEVs purified from human blood are captured and identified using nanobox-based SERS barcodes under alternating current. Upon laser excitation, BuEVs bridge the gold microelectrode and the SERS barcode, forming a nanocavity that detects Raman signals corresponding to specific protein expression levels. Copyright 2024, American Chemical Society. Reprinted with permission from ref 611. (B) Single-particle automated Raman trapping analysis (SPARTA) platform. SPARTA uses surface plasmon resonance microscopy (SPRM) for automated SiEV analysis, generating detailed compositional Raman spectra for over 14,000 individual SiEVs. Copyright 2021, American Chemical Society. Available under a CC-BY 4.0.^610^

RS can also be integrated with nanoplasmonic techniques, which amplify the Raman signals of molecules near metal nanostructures, enabling highly sensitive detection of EV molecules. These systems, which can be used in conjunction with nanomaterials and machine learning algorithms to enhance the accuracy, sensitivity, and selectivity of EV analysis, are proving to be important for clinical diagnosis.^612^ In one example, exosomes from prostate cancer cells were detected in undiluted patient serum or microvesicles in whole blood at the single-particle level by optical microfibers modified with nanomaterials.^612^ Specifically, the microfibers were modified with the nanomaterial tungsten disulfide (WS_2_)–supported gold nanobipyramid (Au NBPs) nanointerfaces and EpCAM aptamers to detect exosomes. Leveraging near-field LSPR, this integrated technology achieved ultrasensitive detection of exosomes, with a limit of detection value of 23.5 particles/mL in pure PBS and 570.6 particles/mL in 10% serum, respectively.

In another example, SERS was combined with machine learning to analyze EVs for diagnosing and staging thyroid cancer. This integrated technology profiled exosomes in clinical blood samples by using SERS on substrates functionalized with MXene-coated gold@silver (core@shell) nanoparticles and combined this with deep learning. This technology achieved a dynamic range of 0.5 × 10^10^ to 2.0 × 10^11^ EVs/mL with a limit of detection of 1.7 × 10^9^ EVs/mL. Impressively, it demonstrated a diagnostic accuracy of 96.0% in differentiating thyroid cancer patients from healthy controls and an accuracy of 86.6% in staging the cancer patients.^613^ These advanced techniques enable rapid, sensitive, and label-free analysis of both BuEVs and SiEVs, offering great promise for precision diagnostics and personalized medicine.

### 6.9 Nanoplasmonic Techniques

Nanoplasmonic techniques leverage plasmonic nanostructures, primarily composed of noble metals like gold and silver, to enhance electromagnetic fields at the nanoscale through surface plasmon resonance (SPR) and LSPR. These techniques significantly improve the sensitivity of biosensing for biomolecule detection, particularly through SERS, which allows for the identification of specific biomolecules at low concentrations. In addition, they enhance fluorescence signals, improving imaging resolution in biological samples.^614,615^ The localized electromagnetic “hotspots” generated by these nanostructures enable the detection of EVs, making nanoplasmonic techniques highly valuable for advancing diagnostic applications in clinical settings.^8,182,616^

LSPR arises in nanoscale metallic structures, such as gold or silver nanoparticles, where localized oscillations of conduction electrons are confined within the nanostructure. LSPR-based biosensors offer high sensitivity and can be implemented in simplified optical configurations, including dark-field and colorimetric detection.^617^ To enhance LSPR biosensing capabilities, various strategies have been explored, often requiring trade-offs between signal amplification, target specificity, and assay complexity. To address these challenges, a rapid copper nanoshell-enhanced immunoassay (Cu-NEI) was developed, leveraging in situ copper growth for efficient and cost-effective signal amplification. This method exploits the superior plasmonic properties of copper nanoshells while maintaining assay simplicity (Figure 13A).^616^ In the Cu-NEI assay, Cu nanoshells are grown on antibody-conjugated gold nanoparticles (AuNPs) that specifically bind to LAM biomarkers on EVs, facilitating the detection of -derived LAM for TB diagnostics. The Cu nanoshells preferentially form cubic and tetrahedral morphologies on monocrystalline AuR probes, significantly enhancing the scattering signal. This streamlined approach is compatible with high-throughput multiwell formats and maintains a high signal-to-noise ratio, crucial for sensitive detection. Furthermore, the assay integrates seamlessly with dark-field microscopy, supporting its potential for clinical translation. Validation in a TB-screened cohort demonstrated robust diagnostic performance, achieving a sensitivity of 76.19%, specificity of 100%, and overall accuracy of 83.81%, with an AUC of 0.92. These findings highlight the Cu-NEI assay as a promising platform for EV-based TB diagnostics.

**Figure 13:**
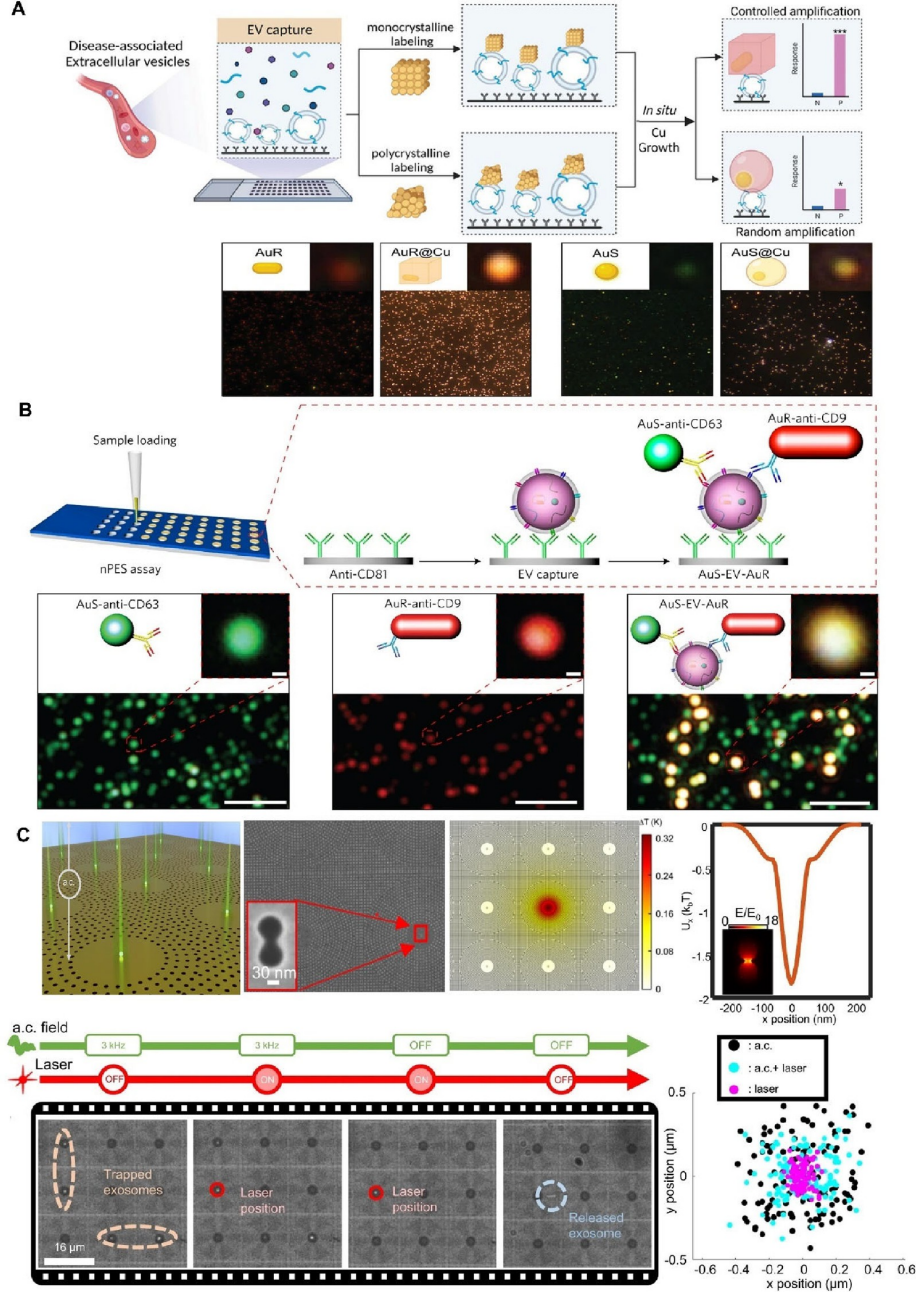
Advanced nanoplasmonic techniques for BuEV and SiEV analysis. (A) Copper nanoshell–enhanced immunoassay (Cu-NEI). Copper nanoshells are grown in situ on antibody-conjugated gold nanoparticles that specifically bind to LAM biomarkers on EVs for the detection of –derived LAM for TB diagnostics. Copyright 2022, Wiley-VCH GmbH. Reprinted with permission from ref 616. (B) Nanoplasmon-Enhanced Scattering (nPES) assay for EV Detection. Dark-field microscope (DFM) images of AuS-anti-CD63 (green), AuR-anti-CD9 (red) and AuS-EV-AuR complexes, which are detectable as bright yellow dots. Scale bars: main images, 2 μm; magnified images, 100 nm. Copyright 2017, Springer Nature. Reproduced with permission from ref 618. (C) Geometry-induced electrohydrodynamic tweezers (GET) for SiEV trapping. The SEM image shows the plasmonic double nanohole aperture antenna at the core of the trap, with the inset providing a closer view. Temperature distribution analysis at the surface of the plasmonic antenna on a sapphire substrate confirms that the trapping intensity remains controlled to avoid overheating. Frame-by-frame sequence SiEV are dynamically trapped and released by superposition electrohydrodynamic forces with plasmon-enhanced optical trapping potential upon laser illumination, SiEV is seamlessly manipulated and transferred from one trap to the next. Copyright 2023, Springer Nature. Reproduced with permission from ref 622.

An advanced nanoplasmonic technique, the nanoplasmon-enhanced scattering (nPES) assay addresses the limitations of conventional EV assays, such as large sample requirements, lengthy processing times, and high costs. By leveraging antibody-conjugated gold nanospheres and nanorods, nPES enables rapid, ultrasensitive, and cost-effective detection of tEVs from just 1 μL of plasma. These nanoparticles selectively bind to captured EVs on a sensor chip, generating a localized plasmonic effect that enhances detection sensitivity and specificity (Figure 13B).^618^ A major application of nPES is the detection of EphA2-positive EVs, a biomarker linked to pancreatic cancer. The EphA2-EV nPES assay demonstrated high diagnostic accuracy, effectively distinguishing pancreatic cancer from pancreatitis and healthy controls. Furthermore, EphA2-EV levels correlated with tumor progression and early treatment responses, outperforming conventional ELISA. A pilot study involving patients with pancreatic cancer (stages I–III), pancreatitis, and healthy individuals validated its diagnostic performance, showing that changes in EphA2-EV levels reflected treatment responses. With its high-throughput, rapid, and low-cost capabilities, the nPES assay represents a significant advancement in EV-based diagnostics, offering potential applications in early disease detection and real-time therapeutic monitoring across diverse clinical settings.^618^

SPR has emerged as a label-free technique for analyzing EVs, ranging from BuEVs to SiEVs. It detects changes in the refractive index of a plasmonic surface due to molecular interactions, providing a platform for biomarker detection.^619^ To enhance the sensitivity of SPR assays, nanomaterials with unique optical properties have been incorporated into SPR platforms, significantly improving signal amplification and detection sensitivity.^620^ Recent advancements in SPR techniques have transformed the characterization of EVs from BuEV to SiEV levels. These advances offer enhanced sensitivity, specificity, and multiplexing capabilities, enabling the detection of EV biomarkers in complex biological samples and improving clinical diagnosis.^621^ However, these SPR approaches often rely on large spectrometers, limiting portability. To address this, alternative techniques such as the digital SiEV analyzer (DEA) have been introduced. DEA automates SiEV analysis within the SPR platform, allowing for subpopulation identification, counting, and sizing. Despite these advances, challenges such as low contrast and signal-to-noise ratios persist in SiEV detection. Novel SPR sensor designs are tackling these challenges by improving the detection of low-abundance biomarkers.^52^

Beyond these SPR innovations, a new method called the geometry-induced electrohydrodynamic tweezers (GET) platform has been developed (Figure 13C), which rapidly traps SiEVs from the surrounding solution at designated locations within seconds.^622^ The GET platform comprises a finite array of plasmonic nanoholes arranged in a circular geometry with an inner void region. This design generates multiple electrohydrodynamic potentials and integrates nanoscale plasmonic cavities at the center of each trap. Positioning SiEVs near plasmonic cavities enables instant plasmon-enhanced optical trapping upon laser illumination without causing heating damage. This approach enhances trapping efficiency even in low-particle-concentration media, significantly boosting analysis throughput. Thus, these noninvasive, scalable hybrid nanotweezers mark a significant advancement in high-throughput, tether-free plasmon-enhanced SiEV trapping and spectroscopy, holding great potential for advancing diagnostic techniques based on SiEVs.

A novel concentric gradient nanoplasmonic sensor offers label-free, sensitive, and quantitative analysis of tumor-derived BuEVs. This sensor, comprising a wafer-scale metasurface with gradient metal nanostructures, demonstrated excellent sensitivity in detecting EVs from the plasma of cancer patients, with a high sensing performance of 9.23 × 10^–5^ refractive index units (RIU), achieving real-time measurements of BuEV binding at concentrations as low as 143 femtomolar.^623^ Moreover, a compact imaging-based sensing device was designed, combining large-area and real-time imaging with spectroscopic approaches, promising for point-of-care diagnostics.^624^

Despite these advancements, limitations remain in systems like nanoplasmonic exosome (nPLEX), which struggle with bulk analysis sensitivity and lack multiplexing capabilities. Innovations like the fluorescence-amplified extracellular vesicle sensing technology (FLEX) and the next-generation enhanced fluorescence detection (nPLEX-FL) platform address these issues by improving fluorescence signal amplification and enabling multiplexed analysis of SiEV.

In the FLEX assay, EVs are captured on a plasmonic gold nanowell surface and immunolabeled for specific biomarkers, enabling protein profiling of EVs at the SiEV level.^110^ Using this assay, researchers have identified biomarkers of cholangiocarcinoma, such as MUC1, EGFR, and EPCAM, and have used these biomarkers to detect tEVs in clinical samples. This innovation detected cholangiocarcinoma with an AUC of 0.93, suggesting its potential as a reliable liquid biopsy test for early screening and detection of this type of cancer.

In the nPLEX-FL platform, using periodic gold nanohole structures, amplifies fluorescence signals associated with EVs. This enhancement allows for sensitive and multiplexed analysis of SiEVs, improving the detection and profiling of various biomarkers.^625^ These advancements in high-throughput SPR platforms promise to improve clinical diagnostics by providing rapid, accurate, and personalized tools for disease detection and molecular monitoring, while also enabling real-time monitoring of EV interactions with capture molecules, significantly aiding in biomarker discovery.

### 6.10 Digital Techniques

Digital techniques encompass analytical methods that utilize digital quantification to enable precise detection and measurement of biomolecules. By converting biological signals into discrete, countable units, these techniques facilitate highly sensitive and accurate molecular-level assessments.^626^ These approaches can also be integrated with digitized sensors with advanced AI algorithms, offering powerful platforms for the simultaneous detection of multiple targets with exceptional sensitivity and precision.^627^

In the context of EV analysis, digital techniques offer significant advantages, particularly in overcoming the limitations of BuEV analysis. Diagnosing diseases using BuEV presents challenges due to the low expression levels of biomarkers and the complex physical and biological properties of EV samples. Thus, recent advancements in digital-based methods have shifted the focus from BuEVs to SiEV analysis, enabling the examination of individual vesicles in clinical samples and their potential use as diagnostic tools.^242, 460^ For example, a highly sensitive double digital assay has been developed for the absolute quantification of individual molecules in SiEVs.^115^ Another example is digital polymerase chain reaction (dPCR), a highly sensitive method that allows for the absolute quantification of nucleic acids. Unlike conventional PCR, which provides relative quantification, dPCR partitions a sample into thousands of individual reactions, detecting and counting DNA or RNA molecules in each partition. This method enhances precision and sensitivity, particularly in cases of low biomarker expression, making it a valuable tool for analyzing SiEVs. Its application in disease diagnosis has demonstrated the potential to identify rare genetic mutations and other molecular alterations with greater accuracy.^242^ Moreover, the digital enzyme-linked immunosorbent assay (d-ELISA) builds upon the traditional ELISA method by providing digital quantification of protein biomarkers. By isolating and detecting single molecules, d-ELISA increases the detection sensitivity, which is crucial when analyzing complex and heterogeneous EV populations. This technique has shown promise in accurately measuring low-abundance proteins, offering an improvement over BuEV analysis. Clinical studies have highlighted its utility in diagnosing diseases in which protein expression levels are key indicators of disease progression or response to treatment.^440^

Integrated digital microfluidics harnesses the combined strengths of digital quantification and precise fluid control, enabling sensitive, high-throughput analysis of EV and SiEV biomarkers while serving as a robust platform for advanced diagnostics such as early tumor detection and multiplexed biomarker profiling. An integrated digital microfluidics combines the advantages of digital quantification with microfluidic platforms, enabling the manipulation and analysis of small volumes of fluid containing EVs or SiEVs. This technology is particularly effective for high-throughput analyses, allowing for the simultaneous processing of multiple samples. Integrated digital microfluidics also offers advantages in automation, speed, and multiplexing, which are critical for large-scale clinical diagnostics. Its potential lies in personalized medicine, where tailored treatment strategies rely on precise biomarker profiling at the SiEV level.^459^ One pioneering development is the digital polymerase chain reaction (dPCR) chip, specifically designed to detect EV-associated biomarkers in saliva for the early diagnosis of tumors. This chip utilizes microfluidic technology with precisely engineered microstructures to partition the sample into thousands of individual reaction chambers, each containing only a few molecules or even a single molecule of interest. By doing so, the dPCR chip allows for highly accurate quantification of biomolecules carried by BuEVs. With exceptional sensitivity, detecting as few as 10 copies of EV lncRNAs, the chip has effectively discriminated lung cancer cases from healthy controls, demonstrating higher precision compared to that of qPCR. Thus, the dPCR chip is a promising technology for noninvasive, early screening of tumors (Figure 14A).^628^

**Figure 14:**
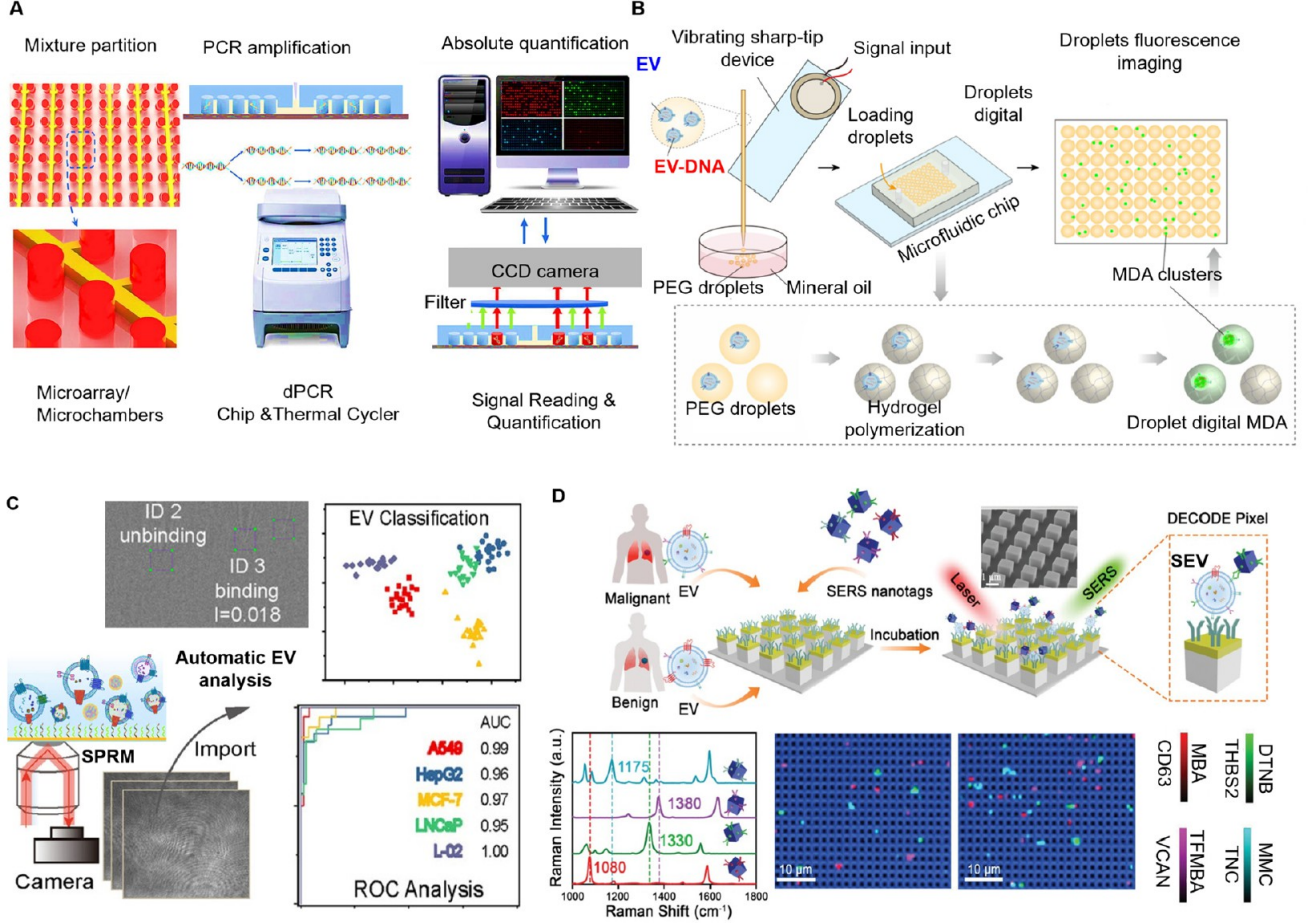
Advanced digital techniques for SiEV analysis. (A) Workflow for digital PCR (dPCR) chip detection of SiEV long noncoding RNAs. The process includes partitioning SiEV mixtures into microchannels and microchambers, PCR amplification, and absolute quantification. Copyright 2023, Elsevier. Reprinted with permission from ref 628. (B) Integrated digital droplet technology with microfluidic chip using hydrogel-based digital droplet multiple displacement amplification (ddMDA) for SiEV DNA analysis. Copyright 2024, American Chemical Society. Reprinted with permission from ref 477. (C) Surface plasmon resonance microscopy (SPRM) for automatic SiEV analysis, capturing SiEVs of varying sizes from biological samples. The developed digital EV analyzer (DEA) software enables size distribution and dynamic single-EV tracking, with classification based on size-dependent surface protein signatures. Copyright 2023, Shanghai Fuji Technology Consulting Co., Ltd., authorized by Professional Community of Experimental Medicine, National Association of Health Industry and Enterprise Management (PCEM) and John Wiley & Sons Australia, Ltd. Reprinted with permission from ref 631. (D) Digital decoding of SiEV phenotypes differentiating early malignant and benign lung lesions. The DECODE chip distinguishes these lesions through SiEV counting and phenotyping using anti-TNC antibody conjugated nanopillars and nanobox-based SERS barcodes targeting CD63, THSB2, VCAN, and TNC. SEM images and SERS spectra (MMC, TFMBA, DTNB, MBA) demonstrate the detection process. Copyright 2022, Wiley-VCH GmbH. Reprinted with permission from ref 458.

In addition, a versatile droplet digital immuno-PCR (ddiPCR) assay combines the high specificity of immuno-PCR with the sensitivity of ddPCR to profile surface proteins of SiEV, enabling the detection and quantification of multiple EV subpopulations within a sample. Immuno-PCR involves attaching DNA tags to antibodies, which bind to specific EV surface proteins, while ddPCR amplifies these DNA tags within individual droplets, allowing for precise and highly sensitive measurements. In a clinical study, the ddiPCR approach simultaneously profiled EV proteins associated with breast cancer and hepatocellular carcinoma, showing significant differences in multisubpopulation EV counts between cancer patients and healthy controls that were based on specific EV surface markers for each cancer type.^629^ Notably, ddiPCR successfully profiled biomarkers such as CD9/CD63/CD81, HER2, and EpCAM for breast cancer and CD9/CD63/CD81, GPC-3, and EpCAM for hepatocellular carcinoma. Further improvements in disease diagnosis could be achieved by integrating ddiPCR with machine learning, promising enhanced accuracy and efficiency in cancer diagnostics.

Recently, researchers have developed a cost-effective dual-color membrane digital ELISA (MemdELISA) platform.^117^ This innovative platform utilizes track-etched polycarbonate membranes, effectively eliminating air bubbles and forming microwells, and enables a duplex digital protein colorimetric assay, allowing for the simultaneous detection of protein biomarkers. It successfully identified breast cancer biomarkers, namely EpCAM^+^ EVs and GPC-1^+^ EVs, from breast cancer cells. The MemdELISA platform exhibits a wide dynamic range and low detection limit, and with easy setup, low cost, and high-throughput capabilities, it shows promise for advancing biomarker detection technologies in various biomedical applications.

Another breakthrough is EV-CLIP, a highly sensitive droplet-based digital method leveraging microfluidic compartmentalization. This approach enables high-throughput digital profiling of EVs containing target miRNA or mRNA.^630^ EV-CLIP employs the fusion of EVs with charged liposomes (CLIPs) within a microfluidic chip. The optimized surface charge of the CLIPs enhances the sensitivity and selectivity for detecting EV-derived miRNAs and mRNAs. This was achieved in a microfluidic droplet reactor, allowing digital investigation of miRNAs and mRNAs within SiEVs. This signal is then digitally detected and quantified in a microfluidic droplet reactor, allowing precise analysis of EV-derived miRNAs and mRNAs. In samples from 73 lung cancer patients and 10 healthy donors, EV-CLIP detected *EGFR* mutations L858R and T790 M in blood plasma with high AUC values: 1 for L858R and 0.9784 for T790M. EV-CLIP eliminates the need for sample preprocessing; it does not require prior EV isolation or RNA preparation, simplifying the detection process while minimizing the loss of BuEVs. Its success in serial monitoring during chemotherapy further suggests its potential for precise quantification of rare EV subpopulations. Consequently, EV-CLIP can facilitate biomarker discovery and enhance our understanding of diverse EV populations in various disease states, particularly in diagnostics.

Furthermore, a rapid and fully automated sample preparation platform using digital microfluidic (DMF) technology has been developed for clinical liquid biopsy of tumors.^459^ This platform integrates EV pretreatment protocols with a reusable DMF chip technique, allowing for automated sample processing in 20–30 min and immediate RT-qPCR analyses of EV-derived miRNAs. According to clinical validation, this platform was effective for detecting biomarkers of NSCLC, such as EV-miR-486-5p and miR-21-5p, and required only a small sample volume (20–40 μL), consistent with results obtained using a commercial exosome miRNA extraction kit. Therefore, this approach provides a simple solution to EV isolation for liquid biopsy, holding promise for early cancer detection.

Moreover, a hydrogel-based droplet digital multiple displacement amplification (ddMDA) approach has been introduced for comprehensive profiling of EV DNA at the SiEV level (Figure 14B).^477^ This method disperses SiEVs in thousands of hydrogel droplets, where they undergo lysis for DNA amplification and identification. The droplet microfluidics strategy provides single-molecule sensitivity and enables absolute quantification of DNA-containing EVs, offering insights into EV DNA heterogeneity in terms of content, distribution, and properties. By encapsulating SiEVs into monodispersed droplets, this approach reduces sample cross-contamination risk and allows for DNA sequencing of ddMDA products, thus providing a robust tool for investigating EV DNA content and its implications for early cancer detection and treatment response monitoring.

To improve SiEV analysis, an automated digital EV analyzer (DEA) has been developed for size-dependent subpopulation analysis in surface plasmon resonance microscopy (SPRM). This software automates SiEV identification, counting, sizing, and dwell time quantification, potentially advancing SPRM technology in cancer diagnosis by accurately grouping EVs from different origins (Figure 14C).^631^

Ultrasensitive SiEV detection using a high-throughput droplet digital enzyme-linked immunosorbent assay (DEVA) enables EV quantification at a detection limit of 9 EVs per μL, greatly surpassing existing gold standard methods. DEVA operates by emulsifying a biological sample into thousands of tiny droplets, each acting as an individual reaction chamber. Inside these droplets, specific capture antibodies bind to target markers on the EVs. After this binding process, an enzyme-linked secondary antibody reacts with a substrate to produce a detectable signal, such as fluorescence or color change, that indicates the presence of EVs. Each droplet is analyzed to determine the signal, enabling precise quantification of EVs, including rare subpopulations, even in complex bodily fluids, demonstrating its clinical potential.^572^

In addition, a digital SiEV counting detection technology known as DECODE represents a significant advancement in noninvasive lung cancer diagnostics (Figure 14D).^458^ This innovative technology captures SiEVs on a nanostructured pillar chip and profiles biomarkers using SERS barcodes, allowing for precise, bias-free digital readouts. By analyzing EV biomarkers such as TNC, CD63, THSB2, and VCAN, DECODE effectively distinguishes between malignant and benign lung lesions with high accuracy (AUC = 0.85), particularly in early-stage cancer. Compared to BuEV methods that provide averaged information and can lead to quantification bias and limited sensitivity, the DECODE approach generates comprehensive, detailed molecular profiles of SiEVs, enhancing diagnostic accuracy and sensitivity. This highlights its potential as a reliable screening tool, one that can reduce the need for invasive tissue biopsies and benefit the broader population through early detection and improved clinical outcomes.

Tyramide signal amplification (TSA) assays can enhance the fluorescent signal readout, compensating for the low relative abundance of proteins in SiEVs.^446^ Utilizing advanced microfluidic technology, this method effectively compartmentalizes SiEVs, demonstrating its capability for digital partitioning. This system shows potential for profiling crucial diagnostic and prognostic cancer biomarkers. Its ability to accurately quantify rare protein molecules from SiEVs offers significant insights into EV heterogeneity and possibly the identification of new biomarkers.

These recent digital-based advances represent a significant leap forward in the understanding and diagnosis of diseases through the detailed analysis of SiEVs, which offers a more precise, sensitive, and cost-effective approach compared to conventional BuEV analysis. These methods enhance early detection, treatment monitoring, and biomarker discovery across various disease states.

### 6.11 Electrochemical Systems

Electrochemical systems detect the interaction of a target analyte with a sensor, transforming analyte concentration into an electrochemical response signal. Common electrochemical systems include cyclic voltammetry, electrochemical impedance spectroscopy, square wave voltammetry, linear sweep voltammetry, and differential pulse voltammetry.^383,632^ Target analytes encompass a broad spectrum, from biomolecules such as DNA, RNA, and proteins to EVs and cells. Thus, these systems offer insights into disease mechanisms and biomarker identification.^360^

Electrochemical biosensors are customizable with specific protein markers such as CD63, CD9, or EpCAM, which are immobilized on electrode surfaces. This approach effectively targets protein biomarkers present on EV membranes, making electrochemical-based techniques indispensable in clinical EV analysis. They offer sensitivity, specificity, and efficiency beyond traditional analysis methods.^633^

Researchers are currently focusing on developing advanced electrochemical techniques, which involve the use of nanocomposite materials with high conductivity and surface area. Integrating these materials with microfluidic systems or magneto-electrochemical methods into 96-well assays has demonstrated enhanced downstream processing, significantly improving the efficiency and performance of EV analysis for clinical samples.^634,635^

Numerous studies have described the development of electrochemical biosensors capable of detecting EV-related DNA, RNA, and protein biomarkers. These approaches provide valuable diagnostic insights by enabling the identification of disease-related EVs. This aids in early diagnosis and monitoring disease progression.^633^ A paper-based electrochemical strip was developed by screen printing conductive ink and modifying it with AuNPs to detect cancer-derived EVs in bodily fluids. The system had a linear range extending to 10^5^ EVs/mL and an limit of detection of 0.7 × 10^3^ EVs/mL, allowing for the detection of αvβ6-expressing cancer cells.^636^

For clinical applications, integrating EV isolation and detection is crucial. A high-throughput integrated magneto-electrochemical (HiMEX) device was developed for this purpose, featuring a 96-well assay that enriches EVs with antibody-coated magnetic beads and also performs electrochemical detection (Figure 15A).^462^ This system utilizes a combination of antibodies targeting clinically relevant tumor biomarkers (EGFR, EpCAM, CD24, and GPA33) for the detection and analysis of colorectal cancer. In a prospective study using this device and involving 90 patients, the burden of tEVs predicted 5-year disease-free survival. Additionally, in a longitudinal analysis of plasma from 11 patients, EV burden declined postsurgery and increased upon relapse. This method achieved a detection sensitivity of 94%, a specificity of 100%, and an accuracy of 96% for colorectal cancer diagnosis using conventional and EV markers.

**Figure 15:**
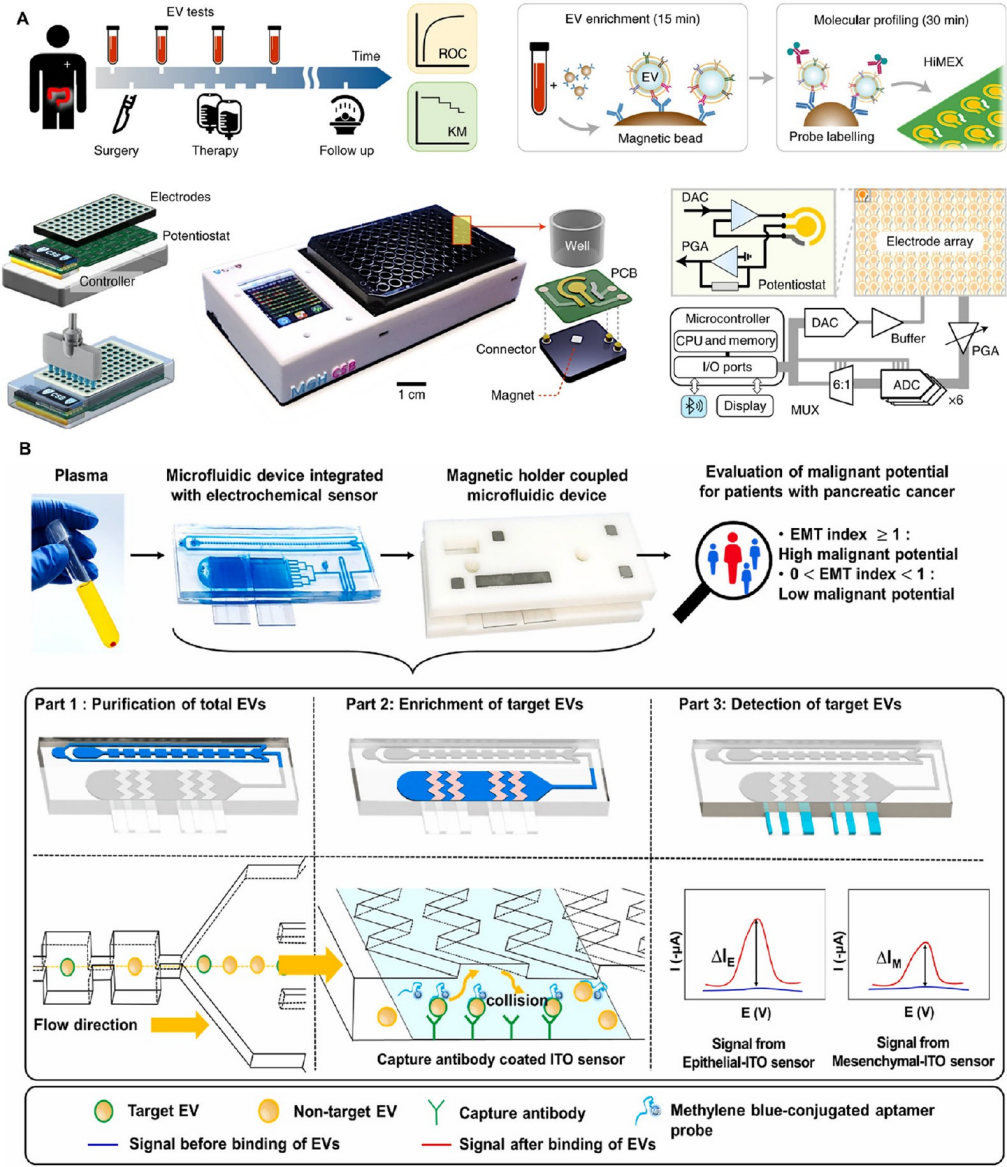
Electrochemical systems for clinical EV analyses. (A) High-throughput integrated magneto-electrochemical (HiMEX) device. This device integrates magneto-electrochemical technology for rapid and efficient profiling of tumor-derived EVs (tEVs) from plasma. Copyright 2021, Springer Nature. Reproduced with permission from ref 462. (B) Electrochemical ITO sensor–integrated microfluidic device. This device consists of 2 key components designed to optimize EV purification and detection. First, the multiorifice flow fractionation (MOFF) channel removes blood cells and debris, delivering a purified EV sample. Next, the geometrically activated surface interaction (GASI) chamber, fitted with electrochemical ITO sensors, enriches and detects EVs with high specificity and efficiency, offering a streamlined approach for EV analysis. Copyright 2023, Elsevier. Reprinted with permission from ref 637.

Microfluidic chips that integrate EV capture with electrochemical detection present an efficient approach for clinical analysis. A recent study evaluating the malignancy of pancreatic cystic neoplasms utilized such a microfluidic device (Figure 15B).^637^ The integrated device consists of 2 essential components: a multiorifice flow-fractionation (MOFF) channel, which effectively extracts pure EVs by removing blood cellular debris, and an indium–tin-oxide (ITO) sensor coupled with a geometrically activated surface interaction (GASI) channel for the enrichment and quantification of tEVs. Specific antibodies immobilized on the ITO surfaces capture tdEVs, showing a linear response between 10^3^ and 10^9^ tdEVs/mL. Antibodies targeting EpCAM and CD49f were used for quantitative EV measurement and an evaluation of the epithelial-to-mesenchymal transition index. Such devices isolate EVs from blood and analyze them directly, enhancing speed, sensitivity, and specificity of clinical EV analysis.

The development of multiplexed electrochemical biosensors enhances the diagnostic capabilities of EV analysis. These biosensors can target multiple EV biomarkers simultaneously, providing comprehensive disease status information from clinical samples. A notable example is the electrochemical sensor that incorporates PbS colloidal quantum dots (CQDs) into the antibody immobilization structure. When a sample of small EVs is detected by this sensor in the presence of an applied electric field, specific capture is mediated by anti-CD63 antibodies. The charge transfer occurring at the binding interface between PbS CQDs and anti-CD63 is converted into electrical signals. This phenomenon is attributed to the abundance of active sites and the distinctive capacitive effect of the CQDs. This sensor achieves quantitative detection of small EVs, covering a dynamic range spanning nearly 6 orders of magnitude and having a detection limit of 19 particles mL^–1^.^635^ Moreover, it has demonstrated clinical applicability, by detecting surface proteins of EVs derived from breast cancer cell lines.

Another innovative method is the proximity-guaranteed DNA machine for accurate identification of EVs released by breast cancer, which is beneficial to explore the subtype features of breast cancer.^638^ This method utilizes a programmable DNA machine, an engineered nucleic acid device designed to detect coexpression of specific EV biomarkers using the CRISPR-Cas12a system. HER-2 and EGFR were chosen as model targets due to their coexpression being associated with poor clinical outcomes in HER-2^+^ breast cancer. The DNA machine is activated through proximity-driven copper-free click ligation, which facilitates the simultaneous targeting of both biomarkers. Once the probes bind to HER-2 and EGFR on the same EV, their proximity triggers the ligation of their terminal modifications, initiating a cascade of DNA reactions. This process amplifies the signal, enabling sensitive and specific identification of breast cancer EVs. The method has shown high sensitivity and specificity when tested with EVs derived from breast cancer cell lines and clinical samples, allowing use not only for the identification of breast cancer patients with special subtypes but also for the staging of tumor progression. Recently, genomic tools such as Argonaute-based sensors have been employed on digital platforms to enable ultrasensitive and multiplex detection, serving as a powerful tool for the analysis of various biomolecules.^639^ We envision these technical advances can be further integrated to improve the throughput in EV analysis.

A new electrochemical biosensing method based on a proximity labeling–assisted click conjugation strategy has been proposed for specific subgroup analyses of circulating EVs. This method, demonstrated with CD44^+^ EVs as a model, showed satisfactory utility for clinical blood samples and versatility with other EV targets, providing reliable guidance for cancer diagnoses and management strategies.^633^

Another noteworthy development is the laser-induced graphene-based electrochemical microfluidic chip for simultaneous analysis of miRNAs. This chip allows precise multiplexed quantification of miR-21, miR-155, and miR-1246, at concentrations ranging from 0.5 to 1000 pM with a limit of detection down to 0.17 pM, 0.11 pM, and 0.24 pM, respectively, demonstrating high sensitivity and specificity and offering a potential tool for detecting exosomes in clinical serum samples.^636^

Electrochemical sensing strategies integrated with aptamers, DNA nanomachines, rolling circle amplification (RCA), and label-free and homogeneous electrochemical techniques have shown promise for tumor-derived exosome detection via simultaneously targeting exosomal MUC1 and PD-L1. This approach was incorporated into a separation-free electrochemical detection assay, and the results strongly correlated with findings from computerized tomography and pathological analyses, demonstrating 100% specificity, 92% sensitivity, and an overall accuracy of 94.6%, with an AUC of 0.97.^640^

Furthermore, field-effect transistors (FETs), are micro electrochemical system, offer an effective strategy for electrical EV sensing. The FET setup includes a gate electrode, a semiconductor (like a graphene film), and EV-specific capture molecules, such as anti-CD63 antibodies, immobilized on the surface of the semiconductor. When an EV binds to these capture molecules, the surface charge of the EV influences the local electric field at the semiconductor interface. This, in turn, affects the current flow through the semiconductor.^641^ FET-based biosensors have been employed for the ultrasensitive detection of various biomolecules at very low concentrations in all types of bodily fluids, without using any labeling and amplification strategies.^642^ These biosensors provide early disease diagnosis with high specificity and sensitivity at a relatively low cost, and they are easily deployable in point-of-care devices.

To ensure the clinical utility of electrochemical techniques for EV analysis, robust evaluation of assay performance is essential. This involves validation studies using clinical samples to assess sensitivity, specificity, and reproducibility. Standardization of assay protocols and quality control measures are necessary for reliability and consistency across different laboratories. Furthermore, the development of point-of-care electrochemical devices holds great promise for rapid and decentralized disease diagnosis using EV analysis. Advanced electrochemical methods offer a precise and versatile approach to detecting EV-derived protein and nucleic acid biomarkers, which can facilitate the early diagnosis and continuous monitoring of various diseases. With continued advancements in assay development, standardization, and point-of-care device design promise to improve clinical diagnosis and improve patient outcomes.

### 6.12 Artificial Intelligence and Machine Learning

AI and ML are important tools for clinical diagnostics by enabling more accurate, efficient, and insightful analysis of the complex data derived from clinical samples. AI refers to systems designed to mimic human intelligence, while ML, a subset of AI, involves algorithms that allow systems to learn from data and make predictions or decisions on the basis of that data.^643^ These technologies are increasingly crucial for identifying patterns and extracting meaningful insights from large, complex data sets, particularly in genomics, proteomics, and other diagnostic fields, where traditional methods struggle to manage the volume and complexity of information.^639,644^ Specifically, ML is enhancing diagnostic accuracy by analyzing data from EVs, ranging from BuEVs to SiEVs, and providing deeper insights into the molecular mechanisms of diseases.

The transformative impact of AI and ML in diagnostics is reflected in numerous applications that demonstrate the ability of these technologies to uncover previously hidden patterns and improve the reliability of clinical decision-making.^645^ Notably, ML has been applied to SERS, a technique with exceptional sensitivity that provides highly detailed information regarding molecular structure. ML algorithms have been employed to analyze the spectral data generated by SERS, helping to identify disease-specific molecular signatures. Without ML, the intricate and vast amount of spectral data would be difficult to interpret manually, and significant patterns relevant for diagnostic purposes might be missed. By automating and refining this analysis, ML models can detect subtle disease-related changes in EV profiles that traditional diagnostic methods might overlook, leading to more accurate and reliable diagnoses.^601,612^

ML has also played a critical role in analyzing data from the procoagulant EV barcode (PEVB) assay, which was developed to assess venous thromboembolism risk in cancer patients.^646^ This assay uses TiO_2_ nanoflowers to capture EVs efficiently, and the data generated is complex and multidimensional. ML algorithms analyze this data, enabling the assay to distinguish between high- and low-risk patients with better accuracy than that of traditional diagnostic methods. ML also enables the identification of subtle differences in EV profiles that would be difficult to assess using conventional statistical methods alone, improving both sensitivity (96.8%) and specificity (97.1%), surpassing traditional analyses for venous thromboembolism. Similarly, ML has played a critical role in analyzing data generated by an electrochemical sensing platform for the detection of gastric cancer. This multibiomarker platform integrates tetrahedron-Dox-AuNPs (TDA) tags with DNA tetrahedrons to detect small EV-derived circRNAs, and it generates complex data sets that require advanced computational techniques for accurate interpretation. ML is employed to analyze the data, enabling the identification of biomarkers associated with early-stage gastric cancer. By leveraging ML, the platform can identify disease signatures with greater precision and speed than traditional methods, facilitating early detection and personalized treatment. The platform is highly specific, sensitive, rapid, and user-friendly, making it an ideal tool for clinical diagnostics.^647^

In another breakthrough, 3 supervised ML feature selection methods were used in conjunction with real-time qPCR to identify plasma sEV, derived miRNA biomarkers associated with PDAC.^199^ This study demonstrates the use of a plasma sEV–miRNA diagnostic signature for distinguishing individuals with PDAC from those without PDAC, those with benign pancreatic diseases, and healthy controls. ML methods, including LASSO regression, random forest, and support vector machine recursive feature elimination, were employed to identify key miRNAs. The miRNA miR-664a-3p emerged as a crucial biomarker, associated with PDAC features such as vascular invasion and poor differentiation, promoting epithelial-mesenchymal transition and angiogenesis. Combining the miRNA signature with the clinical biomarker CA19–9 improved diagnostic accuracy. These findings highlight the potential of sEV-miRNA signatures, supported by ML, as a powerful tool for early PDAC detection and insights into disease mechanisms.

In another study, ML algorithms such as linear discriminant analysis, support vector machine, and logistic regression were integrated with an approach termed DNA cascade reaction triggered individual EV nanoencapsulation (DCR-IEVN) to diagnose HCC from patient serum (Figure 16A).^648^ This integrated approach accurately quantified multiple tEV subpopulations, including EpCAM^+^PDL1^+^ EVs, EpCAM^+^MUC1^+^ EVs, and PDL1^+^MUC1^+^ EVs, without interference from nontumor EVs and particles. It was also able to distinguish between patients with hepatocellular carcinoma, patients with cirrhosis, and healthy donors with remarkable accuracy, surpassing traditional clinical indicators like the AST/ALT ratio, CEA, and AFP. In the validation cohort (30 patients), the trained linear discriminant analysis model (trained on 120 patients) achieved the highest overall accuracy of 93.3%, underscoring the reliability and robustness of this technology. Moreover, this approach streamlined the workflow, requiring only small-volume serum samples and routine clinical devices, facilitating the use of tEVs for diagnosis in clinical practice. Thus, the integration of ML with DCR-IEVN offers a potent analytical tool that holds promise for precise disease diagnosis and marks a significant advancement in the field of personalized medicine.

**Figure 16:**
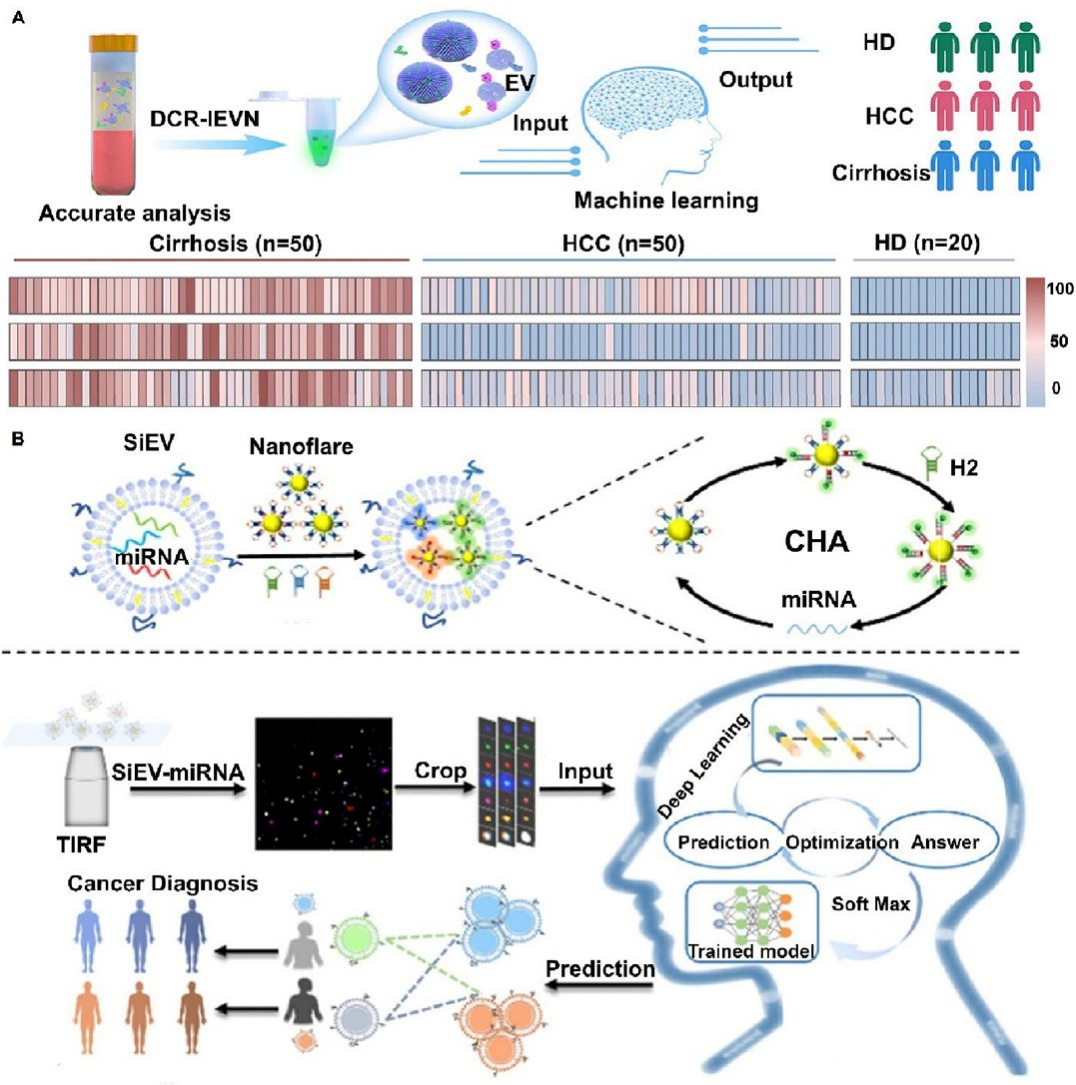
Artificial intelligence and machine learning–based advanced approaches for clinical EV analyses. (A). DNA cascade reaction–triggered SiEV nanoencapsulation (DCR-IEVN) assay. This assay enables the selective profiling of tumor-derived extracellular vesicles (tEVs) in serum samples, which are often mixed with normal cell–derived EVs and free proteins. The DCR-IEVN method employs dual-affinity probes to recognize tEVs, initiating a primer exchange reaction (PER) cycle, followed by hairpin stacking and quantum dot (QD) binding. These steps result in the encapsulation of tEVs into flower-like structures larger than 600 nm. Machine learning is applied to analyze and classify tEV subpopulations, distinguishing between patients with hepatocellular carcinoma, patients with cirrhosis, and healthy donors. Copyright 2024, American Chemical Society. Reprinted with permission from ref 648. (B) Multi-miRNA total internal reflection fluorescence (TIRF) imaging and deep learning algorithm for the automatic detection, analysis, and classification of SiEV images. The multi-miRNA TIRF approach provides a high-resolution platform for SiEV profiling, enabling precise and automated interpretation of EV-derived miRNA signatures in various clinical samples. Copyright 2024, American Chemical Society. Reprinted with permission from ref 466.

For the first time, TIRF imaging was integrated with deep learning, which enhanced the profiling of multiple miRNAs in SiEVs for cancer diagnosis (Figure 16B).^466^ TIRF, with its high resolution and superior signal-to-noise ratio, facilitates the simultaneous in situ detection of multiple miRNAs within SiEVs, and the deep learning algorithm automates high-resolution image analysis, eliminating the need for complex and often inaccurate manual feature extraction. Through this approach, researchers discovered that the most significant variation between EVs from 5 cancer cell types and normal plasma was found in the triple-positive EV subpopulation. The classification accuracy for distinguishing single triple-positive EVs across 6 sources exceeded 95%. In a clinical cohort comprising 20 cancer patients (5 with cancer of the lung, 5, breast; 5, cervix, and 5, colon) and 5 healthy controls, the method achieved a 100% overall prediction accuracy. This SiEV strategy opens new avenues for identifying more specific EV biomarkers, advancing cancer diagnosis and classification.

### 6.13 Other Analytical Approaches

Advanced analytical techniques are continually evolving to enhance the detection and characterization of BuEVs and SiEVs, providing new insights into disease mechanisms and potential biomarkers for diagnosis. Several notable techniques do not fit neatly into the previously mentioned categories, so we discuss them here.

One such technique involves label-free characterization of SiEVs using two-photon fluorescence lifetime imaging microscopy (FLIM) of NAD­(P)­H.^558^ In a proof-of-concept study, researchers imaged EVs derived from various cell lines using FLIM to assess their NAD­(P)H fluorescence lifetime repeatability, heterogeneity, and functional characteristics, with the goal of understanding the relationship between EVs and their parent cells. The study illustrated the feasibility, repeatability, and utility of two-photon FLIM for analyzing NAD­(P)H in isolated EVs. This noninvasive and label-free optical metabolic imaging technique opens up new possibilities for studying and characterizing EVs in greater detail in future research.

Another innovative technique incorporates a novel nanoprobe based on dissociation-enhanced luminescence technology, enabling the analysis of multiple surface proteins on small EVs and facilitating the accurate diagnosis of diseases like breast cancer.^649^ In this technique, small EVs are captured on a solid surface (like a microplate) via antibodies that specifically target CD63, a transmembrane protein commonly found on the surface of small EVs. After the small EVs are immobilized, the nanoprobe comprising NaEuF_4_ nanoparticles conjugated with additional antibodies, including anti-EpCAM, anti-EGFR, and anti-PD-L1, is introduced. These antibodies target specific proteins associated with breast cancer, enhancing the specificity of the detection. To enhance the sensitivity of detection, the NaEuF_4_ nanoparticles serve as tags for signal amplification. When the nanoprobe binds to the targeted surface proteins on the small EVs, the Eu^3+^ ions within the NaEuF_4_ nanoparticles undergo a process called dissolution-enhanced luminescence. In this process, the presence of the targeted proteins activates the Eu^3+^ ions, leading to a luminescent signal that is much stronger than the inherent signals from the EVs themselves. This amplification strategy allows for the detection of multiple surface proteins on the small EVs simultaneously. By detecting these proteins, researchers can gather critical information regarding the presence and abundance of biomarkers related to breast cancer. This method enhances sensitivity and identifies different stages of breast cancer with high accuracy (90.5%), thus aiding in diagnostic efforts.^649^

Another innovative technique, termed signal amplifying vesicles in array (SAViA), has been developed to enhance EV assays by improving the immobilization of EVs for superior sensitivity and throughput (Figure 17A).^400^ The SAViA assay operates by analyzing CD63 titration curves through a dual approach that combines EV physisorption onto a solid support and tyramide-assisted signal amplification to significantly enhance detection sensitivity, achieving a lower estimated detection limit (2.4 × 10^4^ EV/mL) compared to that of the conventional sandwich-type ELISA (3.0 × 10^8^ EV/mL). This heightened sensitivity allows for the detection of biomarkers within a small plasma volume (25 μL), enabling the adoption of a convenient 384-well plate format for high-throughput analysis of multiple biomarkers. This advancement is particularly crucial for the detection of multiple proteins in low-abundance EVs associated with early-stage ovarian cancer. Single markers and their combinations were used as classifiers for cancer patients (*n* = 37) and controls (*n* = 14), with EVs associated with high-grade serous ovarian carcinoma showing the highest AUC with a minimal marker set. A pilot clinical study (*n* = 51) further identified 5 candidate EV markers, EpCAM, CD24, VCAN, HE4, and TNC, whose combined expression distinguished high-grade serous ovarian carcinoma from noncancer with 89% sensitivity and 93% specificity.^400^

**Figure 17:**
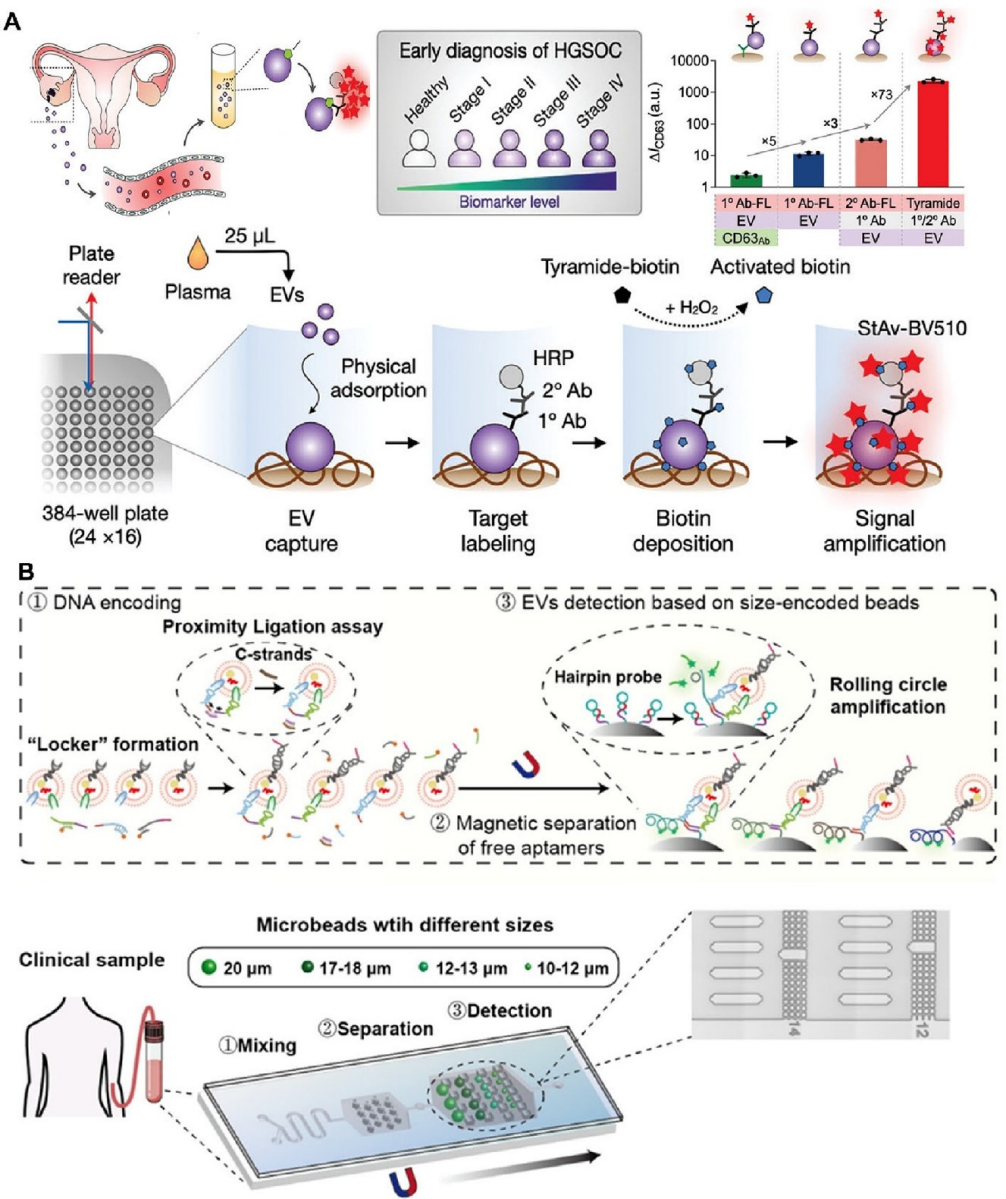
Other advanced techniques for EV Analysis. (A) Signal amplifying vesicles in array (SAViA) for high-grade serous ovarian cancer (HGSOC) detection. EV markers specific to fallopian tube (FT) carcinoma were identified in the blood samples of HGSOC patients, enabling differentiation between early stage (I & II) and late-stage (III & IV) disease. In the SAViA method, EVs are captured on a multiwell plate via physical adsorption. The target EV protein is labeled with a primary antibody (1° Ab), followed by a secondary antibody (2° Ab) conjugated with horseradish peroxidase (HRP). Upon the addition of tyramide-biotin and hydrogen peroxide (H_2_O_2_), HRP catalyzes the dense deposition of biotin, which is subsequently detected by using fluorescent streptavidin (StAv-BV510). Copyright 2023, Wiley-VCH GmbH. Reprinted with permission from ref 400. (B) Proximity ligation assay (PLA)-based approach for EV phenotyping. This method integrates an aptamer/microbead-based assay with a size-based microarray readout platform for EV detection. The triple-marker assay employs EGFR aptamer–modified microbeads to capture EVs, while dual aptamers specific for PD-L1 and EpCAM trigger PLA and subsequent rolling circle amplification (RCA) reactions on captured EVs, enabling highly specific and sensitive EV phenotyping. Copyright 2022, American Chemical Society. Reprinted with permission from ref 651.

Furthermore, a label-free fluorescent biosensor has been developed, utilizing a novel dual aptamer recognition–based approach assisted by functionalized metal–organic frameworks and RCA to achieve ultrasensitive detection of cancer-derived EVs.^650^ In this technique, aptamers are used to convert the protein signal on the EV surface into a nucleic acid signal, which is further amplified by RCA. The fluorescence emitted is positively correlated with the concentration of specific EVs, allowing for highly sensitive and accurate label-free fluorescent detection. Because this technique relies on aptamer–protein recognition for both EV separation and assay processes, it effectively eliminates interference by other impurities in the sample and improves the accuracy of disease diagnosis.

An advanced technique, the size-coded affinity microbead probe strategy for EV phenotyping, utilizes a microfluidic chip with spacer arrays to segregate microbeads by size, enabling location-specific signal generation for different EV biomarkers (Figure 17B).^651^ This approach employs a proximity ligation assay (PLA)–based method for tEV detection and involves three key steps. First, EVs are labeled with biomarker-specific aptamers carrying extended binding sequences, which enable PLA reactions to generate biomarker-specific DNA tags. Second, microbeads of different sizes, modified with complementary hairpin probes, capture tagged EVs after unbound aptamers are removed. Finally, hybridization with hairpin probes triggers toehold-activated RCA for signal readout. This method enables a single fluorophore to detect multiple biomarkers, avoiding signal overlap, and is adaptable for different tEV phenotypes by modifying detected biomarkers. Clinical cohort studies highlight its potential for cancer diagnosis and personalized treatment.

Another notable technique for cancer detection was reported in a recent study.^43^ Using liquid biopsies from breast cancer patients, this technique allows for the precise quantification and profiling of exosome surface proteins via whispering gallery mode (WGM) microlasers. The use of miniaturized laser probes offers high precision and sensitivity, enabling accurate analysis of exosomes and their protein profiles for breast cancer diagnosis.

Another assay combines Förster resonance energy transfer (FRET)-based DNA tetrahedron (FDT) for efficient EV internalization and target mRNA detection, with size-selective thermophoretic accumulation to amplify the FRET signal within EVs (Figure 18A).^468^ The DNA tetrahedron-based thermophoretic assay selectively enhances the detection of PSA mRNA in serum EVs by utilizing the thermophoretic effect, which causes particles to move based on their size in a temperature gradient. This approach achieves an impressive limit of detection of 14 aM for PSA mRNA in serum EVs, without the need for RNA extraction and enzyme amplification. The assay demonstrated that EV PSA mRNA is more effective than serum PSA protein, the current gold standard in prostate cancer screening, in distinguishing between prostate cancer and benign prostatic hyperplasia (AUC: 0.93 vs 0.74; *n* = 42 patients). This innovative technology has the potential to significantly broaden the applications of DNA nanostructure-enabled liquid biopsy.

**Figure 18:**
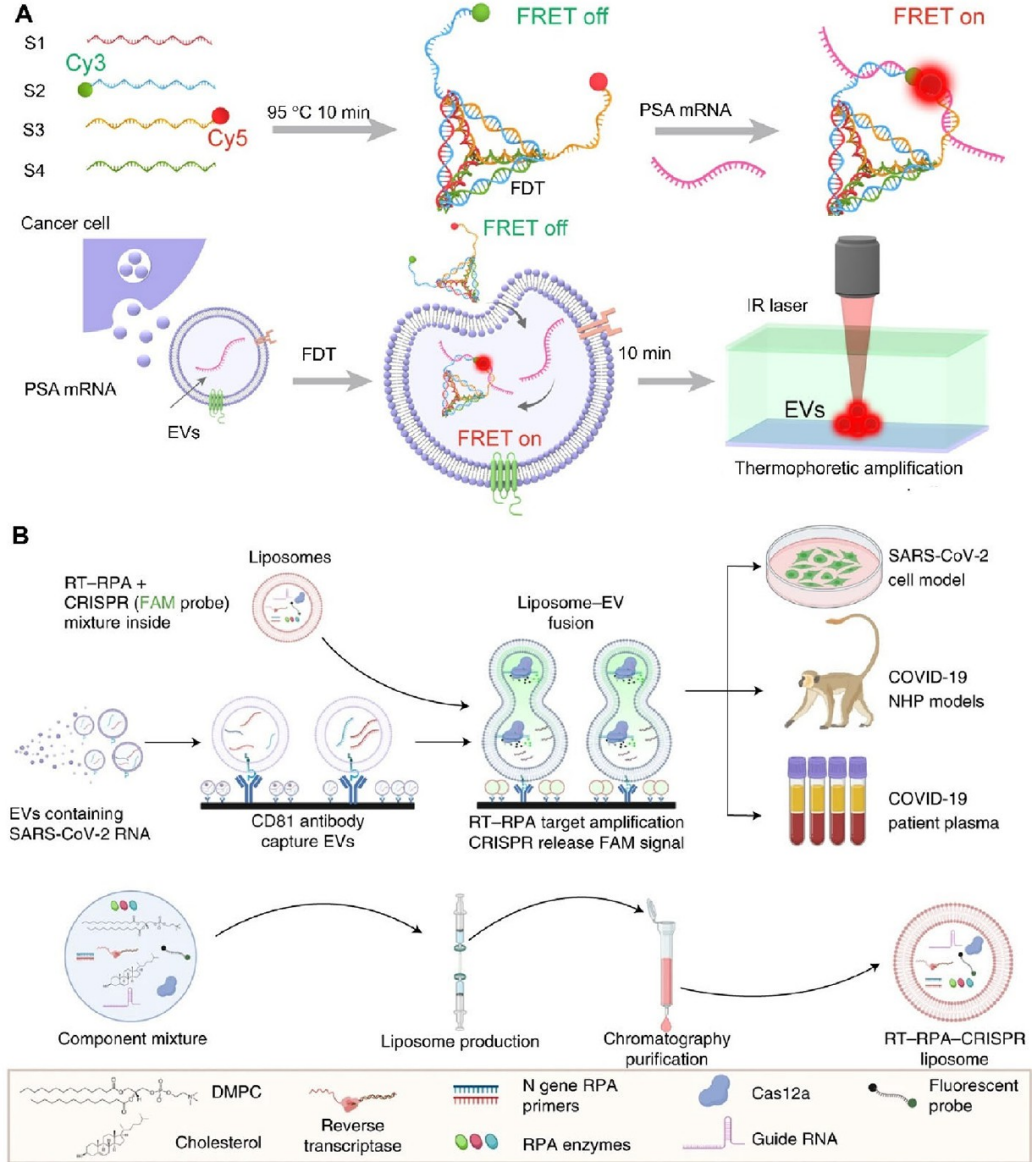
Förster resonance energy transfer (FRET)–based techniques for EV mRNA and miRNA detection. (A) DNA tetrahedron-based thermophoretic assay (DTTA) for EV mRNA detection. This method integrates a FRET-based DNA tetrahedron (FDT) for efficient EV internalization and target mRNA detection with size-selective thermophoretic accumulation to amplify the FRET signal. The FDT consists of 2 recognition sequences labeled with Cy3 and Cy5 fluorophores, which switch the FRET signal from “off” to “on” upon binding to target mRNA. To facilitate passive internalization into EVs, FDT adopts a corner-attachment mode, overcoming the energy barrier of EV membranes. Once inside the EVs, FRET activation occurs upon target mRNA binding, and subsequent thermophoretic enrichment enhances the signal, enabling ultrasensitive in situ detection of EV mRNA. Copyright 2021, Elsevier. Reprinted with permission from ref 468. (B) CRISPR-enhanced RT–RPA fluorescent detection system (CRISPR-FDS) is an advanced diagnostic platform for the ultrasensitive detection of SARS-CoV-2 RNA using EVs. The CRISPR-FDS assay involves CD81-mediated capture of EVs, followed by their fusion with RT–RPA–CRISPR-loaded liposomes. This fusion facilitates RT–RPA-mediated target amplification, generating a fluorescent signal through CRISPR-induced cleavage of a quenched fluorescent probe. The intensity of the signal correlates with the concentration of target amplicons. The assay was performed with cell culture media and plasma samples from nonhuman primate (NHP) COVID-19 disease models and COVID-19 patients. FAM, carboxyfluorescein. The RT–RPA–CRISPR liposome synthesis workflow and reagents include DMPC (1,2-dimyristoyl-*sn*-glycerol-3-phosphorylcholine). Copyright 2021, Springer Nature. Reproduced with permission from ref 253.

The all-in-one fusogenic nanoreactor (FNR) is an innovative diagnostic platform for rapid detection of EV miRNAs.^652^ It incorporates DNA-fueled molecular machines (DMMs) that, via their hemagglutinin (HA) protein coating, mimic the fusogenic properties of enveloped viruses and facilitate selective fusion with EVs. This fusion allows DMMs to recognize target miRNAs and trigger a cascade strand-displacement reaction, leading to nonenzymatic signal amplification and fluorescent signal generation within 30 min. The FNR has demonstrated 86.7% accuracy in classifying major breast cancer cell lines and 86.4% accuracy in differentiating cancer patients from healthy controls. Increasing the number of EV miRNAs (miR-200c, miR-222, and miR-375) analyzed from 1 to 3 improved patient discrimination accuracy from 78.8 to 95.4%. This platform offers a nondestructive, straightforward approach for EV processing and signal amplification in a single step, eliminating time-consuming procedures like EV isolation, RNA extraction, and enzymatic amplification. The FNR presents a powerful, mix-and-read solution for sensitive detection of EV miRNAs, enhancing personalized breast cancer treatment.

The CRISPR-enhanced RT-RPA fluorescent detection system (CRISPR-FDS) represents an advanced diagnostic platform for the ultrasensitive detection of SARS-CoV-2 RNA using EVs. This assay leverages a FRET dequenching method, where the FRET activity decreases as the liposome/EV ratio increases. This dilution effect of FRET dyes in the liposome membrane leads to a reduction in the donor and acceptor signals, enhancing and attenuating the respective signals. The CRISPR-FDS assay begins with the capture of EVs from plasma using antibodies targeting the CD81 surface protein. These captured EVs are then fused with liposomes containing reverse transcriptase (RT), recombinase polymerase amplification (RPA), and CRISPR-Cas12a reagents. This fusion facilitates a workflow similar to ELISAs commonly used in clinical diagnostics. The CRISPR-Cas12a component, guided by RNA, binds to an RT-RPA amplicon, triggering concentration-dependent cleavage of a quenched oligonucleotide probe, enabling the highly sensitive detection of SARS-CoV-2 RNA. By combining antibody-mediated EV capture with liposome-mediated reagent delivery, the CRISPR-FDS assay demonstrates exceptional sensitivity, outperforming RT-qPCR in some cases. The assay can detect SARS-CoV-2-positive EVs as early as day 1 postinfection, with signals detectable for up to 28 days in nonhuman primates and 20–60 days in young children. This nanoparticle-based, noninfectious method significantly extends the detection window, enabling the diagnosis of COVID-19 in patients without detectable RNA in the respiratory tract, offering a promising advancement in clinical diagnostics (Figure 18B).^253^

As these technologies continue to evolve, they hold promise for advancing clinical diagnostics and improving patient outcomes. However, further research and validation are necessary to realize their full potential in clinical practice.

## 7 Clinical Utility and Evaluation

### 7.1 Clinical Utility

EVs carry diverse biomolecules, such as proteins and miRNAs, that reflect physiological and pathological states, making them valuable disease biomarkers. Validating EV-associated biomarkers and detection methods in clinical samples is a crucial step toward advancing disease understanding and improving diagnostics. This serves as a key milestone in integrating EV-based diagnostics and therapeutics into clinical practice, enabling more precise and personalized interventions. Table 3 shows several representative examples of preclinically validated EV-associated biomarkers, detailing their disease relevance, isolation and analysis methods, and clinical cohort assessments. However, variations in sample collection, isolation, and detection methods complicate hinder direct comparisons of diagnostic performance, posing a key challenge highlighting the need for future research. Standardized protocols and rigorous validation in well-characterized patient cohorts are essential to ensure the reliability and clinical translation of these biomarkers.

**Table 3:** EV-Associated Biomarkers in Clinical Diagnostics: Impact of Isolation and Analysis Techniques^a^

isolation method	advanced analysis technique or instrument	pathological condition	EV type	biomarkers	type of clinical samples	total patients (*n* = )	AUC/ROC	sensitivity (%)	pecificity (%)	refs
EXODUS	ML/RAPIDx/MALDI-TOF MS	acute pancreatitis	BuEVs	SAA1–1, desR-SAA1–2, SAA2, SAA1–2	plasma	115	0.92–0.97	N/A	N/A	^598^
TEI kit	ILN/TIRF microscopy	pancreatic cancer	SiEV	CA19–9, GPC1 mRNA	blood, serum, or plasma	91	0.92–0.97	N/A	100	^118^
N/A	3D-EGN/SERS	pancreatic, prostate, lung, colorectal cancer	BuEVs	HER2	urine	218	0.96	80	90	^653^
EIC	MXene-coated Au@Ag NP/SERS	thyroid cancer	BuEVs	N/A	blood, plasma	100	0.96	95	100	^613^
UC/SEC	TiO_2_/PEVB	venous thromboembolism	BuEVs	N/A	blood	193		97.1	96.8	^646^
exosome purificationkits	ML/qRT-PCR	pancreatic cancer	EV-miRNAs	miR-664a-3p, miR-664a-3p	plasma	251	0.78–0.97	73.7–89.5	97.0–100	^199^
UC	ML/qRT-PCR	inflammatory bowel disease (IBD)	EV-ln *R*NA	lncRNA H19	blood, plasma	68	0.86–1	87–100	82–100	^654^
UC	SPR/AI	hepatocellular carcinoma	BuEVs	EpCAM+PDL1+ EVs, EpCAM+MUC1+ EVs, and PDL1+MUC1+ EVs	tumor	150	0.93–0.97	N/A	N/A	^648^
UC	NaEuF_4_ nanoprobe/luminescence	breast cancer	BuEVs	CD63/EpCAM	plasma	21	0.93	90.5	N/A	^649^
UC	SAViA	ovarian cancer	BuEV	EpCAM, CD24, HE4, VCAN, TNC	plasma	71	0.95	89	93	^400^
density gradient UC	HiMEX	colorectal cancer	BuEVs	EGFR, EpCAM, CD24, GPA33	blood, plasma	142	0.96	94	100	^462^
UC	electrochemical system/ITO	NSCLC	BuEV	MUC1, PD-L1	tumor	37	0.97	94.6	92	^640^
UC	AuNPs-Tetrahedron/Electrocehmical (DPV)	gastric cancer	sEV-circRNA	SiEV-circNRIP1, SiEV-circRANGAP1, SiEV-circCORO1C, SiEV-circSHKBP1	plasma	75	0.85–1	60.5–95	61.5–95	^647^
N/A	flow cytometry	Parkinson disease	EV α-Synuclein	L1CAM	tissue	576	0.9	81	87	^655^
UC	nanopalsmonic/Cu-NEI		BuEV	LAM	serum	31	0.92	76.2	100	^616^

a This table provides several representative examples of EV-associated biomarkers and their relevance to various diseases, including the isolation techniques employed, advanced quantitative methods or instruments used for biomarker characterization, associated pathological conditions, types of EVs analyzed, identified biomarkers, clinical sample sources, and key diagnostic performance metrics (AUC/ROC, sensitivity, specificity).

### 7.2 Current Clinical Trials of EVs for Diagnostic Applications

EVs have garnered significant attention in recent years for their potential as biomarkers for the noninvasive diagnosis and prognosis of various diseases. This has led to over 112 registered clinical trials exploring EVs, with more than 40 focusing specifically on their role in diagnostics. The clinical application of EV-based diagnostics spans several major diseases, including cancer, neurodegenerative conditions, and cardiovascular diseases, with efforts particularly focused on early detection. Table 4 provides a summary of registered clinical trials investigating EVs for diagnostic purposes, with data sourced from ClinicalTrials.gov (https://clinicaltrials.gov/) accessed on March 8, 2025. Although numerous clinical trials have been initiated to explore the clinical utility of EVs, relatively few have successfully progressed through all stages of validation, highlighting the significant bottleneck that impedes their widespread adoption in clinical practice. This bottleneck stems from several challenges, including the lack of standardized protocols for EV isolation, which leads to variability in the quantity and quality of EVs across different samples. Additionally, issues with reproducibility, the complexity of detecting low-abundance biomarkers, and the difficulty of achieving high sensitivity and specificity in assays hinder progress. Furthermore, regulatory hurdles, limited large-scale clinical validation, and the need for robust and cost-effective diagnostic platforms further complicate the transition of EV-based diagnostics from research settings to routine clinical use. Despite these obstacles, the growing interest in EVs reflects their potential as pivotal components of liquid biopsy strategies, with the capability to transform early disease detection and patient monitoring.

**Table 4:** Summary of Registered Clinical Trials Investigating EVs for Diagnostic Applications

trial identifier	year	trial name	disease focus	EV subtypes	EV biomarker(s)	sample type(s)	status	key outcomes/goals
NCT02702856	2016	clinical validation of a urinary exosome gene signature in men presenting for suspicion of prostate cancer	prostate cancer	exosomes RNAs	exosomal RNA (ExoDx EPI score)	urine	completed	To validate the Exosome Urine Test for excluding high Gleason grade prostate cancer with high certainty and evaluate its broader applicability for predicting prostate cancer presence, supporting its use in clinical practice as a noninvasive diagnostic tool.
NCT03775447	2021	Fox BioNet Project: ECV-003	Parkinson disease	EVs	LRRK2	CSF	completed	To optimize preanalytical CSF EV isolation protocols for increasing the detection of LRRK2 activity in human CSF.
NCT04913545	2021	the sensitivity and specificity of using salivary miRNAs in detection of malignant transformation of oral lesions	malignant transformation of oral lesions	EV miRNAs	EV miRNAs 412, 512	saliva	completed	To assess whether salivary miRNAs 412 and 512 can serve as reliable biomarkers for early detection of malignant transformation in potentially malignant oral lesions, with the outcome being compared to biopsy diagnoses for validation.
NCT04720599	2021	clinical evaluation of ExoDx prostate (IntelliScore) in men presenting for initial prostate biopsy	prostate cancer	exosomes RNAs	exosomal RNA (ExoDx EPI score)	urine	completed	To confirm the performance of the ExoDx prostate gene expression assay in patients presenting for an initial prostate biopsy and support of CE-marking, the test for a European Union Launch.
NCT04603326	2023	FoxBioNet: ECV (Extracellular Vesicle) 004	Parkinson disease	EVs	LRRK2	CSF	completed	To identify reliable markers of LRRK2 activity in CS, specifically to distinguish pathogenic LRRK2 variant carriers from idiopathic Parkinson disease patients and healthy controls.
NCT04164134	2023	new strategies to detect cancers in carriers of mutations in RB1 (NIRBTEST)	childhood cancer retinoblastoma (Rb)	EVs		blood	completed	To identify biomarkers for the early detection and monitoring of second primary malignancies (SPMs) in RB1-mutation carriers, while minimizing participant burden by utilizing existing clinical visits and reducing the need for additional venipunctures.
NCT06202547	2025	saliva and extracellular vesicles for Parkinson disease (RaSPiD)	Parkinson disease	EVs		saliva	completed	To validate a novel diagnostic tool for early and accurate diagnosis, as well as personalized rehabilitation for Parkinson disease and atypical Parkinsonism, leveraging salivary EVs and Raman spectroscopy to offer a fast, reliable, and noninvasive way of assessing patient status and therapeutic responses.
NCT04523389	2020	contents of circulating extracellular vesicles: biomarkers in colorectal cancer patients (ExoColon)	colorectal cancer	exosomes	EV miRNAs	blood	recruiting	To validate circulating exosomes as biomarkers for early diagnosis and prognosis in colon cancer, using the stability and specificity of miRNAs. This noninvasive approach could improve early cancer detection and monitor treatment effectiveness, with potential for broader personalized medicine applications.
NCT05417048	2023	clinical study of glycosylated extracellular vesicles for early diagnosis of breast cancer	breast cancer	EVs	CD63, CD9, EV miRNAs	blood	recruiting	To evaluate the clinical diagnostic performance of glycosylated EVs and their contents for early detection of breast cancer. The study will isolate glycosylated EVs from the serum of cancer and noncancer patients using the GlyExo-Capture technology.
NCT05625529	2023	ExoLuminate study for early detection of pancreatic cancer	pancreatic cancer	EVs		blood	recruiting	To establish whether the ExoVerita assay can serve as a reliable and noninvasive tool for the early detection of PDAC in high-risk populations, potentially providing a better alternative to current methods.
NCT06169540	2023	salivary extracellular vesicle associated lncRNAs in heart failure (SEAL-HF)	heart failure	EVs	EV lncRNAs	blood, plasma, saliva	recruiting	To determine the relationship between the levels of ribonucleic acid (RNA) circulating molecules, including ones in EVs from different organs in the blood and in the saliva of patients with acute decompensated heart failure (ADHF) and chronic heart failure (CHF) to see if a new, noninvasive diagnostic test can be developed for heart failure exacerbation.
NCT06298682	2024	characterization of exosome platelets-released	cardiovascular disease	exosomes, lEVs, and apoptotic bodies		blood	recruiting	To explore the potential of platelet-derived EVs as biomarkers for cardiovascular disease and understand how antiplatelet agents impact their release and content, advancing the clinical application of EVs in diagnostics.
NCT06672302	2024	a prospective study to develop and clinically validate an in vitro diagnostic medical device that uses blood to classify patients at high risk for breast cancer	breast cancer	EVs		blood	recruiting	To focus on the application of SERS to analyze EVs for the classification of high-risk and low-risk breast cancer patients. The goal is to validate a novel liquid biopsy–based diagnostic device, combining SERS with AI to enhance early detection and reduce unnecessary biopsies.
NCT05798338	2024	characterization of extracellular vesicles in breast cancer patients	breast cancer	EVs	EV miRNAs	tumor	recruiting	To evaluate how the single molecule array (SiMoA), a digital ELISA technology capable of detecting extremely low concentrations of EV-associated proteins, can enable the quantification of EVs in plasma from patients with breast cancer. This approach aims to provide valuable diagnostic and prognostic information by assessing EV levels in breast cancer patients.
NCT06607900	2024	HUC-MSC-sEV-001 nasal drops for neurodegenerative diseases	Alzheimer disease, Parkinson disease, multiple system atrophy, Lewy body dementia, and frontotemporal dementia	exosomes			not yet recruiting	To evaluate the safety and preliminary efficacy of human umbilical cord mesenchymal stem cell–derived sEVs hUC-MSC-SiEV-001 nasal drops in multiple neurodegenerative diseases, including Alzheimer disease, Parkinson disease, multiple system atrophy, Lewy body dementia, and frontotemporal dementia.
NCT06408961	2024	EVOC—EVs in obesity and cardiometabolic disease	obesity and cardiometabolic disease	EVs, EV mRNAs		blood, subcutaneous, visceral fat tissue	not yet recruiting	To research the impact of molecular signals from the heart, liver, and fat tissue on cardiovascular disease risk, and the presentation of Type II diabetes and diseases that affect the heart, blood vessels and metabolism (cardiometabolic disease). Specifically, the focus is on the content and function of EVs.

#### 7.2.1 Completed Clinical Trials

The exploration of EVs as versatile biomarkers has gained significant momentum through initiatives like the Fox BioNet initiative and related clinical trials. These efforts exemplify the potential of EVs as noninvasive diagnostic tools across various diseases.

The Fox BioNet initiative includes 2 projects advancing cerebrospinal fluid EV research in Parkinson's disease. Fox BioNet Project ECV-003 (NCT03775447) focuses on optimizing preanalytical protocols for CSF EV isolation to enhance the detection of leucine-rich repeat kinase 2 (LRRK2) activity. Key objectives include evaluating various isolation methods to enrich biomarkers such as LRRK2, phosphorylated LRRK2 (p1292-LRRK2), Rabs, and phosphorylated Rabs (pRabs), as well as ensuring interlaboratory reliability, network efficiency, standardized protocol adherence, and robust biosample collection. Building on these efforts, Fox BioNet Project ECV-004 (NCT04603326) aims to identify reliable markers of LRRK2 activity by assessing the performance of assays, including quantitative Western blot, immunoassays, and LC-MS to measure LRRK2 activity and its downstream pathways. By prioritizing assays with high sensitivity and reproducibility, the project seeks to differentiate pathogenic LRRK2 variant carriers from idiopathic Parkinson's disease patients and healthy controls. Beyond the Fox BioNet initiative, other completed clinical trials have further demonstrated the utility of EV-based diagnostics. For example, the trial, New Strategies to Detect Cancers in Carriers of Mutations in RB1 (NCT04164134), developed noninvasive cancer tests based on RNA sequencing data from platelets (ThromboSeq) or EVs derived from tumor cells in blood. This trial aimed to determine the noncancerous baseline in adult RB1-mutation carriers (heritable-Rb survivors) and contribute to the biobanking of blood and tissue samples for future research. The platelet or EV-based tests offer promising potential for early tumor detection in RB1-mutation carriers. Another significant study is The Sensitivity and Specificity of Using Salivary miRNAs in Detection of Malignant Transformation of Oral Lesions (NCT04913545), which evaluated the diagnostic accuracy of salivary EV-derived miRNAs (miR-412 and miR-512) in detecting malignant transformation. Through EV isolation and qRT-PCR analysis, this study demonstrated that salivary miRNAs could serve as noninvasive biomarkers for the early detection of oral cancer. Furthermore, a newly completed clinical trial advancing EV-based diagnostics, the Saliva and Extracellular Vesicles for Parkinson's Disease (RaSPiD) trial (NCT06202547), explored the potential of salivary EVs in neurodegenerative disease diagnostics. This study aimed to validate a novel diagnostic tool for the early and accurate detection of Parkinson's disease and atypical Parkinsonism, leveraging salivary EVs and Raman spectroscopy. By providing a fast, reliable, and noninvasive method for assessing patient status and therapeutic responses, this approach represents a promising advancement in personalized rehabilitation strategies for Parkinson's disease.

Collectively, these studies underscore the role of EV-based diagnostics in advancing precision medicine and enabling early disease detection, indicating their promise for broader clinical applications.

#### 7.2.2 Active and Recruiting Clinical Trials

Several ongoing trials explore the diagnostic potential of EVs in a variety of diseases. The Salivary Extracellular Vesicle Associated lncRNAs in Heart Failure (SEAL-HF) (NCT06169540) trial aims to determine the diagnostic value of salivary and blood EV-derived RNAs in distinguishing acute decompensated heart failure (ADHF) from chronic heart failure (CHF). This multicohort study seeks to correlate EV RNA levels in plasma and saliva, providing a potential noninvasive diagnostic tool for heart failure exacerbation. The trial Circulating Extracellular Vesicles: Biomarkers in Colorectal Cancer Patients (NCT04523389) focuses on the role of circulating EV-derived miRNAs as biomarkers of disease progression in colon cancer. Because of their stability and protection from RNAase degradation, exosomal miRNAs are being investigated for their potential to serve as early diagnostic and prognostic biomarkers in colorectal cancer. The ExoLuminate Study for Early Detection of Pancreatic Cancer (NCT05625529) is designed to compare the performance of the ExoVerita assay with standard-of-care methods in detecting PDAC. This study targets individuals with elevated risk for PDAC, aiming to improve early detection of this highly lethal cancer through noninvasive EV-based assays. Other notable ongoing trials include the Application of Circulating Extracellular Vesicles in Early Disease Assessment and Prognosis After Traumatic Brain Injury (NCT05279599), which investigates EV-derived biomarkers as early indicators of traumatic brain injury (TBI) outcomes. Additionally, the trial Circulating EV Long RNA Profiles in SCLC (NCT05191849) aims to identify EV-derived long RNAs as biomarkers for predicting therapeutic responses in small cell lung cancer (SCLC).

#### 7.2.3 Emerging Technologies and Platforms

Emerging diagnostic platforms like ExoLuminate show promise in enhancing the sensitivity and specificity of detecting disease-related EVs. One cutting-edge platform currently under investigation is the Single Molecule Array (SiMoA), a digital ELISA technology capable of detecting extremely low concentrations of EV-associated proteins. This platform is being tested in breast cancer patients (NCT05798338), where it holds promise as a noninvasive method for quantifying plasma EV levels, providing diagnostic and prognostic insights, particularly in relation to neoadjuvant treatments. Similarly, the Clinical Study of Glycosylated Extracellular Vesicles for Early Diagnosis of Breast Cancer (NCT05417048) uses the novel GlyExo-capture technology to isolate glycosylated EVs from patient serum. This prospective study integrates ML with miRNA sequencing to develop an early diagnostic model for breast cancer, validated by qPCR.

#### 7.2.4 Other Investigational Applications

In the metabolic disease domain, the EVs in the Obesity and Cardiometabolic Disease (EVOC) study (NCT06408961) focus on EV-derived proteins, RNAs, and metabolites to assess their role in cardiovascular risk and type II diabetes. This study explores how EVs from subcutaneous and visceral fat influence heart and liver cells, with implications for cardiometabolic disease research. The Extracellular Vesicle Micro RNA Profiling in Congenital Heart Disease (EVmiRNA) trial (NCT06434207) investigates the role of EV-derived miRNAs in regulating clotting mechanisms in newborns with congenital heart disease (CHD). This study aims to better understand perioperative clotting profiles and to identify novel biomarkers for thrombosis in infants with severe CHD. Finally, the HUC-MSC-sEV-001 Nasal Drops for Neurodegenerative Diseases (NCT06607900) trial evaluates the safety and preliminary efficacy of human umbilical cord mesenchymal stem cell–derived small EVs (hUC-MSC-sEV-001) administered as nasal drops in patients with neurodegenerative diseases such as Alzheimer's and Parkinson's disease. This novel approach leverages the therapeutic potential of EVs to target neurodegenerative disorders.

EVs have emerged as promising diagnostic biomarkers, with ongoing clinical trials primarily focusing on the bulk population of EVs and their collective content of nucleic acids, proteins, and lipids. The potential of EV-based diagnostics to improve early disease detection, prognostics, and patient monitoring is evident. Despite the challenges of sensitivity, specificity, and standardization, the successful integration of EV-based diagnostics into routine clinical practice could facilitate earlier interventions and more accurate disease monitoring. Although single-EV analysis has not yet been fully realized in clinical trials because of the technical challenges of isolating and characterizing SiEVs, the potential benefits are substantial. As technologies for single-vesicle detection and analysis continue to advance, integrating SiEV analysis into clinical trials appears inevitable, promising to unlock new diagnostic frontiers and move EV-based diagnostics closer to routine clinical use.

### 7.3 Validation of SiEV Analysis in Clinical Diagnostics

SiEV analysis represents an exciting frontier in clinical diagnostics, offering a potential solution to the limitations of BuEV analysis. By focusing on SiEV analysis, researchers are uncovering detailed insights into molecular and functional heterogeneity, which is particularly valuable in complex diseases like cancer, where tumor heterogeneity presents significant challenges. Validating SiEV for clinical diagnostics involves a multifaceted approach, integrating cutting-edge technologies such as single-particle interferometric reflectance imaging sensor (SP-IRIS), total internal reflection fluorescence (TIRF) microscopy, immunomagnetic-activated cytometry (NanoEPIC), SiEV-RNA sequencing, digital droplet PCR, immuno-digital droplet PCR (iddPCR), droplet-based single-exosome-counting ELISA (droplet digital ExoELISA), nanopore, and nano plasmonic (nPES). These methods allow precise characterization of SiEVs in terms of size, concentration, and molecular content. Clinical trials for SiEV-based diagnostics should begin with the development and optimization of isolation techniques, such as microfluidics and immunocapture-integrated nanomaterials, tailored to enrich specific subpopulations of SiEVs. Subsequent phases should involve comparative studies with established diagnostic standards, like tissue biopsies and blood-based biomarkers, to demonstrate the clinical utility of SiEV analysis. Key validation metrics include analytical sensitivity, specificity, reproducibility, and clinical relevance. Incorporating advanced methodologies like droplet digital PCR and super-resolution microscopy has enhanced the accuracy and reliability of SiEV measurements, providing robust data for clinical decision-making. As the field progresses, large-scale multicenter trials and longitudinal studies are essential to validate the clinical efficacy of SiEV diagnostics across diverse populations and disease states. The future of SiEV analysis in clinical diagnostics is promising, with potential applications in early disease detection, personalized medicine, and real-time therapeutic monitoring. Addressing current challenges, such as developing high-throughput, cost-effective, and standardized platforms, will be critical to fully realizing the clinical potential of SiEV. Collaboration among researchers, clinicians, and industry stakeholders, supported by regulatory oversight, will be crucial for successfully translating SiEV technologies into routine clinical practice.

### 7.4 Comparison of BuEV Analysis and SiEV Analysis Technologies

EV analysis plays a crucial role in advancing biomedical and clinical research, with distinct methodologies for BuEV and SiEV analysis. A comparative evaluation of these approaches highlights their respective advantages and limitations, offering insights into their respective applicability in clinical diagnostics. These approaches differ in aspects such as sensitivity, molecular profiling and specificity, sample requirements, throughput and scalability, data complexity and interpretation, cost and accessibility, and clinical use cases.

BuEV analysis amplifies signals from large vesicle populations, making it effective for detecting abundant biomarkers but less suited for rare ones. In contrast, SiEV analysis isolates individual vesicles, enhancing rare biomarker detection, though background noise remains a challenge. BuEV enables broad, molecular profiling for large-scale screening but lacks the resolution to distinguish EV subpopulations. SiEV offers high specificity and in-depth analysis, essential for personalized medicine and precise disease characterization. While BuEV requires larger sample volumes, which may not always be available in clinical settings, SiEV’s higher sensitivity allows for lower sample requirements. However, both methods demand high-purity EV isolation, which remains technically challenging, time-consuming, and resource-intensive. Additionally, handling small EV volumes requires precision to ensure reliable results and avoid variation between batches.

From a throughput and scalability perspective, BuEV analysis is advantageous. Its high-throughput nature makes it well-suited for large-scale studies, such as population-based screenings and clinical diagnostics. Its ability to process large numbers of samples efficiently is a significant advantage, especially in clinical settings with many patients. In contrast, SiEV analysis, while offering high specificity, faces limitations in throughput and scalability. The need for high-purity EV isolation, careful handling of small sample volumes, and the use of more complex detection methods all contribute to the increased demands in terms of time, resources, and technical expertise. As a result, compared to BuEV methods, SiEV analysis is less suited for large-scale studies.

Data interpretation in BuEV analysis is relatively straightforward due to its reliance on well-established techniques. SiEV analysis, on the other hand, requires advanced computational tools and expertise to accurately interpret the data. This complexity in interpretation can hinder its routine clinical implementation, although the insights provided are far more precise.

BuEV analysis tends to be more cost-effective, with well-established technologies such as ELISA and flow cytometry enabling easy integration into clinical workflows. In contrast, SiEV analysis requires specialized equipment, such as microfluidics or nanopore sensing devices, along with technical expertise, making SiEV analysis more expensive and less accessible for routine use. This restricts its use primarily to research settings or specialized clinical applications.

In summary, BuEV analysis is well-suited for broad, high-throughput screening and large-scale disease monitoring, with the benefit of being cost-effective and clinically accessible. However, it is not capable of detecting rare, disease-specific biomarkers or distinguishing EV subpopulations, limiting its utility in precision medicine and early-stage disease detection. SiEV analysis, on the other hand, excels in detecting rare biomarkers and providing precise molecular profiling, making it invaluable for early disease diagnosis and personalized medicine. Despite its promise, the high cost, low throughput, and technical complexity of SiEV analysis present barriers to its widespread adoption.

Table 5 offers a detailed comparison of BuEV and SiEV analysis technologies, providing insights into their respective strengths and weaknesses. Moving forward, integrating both approaches, leveraging the sensitivity of BuEV analysis for broad screening and the specificity of SiEV analysis for detailed molecular profiling, may offer a more comprehensive solution for EV research and clinical diagnostics. Future advancements in SiEV technologies, particularly advancements addressing challenges related to sensitivity, scalability, and cost, will be crucial in bridging the gap between these approaches and expanding their application in diagnostics and personalized medicine. Building on these insights, the next section delves into the methodologies employed in BuEV and SiEV analysis, with a focus on state-of-the-art techniques. It also explores recent advancements in EV research, illustrating how the field is evolving and how innovative technologies are shaping the future of EV-based diagnostics.

**Table 5:** Comparison of the Advantages and Limitations of BuEV and SiEV Analysis Technologies

aspect	BuEV analysis	SiEV analysis
sensitivity	Pros	Pros
	higher sensitivity for abundant biomarkers across many vesicles	high sensitivity for rare or low-abundance biomarkers on individual EVs.
	enhanced signal from aggregated EV populations	ideal for detecting subtle biomarker changes in early disease stages
	Cons	Cons
	misses rare biomarkers expressed on small subsets of EVs	detection may be limited for extremely low-abundance biomarkers
	aggregated data can mask heterogeneous populations	higher background noise from low signals
molecular profiling and specificity	Pros	Pros
	broad molecular profiling of EVs, useful for general disease identification	high specificity for individual EVs, enabling precise characterization of rare subpopulations
		ability to identify disease-specific EVs with rare or unique biomarker combinations
	Cons	Cons
	masking of EV heterogeneity due to averaging across the population	limited by current technologies for detecting multiple biomarkers on SiEVs
	difficult to detect disease-specific or rare EV subpopulations	
sample requirements	Pros	Pros
	less demanding in terms of sample volume and processing time	requires smaller EV sample volumes while maintaining high sensitivity for detecting low-abundance biomarkers
	suitable for clinical use with typical blood or plasma samples	provides more detailed data from smaller EV subpopulations, useful for targeted analysis
	Cons	Cons
	loss of rare EV subpopulations during isolation or processing	requires higher amounts of EVs isolated with high purity, which can be challenging in clinical settings
throughput and scalability	Pros	Pros
	high throughput, suitable for large-scale screening (e.g., population studies)	N/A
	easily integrates into routine clinical laboratories	
	Cons	Cons
	N/A	low throughput, more time-consuming, and more labor-intensive than BuEV analysis
		not yet scalable for large patient cohorts or mass screening
data complexity and interpretation	Pros	Pros
	easier to interpret with aggregated data across EV populations	can provide detailed molecular insights into disease heterogeneity and progression
	simple workflow, suitable for routine clinical diagnostics	
	Cons	Cons
	N/A	high complexity due to individual EV profiling
		requires advanced computational tools and expert knowledge for data analysis
cost and accessibility	Pros	Pros
	more cost-effective for clinical laboratories due to simpler protocols and less expensive equipment	N/A
	utilizes well-established and commercially available methods like ELISA, flow cytometry, and Western blotting	
	Cons	Cons
	N/A	emerging technologies for SiEV analysis are still developing and may have limitations in sensitivity or throughput
		expensive, requiring specialized equipment and expertise
		limited infrastructure for routine clinical use
clinical use cases	Pros	Pros
	well-suited for broad disease screening	best for early detection of diseases and personalized medicine
	ideal for monitoring disease progression and therapeutic efficacy in later-stage conditions	crucial for understanding complex diseases with high molecular heterogeneity
	Cons	Cons
	less effective for early stage detection or rare diseases	not yet widely used for mass screening
	may overlook subtle changes in disease biomarkers	may be too slow and costly for large-scale applications

## 8 Conclusions

The integration of BuEVs and SiEVs in clinical diagnostics is reshaping our understanding of disease biology and biomarker discovery for noninvasive diagnostic methods. EVs, with their diverse cargo of nucleic acids, proteins, and lipids, reflect the physiological and pathological states of their cells of origin, making them powerful tools for diagnostics. Recent advancements in integrated isolation technologies, particularly the combination of immunoaffinity capture with nanomaterials and microfluidic systems, have significantly improved the sensitivity and specificity of downstream EV detection in complex biological samples. These innovations now allow reliable detection of EVs at low concentrations, a critical factor for clinical applications.

A major breakthrough in EV research is the ability to differentiate between various EV subtypes, offering a refined understanding of EV heterogeneity and the distinct roles these vesicles play in different diseases. Since EVs originate from specific cellular processes and carry unique molecular signatures, they likely contribute to disease progression in distinct ways. Advanced analytical methods, such as single-particle tracking microscopy, single-vesicle RNA sequencing, nanopore-based assays, nanoplasmonic techniques, immuno-digital droplet platforms, and microfluidic systems integrated with nanomaterials, are now capable of dissecting the complex cargo within SiEVs. These tools provide unprecedented insights into the molecular profiles of disease-associated EVs, unlocking new possibilities for precision diagnostics.

EV analysis from biological fluids, such as blood, and urine has emerged as a powerful tool for noninvasive diagnostics, particularly in the development of noninvasive liquid biopsies. This approach is poised to contributing to disease detection, monitor progression, and inform personalized treatment strategies, especially in oncology, cardiovascular diseases, and neurodegenerative disorders. As the field advances, EV-based diagnostics are expected to complement existing clinical tools, enriching disease-specific biomarker discovery when combined with high-throughput sequencing, proteomics, and imaging technologies. The integration of these methods will lead to more comprehensive and precise characterizations of disease states, improving diagnostic accuracy and patient stratification. Furthermore, the development of point-of-care devices capable of real-time EV analysis could transform clinical practice by enabling rapid, on-site diagnostics.

The shift from BuEV measurement to SiEV analysis represents an important development in clinical diagnostics. Despite ongoing challenges related to standardization, cost, scalability, and data interpretation, the potential benefits for precision medicine and early disease detection are profound. Continued research, technological innovation, and collaboration across disciplines will be essential to fully realize the promise of EV-based diagnostics.

## 9 Perspective and Future Directions

### 9.1 Perspective

The field of EV research has experienced a significant shift, particularly with the move from BuEV analysis to the more precise examination of SiEVs. This evolution has opened new avenues for clinical diagnostics, offering the potential to detect and characterize disease biomarkers with unprecedented sensitivity. SiEV analysis provides a level of molecular granularity that BuEV analysis cannot match, uncovering heterogeneity within EV populations. Furthermore, integrating SiEV analysis with multiomic technologies such as proteomics, genomics, and transcriptomics offers the potential for comprehensive disease profiling. The detailed profiling of SiEVs can differentiate between disease stages and reveal unique molecular signatures associated with specific conditions. This precision is particularly important for diseases where diagnostic markers may be present in minimal amounts or for early diagnosis. The ability to isolate and analyze individual SiEVs allows for the identification of rare biomarkers that might otherwise be overlooked, offering insights into disease mechanisms and enabling more personalized diagnostic approaches.

Despite these advancements, significant challenges persist in accurately identifying and differentiating EV subtypes. A key issue is the overlapping size of exosomes, microvesicles, and NEVs, complicating the classification of EVs. The lack of standardized methods for distinguishing these vesicle types impedes consistent analysis. Future research should focus on developing refined techniques for separating and identifying EV subtypes based on their unique biophysical and biochemical properties. Enhanced classification will deepen our understanding of the distinct functions and roles of these particles in disease and health.

Despite the advantages of SiEV analysis, several barriers must be addressed before it can be widely implemented in clinical practice. One main barrier is the lack of standardized protocols for EV isolation and characterization, which impedes reproducibility across laboratories. The complexity of biological systems, combined with the technical noise inherent in SiEV analysis, complicates the accurate interpretation of results. Developing robust validation methods is essential to ensure that SiEV data can be reliably interpreted and applied in diverse clinical settings. Advancements in computational tools, including ML algorithms, are helping to distinguish true biological signals from artifacts. These tools can enhance the reliability of SiEV-based diagnostics by refining data analysis and improving the accuracy of biomarker detection. Another main barrier to the widespread adoption of SiEV diagnostics is the high cost and technical expertise required for current SiEV analysis methods. The development of cost-effective, automated platforms is critical to making SiEV diagnostics more accessible. Innovations in nanotechnology, microfluidics, and single-molecule sensors are expected to play a key role in enhancing the efficiency of SiEV isolation and analysis while reducing costs. These advancements will allow for the rapid, high-throughput processing of clinical samples, making SiEV analysis a practical tool for routine diagnostics.

### 9.2 Future Research Directions

Looking ahead, significant effort must be dedicated to addressing the current obstacles in SiEV research and diagnostics. A critical challenge remains the establishment of universally accepted protocols for SiEV isolation, characterization, and data interpretation. Developing consensus guidelines will be essential to ensure reproducibility and reliability across different clinical settings and research institutions. Validation studies involving large, diverse patient populations will be needed to confirm the clinical utility of SiEV-based diagnostics. The current high costs of SiEV analysis hinder its integration into routine clinical practice. Future research should focus on developing affordable, automated platforms that can process numerous samples while maintaining sensitivity and specificity. Innovations in nanomaterials, coupled with advances in microfluidics, are expected to streamline SiEV isolation and reduce contamination from NEVs, making the technology more accessible.

The large data sets generated by SiEV analysis present both an opportunity and a challenge. The convergence of SiEV analysis with proteomic, genomic, and transcriptomic data holds great promise for creating comprehensive disease profiles. By integrating these data sets, researchers can gain deeper insights into disease mechanisms, identify novel biomarkers, and refine diagnostic strategies. Future advancements in bioinformatics and data integration tools will be crucial for managing the complexity of multiomic SiEV data sets and ensuring that meaningful biological insights are extracted. AI- and ML-driven tools will be essential for distinguishing meaningful patterns from background noise and for enhancing the precision of diagnostic predictions. Predictive models and decision-support systems, fueled by AI, could help understand how SiEV data is interpreted and applied in clinical settings.

A major focus of future research should be on discovering novel biomarkers within SiEVs, particularly those linked to underexplored diseases or conditions where early diagnosis is critical. SiEV analysis provides a unique opportunity to identify low-abundance molecular signatures that could lead to breakthroughs in understanding disease pathogenesis and developing targeted therapies. Research into miRNAs, lncRNAs, and specific proteins associated with disease progression will be particularly valuable.

As SiEV diagnostics move closer to clinical implementation, navigating regulatory challenges will become increasingly important. The establishment of clear guidelines for SiEV-based assays, including criteria for clinical validation and approval by regulatory agencies, will be essential for ensuring that these technologies can be safely and effectively deployed. Ethical considerations, such as patient consent and data privacy, must also be carefully addressed, particularly in the context of personalized medicine.

The future of SiEV analysis in clinical diagnostics is bright, with the potential to advance disease detection, treatment monitoring, and patient care. However, the path forward requires overcoming significant challenges related to standardization, scalability, and cost. By fostering interdisciplinary collaboration among biologists, engineers, clinicians, and data scientists, and by leveraging the latest advances in nanotechnology, bioinformatics, and AI, the full potential of EVs as diagnostic tools can be realized. As research continues to refine EV isolation and analysis technologies, clinical validation studies will be required to confirm their efficacy. Because of the enhanced precision of SiEV analysis, SiEV-based diagnostics will likely become an integral part of personalized healthcare, offering more precise and timely insights into a wide range of diseases.

## Vocabulary

Extracellular vesicles: Extracellular vesicles are nanosized membrane-enclosed vesicles released by all cells that carry an array of biomolecules.
Bulk extracellular vesicles: Populations of extracellular vesicles.
Single extracellular vesicle analysis: In-depth understanding of the physical properties, molecular compositions, and biological roles of EVs at the individual vesicle level.
Heterogeneity: Heterogeneity refers to variability in the biogenesis, biophysical characteristics, composition, and role of EVs.
Extracellular vesicle biomarkers: Extracellular vesicle biomarkers are specific molecules found within or associated with extracellular vesicles, including proteins, lipids, and nucleic acids (such as RNA), that indicate the physiological or pathological status of the parent cells.

## References

[1] Mukherjee A., Bisht B., Dutta S., Paul M. K. (2022). Current Advances
in the Use of Exosomes, Liposomes, and Bioengineered Hybrid Nanovesicles
in Cancer Detection and Therapy. Acta Pharmacologica
Sinica.

[2] Liu Y. J., Wang C. (2023). A Review of
the Regulatory Mechanisms of Extracellular Vesicles-Mediated
Intercellular Communication. Cell Commun. Signal..

[3] Sousa
de Almeida M., Susnik E., Drasler B., Taladriz-Blanco P., Petri-Fink A., Rothen-Rutishauser B. (2021). Understanding Nanoparticle Endocytosis
to Improve Targeting Strategies in Nanomedicine. Chem. Soc. Rev..

[4] Cloet T., Momenbeitollahi N., Li H. (2021). Recent Advances on Protein-Based
Quantification of Extracellular Vesicles. Anal.
Biochem..

[5] van
Niel G., Carter D. R., Clayton A., Lambert D. W., Raposo G., Vader P. (2022). Challenges and Directions in Studying Cell-Cell Communication by
Extracellular Vesicles. Nat. Rev. Mol. Cell
Biol..

[6] O’Brien K., Ughetto S., Mahjoum S., Nair A. V., Breakefield X. O. (2022). Uptake,
Functionality, and Re-Release of Extracellular Vesicle-Encapsulated
Cargo. Cell Rep.

[7] Onkar A., Khan F., Goenka A., Rajendran R. L., Dmello C., Hong C. M., Mubin N., Gangadaran P., Ahn B.-C. (2024). Smart Nanoscale Extracellular Vesicles
in the Brain:
Unveiling Their Biology, Diagnostic Potential, and Therapeutic Applications. ACS Appl. Mater. Interfaces.

[8] Amrollahi P., Zheng W., Monk C., Li C.-Z., Hu T. Y. (2021). Nanoplasmonic
Sensor Approaches for Sensitive Detection of Disease-Associated Exosomes. ACS applied bio materials.

[9] Huang G., Zheng W., Zhou Y., Wan M., Hu T. (2024). Recent Advances
to Address Challenges in Extracellular Vesicle-Based Applications
for Lung Cancer. Acta Pharm. Sin B.

[10] Buzas E. I. (2023). The Roles
of Extracellular Vesicles in the Immune System. Nature Reviews Immunology.

[11] Gill S., Catchpole R., Forterre P. (2019). Extracellular Membrane
Vesicles in
the Three Domains of Life and Beyond. FEMS microbiology
reviews.

[12] Huda M. N., Nafiujjaman M., Deaguero I. G., Okonkwo J., Hill M. L., Kim T., Nurunnabi M. (2021). Potential Use of Exosomes as Diagnostic Biomarkers
and in Targeted Drug Delivery: Progress in Clinical and Preclinical
Applications. ACS Biomaterials Science &
Engineering.

[13] Qian F., Huang Z., Zhong H., Lei Q., Ai Y., Xie Z., Zhang T., Jiang B., Zhu W., Sheng Y. (2022). Analysis and Biomedical Applications of Functional Cargo in Extracellular
Vesicles. ACS Nano.

[14] Jiang L., Gu Y., Du Y., Liu J. (2019). Exosomes: Diagnostic Biomarkers and
Therapeutic Delivery Vehicles for Cancer. Mol.
Pharmaceutics.

[15] Zhao Z., Fan J., Hsu Y.-M. S., Lyon C. J., Ning B., Hu T. Y. (2019). Extracellular
Vesicles as Cancer Liquid Biopsies: From Discovery, Validation, to
Clinical Application. Lab Chip.

[16] Gurunathan S., Kang M.-H., Qasim M., Khan K., Kim J.-H. (2021). Biogenesis,
Membrane Trafficking, Functions, and Next Generation Nanotherapeutics
Medicine of Extracellular Vesicles. Int. J.
Nanomed..

[17] Mahmoudi F., Hanachi P., Montaseri A. (2023). Extracellular Vesicles of Immune
Cells; Immunomodulatory Impacts and Therapeutic Potentials. Clinical Immunology.

[18] Toyofuku M., Schild S., Kaparakis-Liaskos M., Eberl L. (2023). Composition and Functions
of Bacterial Membrane Vesicles. Nat. Rev. Microbiol..

[19] Yang E., Wang X., Gong Z., Yu M., Wu H., Zhang D. (2020). Exosome-Mediated Metabolic Reprogramming:
The Emerging Role in Tumor
Microenvironment Remodeling and Its Influence on Cancer Progression. Signal Transduction Targeted Ther..

[20] Chiang C. y., Chen C. (2019). Toward Characterizing
Extracellular Vesicles at a Single-Particle
Level. J. Biomed. Sci..

[21] Ortmann W., Such A., Cichon I., Baj-Krzyworzeka M., Weglarczyk K., Kolaczkowska E. (2024). Large Extracellular
Vesicle (Ev)
and Neutrophil Extracellular Trap (Net) Interaction Captured in Vivo
During Systemic Inflammation. Sci. Rep..

[22] Kalluri R., LeBleu V. S. (2020). The Biology, Function, and Biomedical
Applications
of Exosomes. Science.

[23] Mercier V., Larios J., Molinard G., Goujon A., Matile S., Gruenberg J., Roux A. (2020). Endosomal Membrane Tension Regulates
Escrt-Iii-Dependent Intra-Lumenal Vesicle Formation. Nature cell biology.

[24] Vietri M., Radulovic M., Stenmark H. (2020). The Many Functions
of Escrts. Nat. Rev. Mol. Cell Biol..

[25] Jin Y., Ma L., Zhang W., Yang W., Feng Q., Wang H. (2022). Extracellular
Signals Regulate the Biogenesis of Extracellular Vesicles. Biol. Res..

[26] Andreu Z., Yáñez-Mó M. (2014). Tetraspanins
in Extracellular Vesicle
Formation and Function. Front. Immunol..

[27] Sapoń K., Mańka R., Janas T., Janas T. (2023). The Role of
Lipid Rafts
in Vesicle Formation. J. Cell Sci..

[28] Sehrawat T. S., Arab J. P., Liu M., Amrollahi P., Wan M., Fan J., Nakao Y., Pose E., Navarro-Corcuera A., Dasgupta D. (2021). Circulating Extracellular Vesicles Carrying
Sphingolipid Cargo for the Diagnosis and Dynamic Risk Profiling of
Alcoholic Hepatitis. Hepatology.

[29] D’Souza-Schorey C., Chavrier P. (2006). Arf Proteins:
Roles in Membrane Traffic and Beyond. Nat. Rev.
Mol. Cell Biol..

[30] van
de Wakker S. I., Meijers F. M., Sluijter J. P. G., Vader P. (2023). Extracellular
Vesicle Heterogeneity and Its Impact for Regenerative Medicine Applications. Pharmacol. Rev..

[31] Xie S., Zhang Q., Jiang L. (2022). Current Knowledge on Exosome Biogenesis,
Cargo-Sorting Mechanism and Therapeutic Implications. Membranes.

[32] Hánělová K., Raudenská M., Masařík M., Balvan J. (2024). Protein Cargo
in Extracellular Vesicles as the Key Mediator in the Progression of
Cancer. Cell Commun. Signal..

[33] Bordanaba-Florit G., Royo F., Kruglik S. G., Falcon-Perez J. M. (2021). Using Single-Vesicle
Technologies to Unravel the Heterogeneity of Extracellular Vesicles. Nat. Protoc..

[34] Hilton S. H., White I. M. (2021). Advances in the Analysis of Single
Extracellular Vesicles:
A Critical Review. Sensors and actuators reports.

[35] Goswami B., Nag S., Ray P. S. (2024). Fates and
Functions of Rna-Binding Proteins under Stress. Wiley Interdiscip. Rev.: RNA.

[36] D’Acunzo P., Pérez-González R., Kim Y., Hargash T., Miller C., Alldred M., Erdjument-Bromage H., Penikalapati S., Pawlik M., Saito M. (2021). Mitovesicles Are a
Novel Population of Extracellular Vesicles of Mitochondrial Origin
Altered in Down Syndrome. Sci. Adv..

[37] Jeppesen D. K., Zhang Q., Franklin J. L., Coffey R. J. (2023). Extracellular Vesicles
and Nanoparticles: Emerging Complexities. Trends
in Cell Biology.

[38] Zhang H., Freitas D., Kim H. S., Fabijanic K., Li Z., Chen H., Mark M. T., Molina H., Martin A. B., Bojmar L. (2018). Identification of Distinct Nanoparticles and Subsets
of Extracellular Vesicles by Asymmetric Flow Field-Flow Fractionation. Nature cell biology.

[39] Hoshino A., Kim H. S., Bojmar L., Gyan K. E., Cioffi M., Hernandez J., Zambirinis C. P., Rodrigues G., Molina H., Heissel S. (2020). Extracellular Vesicle
and Particle
Biomarkers Define Multiple Human Cancers. Cell.

[40] Jeppesen D. K., Zhang Q., Franklin J. L., Coffey R. J. (2022). Are Supermeres
a
Distinct Nanoparticle?. J. Extracellular Biol..

[41] Zhou X., Xie F., Wang L., Zhang L., Zhang S., Fang M., Zhou F. (2020). The Function and Clinical Application of Extracellular Vesicles in
Innate Immune Regulation. Cellular & molecular
immunology.

[42] Lengel H. B., Mastrogiacomo B., Connolly J. G., Tan K. S., Liu Y., Fick C. N., Dunne E. G., He D., Lankadasari M. B., Satravada B. A. (2023). Genomic Mapping of Metastatic Organotropism in Lung
Adenocarcinoma. Cancer Cell.

[43] Suo M., Fu Y., Wang S., Lin S., Zhang J., Wu C., Yin H., Wang P., Zhang W., Wang X. H. (2024). Miniaturized
Laser
Probe for Exosome-Based Cancer Liquid Biopsy. Anal. Chem..

[44] Kapoor K. S., Kong S., Sugimoto H., Guo W., Boominathan V., Chen Y.-L., Biswal S. L., Terlier T., McAndrews K. M., Kalluri R. (2024). Single Extracellular Vesicle Imaging and Computational
Analysis Identifies Inherent Architectural Heterogeneity. ACS Nano.

[45] Abdel
Halim A. S., Rudayni H. A., Chaudhary A. A., Ali M. A. (2023). Micrornas: Small Molecules with Big Impacts in Liver
Injury. Journal of Cellular Physiology.

[46] Ramos A. P., Sebinelli H. G., Ciancaglini P., Rosato N., Mebarek S., Buchet R., Millán J. L., Bottini M. (2022). The Functional Role
of Soluble Proteins Acquired by Extracellular Vesicles. J. Extracell. Biol..

[47] Kalluri R., McAndrews K. M. (2023). The Role
of Extracellular Vesicles in Cancer. Cell.

[48] de
Winde C. M., Veenbergen S., Young K. H., Xu-Monette Z. Y., Wang X. X., Xia Y., Jabbar K. J., van den
Brand M., van der Schaaf A., Elfrink S. (2016). Tetraspanin
Cd37 Protects against the Development of B Cell Lymphoma. J. Clin Invest.

[49] Yoshimura T., Miyoshi H., Shimono J., Nakashima K., Takeuchi M., Yanagida E., Yamada K., Shimasaki Y., Moritsubo M., Furuta T. (2022). Cd37 Expression in Follicular
Lymphoma. Ann. Hematol.

[50] Jeremy E., Artiga E., Elgamal S., Cheney C., Eicher D., Zalponik K., Orwick S., Mao C., Wasmuth R., Harrington B. (2025). Cd37 in Acute Myeloid
Leukemia: A Novel Surface
Target for Drug Delivery. Blood Adv..

[51] Falih
Soliman N., Jasim Mohamad B. (2022). The Impact of Cd37 Ectoenzyme Expression
in Benign and Malignant Colorectal Tumors. Arch.
Razi Inst..

[52] Thakur A., Xu C., Li W. K., Qiu G., He B., Ng S.-P., Wu C.-M. L., Lee Y. (2021). In Vivo Liquid
Biopsy for Glioblastoma
Malignancy by the Afm and Lspr Based Sensing of Exosomal Cd44 and
Cd133 in a Mouse Model. Biosens. Bioelectron..

[53] Zhang L., Yang P., Chen J., Chen Z., Liu Z., Feng G., Sha F., Li Z., Xu Z., Huang Y. (2023). Cd44 Connects Autophagy Decline and
Ageing in the Vascular Endothelium. Nat. Commun..

[54] Mustonen A. M., Capra J., Rilla K., Lehenkari P., Oikari S., Kääriäinen T., Joukainen A., Kröger H., Paakkonen T., Matilainen J., Nieminen P. (2021). Characterization of
Hyaluronan-Coated Extracellular Vesicles in Synovial Fluid of Patients
with Osteoarthritis and Rheumatoid Arthritis. BMC Musculoskeletal Disord..

[55] Fu M., Gao Q., Xiao M., Li R. F., Sun X. Y., Li S. L., Peng X., Ge X. Y. (2024). Extracellular Vesicles Containing
Circmybl1 Induce Cd44 in Adenoid Cystic Carcinoma Cells and Pulmonary
Endothelial Cells to Promote Lung Metastasis. Cancer Res..

[56] Kang K. W., Kim H., Hur W., Jung J. H., Jeong S. J., Shin H., Seo D., Jeong H., Choi B., Hong S. (2021). A Proteomic
Approach to Understand the Clinical Significance of Acute Myeloid
Leukemia-Derived Extracellular Vesicles Reflecting Essential Characteristics
of Leukemia. Mol. Cell Proteomics.

[57] Xing L., Wang Z., Feng Y., Luo H., Dai G., Sang L., Zhang C., Qian J. (2024). The Biological
Roles
of Cd47 in Ovarian Cancer Progression. Cancer
Immunol. Immunother..

[58] Tan M., Zhu L., Zhuang H., Hao Y., Gao S., Liu S., Liu Q., Liu D., Liu J., Lin B. (2015). Lewis Y Antigen Modified
Cd47 Is an Independent Risk Factor for Poor Prognosis and Promotes
Early Ovarian Cancer Metastasis. Am. J. Cancer
Res..

[59] Higgins C.
B., Adams J. A., Ward M. H., Greenberg Z. J., Milewska M., Sun J., Zhang Y., Chiquetto
Paracatu L., Dong Q., Ballentine S. (2023). The Tetraspanin Transmembrane Protein Cd53 Mediates Dyslipidemia
and Integrates Inflammatory and Metabolic Signaling in Hepatocytes. J. Biol. Chem..

[60] Nie P., Zhang W., Meng Y., Lin M., Guo F., Zhang H., Tong Z., Wang M., Chen F., An L. (2023). A Yap/Taz-Cd54 Axis Is Required for Cxcr2-Cd44- Tumor-Specific Neutrophils
to Suppress Gastric Cancer. Protein Cell.

[61] Li X., Wang Q., Ding L., Wang Y. X., Zhao Z. D., Mao N., Wu C. T., Wang H., Zhu H., Ning S. B. (2019). Intercellular
Adhesion Molecule-1 Enhances the Therapeutic Effects of Mscs in a
Dextran Sulfate Sodium-Induced Colitis Models by Promoting Mscs Homing
to Murine Colons and Spleens. Stem Cell Res.
Ther..

[62] Vorobjova T., Metsküla K., Salumäe L., Uibo O., Heilman K., Uibo R. (2025). Immunohistochemical
Evaluation of Lgr5, Cd71, Cd138 and Cxcr3Markers
in the Small Bowel Mucosa of Participants with Celiac Disease and
Persons with Normal Bowel Mucosa. J. Mol. Histol..

[63] Villalobos-Manzo R., Ríos-Castro E., Hernández-Hernández J. M., Oza G., Medina M. A., Tapia-Ramírez J. (2022). Identification of Transferrin
Receptor 1 (Tfr1) Overexpressed in Lung Cancer Cells, and Internalization
of Magnetic Au-Cofe(2)­O(4) Core-Shell Nanoparticles Functionalized
with Its Ligand in a Cellular Model of Small Cell Lung Cancer (Sclc). Pharmaceutics.

[64] Gualdrón-López M., Díaz-Varela M., Toda H., Aparici-Herraiz I., Pedró-Cos L., Lauzurica R., Lacerda M. V. G., Fernández-Sanmartín M. A., Fernandez-Becerra C., Del Portillo H. A. (2021). Multiparameter Flow Cytometry Analysis
of the Human Spleen Applied to Studies of Plasma-Derived Evs from
Plasmodium Vivax Patients. Front Cell Infect
Microbiol.

[65] Tertel T., Tomić S., Đokić J., Radojević D., Stevanović D., Ilić N., Giebel B., Kosanović M. (2022). Serum-Derived
Extracellular Vesicles: Novel Biomarkers Reflecting the Disease Severity
of Covid-19 Patients. J. Extracell. Vesicles.

[66] Ma X., He X., Wang C., Huang X., Li Y., Ma K. (2021). Small Extracellular
Ring Domain Is Necessary for Cd82/Kai1’anti-Metastasis Function. Biochem. Biophys. Res. Commun..

[67] Li S., Li X., Yang S., Pi H., Li Z., Yao P., Zhang Q., Wang Q., Shen P., Li X. (2021). Proteomic Landscape
of Exosomes Reveals the Functional Contributions
of Cd151 in Triple-Negative Breast Cancer. Mol.
Cell Proteomics.

[68] Wong A. H. P., Nga M. E., Chin C. Y., Tai Y. K., Wong H. C., Soo R., An O., Yang H., Seet J. E., Lim Y. C. (2024). Impact
of Cd151 Overexpression on Prognosis and Therapy in Non-Small Cell
Lung Cancer Patients Lacking Egfr Mutations. Cell Proliferation.

[69] Deng Y., Cai S., Shen J., Peng H. (2021). Tetraspanins: Novel Molecular Regulators
of Gastric Cancer. Front Oncol.

[70] Zhang C., Du F. h., Wang R. x., Han W. b., Lv X., Chen G. q. (2024). Tspan6 Reinforces
the Malignant Progression of Glioblastoma
Via Interacting with Cdk5rap3 and Regulating Stat3 Signaling Pathway. Int. J. Biol. Sci..

[71] Dash S., Wu C. C., Wu C. C., Chiang S. F., Lu Y. T., Yeh C. Y., You J. F., Chu L. J., Yeh T. S., Yu J. S. (2022). Extracellular Vesicle
Membrane Protein Profiling and Targeted Mass
Spectrometry Unveil Cd59 and Tetraspanin 9 as Novel Plasma Biomarkers
for Detection of Colorectal Cancer. Cancers
(Basel).

[72] Fang Z., Bai J. (2024). Integrated Bioinformatics Analysis
Reveals the Bidirectional Effects
of Tspan6 for Cisplatin Resistance in Lung Cancer. Chem. Biol. Drug Des..

[73] Pang S., Luo Z., Dong W., Gao S., Chen W., Liu N., Zhang X., Gao X., Li J., Gao K. (2023). Integrin Β1/Fak/Src Signal Pathway Is
Involved in Autism Spectrum
Disorder in Tspan7 Knockout Rats. Life Sci.
Alliance.

[74] McLaughlin K. A., Tombs M. A., Christie M. R. (2020). Autoimmunity
to Tetraspanin-7 in
Type 1 Diabetes. Med. Microbiol Immunol.

[75] Tognoli M. L., Dancourt J., Bonsergent E., Palmulli R., de Jong O. G., Van Niel G., Rubinstein E., Vader P., Lavieu G. (2023). Lack of Involvement
of Cd63 and Cd9 Tetraspanins in the Extracellular Vesicle Content
Delivery Process. Commun. Biol..

[76] Silva A. M., Lázaro-Ibáñez E., Gunnarsson A., Dhande A., Daaboul G., Peacock B., Osteikoetxea X., Salmond N., Friis K. P., Shatnyeva O., Dekker N. (2021). Quantification of Protein Cargo Loading into Engineered
Extracellular Vesicles at Single-Vesicle and Single-Molecule Resolution. J. Extracell. Vesicles.

[77] Dai Y., Wang Y., Cao Y., Yu P., Zhang L., Liu Z., Ping Y., Wang D., Zhang G., Sang Y. (2021). A Multivariate Diagnostic
Model Based on Urinary Epcam-Cd9-Positive
Extracellular Vesicles for Prostate Cancer Diagnosis. Front Oncol.

[78] Zhyvolozhnyi A., Samoylenko A., Bart G., Kaisanlahti A., Hekkala J., Makieieva O., Pratiwi F., Miinalainen I., Kaakinen M., Bergman U. (2024). Enrichment of Sweat-Derived
Extracellular Vesicles of Human and Bacterial Origin for Biomarker
Identification. Nanotheranostics.

[79] Franco C., Ghirardello A., Bertazza L., Gasparotto M., Zanatta E., Iaccarino L., Valadi H., Doria A., Gatto M. (2023). Size-Exclusion Chromatography
Combined with Ultrafiltration Efficiently
Isolates Extracellular Vesicles from Human Blood Samples in Health
and Disease. Int. J. Mol. Sci..

[80] Morasso C., Ricciardi A., Sproviero D., Truffi M., Albasini S., Piccotti F., Sottotetti F., Mollica L., Cereda C., Sorrentino L. (2022). Fast Quantification of Extracellular Vesicles
Levels in Early Breast Cancer Patients by Single Molecule Detection
Array (Simoa). Breast Cancer Research and Treatment.

[81] Sun R., Cai Y., Zhou Y., Bai G., Zhu A., Kong P., Sun J., Li Y., Liu Y., Liao W., Liu J., Cui N., Xiang J., Li B., Zhao J., Wu D., Ran P. (2023). Proteomic Profiling
of Single Extracellular Vesicles Reveals Colocalization
of Sars-Cov-2 with a Cd81/Integrin-Rich Ev Subpopulation in Sputum
from Covid-19 Severe Patients. Front. Immunol..

[82] von
Lersner A. K., Fernandes F., Ozawa P. M. M., Jackson M., Masureel M., Ho H., Lima S. M., Vagner T., Sung B. H., Wehbe M. (2024). Multiparametric Single-Vesicle
Flow Cytometry Resolves Extracellular Vesicle Heterogeneity and Reveals
Selective Regulation of Biogenesis and Cargo Distribution. ACS Nano.

[83] Ji C., Zhang J., Shi L., Shi H., Xu W., Jin J., Qian H. (2024). Engineered Extracellular Vesicle-Encapsulated Chip
as Novel Nanotherapeutics for Treatment of Renal Fibrosis. NPJ Regener. Med..

[84] Xu X., Xie T., Zhou M., Sun Y., Wang F., Tian Y., Chen Z., Xie Y., Wu R., Cen X. (2024). Hsc70 Promotes Anti-Tumor Immunity by Targeting
Pd-L1 for Lysosomal
Degradation. Nat. Commun..

[85] Zhang R., Liang X., Tang S., Song L., Zhang J., Du Y. (2021). Short-Term High-Intensity Treadmill Exercise Promotes Ceramide-Dependent
Extracellular Vesicle Secretion in the Central Nervous System of Mice. Med. Sci. Monit..

[86] Tan W., Zhang J., Liu L., Liang M., Li J., Deng Z., Zheng Z., Deng Y., Liu C., Li Y. (2022). Hsp90
Inhibitor Sta9090 Induced Vps35 Related Extracellular
Vesicle Release and Metastasis in Hepatocellular Carcinoma. Transl Oncol.

[87] Ono K., Sogawa C., Kawai H., Tran M. T., Taha E. A., Lu Y., Oo M. W., Okusha Y., Okamura H., Ibaragi S., Takigawa M., Kozaki K., Nagatsuka H., Sasaki A., Okamoto K., Calderwood S. K., Eguchi T. (2020). Triple Knockdown of Cdc37, Hsp90-Alpha and Hsp90-Beta
Diminishes Extracellular Vesicles-Driven Malignancy Events and Macrophage
M2 Polarization in Oral Cancer. J. Extracell.
Vesicles.

[88] Yamamoto K., Venida A., Yano J., Biancur D. E., Kakiuchi M., Gupta S., Sohn A. S. W., Mukhopadhyay S., Lin E. Y., Parker S. J. (2020). Autophagy
Promotes Immune
Evasion of Pancreatic Cancer by Degrading Mhc-I. Nature.

[89] Luo N., Nixon M. J., Gonzalez-Ericsson P.
I., Sanchez V., Opalenik S. R., Li H., Zahnow C. A., Nickels M. L., Liu F., Tantawy M. N., Sanders M. E., Manning H. C., Balko J. M. (2018). DNA Methyltransferase
Inhibition Upregulates Mhc-I to Potentiate Cytotoxic T Lymphocyte
Responses in Breast Cancer. Nat. Commun..

[90] Kim J. Y., Cha H., Kim K., Sung C., An J., Bang H., Kim H., Yang J. O., Chang S., Shin I. (2023). Mhc Ii
Immunogenicity Shapes the Neoepitope Landscape in Human Tumors. Nat. Genet..

[91] Baleeiro R. B., Bouwens C. J., Liu P., Di Gioia C., Dunmall L. S. C., Nagano A., Gangeswaran R., Chelala C., Kocher H. M., Lemoine N. R. (2022). Mhc Class Ii Molecules
on Pancreatic Cancer Cells Indicate
a Potential for Neo-Antigen-Based Immunotherapy. Oncoimmunology.

[92] Gu Y., Du Y., Jiang L., Tang X., Li A., Zhao Y., Lang Y., Liu X., Liu J. (2022). Αvβ3 Integrin-Specific
Exosomes Engineered with Cyclopeptide for Targeted Delivery of Triptolide
against Malignant Melanoma. J. Nanobiotechnol..

[93] Altei W. F., Pachane B. C., Dos Santos P. K., Ribeiro L. N., Sung B. H., Weaver A. M., Selistre-de-Araújo H. S. (2020). Inhibition
of Αvβ3
Integrin Impairs Adhesion and Uptake of Tumor-Derived Small Extracellular
Vesicles. Cell Commun. Signal..

[94] Moskovitch O., Anaki A., Caller T., Gilburd B., Segal O., Gendelman O., Watad A., Mehrian-Shai R., Mintz Y., Segev S. (2025). The Potential of Autologous Patient-Derived
Circulating Extracellular Vesicles to Improve Drug Delivery in Rheumatoid
Arthritis. Clin. Exp. Immunol..

[95] Chanda D., Otoupalova E., Hough K. P., Locy M. L., Bernard K., Deshane J. S., Sanderson R. D., Mobley J. A., Thannickal V. J. (2019). Fibronectin
on the Surface of Extracellular Vesicles Mediates Fibroblast Invasion. Am. J. Respir. Cell Mol. Biol..

[96] Zhao X., Mai Z., Liu L., Lu Y., Cui L., Yu J. (2024). Hypoxia-Driven
Tns4 Fosters Hnscc Tumorigenesis by Stabilizing Integrin Α5β1
Complex and Triggering Fak-Mediated Akt and Tgfβ Signaling Pathways. International Journal of Biological Sciences.

[97] Peng Z., Hao M., Tong H., Yang H., Huang B., Zhang Z., Luo K. Q. (2022). The Interactions
between Integrin Α(5)­Β(1)
of Liver Cancer Cells and Fibronectin of Fibroblasts Promote Tumor
Growth and Angiogenesis. Int. J. Biol. Sci..

[98] Cheng Y. Q., Yue Y. X., Cao H. M., Geng W. C., Wang L. X., Hu X. Y., Li H. B., Bian Q., Kong X. L., Liu J. F. (2021). Coassembly of Hypoxia-Sensitive
Macrocyclic Amphiphiles
and Extracellular Vesicles for Targeted Kidney Injury Imaging and
Therapy. J. Nanobiotechnol..

[99] He J., Wu X., Li L., Chen J., Liao J., Wu A., Zhang M., Chen Y., Mao X., Shen X. (2024). Construction
of Curcumin-Loaded Macrophage and Huvecs Membrane-Derived
Vesicles for Drug Delivery in Cardiovascular Inflammatory. Journal of Drug Delivery Science and Technology.

[100] Mu W., Xu Y., Gu P., Wang W., Li J., Ge Y., Wang H. (2021). Exosomal Cd44
Cooperates with Integrin Α6β4
to Support Organotropic Metastasis Via Regulating Tumor Cell Motility
and Target Host Cell Activation. Engineering.

[101] Hoshino A., Costa-Silva B., Shen T.-L., Rodrigues G., Hashimoto A., Tesic Mark M., Molina H., Kohsaka S., Di Giannatale A., Ceder S. (2015). Tumour Exosome Integrins
Determine Organotropic Metastasis. Nature.

[102] Petővári G., Tóth G., Turiák L., A L. K., Pálóczi K., Sebestyén A., Pesti A., Kiss A., Baghy K., Dezső K. (2023). Dynamic Interplay in Tumor Ecosystems: Communication
between Hepatoma Cells and Fibroblasts. Int.
J. Mol. Sci..

[103] Hayward S., Gachehiladze M., Badr N., Andrijes R., Molostvov G., Paniushkina L., Sopikova B., Slobodová Z., Mgebrishvili G., Sharma N. (2020). The Cd151-Midkine Pathway
Regulates the Immune Microenvironment in Inflammatory Breast Cancer. J. Pathol.

[104] Wang S., Zhang Q., Tiwari S. K., Lichinchi G., Yau E. H., Hui H., Li W., Furnari F., Rana T. M. (2020). Integrin Αvβ5 Internalizes Zika Virus During
Neural Stem Cells Infection and Provides a Promising Target for Antiviral
Therapy. Cell Rep..

[105] Wu R., Xie Y., Peng Y., Wu X., Ma Y., Lyu F.-J., Zheng Q., Deng Z. (2024). Young Human Plasma-Derived
Extracellular Vesicles Rescue and Reactivate Il-1β and Tnf-Α
Treated Chondrocytes. Exp. Cell Res..

[106] Ji L., Ruan H., Fu Y., Xiong S. (2024). A Study of Antigen
Selection by Extracellular Vesicles as Vaccine Candidates against
Mycobacterium Tuberculosis Infection. J. Med.
Microbiol..

[107] Wang J., Cao Z., Wang P., Zhang X., Tang J., He Y., Huang Z., Mao X., Shi S., Kou X. (2021). Apoptotic Extracellular Vesicles
Ameliorate Multiple
Myeloma by Restoring Fas-Mediated Apoptosis. ACS Nano.

[108] Moret-Tatay I., Nos P., Iborra M., Rausell F., Beltrán B. (2024). Catalase Inhibition
Can Modulate the Ability of Peripheral
Blood T Cells to Undergo Apoptosis in Crohn’s Disease. Clin. Exp. Immunol..

[109] Maniya N. H., Kumar S., Franklin J. L., Higginbotham J. N., Scott A. M., Gan H. K., Coffey R. J., Senapati S., Chang H.-C. (2024). An Anion Exchange Membrane Sensor Detects Egfr and
Its Activity State in Plasma Cd63 Extracellular Vesicles from Patients
with Glioblastoma. Commun. Biol..

[110] Jeong M. H., Son T., Tae Y. K., Park C. H., Lee H. S., Chung M. J., Park J. Y., Castro C. M., Weissleder R., Jo J. H. (2023). Plasmon-Enhanced Single Extracellular
Vesicle Analysis for Cholangiocarcinoma Diagnosis. Adv. Sci. (Weinh).

[111] Moon S., Kim S. I., Lee S., Lee H., Kim Y., Kim J. Y., Kim M. W., Kim J. Y. (2024). Potential Use of
Extracellular Vesicles for the Her2 Status Assessment in Breast Cancer
Patients. Genes, Chromosomes Cancer.

[112] Jiang N., Saftics A., Romano E., Ghaeli I., Resto C., Robles V., Das S., Van Keuren-Jensen K., Seewaldt V. L., Jovanovic-Talisman T. (2024). Multiparametric Profiling of Her2-Enriched
Extracellular Vesicles in Breast Cancer Using Single Extracellular
Vesicle Nanoscopy. J. Nanobiotechnol..

[113] Subsomwong P., Asano K., Akada J., Matsumoto T., Nakane A., Yamaoka Y. (2025). Proteomic Profiling of Extracellular
Vesicles Reveals Potential Biomarkers for Helicobacter Pylori Infection
and Gastric Cancer. Helicobacter.

[114] You Y., Zhang Z., Sultana N., Ericsson M., Martens Y. A., Sun M., Kanekiyo T., Ikezu S., Shaffer S. A., Ikezu T. (2023). Atp1a3 as
a Target for Isolating Neuron-Specific Extracellular Vesicles from
Human Brain and Biofluids. Sci. Adv..

[115] Zhang P., Crow J., Lella D., Zhou X., Samuel G., Godwin A. K., Zeng Y. (2018). Ultrasensitive Quantification
of Tumor Mrnas in Extracellular Vesicles with an Integrated Microfluidic
Digital Analysis Chip. Lab Chip.

[116] Dar G. H., Mendes C. C., Kuan W.-L., Speciale A. A., Conceição M., Görgens A., Uliyakina I., Lobo M. J., Lim W. F., El Andaloussi S. (2021). Gapdh Controls
Extracellular Vesicle Biogenesis and Enhances the Therapeutic Potential
of Ev Mediated Sirna Delivery to the Brain. Nat. Commun..

[117] Sharma H., Yadav V., Burchett A., Shi T., Senapati S., Datta M., Chang H. C. (2025). A Mem-Delisa Platform
for Dual Color and Ultrasensitive Digital Detection of Colocalized
Proteins on Extracellular Vesicles. Biosens
Bioelectron.

[118] Li H., Chiang C., Kwak K. J., Wang X., Doddi S., Ramanathan L. V., Cho S. M., Hou Y., Cheng T., Mo X., Chang Y., Chang H., Cheng W., Tsai W., Nguyen L. T. H., Pan J., Ma Y., Rima X. Y., Zhang J., Reategui E., Chu Y., Chang P. M., Chang P., Huang C. F., Wang C., Shan Y., Li C., Fleisher M., Lee L. J. (2024). Extracellular Vesicular Analysis
of Glypican 1 Mrna and Protein for Pancreatic Cancer Diagnosis and
Prognosis. Adv. Sci..

[119] Sinn B. V., von Minckwitz G., Denkert C., Eidtmann H., Darb-Esfahani S., Tesch H., Kronenwett R., Hoffmann G., Belau A., Thommsen C. (2013). Evaluation
of Mucin-1 Protein and Mrna Expression as Prognostic and Predictive
Markers after Neoadjuvant Chemotherapy for Breast Cancer. Ann. Oncol.

[120] Vidal M. J., Stahl P. D. (1993). The Small Gtp-Binding Proteins Rab4
and Arf Are Associated with Released Exosomes During Reticulocyte
Maturation. Eur. J. Cell Biol..

[121] Sung D. K., Sung S. I., Ahn S. Y., Chang Y. S., Park W. S. (2019). Thrombin Preconditioning Boosts Biogenesis
of Extracellular
Vesicles from Mesenchymal Stem Cells and Enriches Their Cargo Contents
Via Protease-Activated Receptor-Mediated Signaling Pathways. Int. J. Mol. Sci..

[122] Liang W., Sagar S., Ravindran R., Najor R. H., Quiles J. M., Chi L., Diao R. Y., Woodall B. P., Leon L. J., Zumaya E. (2023). Mitochondria Are Secreted
in Extracellular Vesicles When Lysosomal Function Is Impaired. Nat. Commun..

[123] Kim O., Hwangbo C., Tran P. T., Lee J.-H. (2022). Syntenin-1-Mediated
Small Extracellular Vesicles Promotes Cell Growth, Migration, and
Angiogenesis by Increasing Onco-Mirnas Secretion in Lung Cancer Cells. Cell Death Dis..

[124] Cone A. S., Hurwitz S. N., Lee G. S., Yuan X., Zhou Y., Li Y., Meckes D. G. (2020). Alix and
Syntenin-1
Direct Amyloid Precursor Protein Trafficking into Extracellular Vesicles. BMC Mol. Cell Biol..

[125] Brahmer A., Geiß C., Lygeraki A., Neuberger E., Tzaridis T., Nguyen T. T., Luessi F., Régnier-Vigouroux A., Hartmann G., Simon P. (2023). Assessment of Technical and Clinical
Utility of a Bead-Based Flow Cytometry Platform for Multiparametric
Phenotyping of Cns-Derived Extracellular Vesicles. Cell Commun. Signal..

[126] Wang Q., Yu C. (2020). Identification of Biomarkers
Associated
with Extracellular Vesicles Based on an Integrative Pan-Cancer Bioinformatics
Analysis. Med. Oncol..

[127] Mathieu M., Névo N., Jouve M., Valenzuela J. I., Maurin M., Verweij F. J., Palmulli R., Lankar D., Dingli F., Loew D. (2021). Specificities
of Exosome Versus Small
Ectosome Secretion Revealed by Live Intracellular Tracking of Cd63
and Cd9. Nat. Commun..

[128] Beatriz M., Vilaça R., Anjo S. I., Manadas B., Januário C., Rego A. C., Lopes C. (2022). Defective Mitochondria-Lysosomal
Axis Enhances the Release of Extracellular Vesicles Containing Mitochondrial
DNA and Proteins in Huntington’s Disease. J. Extracell. Biol..

[129] You Y., Muraoka S., Jedrychowski M. P., Hu J., McQuade A. K., Young-Pearse T., Aslebagh R., Shaffer S. A., Gygi S. P., Blurton-Jones M. (2022). Human Neural Cell Type-Specific
extracellular
Vesicle Proteome Defines Disease-Related Molecules Associated with
Activated Astrocytes in Alzheimer’s Disease Brain. J. Extracell. Vesicles.

[130] Fang Z., Fu J., Chen X. (2024). A Combined
Immune and
Exosome-Related Risk Signature as Prognostic Biomakers in Acute Myeloid
Leukemia. Hematology.

[131] Greenberg Z. J., Monlish D. A., Bartnett R. L., Yang Y., Shen G., Li W., Bednarski J. J., Schuettpelz L. G. (2020). The Tetraspanin Cd53 Regulates Early B Cell Development
by Promoting Il-7r Signaling. J. Immunol..

[132] Obeagu E., Obeagu G. (2024). The Role of L-Selectin
in Tuberculosis
and Hiv Coinfection: Implications for Disease Diagnosis and Management. Elite J. Public Health.

[133] Grange C., Tapparo M., Tritta S., Deregibus M. C., Battaglia A., Gontero P., Frea B., Camussi G. (2015). Role of Hla-G
and Extracellular Vesicles in Renal Cancer Stem Cell-Induced Inhibition
of Dendritic Cell Differentiation. BMC Cancer.

[134] Voss K., Sewell A. E., Krystofiak E. S., Gibson-Corley K. N., Young A. C., Basham J. H., Sugiura A., Arner E. N., Beavers W. N., Kunkle D. E. (2023). Elevated
Transferrin Receptor Impairs T Cell Metabolism and Function in Systemic
Lupus Erythematosus. Sci. Immunol..

[135] Daou Y., Falabrègue M., Pourzand C., Peyssonnaux C., Edeas M. (2022). Host and Microbiota Derived Extracellular Vesicles: Crucial Players
in Iron Homeostasis. Front Med. (Lausanne).

[136] Zhang Q., Steensma D. P., Yang J., Dong T., Wu M. X. (2019). Uncoupling of Cd71 Shedding with
Mitochondrial Clearance in Reticulocytes
in a Subset of Myelodysplastic Syndromes. Leukemia.

[137] Macrì S., Pavesi E., Crescitelli R., Aspesi A., Vizziello C., Botto C., Corti P., Quarello P., Notari P., Ramenghi U. (2015). Immunophenotypic
Profiling of Erythroid Progenitor-Derived Extracellular Vesicles in
Diamond-Blackfan Anaemia: A New Diagnostic Strategy. PLoS One.

[138] Huang C., Hays F. A., Tomasek J. J., Benyajati S., Zhang X. A. (2020). Tetraspanin Cd82 Interaction with Cholesterol Promotes
Extracellular Vesicle-Mediated Release of Ezrin to Inhibit Tumour
Cell Movement. J. Extracell. Vesicles.

[139] Malla R., Marni R., Chakraborty A. (2023). Exploring
the Role of Cd151 in the Tumor Immune Microenvironment: Therapeutic
and Clinical Perspectives. Biochimica et Biophysica
Acta (BBA)-Reviews on Cancer.

[140] Zhang H., Song Q., Shang K., Li Y., Jiang L., Yang L. (2024). Tspan Protein Family: Focusing on
the Occurrence, Progression, and Treatment of Cancer. Cell Death Discov..

[141] Humbert P. O., Pryjda T. Z., Pranjic B., Farrell A., Fujikura K., de Matos Simoes R., Karim R., Kozieradzki I., Cronin S. J., Neely G. G. (2022). Tspan6 Is a Suppressor
of Ras-Driven Cancer. Oncogene.

[142] Chen L., Liu H., Li Y., Lin X., Xia S., Wanggou S., Li X. (2023). Functional Characterization
of Tspan7
as a Novel Indicator for Immunotherapy in Glioma. Front. Immunol..

[143] Han C., Kang H., Yi J., Kang M., Lee H., Kwon Y., Jung J., Lee J., Park J. (2021). Single-Vesicle
Imaging and Co-Localization Analysis for Tetraspanin Profiling of
Individual Extracellular Vesicles. J. Extracell.
Vesicles.

[144] Larios J., Mercier V., Roux A., Gruenberg J. (2020). Alix-and Escrt-Iii–Dependent
Sorting of Tetraspanins to Exosomes. J. Cell
Biol..

[145] Adriaenssens E., Asselbergh B., Rivera-Mejías P., Bervoets S., Vendredy L., De Winter V., Spaas K., De Rycke R., Van Isterdael G., Impens F. (2023). Small Heat Shock Proteins Operate as Molecular Chaperones
in the Mitochondrial Intermembrane Space. Nat.
Cell Biol..

[146] Shan Q., Ma F., Wei J., Li H., Ma H., Sun P. (2020). Physiological
Functions of Heat Shock Proteins. Current Protein
and Peptide Science.

[147] Alberti G., Paladino L., Vitale A. M., Caruso
Bavisotto C., Conway de Macario E., Campanella C., Macario A. J., Marino Gammazza A. (2021). Functions and Therapeutic Potential
of Extracellular Hsp60, Hsp70, and Hsp90 in Neuroinflammatory Disorders. Applied Sciences.

[148] Fu C., Peng P., Loschko J., Feng L., Pham P., Cui W., Lee K. P., Krug A. B., Jiang A. (2020). Plasmacytoid Dendritic
Cells Cross-Prime Naive Cd8 T Cells by Transferring Antigen to Conventional
Dendritic Cells through Exosomes. Proc. Natl.
Acad. Sci. U. S. A..

[149] Kambayashi T., Laufer T. M. (2014). Atypical Mhc Class Ii-Expressing
Antigen-Presenting Cells: Can Anything Replace a Dendritic Cell?. Nature Reviews Immunology.

[150] Pishesha N., Harmand T. J., Ploegh H. L. (2022). A Guide
to Antigen
Processing and Presentation. Nature Reviews
Immunology.

[151] Altei W. F., Pachane B. C., dos Santos P. K., de Araújo H. S. S. (2021). Extracellular
Vesicles and Integrins:
Partners in Cancer Progression. Role of Exosomes
in Biological Communication Systems.

[152] Smirnova O., Efremov Y., Klyucherev T., Peshkova M., Senkovenko A., Svistunov A., Timashev P. (2024). Direct and Cell-Mediated Ev-Ecm Interplay. Acta Biomaterialia.

[153] Pang X., He X., Qiu Z., Zhang H., Xie R., Liu Z., Gu Y., Zhao N., Xiang Q., Cui Y. (2023). Targeting Integrin Pathways: Mechanisms and Advances in Therapy. Signal Transduction Targeted Therapy.

[154] Wu Y., Wang Q., Jia S., Lu Q., Zhao M. (2024). Gut-Tropic
T Cells and Extra-Intestinal Autoimmune Diseases. Autoimmunity Reviews.

[155] Qiu R., Deng Y., Lu Y., Liu X., Huang Q., Du Y. (2024). Itgb3-Enriched Extracellular Vesicles
Mediate the Formation of Osteoclastic
Pre-Metastatic Niche to Promote Lung Adenocarcinoma Bone Metastasis. Mol. Carcinog.

[156] Yamamoto S., Kuwada T., Shiokawa M., Kitamoto H., Okabe M., Seno H. (2025). Anti-Integrin Αvβ6 Antibody
Titer as a Predictive Biomarker of Future Treatment Escalation in
Patients with Ulcerative Colitis. Gastro Hep
Adv..

[157] Shen Y. Q., Sun L., Wang S. M., Zheng X. Y., Xu R. (2024). Exosomal Integrins in Tumor Progression,
Treatment and Clinical Prediction
(Review). Int. J. Oncol..

[158] Papareddy P., Tapken I., Kroh K., Varma Bhongir R. K., Rahman M., Baumgarten M., Cim E. I., Györffy L., Smeds E., Neumann A. (2024). The Role of Extracellular Vesicle
Fusion with Target Cells in Triggering Systemic Inflammation. Nat. Commun..

[159] Li Y., Wang J., Chen W., Lu H., Zhang Y. (2024). Comprehensive
Review of Ms-Based Studies on N-Glycoproteome and N-Glycome of Extracellular
Vesicles. Proteomics.

[160] Obeagu E., Obeagu G. (2024). P-Selectin and Immune Activation
in Hiv: Clinical Implications. Elite J. Health
Sci..

[161] Giannessi F., Percario Z., Lombardi V., Sabatini A., Sacchi A., Lisi V., Battistini L., Borsellino G., Affabris E., Angelini D. F. (2024). Macrophages Treated
with Interferons Induce Different Responses in Lymphocytes Via Extracellular
Vesicles. Iscience.

[162] Yokoi A., Ochiya T. (2021). Exosomes and Extracellular
Vesicles:
Rethinking the Essential Values in Cancer Biology. Semin. Cancer Biol..

[163] Pasrija R., Naime M. (2021). The Deregulated Immune Reaction and
Cytokines Release Storm (Crs) in Covid-19 Disease. International Immunopharmacology.

[164] Halder L. D., Jo E. A., Hasan M. Z., Ferreira-Gomes M., Krüger T., Westermann M., Palme D. I., Rambach G., Beyersdorf N., Speth C. (2020). Immune Modulation by
Complement Receptor 3-Dependent Human Monocyte Tgf-Β1-Transporting
Vesicles. Nat. Commun..

[165] Mohammadipoor A., Hershfield M. R., Linsenbardt H. R., Smith J., Mack J., Natesan S., Averitt D. L., Stark T. R., Sosanya N. M. (2023). Biological
Function of Extracellular
Vesicles (Evs): A Review of the Field. Molecular
Biology Reports.

[166] Guo T., He C., Venado A., Zhou Y. (2022). Extracellular
Matrix
Stiffness in Lung Health and Disease. Compr.
Physiol..

[167] Fujii F., Kanemasa H., Okuzono S., Setoyama D., Taira R., Yonemoto K., Motomura Y., Kato H., Masuda K., Kato T. A. (2024). Atp1a3 Regulates Protein Synthesis
for Mitochondrial Stability under Heat Stress. Dis. Models Mech..

[168] Pinho S. S., Alves I., Gaifem J., Rabinovich G. A. (2023). Immune
Regulatory Networks Coordinated by Glycans and Glycan-Binding Proteins
in Autoimmunity and Infection. Cellular &
Molecular Immunology.

[169] Macedo-da-Silva J., Santiago V. F., Rosa-Fernandes L., Marinho C. R. F., Palmisano G. (2021). Protein Glycosylation in Extracellular
Vesicles: Structural Characterization and Biological Functions. Mol. Immunol.

[170] Li P., Xu X., Zhang C., Chang Q., Wang J., Wang W., Ren H. (2025). Glycosylation on Extracellular
Vesicles
and Its Detection Strategy: Paving the Way for Clinical Use. Int. J. Biol. Macromol..

[171] Zhang G., Huang X., Gong Y., Ding Y., Wang H., Zhang H., Wu L., Su R., Yang C., Zhu Z. (2024). Fingerprint Profiling of Glycans
on Extracellular Vesicles Via Lectin-Induced Aggregation Strategy
for Precise Cancer Diagnostics. J. Am. Chem.
Soc..

[172] Pace A., Scirocchi F., Napoletano C., Zizzari I. G., D’Angelo L., Santoro A., Nuti M., Rahimi H., Rughetti A. (2022). Glycan-Lectin
Interactions as Novel
Immunosuppression Drivers in Glioblastoma. Int.
J. Mol. Sci..

[173] Li Y., Zhang S., Liu C., Deng J., Tian F., Feng Q., Qin L., Bai L., Fu T., Zhang L. (2024). Thermophoretic Glycan Profiling of Extracellular
Vesicles
for Triple-Negative Breast Cancer Management. Nat. Commun..

[174] Janke C., Magiera M. M. (2020). The Tubulin Code
and Its Role in
Controlling Microtubule Properties and Functions. Nat. Rev. Mol. Cell Biol..

[175] Lappalainen P., Kotila T., Jégou A., Romet-Lemonne G. (2022). Biochemical and Mechanical Regulation of Actin Dynamics. Nat. Rev. Mol. Cell Biol..

[176] Shehjar F., Almarghalani D. A., Mahajan R., Hasan S. A.-M., Shah Z. A. (2024). The Multifaceted
Role of Cofilin in Neurodegeneration
and Stroke: Insights into Pathogenesis and Targeting as a Therapy. Cells.

[177] Hu Q., Su H., Li J., Lyon C., Tang W., Wan M., Hu T. Y. (2020). Clinical
Applications of Exosome Membrane Proteins. Precis
Clin Med..

[178] Alexander M., Ramstead A. G., Bauer K. M., Lee S.-H., Runtsch M. C., Wallace J., Huffaker T. B., Larsen D. K., Tolmachova T., Seabra M. C. (2017). Rab27-Dependent Exosome
Production Inhibits Chronic Inflammation and Enables Acute Responses
to Inflammatory Stimuli. J. Immunol..

[179] de Souza Ferreira L.
P., da Silva R. A., Gil C. D., Geisow M. J. (2023). Annexin
A1, A2, A5, and A6 Involvement in Human Pathologies. Proteins: Struct., Funct., Bioinf..

[180] Cao M., Luo X., Wu K., He X. (2021). Targeting Lysosomes
in Human Disease: From Basic Research to Clinical Applications. Signal Transduction Targeted Ther..

[181] Mehta K., Yentsch H., Lee J., Yook Y., Lee K. Y., Gao T. T., Tsai N. P., Zhang K. (2024). Phosphatidylinositol-3-Phosphate
Mediates Arc Capsid Secretion through the Multivesicular Body Pathway. Proc. Natl. Acad. Sci. U. S. A..

[182] Chin L. K., Son T., Hong J.-S., Liu A.-Q., Skog J., Castro C. M., Weissleder R., Lee H., Im H. (2020). Plasmonic Sensors for Extracellular Vesicle Analysis:
From Scientific Development to Translational Research. ACS Nano.

[183] Zhang Y., Liang F., Zhang D., Qi S., Liu Y. (2023). Metabolites as Extracellular Vesicle Cargo in Health, Cancer, Pleural
Effusion, and Cardiovascular Diseases: An Emerging Field of Study
to Diagnostic and Therapeutic Purposes. Biomedicine
& Pharmacotherapy.

[184] Bhat O. M., Mir R. A., Nehvi I. B., Wani N. A., Dar A. H., Zargar M. A. (2024). Emerging Role of Sphingolipids and
Extracellular Vesicles in Development and Therapeutics of Cardiovascular
Diseases. Int. J. Cardiol Heart Vasc.

[185] Rausch L., Flaskamp L., Ashokkumar A., Trefzer A., Ried C., Buchholz V. R., Obst R., Straub T., Brocker T., Kranich J. (2023). Phosphatidylserine-Positive
Extracellular Vesicles Boost Effector Cd8+ T Cell Responses During
Viral Infection. Proc. Natl. Acad. Sci. U. S.
A..

[186] Skotland T., Llorente A., Sandvig K. (2023). Lipids in Extracellular
Vesicles: What Can Be Learned About Membrane Structure and Function?. Cold Spring Harbor Perspectives in Biology.

[187] Kumar M. A., Baba S. K., Sadida H. Q., Marzooqi S. A., Jerobin J., Altemani F. H., Algehainy N., Alanazi M. A., Abou-Samra A.-B., Kumar R. (2024). Extracellular
Vesicles as Tools and Targets in Therapy for Diseases. Signal Transduction Target Ther..

[188] Li Q., Zhan S., Yang X., Zhang Z., Sun N., Wang X., Kang J., Du R., Hong X., Yue M. (2024). Choline Phosphate-Grafted
Nanozymes as Universal Extracellular
Vesicle Probes for Bladder Cancer Detection. ACS Nano.

[189] Somnay Y. R., Kunnimalaiyaan M. (2012). The Phosphatidylinositol 3-Kinase/Akt
Signaling Pathway in Neuroendocrine Tumors. Glob. J. Biochem..

[190] Iwabuchi K., Nakayama H., Oizumi A., Suga Y., Ogawa H., Takamori K. (2015). Role of Ceramide from
Glycosphingolipids
and Its Metabolites in Immunological and Inflammatory Responses in
Humans. Mediators Inflamm.

[191] Yuyama K., Sun H., Igarashi Y., Monde K., Hirase T., Nakayama M., Makino Y. (2022). Immuno-Digital
Invasive
Cleavage Assay for Analyzing Alzheimer’s Amyloid ß-Bound
Extracellular Vesicles. Alzheimers Res. Ther..

[192] Ghadami S., Dellinger K. (2023). The Lipid Composition of Extracellular
Vesicles: Applications in Diagnostics and Therapeutic Delivery. Front. Mol. Biosci..

[193] Huang Q., Kang T., Shen S., Liu L., Zhang L., Zou X., Wu J. (2025). Extracellular Vesicular
Delivery of Ceramides from Pulmonary Macrophages to Endothelial Cells
Facilitates Chronic Obstructive Pulmonary Disease. Cell Commun. Signal.

[194] Yang M., Zhang Y., Li M., Liu X., Darvishi M. (2023). The Various Role of Micrornas in Breast Cancer Angiogenesis,
with a Special Focus on Novel Mirna-Based Delivery Strategies. Cancer Cell Int..

[195] Wei Y., Wang Z., Qin Z., Wan Q., Li Y., Tay F. R., Wang C., Zhang T., Niu L. (2024). The Contribution
of Extracellular Rna and Its Derived Biomaterials in Disease Management. BMEMat.

[196] Doghish A. S., Elballal M. S., Elazazy O., Elesawy A. E., Elrebehy M. A., Shahin R. K., Midan H. M., Sallam A.-A. M. (2023). The
Role of Mirnas in Liver Diseases: Potential Therapeutic and Clinical
Applications. Pathology-Research and Practice.

[197] Zhang L., Wu T., Shan Y., Li G., Ni X., Chen X., Hu X., Lin L., Li Y., Guan Y. (2021). Therapeutic Reversal of Huntington’s Disease
by in Vivo Self-Assembled
Sirnas. Brain.

[198] Wang M., Yu F., Li P., Wang K. (2020). Emerging Function
and Clinical Significance of Exosomal Circrnas in Cancer. Molecular Therapy-Nucleic Acids.

[199] Pu X., Zhang C., Ding G., Gu H., Lv Y., Shen T., Pang T., Cao L., Jia S. (2024). Diagnostic
Plasma Small Extracellular Vesicles Mirna Signatures for Pancreatic
Cancer Using Machine Learning Methods. Translational
Oncology.

[200] Nguyen L. T., Zhang J., Rima X. Y., Wang X., Kwak K. J., Okimoto T., Amann J., Yoon M. J., Shukuya T., Chiang C. L. (2022). An Immunogold Single Extracellular
Vesicular Rna and Protein (Auserp) Biochip to Predict Responses to
Immunotherapy in Non-Small Cell Lung Cancer Patients. J. Extracell. Vesicles.

[201] Hu M., Brown V., Jackson J. M., Wijerathne H., Pathak H., Koestler D. C., Nissen E., Hupert M. L., Muller R., Godwin A. K. (2023). Assessing
Breast Cancer
Molecular Subtypes Using Extracellular Vesicles’ Mrna. Anal. Chem..

[202] Suresh P. S., Thankachan S., Venkatesh T. (2023). Landscape
of Clinically Relevant Exosomal Trna-Derived Non-Coding Rnas. Molecular Biotechnology.

[203] Lu G., Jiang X., Wu A., Zhou J., Liu H., He F., Zhang Q., Zen K., Gu S., Wang J. (2021). Two Small
Extracellular Vesicle Srnas Derived from Mycobacterium Tuberculosis
Serve as Diagnostic Biomarkers for Active Pulmonary Tuberculosis. Frontiers in Microbiology.

[204] Campos C. D. M., Childers K., Gamage S. S. T., Wijerathne H., Zhao Z., Soper S. A. (2021). Analytical Technologies
for Liquid
Biopsy of Subcellular Materials. Annu. Rev.
Anal Chem. (Palo Alto Calif).

[205] Miceli R. T., Chen T. Y., Nose Y., Tichkule S., Brown B., Fullard J. F., Saulsbury M. D., Heyliger S. O., Gnjatic S., Kyprianou N. (2024). Extracellular
Vesicles, Rna Sequencing, and Bioinformatic Analyses: Challenges,
Solutions, and Recommendations. J. Extracell.
Vesicles.

[206] Wijerathne H., Witek M. A., Jackson J. M., Brown V., Hupert M. L., Herrera K., Kramer C., Davidow A. E., Li Y., Baird A. E. (2020). Affinity Enrichment
of Extracellular Vesicles from
Plasma Reveals Mrna Changes Associated with Acute Ischemic Stroke. Commun. Biol..

[207] Zhao Z., Wijerathne H., Godwin A. K., Soper S. A. (2021). Isolation
and Analysis Methods of Extracellular Vesicles (Evs). Extracell. Vesicles Circ. Nucl. Acids.

[208] Elzanowska J., Semira C., Costa-Silva B. (2021). DNA in Extracellular
Vesicles: Biological and Clinical Aspects. Molecular
Oncology.

[209] Ghanam J., Chetty V. K., Barthel L., Reinhardt D., Hoyer P.-F., Thakur B. K. (2022). DNA in Extracellular Vesicles: From
Evolution to Its Current Application in Health and Disease. Cell Biosci..

[210] Zhu D., Ma X., Wang J., Chen T., Yang J., Liu Y., Lin Z., Wu M., Hu T. Y., Zhang Y. (2025). A Sequential
Release Micro-Nano System for Colitis Therapy Via Gut Microbiota and
Immune Regulation. Angew. Chem., Int. Ed. Engl..

[211] Wang Y., Lou P., Xie Y., Liu S., Li L., Wang C., Du D., Chen Y., Lu Y., Cheng J., Liu J. (2024). Nutrient Availability Regulates the
Secretion and Function of Immune Cell-Derived Extracellular Vesicles
through Metabolic Rewiring. Science. Sci. Adv..

[212] Del Prete A., Salvi V., Soriani A., Laffranchi M., Sozio F., Bosisio D., Sozzani S. (2023). Dendritic Cell Subsets
in Cancer Immunity and Tumor Antigen Sensing. Cellular & Molecular Immunology.

[213] Wang Y., Zhao M., Liu S., Guo J., Lu Y., Cheng J., Liu J. (2020). Macrophage-Derived
Extracellular
Vesicles: Diverse Mediators of Pathology and Therapeutics in Multiple
Diseases. Cell Death Dis..

[214] Izquierdo-Altarejos P., Moreno-Manzano V., Felipo V. (2024). Pathological and Therapeutic
Effects of Extracellular Vesicles in Neurological and Neurodegenerative
Diseases. Neural Regeneration Research.

[215] Nampoothiri S., Nogueiras R., Schwaninger M., Prevot V. (2022). Glial Cells as Integrators of Peripheral
and Central
Signals in the Regulation of Energy Homeostasis. Nature metabolism.

[216] Gao X., Gao H., Yue K., Cao X., Yang E., Zhang Z., Huang Y., Li X., Ding D., Luo P. (2023). Observing Extracellular
Vesicles Originating from Endothelial
Cells in Vivo Demonstrates Improved Astrocyte Function Following Ischemic
Stroke Via Aggregation-Induced Emission Luminogens. ACS Nano.

[217] Lee E. J., Choi Y., Lee H. J., Hwang D. W., Lee D. S. (2022). Human Neural Stem Cell-Derived Extracellular Vesicles
Protect against Parkinson’s Disease Pathologies. J. Nanobiotechnol..

[218] Gao C., Jiang J., Tan Y., Chen S. (2023). Microglia in Neurodegenerative
Diseases: Mechanism and Potential Therapeutic Targets. Signal Transduction Targeted Ther..

[219] Murphy D. E., de Jong O. G., Brouwer M., Wood M. J., Lavieu G., Schiffelers R. M., Vader P. (2019). Extracellular Vesicle-Based
Therapeutics: Natural Versus Engineered Targeting and Trafficking. Exp. Mol. Med..

[220] Pistono C., Bister N., Stanová I., Malm T. (2021). Glia-Derived Extracellular Vesicles: Role in Central Nervous System
Communication in Health and Disease. Frontiers
in Cell and Developmental Biology.

[221] Norman M., Ter-Ovanesyan D., Trieu W., Lazarovits R., Kowal E. J., Lee J. H., Chen-Plotkin A. S., Regev A., Church G. M., Walt D. R. (2021). L1cam Is Not Associated
with Extracellular Vesicles in Human Cerebrospinal Fluid or Plasma. Nat. Methods.

[222] Wang X., Yang H., Liu C., Liu K. (2023). A New Diagnostic
Tool for Brain Disorders: Extracellular Vesicles Derived from Neuron,
Astrocyte, and Oligodendrocyte. Front. Mol.
Neurosci..

[223] Li T., Zhang L., Wang P., Yu J., Zhong J., Tang Q., Zhu T., Chen K., Li F., Hong P. (2024). Extracellular Vesicles from Neural Stem Cells
Safeguard
Neurons in Intracerebral Hemorrhage by Suppressing Reactive Astrocyte
Neurotoxicity. Cell Rep.

[224] Hermann D. M., Peruzzotti-Jametti L., Giebel B., Pluchino S. (2024). Extracellular
Vesicles Set the Stage for Brain Plasticity and Recovery by Multimodal
Signalling. Brain.

[225] Beatriz M., Rodrigues R. J., Vilaça R., Egas C., Pinheiro P. S., Daley G. Q., Schlaeger T. M., Raimundo N., Rego A. C., Lopes C. (2023). Extracellular
Vesicles
Improve Gabaergic Transmission in Huntington’s Disease Ipsc-Derived
Neurons. Theranostics.

[226] Saeedi S., Israel S., Nagy C., Turecki G. (2019). The Emerging
Role of Exosomes in Mental Disorders. Transl.
Psychiatry.

[227] Chen Y., Zhu Q., Cheng L., Wang Y., Li M., Yang Q., Hu L., Lou D., Li J., Dong X. (2021). Exosome Detection Via
the Ultrafast-Isolation System:
Exodus. Nat. Methods.

[228] Palanisamy C. P., Pei J., Alugoju P., Anthikapalli N. V. A., Jayaraman S., Veeraraghavan V. P., Gopathy S., Roy J. R., Janaki C. S., Thalamati D. (2023). New Strategies
of Neurodegenerative
Disease Treatment with Extracellular Vesicles (Evs) Derived from Mesenchymal
Stem Cells (Mscs). Theranostics.

[229] Upadhya R., Madhu L. N., Attaluri S., Gitaí D. L. G., Pinson M. R., Kodali M., Shetty G., Zanirati G., Kumar S., Shuai B. (2020). Extracellular Vesicles from Human
Ipsc-Derived Neural Stem Cells: Mirna and Protein Signatures, and
Anti-Inflammatory and Neurogenic Properties. J. Extracell. Vesicles.

[230] Yudintceva N., Bobkov D., Sulatsky M., Mikhailova N., Oganesyan E., Vinogradova T., Muraviov A., Remezova A., Bogdanova E., Garapach I. (2024). Mesenchymal Stem Cells-Derived Extracellular
Vesicles for Therapeutics of Renal Tuberculosis. Sci. Rep..

[231] Xia Y., Hu G., Chen Y., Yuan J., Zhang J., Wang S., Li Q., Wang Y., Deng Z. (2021). Embryonic
Stem Cell Derived Small Extracellular Vesicles Modulate Regulatory
T Cells to Protect against Ischemic Stroke. ACS Nano.

[232] Al Naem M., Bourebaba L., Kucharczyk K., Röcken M., Marycz K. (2020). Therapeutic Mesenchymal Stromal Stem
Cells: Isolation, Characterization and Role in Equine Regenerative
Medicine and Metabolic Disorders. Stem cell
reviews and reports.

[233] Varderidou-Minasian S., Lorenowicz M. J. (2020). Mesenchymal
Stromal/Stem Cell-Derived
Extracellular Vesicles in Tissue Repair: Challenges and Opportunities. Theranostics.

[234] Samuels M., Jones W., Towler B., Turner C., Robinson S., Giamas G. (2023). The Role of Non-Coding
Rnas in Extracellular
Vesicles in Breast Cancer and Their Diagnostic Implications. Oncogene.

[235] Williams T., Salmanian G., Burns M., Maldonado V., Smith E., Porter R. M., Song Y. H., Samsonraj R. M. (2023). Versatility
of Mesenchymal Stem Cell-Derived Extracellular Vesicles in Tissue
Repair and Regenerative Applications. Biochimie.

[236] Peng X., Wang Q., Li W., Ge G., Peng J., Xu Y., Yang H., Bai J., Geng D. (2023). Comprehensive Overview of Microrna Function in Rheumatoid Arthritis. Bone Res..

[237] Bandu R., Oh J. W., Kim K. P. (2019). Mass Spectrometry-Based
Proteome Profiling of Extracellular Vesicles and Their Roles in Cancer
Biology. Experimental & molecular medicine.

[238] Min X.-l., Liu H.-j., Dou X.-k., Chen F.-x., Zhao Q., Zhao X.-h., Shi Y., Zhao Q.-y., Sun S.-j., Wang Z. (2024). Extracellular Vesicles
from Neural
Stem Cells Carry Microrna-16–5p to Reduce Corticosterone-Induced
Neuronal Injury in Depression Rats. Neuroscience.

[239] Ma K., Xu H., Zhang J., Zhao F., Liang H., Sun H., Li P., Zhang S., Wang R., Chen X. (2019). Insulin-Like
Growth Factor-1 Enhances Neuroprotective Effects of Neural Stem Cell
Exosomes after Spinal Cord Injury Via an Mir-219a-2–3p/Yy1Mechanism. Aging (Albany NY).

[240] Kou M., Huang L., Yang J., Chiang Z., Chen S., Liu J., Guo L., Zhang X., Zhou X., Xu X., Yan X., Wang Y., Zhang J., Xu A., Tse H. f., Lian Q. (2022). Mesenchymal Stem Cell-Derived Extracellular Vesicles for Immunomodulation
and Regeneration: A Next Generation Therapeutic Tool?. Cell Death Dis..

[241] Morales R.-T. T., Ko J. (2022). Future of Digital Assays
to Resolve
Clinical Heterogeneity of Single Extracellular Vesicles. ACS Nano.

[242] Rangel-Ramírez V. V., González-Sánchez H. M., Lucio-García C. (2023). Exosomes: From Biology to Immunotherapy in Infectious
Diseases. Infectious Diseases.

[243] Rodrigues M., Fan J., Lyon C., Wan M., Hu Y. (2018). Role of Extracellular Vesicles in Viral and Bacterial
Infections:
Pathogenesis, Diagnostics, and Therapeutics. Theranostics.

[244] Kuang H., Dou G., Cheng L., Wang X., Xu H., Liu X., Ding F., Yang X., Liu S., Bao L. (2023). Humoral
Regulation of Iron Metabolism by Extracellular
Vesicles Drives Antibacterial Response. Nat.
Metab.

[245] Chronopoulos A., Kalluri R. (2020). Emerging Role of Bacterial Extracellular
Vesicles in Cancer. Oncogene.

[246] Tulkens J., De Wever O., Hendrix A. (2020). Analyzing
Bacterial
Extracellular Vesicles in Human Body Fluids by Orthogonal Biophysical
Separation and Biochemical Characterization. Nat. Protoc..

[247] Doré, E. ; Boilard, E. Bacterial Extracellular Vesicles and Their Interplay with the Immune System. Pharmacology & Therapeutics 2023, 247 108443. 10.1016/j.pharmthera.2023.108443 37210006

[248] Lai L., Su Y., Hu C., Peng Z., Xue W., Dong L., Hu T. Y. (2025). Integrating
Aggregate Materials and
Machine Learning Algorithms: Advancing Detection of Pathogen-Derived
Extracellular Vesicles. Aggregate.

[249] Gan Y., Zhao G., Wang Z., Zhang X., Wu M. X., Lu M. (2023). Bacterial Membrane Vesicles: Physiological Roles, Infection Immunology,
and Applications. Adv. Sci..

[250] Zheng W., LaCourse S. M., Song B., Singh D. K., Khanna M., Olivo J., Stern J., Escudero J. N., Vergara C., Zhang F. (2022). Diagnosis of Paediatric
Tuberculosis by Optically Detecting Two Virulence Factors on Extracellular
Vesicles in Blood Samples. Nature biomedical
engineering.

[251] Tan Y.-j., Jin Y., Zhou J., Yang Y.-f. (2024). Lipid Droplets
in Pathogen Infection and Host Immunity. Acta
Pharmacologica Sinica.

[252] Suri K., D’Souza A., Huang D., Bhavsar A., Amiji M. (2023). Bacterial Extracellular Vesicle Applications in Cancer Immunotherapy. Bioactive Materials.

[253] Ning B., Huang Z., Youngquist B. M., Scott J. W., Niu A., Bojanowski C. M., Zwezdaryk K. J., Saba N. S., Fan J., Yin X.-M. (2021). Liposome-Mediated
Detection of Sars-Cov-2 Rna-Positive Extracellular Vesicles in Plasma. Nat. Nanotechnol..

[254] Pordanjani P. M., Bolhassani A., Milani A., Pouriayevali M. H. (2023). Extracellular
Vesicles in Vaccine Development and Therapeutic Approaches for Viral
Diseases. Process Biochemistry.

[255] He B., Wang H., Liu G., Chen A., Calvo A., Cai Q., Jin H. (2023). Fungal Small
Rnas Ride in Extracellular Vesicles to
Enter Plant Cells through Clathrin-Mediated Endocytosis. Nat. Commun..

[256] Salas N., Coceres V. M., Melo T. d. S., Pereira-Neves A., Maguire V. G., Rodriguez T. M., Sabatke B., Ramirez M. I., Sha J., Wohlschlegel J. A. (2022). Vps32,
a Member of the Escrt Complex,
Modulates Adherence to Host Cells in the Parasite Trichomonas Vaginalis
by Affecting Biogenesis and Cargo Sorting of Released Extracellular
Vesicles. Cell. Mol. Life Sci..

[257] Rooney J., Northcote H. M., Williams T. L., Cortés A., Cantacessi C., Morphew R. M. (2022). Parasitic Helminths and the Host
Microbiome–a Missing ‘Extracellular Vesicle-Sized’link?. Trends in Parasitology.

[258] Almeria C., Kreß S., Weber V., Egger D., Kasper C. (2022). Heterogeneity of Mesenchymal Stem Cell-Derived Extracellular
Vesicles Is Highly Impacted by the Tissue/Cell Source and Culture
Conditions. Cell Biosci..

[259] Wan M., Ning B., Spiegel S., Lyon C. J., Hu T. Y. (2020). Tumor-Derived
Exosomes (Tdes): How to Avoid the Sting in the Tail. Med. Res. Rev..

[260] Yao C., Wang C. (2023). Platelet-Derived Extracellular Vesicles for Drug Delivery. Biomaterials Science.

[261] Hallal S., Tűzesi Á., Grau G. E., Buckland M. E., Alexander K. L. (2022). Understanding
the Extracellular Vesicle Surface for
Clinical Molecular Biology. J. Extracell. Vesicles.

[262] Burnouf T., Chou M.-L., Lundy D. J., Chuang E.-Y., Tseng C.-L., Goubran H. (2023). Expanding Applications of Allogeneic
Platelets, Platelet Lysates, and Platelet Extracellular Vesicles in
Cell Therapy, Regenerative Medicine, and Targeted Drug Delivery. J. Biomed. Sci..

[263] Hallal S. M., Sida L. A., Tűzesi C. Á., Shivalingam B., Sim H. W., Buckland M. E., Satgunaseelan L., Alexander K. L. (2024). Size Matters: Biomolecular Compositions of Small and
Large Extracellular Vesicles in the Urine of Glioblastoma Patients. J. Extracell. Biol..

[264] Longobardi A., Nicsanu R., Bellini S., Squitti R., Catania M., Tiraboschi P., Saraceno C., Ferrari C., Zanardini R., Binetti G. (2022). Cerebrospinal Fluid Ev Concentration
and Size are Altered in Alzheimer’s Disease and Dementia with
Lewy Bodies. Cells.

[265] Vats R., Yadav P., Bano A., Wadhwa S., Bhardwaj R. (2024). Salivary Biomarkers
in Non-Invasive Oral Cancer Diagnostics:
A Comprehensive Review. J. Appl. Oral Sci..

[266] Holzhausen E. A., Kupsco A., Chalifour B. N., Patterson W. B., Schmidt K. A., Mokhtari P., Baccarelli A. A., Goran M. I., Alderete T. L. (2023). Influence of Technical and Maternal-Infant
Factors on the Measurement and Expression of Extracellular Mirna in
Human Milk. Front Immunol..

[267] Chen T. Y., Mihalopoulos M., Zuluaga L., Rich J., Ganta T., Mehrazin R., Tsao C. K., Tewari A., Gonzalez-Kozlova E., Badani K. (2023). Clinical Significance of Extracellular
Vesicles in Prostate and Renal Cancer. Int.
J. Mol. Sci..

[268] Llorente A., Broka̅ne A., Mlynska A., Puurand M., Sagini K., Folkmane S., Hjorth M., Martin-Gracia B., Romero S., Skorinkina D. (2024). From Sweat
to Hope: The Role of Exercise-Induced
Extracellular Vesicles in Cancer Prevention and Treatment. J. Extracell. Vesicles.

[269] De Sousa K. P., Rossi I., Abdullahi M., Ramirez M. I., Stratton D., Inal J. M. (2023). Isolation and Characterization
of Extracellular Vesicles and Future Directions in Diagnosis and Therapy. Wiley Interdiscip. Rev.: Nanomed. Nanobiotechnol..

[270] Shekari F., Alibhai F. J., Baharvand H., Börger V., Bruno S., Davies O., Giebel B., Gimona M., Salekdeh G. H., Martin-Jaular L. (2023). Cell Culture-Derived
Extracellular Vesicles: Considerations for Reporting Cell Culturing
Parameters. J. Extracell. Biol..

[271] Millan C., Prause L., Vallmajo-Martin Q., Hensky N., Eberli D. (2022). Extracellular
Vesicles from 3d Engineered
Microtissues Harbor Disease-Related Cargo Absent in Evs from 2d Cultures. Adv. Healthcare Mater..

[272] Welsh J. A., van der Pol E., Bettin B. A., Carter D. R. F., Hendrix A., Lenassi M., Langlois M., Llorente A., van de Nes A. S., Nieuwland R., Tang V., Wang L., Witwer K. W., Jones J. C. (2020). Towards Defining Reference Materials
for Measuring Extracellular Vesicle Refractive Index, Epitope Abundance,
Size and Concentration. J. Extracell. Vesicles.

[273] Lee Y., Kim J.-H. (2022). The Emerging Roles
of Extracellular Vesicles as Intercellular
Messengers in Liver Physiology and Pathology. Clinical and Molecular Hepatology.

[274] Kalluri R. (2024). The Biology and Function of Extracellular Vesicles
in Immune Response and Immunity. Immunity.

[275] Ruf B., Greten T. F., Korangy F. (2023). Innate Lymphoid
Cells and Innate-Like
T Cells in Cancer—at the Crossroads of Innate and Adaptive
Immunity. Nature Reviews Cancer.

[276] Alberts, B. ; Johnson, A. ; Lewis, J. ; Raff, M. ; Roberts, K. ; Walter, P. Helper T Cells and Lymphocyte Activation. In Mol. Biol. Cell. 4th ed., Garland Science, 2002.

[277] Luo X. H., Zhu Y., Mao J., Du R. C. (2021). T Cell
Immunobiology and Cytokine Storm of Covid-19. Scand. J. Immunol..

[278] Soder R. P., Dawn B., Weiss M. L., Dunavin N., Weir S., Mitchell J., Li M., Shune L., Singh A. K., Ganguly S. (2020). A Phase
I Study to Evaluate
Two Doses of Wharton’s Jelly-Derived Mesenchymal Stromal Cells
for the Treatment of De Novo High-Risk or Steroid-Refractory Acute
Graft Versus Host Disease. Stem Cell Rev. Rep.

[279] Zhang J., Gu J., Wang X., Ji C., Yu D., Wang M., Pan J., Santos H. A., Zhang H., Zhang X. (2024). Engineering and Targeting Neutrophils
for Cancer Therapy. Adv. Mater..

[280] Marar C., Starich B., Wirtz D. (2021). Extracellular Vesicles
in Immunomodulation and Tumor Progression. Nature
immunology.

[281] Lee K. S., Yeom S. H., Kim M. K., Woo C. H., Choi Y. C., Choi J. S., Cho Y. W. (2024). Therapeutic Potential
of Human Stem Cell-Derived Extracellular Vesicles in Idiopathic Pulmonary
Fibrosis. Extracellular Vesicle.

[282] El-Dessouki A. M., Alzokaky A. A., Raslan N. A., Ibrahim S., Salama L. A., Yousef E. H. (2024). Piracetam Mitigates
Nephrotoxicity
Induced by Cisplatin Via the Ampk-Mediated Pi3k/Akt and Mapk/Jnk/Erk
Signaling Pathways. International Immunopharmacology.

[283] da Costa Fernandes C.
J. (2024). Unveiling the Intricacies
of Bone
Homeostasis: Epigenetic Regulation, Extracellular Vesicles, and Angiogenesis
Integration. Extracell. Vesicle.

[284] Nguyen P. H. D., Jayasinghe M. K., Le A. H., Peng B., Le M. T. (2023). Advances in Drug Delivery Systems Based on Red Blood Cells and Their
Membrane-Derived Nanoparticles. ACS Nano.

[285] Zhang Z., Wu W., Li M., Du L., Li J., Yin X., Zhang W. (2024). Mesenchymal Stem Cell-Derived
Extracellular
Vesicles: A Novel Nanoimmunoregulatory Tool in Musculoskeletal Diseases. Nano Today.

[286] Liu S., Wu X., Chandra S., Lyon C., Ning B., Jiang L., Fan J., Hu T. Y. (2022). Extracellular
Vesicles:
Emerging Tools as Therapeutic Agent Carriers. Acta Pharm. Sin B.

[287] Bhattacharya B., Nag S., Mukherjee S., Kulkarni M., Chandane P., Mandal D., Mukerjee N., Mirgh D., Anand K., Adhikari M. D. (2024). Role
of Exosomes in Epithelial-Mesenchymal Transition. ACS Applied Bio Materials.

[288] Saliba J. G., Zheng W., Shu Q., Li L., Wu C., Xie Y., Lyon C. J., Qu J., Huang H., Ying B., Hu T. Y. (2025). Enhanced Diagnosis of Multi-Drug-Resistant
Microbes Using Group Association Modeling and Machine Learning. Nat. Commun..

[289] Panda S. S., Sahoo R. K., Patra S. K., Biswal S., Biswal B. K. (2024). Molecular Insights to Therapeutic
in Cancer: Role of
Exosomes in Tumor Microenvironment, Metastatic Progression and Drug
Resistance. Drug Discovery Today.

[290] Crow J., Atay S., Banskota S., Artale B., Schmitt S., Godwin A. K. (2017). Exosomes as Mediators
of Platinum
Resistance in Ovarian Cancer. Oncotarget.

[291] Hu T., Wolfram J., Srivastava S. (2021). Extracellular
Vesicles in Cancer
Detection: Hopes and Hypes. Trends Cancer.

[292] Liu Q., Li D., Pan X., Liang Y. (2023). Targeted Therapy Using
Engineered Extracellular Vesicles: Principles and Strategies for Membrane
Modification. J. Nanobiotechnol..

[293] Zhang C., Qin C., Dewanjee S., Bhattacharya H., Chakraborty P., Jha N. K., Gangopadhyay M., Jha S. K., Liu Q. (2024). Tumor-Derived Small Extracellular
Vesicles in Cancer Invasion and Metastasis: Molecular Mechanisms,
and Clinical Significance. Mol. Cancer.

[294] Atay S., Godwin A. K. (2014). Tumor-Derived Exosomes: A Message
Delivery System for Tumor Progression. Commun.
Integr Biol..

[295] Atay S., Wilkey D. W., Milhem M., Merchant M., Godwin A. K. (2018). Insights into the Proteome of Gastrointestinal
Stromal
Tumors-Derived Exosomes Reveals New Potential Diagnostic Biomarkers. Mol. Cell Proteomics.

[296] Ramirez-Garrastacho M., Bajo-Santos C., Line A., Martens-Uzunova E. S., de la Fuente J. M., Moros M., Soekmadji C., Tasken K. A., Llorente A. (2022). Extracellular
Vesicles as a Source
of Prostate Cancer Biomarkers in Liquid Biopsies: A Decade of Research. British journal of cancer.

[297] O’Grady T., Njock M.-S., Lion M., Bruyr J., Mariavelle E., Galvan B., Boeckx A., Struman I., Dequiedt F. (2022). Sorting and Packaging of Rna into
Extracellular Vesicles
Shape Intracellular Transcript Levels. BMC Biol..

[298] Bukong T. N., Momen-Heravi F., Kodys K., Bala S., Szabo G. (2014). Exosomes from Hepatitis C Infected Patients Transmit Hcv Infection
and Contain Replication Competent Viral Rna in Complex with Ago2-Mir122-Hsp90. PLoS pathogens.

[299] Mukerjee N., Maitra S., Ghosh A., Sengupta T., Alexiou A., Subramaniyan V., Anand K. (2024). Synergizing Proteolysis-Targeting
Chimeras and Nanoscale Exosome-Based Delivery Mechanisms for Hiv and
Antiviral Therapeutics. ACS Applied Nano Materials.

[300] Zhang, R. ; Yuan, M. ; Giri, B. R. ; Li, S. ; Cheng, G. ; Wu, Z. Extracellular Vesicle Biomarkers for Infectious Diseases. In Extracellular Vesicles: From Bench to Bedside; Springer, 2024; pp 385–407.

[301] DeMarino C., Cowen M., Williams A., Khatkar P., Abulwerdi F. A., Henderson L., Denniss J., Pleet M. L., Luttrell D. R., Vaisman I. (2024). Autophagy Deregulation
in Hiv-1-Infected Cells Increases Extracellular Vesicle Release and
Contributes to Tlr3 Activation. Viruses.

[302] Zhang H., Tang M., Li D., Xu M., Ao Y., Lin L. (2024). Applications and Advances in Molecular
Diagnostics:
Revolutionizing Non-Tuberculous Mycobacteria Species and Subspecies
Identification. Front. Public Health.

[303] Wu W.-C., Song S.-J., Zhang Y., Li X. (2020). Role of Extracellular
Vesicles in Autoimmune Pathogenesis. Frontiers
in immunology.

[304] Tian J., Casella G., Zhang Y., Rostami A., Li X. (2020). Potential
Roles of Extracellular Vesicles in the Pathophysiology,
Diagnosis, and Treatment of Autoimmune Diseases. International journal of biological sciences.

[305] González-Blanco C., Iglesias-Fortes S., Lockwood Á. C., Figaredo C., Vitulli D., Guillén C. (2024). The Role of
Extracellular Vesicles in Metabolic Diseases. Biomedicines.

[306] Li W., Yu L. (2024). Role and Therapeutic
Perspectives of Extracellular
Vesicles Derived from Liver and Adipose Tissue in Metabolic Dysfunction-Associated
Steatotic Liver Disease. Artificial Cells, Nanomedicine,
and Biotechnology.

[307] Jiang H., Qian Y., Shen Z., Liu Y., He Y., Gao R., Shen M., Chen S., Fu Q., Yang T. (2021). Circulating Microrna-135a-3p in Serum Extracellular Vesicles as a
Potential Biological Marker of Non-Alcoholic Fatty Liver Disease. Mol. Med. Rep..

[308] Wang C., Wang S., Kang D. D., Dong Y. (2023). Biomaterials
for in Situ Cell Therapy. BMEMat.

[309] Ruan S., Greenberg Z., Pan X., Zhuang P., Erwin N., He M. (2022). Extracellular Vesicles as an Advanced
Delivery Biomaterial for Precision Cancer Immunotherapy. Adv. Healthc Mater..

[310] Ding Z., Greenberg Z. F., Serafim M. F., Ali S., Jamieson J. C., Traktuev D. O., March K., He M. (2024). Understanding
Molecular Characteristics of Extracellular Vesicles Derived from Different
Types of Mesenchymal Stem Cells for Therapeutic Translation. Extracell Vesicle.

[311] Belhadj Z., Qie Y., Carney R. P., Li Y., Nie G. (2023). Current Advances in Non-Viral Gene Delivery Systems: Liposomes Versus
Extracellular Vesicles. BMEMat.

[312] Elsharkasy O. M., Nordin J. Z., Hagey D. W., de Jong O. G., Schiffelers R. M., Andaloussi S. E., Vader P. (2020). Extracellular Vesicles
as Drug Delivery Systems: Why and How?. Adv.
Drug Deliv Rev..

[313] Xing S., Lu Z., Huang Q., Li H., Wang Y., Lai Y., He Y., Deng M., Liu W. (2020). An Ultrasensitive Hybridization Chain Reaction-Amplified Crispr-Cas12a
Aptasensor for Extracellular Vesicle Surface Protein Quantification. Theranostics.

[314] Das S., Lyon C. J., Hu T. (2024). A Panorama of Extracellular Vesicle
Applications: From Biomarker Detection to Therapeutics. ACS Nano.

[315] Kim H. I., Park J., Zhu Y., Wang X., Han Y., Zhang D. (2024). Recent Advances in Extracellular Vesicles for Therapeutic
Cargo Delivery. Experimental & Molecular
Medicine.

[316] Muskan M., Abeysinghe P., Cecchin R., Branscome H., Morris K. V., Kashanchi F. (2024). Therapeutic Potential of Rna-Enriched
Extracellular Vesicles: The Next Generation in Rna Delivery Via Biogenic
Nanoparticles. Molecular Therapy.

[317] Sabino-Santos G., Leggio C. E., Litwin S. M., Waheed N., Bai S., Ulusan S., Karunathilake A., Elliott D. H., Smira A. R., Chandra S. (2024). Post-Covid Immunity
in Patients with Solid Tumor or
Hematological Malignancies Treated with Sars-Cov-2 Monoclonal Antibodies. Immun. Inflamm. Dis..

[318] Mehta A., Michler T., Merkel O. M. (2021). Sirna Therapeutics
against Respiratory Viral Infections—What Have We Learned for
Potential Covid-19 Therapies?. Adv. Healthcare
Mater..

[319] Yu Y., Li M., Zhao Y., Fan F., Wu W., Gao Y., Bai C. (2022). Immune Cell-Derived Extracellular
Vesicular Micrornas
Induce Pancreatic Beta Cell Apoptosis. Heliyon.

[320] Cheng X., Henick B. S., Cheng K. (2024). Anticancer
Therapy
Targeting Cancer-Derived Extracellular Vesicles. ACS Nano.

[321] Anderson N. L., Anderson N. G. (2002). The Human Plasma Proteome: History,
Character, and Diagnostic Prospects. Mol. Cell
Proteomics.

[322] Théry C., Witwer K. W., Aikawa E., Alcaraz M. J., Anderson J. D., Andriantsitohaina R., Antoniou A., Arab T., Archer F., Atkin-Smith G. K. (2018). Minimal Information for Studies of
Extracellular Vesicles 2018 (Misev2018): A Position Statement of the
International Society for Extracellular Vesicles and Update of the
Misev 2014 Guidelines. J. Extracell. Vesicles.

[323] Zhou X., Jia Y., Mao C., Liu S. (2024). Small Extracellular
Vesicles: Non-Negligible Vesicles in Tumor Progression, Diagnosis,
and Therapy. Cancer Letters.

[324] Saeedi S., Rezayi S., Keshavarz H., Niakan Kalhori S. R. (2023). MRI-Based Brain Tumor Detection Using Convolutional
Deep Learning Methods and Chosen Machine Learning Techniques. BMC Med. Inf. Decis. Making.

[325] Pink R. C., Beaman E.-M., Samuel P., Brooks S. A., Carter D. R. F. (2022). Utilising Extracellular Vesicles
for Early Cancer Diagnostics:
Benefits, Challenges and Recommendations for the Future. Br. J. Cancer.

[326] In’t Veld S. G. J. G., Arkani M., Post E., Antunes-Ferreira M., D’Ambrosi S., Vessies D. C. L., Vermunt L., Vancura A., Muller M., Niemeijer A. L. N., Tannous J., Meijer L. L., Le Large T. Y. S., Mantini G., Wondergem N. E., Heinhuis K. M., van Wilpe S., Smits A. J., Drees E. E. E., Roos E., Leurs C. E., Tjon Kon Fat L. A., van der Lelij E. J., Dwarshuis G., Kamphuis M. J., Visser L. E., Harting R., Gregory A., Schweiger M. W., Wedekind L. E., Ramaker J., Zwaan K., Verschueren H., Bahce I., de Langen A. J., Smit E. F., van den Heuvel M. M., Hartemink K. J., Kuijpers M. J. E., oude Egbrink M. G. A., Griffioen A. W., Rossel R., Hiltermann T. J. N., Lee-Lewandrowski E., Lewandrowski K. B., De Witt Hamer P. C., Kouwenhoven M., Reijneveld J. C., Leenders W. P. J., Hoeben A., Verdonck-de
Leeuw I. M., Leemans C. R., Baatenburg de Jong R. J., Terhaard C. H. J., Takes R. P., Langendijk J. A., de Jager S. C., Kraaijeveld A. O., Pasterkamp G., Smits M., Schalken J. A., Łapińska-Szumczyk S., Łojkowska A., Żaczek A. J., Lokhorst H., van de Donk N. W. C. J., Nijhof I., Prins H. J., Zijlstra J. M., Idema S., Baayen J. C., Teunissen C. E., Killestein J., Besselink M. G., Brammen L., Bachleitner-Hofmann T., Mateen F., Plukker J. T. M., Heger M., de Mast Q., Lisman T., Pegtel D. M., Bogaard H. J., Jassem J., Supernat A., Mehra N., Gerritsen W., de Kroon C. D., Lok C. A. R., Piek J. M. J., Steeghs N., van Houdt W. J., Brakenhoff R. H., Sonke G. S., Verheul H. M., Giovannetti E., Kazemier G., Sabrkhany S., Schuuring E., Sistermans E. A., Wolthuis R., Meijers-Heijboer H., Dorsman J., Oudejans C., Ylstra B., Westerman B. A., van den Broek D., Koppers-Lalic D., Wesseling P., Nilsson R. J. A., Vandertop W. P., Noske D. P., Tannous B. A., Sol N., Best M. G., Wurdinger T. (2022). Detection and Localization of Early-and
Late-Stage Cancers Using Platelet Rna. Cancer
Cell.

[327] Liu Y., Fan J., Xu T., Ahmadinejad N., Hess K., Lin S. H., Zhang J., Liu X., Liu L., Ning B. (2020). Extracellular Vesicle Tetraspanin-8
Level Predicts
Distant Metastasis in Non-Small Cell Lung Cancer after Concurrent
Chemoradiation. Sci. Adv..

[328] Hu Q., Zhang S., Yang Y., Yao J. Q., Tang W. F., Lyon C. J., Hu T. Y., Wan M. H. (2022). Extracellular Vesicles
in the Pathogenesis and Treatment of Acute Lung Injury. Mil. Med. Res..

[329] Qi M., Xia Y., Wu Y., Zhang Z., Wang X., Lu L., Dai C., Song Y., Xu K., Ji W., Zhan L. (2022). Lin28b-High
Breast Cancer Cells Promote Immune Suppression in the
Lung Pre-Metastatic Niche Via Exosomes and Support Cancer Progression. Nat. Commun..

[330] Lu C., Xiao W., Su Y., Zhang X., Chen Y., Lu K., Teng P., Liang J., Yang H., Song Q., Tang Y., Cao D. (2023). Rapid Evaluation of Lung Adenocarcinoma
Progression by Detecting Plasma Extracellular Vesicles with Lateral
Flow Immunoassays. ACS Sensors.

[331] Stridfeldt F., Cavallaro S., Hååg P., Lewensohn R., Linnros J., Viktorsson K., Dev A. (2023). Analyses of Single Extracellular Vesicles from Non-Small Lung Cancer
Cells to Reveal Effects of Epidermal Growth Factor Receptor Inhibitor
Treatments. Talanta.

[332] Bhatta R., Han J., Liu Y., Bo Y., Lee D., Zhou J., Wang Y., Nelson E. R., Chen Q., Zhang X. S. (2023). Metabolic Tagging of Extracellular
Vesicles and Development
of Enhanced Extracellular Vesicle Based Cancer Vaccines. Nat. Commun..

[333] Griesinger F., Eberhardt W., Nusch A., Reiser M., Zahn M.-O., Maintz C., Bernhardt C., Losem C., Stenzinger A., Heukamp L. C. (2021). Biomarker Testing
in Non-Small Cell Lung Cancer in Routine Care: Analysis of the First
3,717 Patients in the German Prospective, Observational, Nation-Wide
Crisp Registry (Aio-Trk-0315). Lung Cancer.

[334] Srivastava S., Jayaswal N., Kumar S., Sharma P. K., Behl T., Khalid A., Mohan S., Najmi A., Zoghebi K., Alhazmi H. A. (2024). Unveiling the Potential
of Proteomic
and Genetic Signatures for Precision Therapeutics in Lung Cancer Management. Cellular Signalling.

[335] Padinharayil H., George A. (2024). Small Extracellular Vesicles: Multi-Functional
Aspects in Non-Small Cell Lung Carcinoma. Critical
Reviews in Oncology/Hematology.

[336] Vaclova T., Grazini U., Ward L., O’Neill D., Markovets A., Huang X., Chmielecki J., Hartmaier R., Thress K. S., Smith P. D. (2021). Clinical Impact
of Subclonal Egfr T790m Mutations in Advanced-Stage Egfr-Mutant Non-Small-Cell
Lung Cancers. Nat. Commun..

[337] Maher M. H., Treekitkarnmongkol W., Ghatak S., Dai J., Liu S., Nguyen T., Duose D. Y., Kim M. P., Hu T. Y., Hurd M. W. (2025). An Integrated Multi-Omics Biomarker Approach Using
Molecular Profiling and Micrornas for Evaluation of Pancreatic Cyst
Fluid. Cancer Cytopathol..

[338] Passaro A., Jänne P. A., Mok T., Peters S. (2021). Overcoming
Therapy Resistance in Egfr-Mutant Lung Cancer. Nature Cancer.

[339] Li X., Wu F. (2023). Mesenchymal
Stem Cell-Derived Extracellular Vesicles
Transfer Mir-598 to Inhibit the Growth and Metastasis of Non-Small-Cell
Lung Cancer by Targeting Thbs2. Cell Death Discovery.

[340] Saadh M. J., Mahdi M. S., Allela O. Q. B., Alazzawi T. S., ubaid M., Rakhimov N. M., Athab Z. H., Ramaiah P., Chinnasamy L., Alsaikhan F., Farhood B. (2024). Critical Role of Mir-21/Exosomal
Mir-21 in Autophagy Pathway. Pathol., Res. Pract..

[341] Wang Q.-M., Lian G.-Y., Sheng S.-M., Xu J., Ye L.-L., Min C., Guo S.-F. (2024). Exosomal Lncrna
Neat1 Inhibits Nk-Cell Activity to Promote Multiple Myeloma Cell Immune
Escape Via an Ezh2/Pbx1 Axis. Molecular Cancer
Research.

[342] Ma X., Chen Z., Chen W., Chen Z., Shang Y., Zhao Y., Li L., Zhou C., He J., Meng X. (2024). Lncrna Al139294. 1
Can Be Transported by Extracellular Vesicles to
Promote the Oncogenic Behaviour of Recipient Cells through Activation
of the Wnt and Nf-Κb2 Pathways in Non-Small-Cell Lung Cancer. J. Exp. Clin. Cancer Res..

[343] Qian X., Yang J., Qiu Q., Li X., Jiang C., Li J., Dong L., Ying K., Lu B., Chen E. (2021). Lcat3, a Novel M6a-Regulated Long Non-Coding Rna, Plays
an Oncogenic Role in Lung Cancer Via Binding with Fubp1 to Activate
C-Myc. J. Hematol. Oncol..

[344] Zygulska A. L., Pierzchalski P. (2022). Novel Diagnostic Biomarkers in Colorectal
Cancer. International journal of molecular sciences.

[345] Ferlizza E., Solmi R., Sgarzi M., Ricciardiello L., Lauriola M. (2021). The Roadmap of Colorectal Cancer
Screening. Cancers.

[346] Brocco D., Simeone P., Buca D., Marino P. D., De Tursi M., Grassadonia A., De Lellis L., Martino M. T., Veschi S., Iezzi M. (2022). Blood
Circulating Cd133+ Extracellular Vesicles Predict Clinical Outcomes
in Patients with Metastatic Colorectal Cancer. Cancers.

[347] Glass S. E., Coffey R. J. (2022). Recent Advances
in the Study of Extracellular
Vesicles in Colorectal Cancer. Gastroenterology.

[348] Xie J., Xing S., Jiang H., Zhang J., Li D., Niu S., Huang Z., Yin H. (2024). Extracellular Vesicles-Derived Cxcl4
Is a Candidate Serum Tumor Biomarker for Colorectal Cancer. iScience.

[349] Huang Z., Deng C., Ma C., He G., Tao J., Zhang L., Hu X., Mo Y., Qiu L., Zhang N. (2024). Identification and Validation of the Surface Proteins
Fibg, Pdgf-Β,
and Tgf-Β on Serum Extracellular Vesicles for Non-Invasive Detection
of Colorectal Cancer: Experimental Study. Int.
J. Surg..

[350] Datta B., Paul D., Dey T., Pal S., Rakshit T. (2022). Importance of Extracellular Vesicle Derived Rnas as
Critical Colorectal Cancer Biomarkers. ACS bio
& med Chem. Au.

[351] Du M., Gu D., Xin J., Peters U., Song M., Cai G., Li S., Ben S., Meng Y., Chu H. (2023). Integrated Multi-Omics
Approach to Distinct Molecular Characterization
and Classification of Early-Onset Colorectal Cancer. Cell Reports Medicine.

[352] Rathore M., Zhang W., Wright M. l., Bhattacharya R., Fan F., Vaziri-Gohar A., Winter J., Wang Z., Markowitz S. D., Willis J. (2022). Liver Endothelium Promotes Her3-Mediated Cell Survival
in Colorectal Cancer with Wild-Type and Mutant Kras. Molecular Cancer Research.

[353] Hinger S. A., Abner J. J., Franklin J. L., Jeppesen D. K., Coffey R. J., Patton J. G. (2020). Rab13 Regulates
Sev Secretion in
Mutant Kras Colorectal Cancer Cells. Sci. Rep..

[354] Cooks T., Pateras I. S., Jenkins L. M., Patel K. M., Robles A. I., Morris J., Forshew T., Appella E., Gorgoulis V. G., Harris C. C. (2018). Mutant P53 Cancers Reprogram Macrophages
to Tumor Supporting Macrophages Via Exosomal Mir-1246. Nat. Commun..

[355] Selven H., Andersen S., Pedersen M. I., Lombardi A. P. G., Busund L.-T. R., Kilvær T. K. (2022). High Expression
of Mir-17-5p and
Mir-20a-5p Predicts Favorable Disease-Specific Survival in Stage I-III
Colon Cancer. Sci. Rep..

[356] Canning A. J., Chen X., Li J. Q., Jeck W. R., Wang H.-N., Vo-Dinh T. (2023). Mirna Probe Integrated Biosensor
Platform Using Bimetallic Nanostars for Amplification-Free Multiplexed
Detection of Circulating Colorectal Cancer Biomarkers in Clinical
Samples. Biosens. Bioelectron..

[357] Okuno K., Kandimalla R., Mendiola M., Balaguer F., Bujanda L., Fernandez-Martos C., Aparicio J., Feliu J., Tokunaga M., Kinugasa Y. (2023). A MicroRNA
Signature for Risk-Stratification
and Response Prediction to Folfox-Based Adjuvant Therapy in Stage
II and III Colorectal Cancer. Mol. Cancer.

[358] Liu Y., Yin Z., Lu P., Ma Y., Luo B., Xiang L., Zhang W., He Y., Liang X. (2020). Lung Carcinoma
Cells Secrete Exosomal Malat1 to Inhibit Dendritic Cell Phagocytosis,
Inflammatory Response, Costimulatory Molecule Expression and Promote
Dendritic Cell Autophagy Via Akt/Mtor Pathway. OncoTargets and therapy.

[359] Huang L., Yang G., Shao Y., Sun J., Yang X., Hong H., Aikemu B., Yesseyeva G., Li S., Ding C. (2024). Cancer-Derived Exosomal Lncrna Snhg3 Promotes the Metastasis
of Colorectal Cancer through Hnrnpc-Mediating Rna Stability of Β-Catenin. Int. J. Biol. Sci..

[360] Tran H. L., Dega N. K., Lu S.-M., Huang Y.-F., Doong R.-a. (2022). Ultrasensitive Detection of Breast
Cancer Cells with
a Lectin-Based Electrochemical Sensor Using N-Doped Graphene Quantum
Dots as the Sensing Probe. Sens. Actuators,
B.

[361] Amrollahi P., Rodrigues M., Lyon C. J., Goel A., Han H., Hu T. Y. (2019). Ultra-Sensitive
Automated Profiling of EpCAM Expression
on Tumor-Derived Extracellular Vesicles. Front.
Genet..

[362] Lan M., Ren Z., Cheng C., Li G., Yang F. (2024). Small Extracellular
Vesicles Detection Using Dielectrophoresis-Based Microfluidic Chip
for Diagnosis of Breast Cancer. Biosens. Bioelectron..

[363] Na B., Yu X., Withers T., Gilleran J., Yao M., Foo T. K., Chen C., Moore D., Lin Y., Kimball S. D. (2019). Therapeutic Targeting
of Brca1 and Tp53 Mutant Breast
Cancer through Mutant P53 Reactivation. NPJ
Breast Cancer.

[364] Witek M. A., Soper S. A., Godwin A. K. (2023). Changing
the Paradigm
in Prognostic Breast Cancer Testing Based on Extracellular Vesicles. Res. J. Biol..

[365] Shi J., Zhang H., Cui Y., Xing J., Wang W., Chen J., Wang S., Yang Z. (2024). Extracellular Vesicles
for Breast Cancer Diagnosis and Therapy. Extracellular
Vesicle.

[366] Cao Y., Yu X., Zeng T., Fu Z., Zhao Y., Nie B., Zhao J., Yin Y., Li G. (2022). Molecular Characterization
of Exosomes for Subtype-Based Diagnosis of Breast Cancer. J. Am. Chem. Soc..

[367] Yang Z., LaRiviere M. J., Ko J., Till J. E., Christensen T., Yee S. S., Black T. A., Tien K., Lin A., Shen H. (2020). A Multianalyte Panel Consisting of Extracellular
Vesicle Mirnas and Mrnas, Cfdna, and Ca19–9 Shows Utility for
Diagnosis and Staging of Pancreatic Ductal Adenocarcinoma. Clin. Cancer Res..

[368] Dong H., Wang W., Chen R., Zhang Y., Zou K., Ye M., He X., Zhang F., Han J. (2018). Exosome-Mediated
Transfer of Lncrna-Snhg14 Promotes Trastuzumab Chemoresistance in
Breast Cancer. Int. J. Oncol..

[369] Cheng C. A., Hou K. C., Hsu C. W., Chiang L. C. (2024). Ultrasensitive
and High-Resolution Protein Spatially Decoding Framework for Tumor
Extracellular Vesicles. Adv. Sci..

[370] Ferguson S., Yang K. S., Zelga P., Liss A. S., Carlson J. C., Del Castillo C. F., Weissleder R. (2022). Single-Ev
Analysis (Seva) of Mutated Proteins Allows Detection of Stage 1 Pancreatic
Cancer. Sci. Adv..

[371] Huynh T. V., Tran H. L., Thi Ngoc Anh N., Doong R.-A. (2024). Electrochemical Sensor for Rapid Diagnosis and Early-Stage
Detection of Pancreatic Cancer Using Er-Gqds Decorated Mos2 Nanoflowers. Sens. Actuators, B.

[372] Treekitkarnmongkol W., Dai J., Liu S., Sankaran D., Nguyen T., Balasenthil S., Hurd M. W., Chen M., Katayama H., Roy-Chowdhuri S. (2024). Blood-Based Microrna
Biomarker Signature of Early-Stage Pancreatic Ductal Adenocarcinoma
with Lead-Time Trajectory in Prediagnostic Samples. Gastro Hep Adv..

[373] Sun D., Zhao Z., Spiegel S., Liu Y., Fan J., Amrollahi P., Hu J., Lyon C. J., Wan M., Hu T. Y. (2021). Dye-Free Spectrophotometric Measurement of Nucleic Acid-to-Protein
Ratio for Cell-Selective Extracellular Vesicle Discrimination. Biosens Bioelectron.

[374] Yu Z., Yang Y., Fang W., Hu P., Liu Y., Shi J. (2023). Dual Tumor Exosome Biomarker Co-Recognitions Based
Nanoliquid Biopsy
for the Accurate Early Diagnosis of Pancreatic Cancer. ACS Nano.

[375] Rodrigues M., Richards N., Ning B., Lyon C. J., Hu T. Y. (2019). Rapid Lipid-Based Approach for Normalization of Quantum-Dot-Detected
Biomarker Expression on Extracellular Vesicles in Complex Biological
Samples. Nano Lett..

[376] Fan J., Wei Q., Koay E. J., Liu Y., Ning B., Bernard P. W., Zhang N., Han H., Katz M. H., Zhao Z. (2018). Chemoresistance Transmission
Via Exosome-Mediated Epha2
Transfer in Pancreatic Cancer. Theranostics.

[377] Kashiro A., Kobayashi M., Oh T., Miyamoto M., Atsumi J., Nagashima K., Takeuchi K., Nara S., Hijioka S., Morizane C. (2024). Clinical Development
of a Blood Biomarker Using Apolipoprotein-A2 Isoforms for Early Detection
of Pancreatic Cancer. Journal of Gastroenterology.

[378] He J., Long J., Zhai C., Xu J., Bao K., Su W., Jiang L., Shen G., Ding X. (2024). Codetection of Proteins
and Rnas on Extracellular Vesicles for Pancreatic Cancer Early Diagnosis. Anal. Chem..

[379] Liu D. S., Puik J. R., Patel B. Y., Veno̷ M. T., Vahabi M., Prado M. M., Webber J. P., Rees E., Upton F. M., Bennett K. (2024). Unlocking the Diagnostic Power of
Plasma Extracellular Vesicle Mir-200 Family in Pancreatic Ductal Adenocarcinoma. J. Exp. Clin. Cancer Res..

[380] Deng Z., Zhao Z., Ning B., Basilio J., Mann K., Fu J., Gu Y., Ye Y., Wu X., Fan J. (2019). Nanotrap-Enabled Quantification
of Kras-Induced
Peptide Hydroxylation in Blood for Cancer Early Detection. Nano Research.

[381] Ko J., Bhagwat N., Yee S. S., Ortiz N., Sahmoud A., Black T., Aiello N. M., McKenzie L., O’Hara M., Redlinger C. (2017). Combining Machine Learning
and Nanofluidic
Technology to Diagnose Pancreatic Cancer Using Exosomes. ACS Nano.

[382] Grimaldi A. M., Salvatore M., Cavaliere C. (2023). Diagnostic
and Prognostic Significance of Extracellular Vesicles in Prostate
Cancer Drug Resistance: A Systematic Review of the Literature. Prostate Cancer and Prostatic Diseases.

[383] Tran H. L., Darmanto W., Doong R.-A. (2021). Electrochemical
Immunosensor for Ultra-Sensitive Detection of Attomolar Prostate Specific
Antigen with Sulfur-Doped Graphene Quantum Dot@ Gold Nanostar as the
Probe. Electrochim. Acta.

[384] Roberts M. J., Morton A., Donato P., Kyle S., Pattison D. A., Thomas P., Coughlin G., Esler R., Dunglison N., Gardiner R. A. (2021). (68)­Ga-Psma
Pet/Ct Tumour
Intensity Pre-Operatively Predicts Adverse Pathological Outcomes and
Progression-Free Survival in Localised Prostate Cancer. Eur. J. Nucl. Med. Mol. Imaging.

[385] Padda R. S., Deng F. K., Brett S. I., Biggs C. N., Durfee P. N., Brinker C. J., Williams K. C., Leong H. S. (2019). Nanoscale
Flow Cytometry to Distinguish Subpopulations of Prostate Extracellular
Vesicles in Patient Plasma. Prostate.

[386] Khanna K., Salmond N., Lynn K. S., Leong H. S., Williams K. C. (2021). Clinical Significance of Steap1 Extracellular
Vesicles
in Prostate Cancer. Prostate cancer and prostatic
diseases.

[387] Peng Q., Chiu P. K.-F., Wong C. Y.-P., Cheng C. K.-L., Teoh J. Y.-C., Ng C.-F. (2021). Identification of PiRNA Targets in
Urinary Extracellular Vesicles for the Diagnosis of Prostate Cancer. Diagnostics.

[388] Logozzi M., Angelini D. F., Giuliani A., Mizzoni D., Di Raimo R., Maggi M., Gentilucci A., Marzio V., Salciccia S., Borsellino G. (2019). Increased Plasmatic Levels of Psa-Expressing Exosomes Distinguish
Prostate Cancer Patients from Benign Prostatic Hyperplasia: A Prospective
Study. Cancers.

[389] Wang J. J., Sun N., Lee Y.-T., Kim M., Vagner T., Rohena-Rivera K., Wang Z., Chen Z., Zhang R. Y., Lee J. (2023). Prostate Cancer Extracellular
Vesicle Digital Scoring Assay–a Rapid Noninvasive Approach
for Quantification of Disease-Relevant Mrnas. Nano today.

[390] Greten T. F., Villanueva A., Korangy F., Ruf B., Yarchoan M., Ma L., Ruppin E., Wang X. W. (2023). Biomarkers
for Immunotherapy of Hepatocellular Carcinoma. Nature Reviews Clinical Oncology.

[391] Min Y., Deng W., Yuan H., Zhu D., Zhao R., Zhang P., Xue J., Yuan Z., Zhang T., Jiang Y. (2024). Single Extracellular Vesicle Surface
Protein-Based Blood Assay Identifies
Potential Biomarkers for Detection and Screening of Five Cancers. Mol. Oncol..

[392] Sun N., Zhang C., Lee Y. T., Tran B. V., Wang J., Kim H., Lee J., Zhang R. Y., Wang J. J., Hu J. (2023). Hcc Ev Ecg
Score: An Extracellular Vesicle-Based Protein Assay for Detection
of Early-Stage Hepatocellular Carcinoma. Hepatology.

[393] Xu C., Xu Z., Zhang Y., Evert M., Calvisi D. F., Chen X. (2022). Β-Catenin Signaling in Hepatocellular Carcinoma. J. Clin. Invest..

[394] Boonkaew B., Satthawiwat N., Pinjaroen N., Chuaypen N., Tangkijvanich P. (2023). Circulating
Extracellular Vesicle-Derived
Micrornas as Novel Diagnostic and Prognostic Biomarkers for Non-Viral-Related
Hepatocellular Carcinoma. International Journal
of Molecular Sciences.

[395] Kim S. S., Baek G. O., Ahn H. R., Sung S., Seo C. W., Cho H. J., Nam S. W., Cheong J. Y., Eun J. W. (2020). Serum Small
Extracellular Vesicle-Derived Linc00853
as a Novel Diagnostic Marker for Early Hepatocellular Carcinoma. Molecular oncology.

[396] Wang J. H., Bai Z. z., Niu X. d., Zhu C. l., Liang T., Hu Y. l., Gao Z. H., Da M. X. (2024). Serum Extracellular
Vesicle-Derived Mir-21–5p and Mir-26a-5p as Non-Invasive Diagnostic
Potential Biomarkers for Gastric Cancer: A Preliminary Study. Int. J. Biol. Markers.

[397] Xiao K., Dong Z., Wang D., Liu M., Ding J., Chen W., Shang Z., Yue C., Zhang Y. (2021). Clinical Value of Lncrna Ccat1 in Serum Extracellular Vesicles as
a Potential Biomarker for Gastric Cancer. Oncol.
Lett..

[398] Trinidad C. V., Tetlow A. L., Bantis L. E., Godwin A. K. (2020). Reducing
Ovarian Cancer Mortality through Early Detection: Approaches Using
Circulating Biomarkers. Cancer Prev Res. (Phila).

[399] Sharma T., Nisar S., Masoodi T., Macha M. A., Uddin S., Akil A. A.-S., Pandita T. K., Singh M., Bhat A. A. (2023). Current and Emerging Biomarkers in
Ovarian Cancer Diagnosis;
Ca125 and Beyond. Advances in protein chemistry
and structural biology.

[400] Jo A., Green A., Medina J. E., Iyer S., Ohman A. W., McCarthy E. T., Reinhardt F., Gerton T., Demehin D., Mishra R. (2023). Inaugurating High-Throughput
Profiling of Extracellular
Vesicles for Earlier Ovarian Cancer Detection. Adv. Sci..

[401] Li L., Zhang F., Zhang J., Shi X., Wu H., Chao X., Ma S., Lang J., Wu M., Zhang D. (2023). Identifying Serum Small Extracellular Vesicle Microrna
as a Noninvasive
Diagnostic and Prognostic Biomarker for Ovarian Cancer. ACS Nano.

[402] Trinidad C. V., Pathak H. B., Cheng S., Tzeng S. C., Madan R., Sardiu M. E., Bantis L. E., Deighan C., Jewell A., Rayamajhi S. (2023). Lineage Specific
Extracellular Vesicle-Associated
Protein Biomarkers for the Early Detection of High Grade Serous Ovarian
Cancer. Sci. Rep..

[403] May W. A., Gishizky M. L., Lessnick S. L., Lunsford L. B., Lewis B. C., Delattre O., Zucman J., Thomas G., Denny C. T. (1993). Ewing Sarcoma
11;22 Translocation Produces a Chimeric
Transcription Factor That Requires the DNA-Binding Domain Encoded
by Fli1 for Transformation. Proc. Natl. Acad.
Sci. U. S. A..

[404] Samuel G., Crow J., Klein J. B., Merchant M. L., Nissen E., Koestler D. C., Laurence K., Liang X., Neville K., Staggs V. (2020). Ewing Sarcoma Family
of Tumors-Derived Small Extracellular Vesicle Proteomics Identify
Potential Clinical Biomarkers. Oncotarget.

[405] Zhang P., Samuel G., Crow J., Godwin A. K., Zeng Y. (2018). Molecular Assessment of Circulating
Exosomes toward Liquid Biopsy
Diagnosis of Ewing Sarcoma Family of Tumors. Transl Res..

[406] Turaga S. M., Sardiu M. E., Vishwakarma V., Mitra A., Bantis L. E., Madan R., Merchant M. L., Klein J. B., Samuel G., Godwin A. K. (2023). Identification of
Small Extracellular Vesicle Protein Biomarkers for Pediatric Ewing
Sarcoma. Front. Mol. Biosci..

[407] Crow J., Samuel G., Farrow E., Gibson M., Johnston J., Guest E., Miller N., Pei D., Koestler D., Pathak H., Liang X., Mangels C., Godwin A. K. (2022). Microrna Content of Ewing Sarcoma Derived Extracellular
Vesicles Leads to Biomarker Potential and Identification of a Previously
Undocumented Ews-Fli1 Translocation. Biomarker
Insights.

[408] Markopoulos G., Lampri E., Tragani I., Kourkoumelis N., Vartholomatos G., Seretis K. (2024). Intraoperative Flow
Cytometry for
the Rapid Diagnosis and Validation of Surgical Clearance of Non-Melanoma
Skin Cancer: A Prospective Clinical Feasibility Study. Cancers.

[409] Wang J., Wuethrich A., Sina A. A. I., Lane R. E., Lin L. L., Wang Y., Cebon J., Behren A., Trau M. (2020). Tracking Extracellular
Vesicle Phenotypic Changes Enables Treatment
Monitoring in Melanoma. Sci. Adv..

[410] Guo Y., Zhang X., Wang L., Li M., Shen M., Zhou Z., Zhu S., Li K., Fang Z., Yan B. (2021). The Plasma Exosomal Mir-1180-3p Serves as a Novel Potential
Diagnostic Marker for Cutaneous Melanoma. Cancer
Cell Int..

[411] Zhu J., Huang J., Sun Y., Xu W., Qian H. (2024). Emerging Role
of Extracellular Vesicles in Diabetic Retinopathy. Theranostics.

[412] Sharma P., Roy A., Dhamija R. K., Bhushan S., Baswal K., Kulandaisamy R., Yadav S., Kumar S., Inampudi K. K. (2024). A Comprehensive
Proteomic Profiling of Urinary Exosomes
and the Identification of Early Non-Invasive Biomarker in Patients
with Coronary Artery Disease. Journal of Proteomics.

[413] Qian L., Zhao Q., Yu P., Lü J., Guo Y., Gong X., Ding Y., Yu S., Fan L., Fan H. (2022). Diagnostic Potential
of a Circulating Mirna Model Associated
with Therapeutic Effect in Heart Failure. J.
Transl. Med..

[414] Pasta S., Agnese V., Gallo A., Cosentino F., Di Giuseppe M., Gentile G., Raffa G. M., Maalouf J. F., Michelena H. I., Bellavia D. (2020). Shear
Stress and Aortic
Strain Associations with Biomarkers of Ascending Thoracic Aortic Aneurysm. Annals of thoracic surgery.

[415] Dreyer R., Murugiah K., Nuti S. V., Dharmarajan K., Chen S. I., Chen R., Wayda B., Ranasinghe I. (2014). Most Important
Outcomes Research Papers on Stroke and Transient Ischemic Attack. Circulation: Cardiovascular Quality and Outcomes.

[416] Yilmaz G., Arumugam T. V., Stokes K. Y., Granger D. N. (2006). Role of
T Lymphocytes and Interferon-Γ in Ischemic Stroke. Circulation.

[417] Yang J., Tan C., Wang Y., Zong T., Xie T., Yang Q., Wu M., Liu Y., Mu T., Wang X. (2023). The Circrna Mkln1 Regulates
Autophagy in the Development
of Diabetic Retinopathy. Biochimica et Biophysica
Acta (BBA)-Molecular Basis of Disease.

[418] Fu X., Mishra R., Chen L., Arfat M. Y., Sharma S., Kingsbury T., Gunasekaran M., Saha P., Hong C., Yang P. (2023). Exosomes Mediated Fibrogenesis in Dilated Cardiomyopathy
through a Microrna Pathway. Iscience.

[419] Rogers M. A., Atkins S. K., Zheng K. H., Singh S. A., Chelvanambi S., Pham T. H., Kuraoka S., Stroes E. S. G., Aikawa M., Aikawa E. (2022). Lipoprotein­(a) Induces
Vesicular
Cardiovascular Calcification Revealed with Single-Extracellular Vesicle
Analysis. Front Cardiovasc Med..

[420] Rogers M. A., Buffolo F., Schlotter F., Atkins S. K., Lee L. H., Halu A., Blaser M. C., Tsolaki E., Higashi H., Luther K. (2020). Annexin A1–Dependent
Tethering Promotes Extracellular Vesicle Aggregation Revealed with
Single-Extracellular Vesicle Analysis. Sci.
Adv..

[421] Pei J., Palanisamy C. P., Jayaraman S., Natarajan P. M., Umapathy V. R., Roy J. R., Thalamati D., Ahalliya R. M., Kanniappan G. V., Mironescu M. (2024). Proteomics
Profiling of Extracellular Vesicle for Identification of Potential
Biomarkers in Alzheimer’s Disease: A Comprehensive Review. Ageing Research Reviews.

[422] Muraoka S., DeLeo A. M., Sethi M. K., Yukawa-Takamatsu K., Yang Z., Ko J., Hogan J. D., Ruan Z., You Y., Wang Y. (2020). Proteomic and Biological Profiling of Extracellular
Vesicles from Alzheimer’s Disease Human Brain Tissues. Alzheimers Dement.

[423] Sun Y., Hefu Z., Li B., Lifang W., Zhijie S., Zhou L., Deng Y., Zhili L., Ding J., Li T. (2023). Plasma Extracellular Vesicle Microrna Analysis of Alzheimer’s
Disease Reveals Dysfunction of a Neural Correlation Network. Research.

[424] Cai Y., Chen T., Cai Y., Liu J., Yu B., Fan Y., Su J., Zeng Y., Xiao X., Ren L. (2024). Surface Protein
Profiling and Subtyping of Extracellular Vesicles in Body Fluids Reveals
Non-Csf Biomarkers of Alzheimer’s Disease. J. Extracell. Vesicles.

[425] Li D., Zou S., Huang Z., Sun C., Liu G. (2024). Isolation
and Quantification of L1cam-Positive Extracellular Vesicles on a Chip
as a Potential Biomarker for Parkinson’s Disease. J. Extracell. Vesicles.

[426] Pulliam L., Sun B., McCafferty E., Soper S. A., Witek M. A., Hu M., Ford J. M., Song S., Kapogiannis D., Glesby M. J. (2024). Microfluidic Isolation
of Neuronal-Enriched Extracellular Vesicles Shows Distinct and Common
Neurological Proteins in Long Covid, Hiv Infection and Alzheimer’s
Disease. Int. J. Mol. Sci..

[427] Izco M., Carlos E., Alvarez-Erviti L. (2022). The Two Faces
of Exosomes in Parkinson’s Disease: From Pathology to Therapy. Neuroscientist.

[428] Li Y., Cao Y., Liu W., Chen F., Zhang H., Zhou H., Zhao A., Luo N., Liu J., Wu L. (2024). Candidate Biomarkers of Ev-Microrna in Detecting Rem
Sleep Behavior
Disorder and Parkinson’s Disease. npj
Parkinson’s Dis..

[429] Chatterjee M., Özdemir S., Fritz C., Möbius W., Kleineidam L., Mandelkow E., Biernat J., Doğdu C., Peters O., Cosma N. C. (2024). Plasma Extracellular
Vesicle Tau and Tdp-43 as Diagnostic Biomarkers in Ftd and Als. Nat. Med..

[430] Hallal S. M., Tűzesi Á., Sida L. A., Xian E., Madani D., Muralidharan K., Shivalingam B., Buckland M. E., Satgunaseelan L., Alexander K. L. (2024). Glioblastoma
Biomarkers in Urinary Extracellular Vesicles Reveal the Potential
for a ‘Liquid Gold’biopsy. Br.
J. Cancer.

[431] Neueder A., Nitzschner P., Wagner R., Hummel J., Hoschek F., Wagner M., Abdelmoez A., von Einem B., Landwehrmeyer G. B., Tabrizi S. J. (2024). Huntington
Disease Alters the Actionable Information in Plasma Extracellular
Vesicles. Clin. Transl. Med..

[432] Tao Y., Wei X., Yue Y., Wang J., Li J., Shen L., Lu G., He Y., Zhao S., Zhao F. (2021). Extracellular Vesicle-Derived
Aebp1Mrna as a Novel Candidate Biomarker
for Diabetic Kidney Disease. J. Transl. Med..

[433] Komatsu S., Kato N., Kitai H., Funahashi Y., Noda Y., Tsubota S., Tanaka A., Sato Y., Maeda K., Saito S. (2024). Detecting and Exploring
Kidney-Derived Extracellular Vesicles in Plasma. Clin Exp Nephrol.

[434] Ali H., Malik M. Z., Abu-Farha M., Abubaker J., Cherian P., Nizam R., Jacob S., Bahbahani Y., Naim M., Ahmad S. (2024). Global Analysis of
Urinary Extracellular
Vesicle Small Rnas in Autosomal Dominant Polycystic Kidney Disease. J. Gene Med..

[435] Nakao Y., Amrollahi P., Parthasarathy G., Mauer A. S., Sehrawat T. S., Vanderboom P., Nair K. S., Nakao K., Allen A. M., Hu T. Y. (2021). Circulating Extracellular Vesicles Are a Biomarker for Nafld Resolution
and Response to Weight Loss Surgery. Nanomedicine.

[436] Hu T., Liu C. H., Zheng Y., Ji J., Zheng Y., He S. K., Wu D., Jiang W., Zeng Q., Zhang N. (2024). Mirnas in Patients with
Alcoholic Liver Disease: A
Systematic Review and Meta-Analysis. Expert
Rev. Gastroenterol Hepatol.

[437] Yoshida M., Matsuzaki J., Fujita K., Kimura M., Umezu T., Tokuda N., Yamaguchi T., Kuroda M., Ochiya T., Saito Y., Kimura K. (2024). Plasma Extracellular
Vesicle Micrornas Reflecting the Therapeutic Effect of the Cbp/Β-Catenin
Inhibitor Pri-724 in Patients with Liver Cirrhosis. Sci. Rep..

[438] Sakane S., Hikita H., Shirai K., Sakamoto T., Narumi R., Adachi J., Kakita N., Yamada Y., Toyoda H., Takahashi H. (2024). Proteomic
Analysis of Serum Extracellular
Vesicles Reveals Fibulin-3 as a New Marker Predicting Liver-Related
Events in Masld. Hepatol. Commun..

[439] Hu Q., Lyon C. J., Fletcher J. K., Tang W., Wan M., Hu T. Y. (2021). Extracellular Vesicle Activities Regulating Macrophage-
and Tissue-Mediated
Injury and Repair Responses. Acta Pharm. Sin
B.

[440] Nieri D., Morani C., De Francesco M., Gaeta R., Niceforo M., De Santis M., Giusti I., Dolo V., Daniele M., Papi A. (2024). Enhanced Prothrombotic and Proinflammatory Activity of Circulating
Extracellular Vesicles in Acute Exacerbations of Chronic Obstructive
Pulmonary Disease. Respir Med..

[441] Liu S., Tan X., Liu S. (2024). The Role of
Extracellular Vesicles
in Copd and Potential Clinical Value. Respir.
Res..

[442] Soccio P., Moriondo G., Lacedonia D., Tondo P., Quarato C. M. I., Foschino Barbaro M. P., Scioscia G. (2022). Evs-Mirna: The New Molecular Markers for Chronic Respiratory
Diseases. Life.

[443] Jain S., Nehra M., Kumar R., Dilbaghi N., Hu T., Kumar S., Kaushik A., Li C. Z. (2021). Internet of Medical
Things (Iomt)-Integrated Biosensors for Point-of-Care Testing of Infectious
Diseases. Biosens Bioelectron.

[444] Liao H., Lyon C. J., Ying B., Hu T. (2024). Climate Change,
Its Impact on Emerging Infectious Diseases and New Technologies to
Combat the Challenge. Emerging Microbes Infect..

[445] Huang Z., Lyon C. J., Wang J., Lu S., Hu T. Y. (2023). Crispr Assays for Disease Diagnosis: Progress to and
Barriers Remaining
for Clinical Applications. Adv. Sci. (Weinh).

[446] Reynolds D. E., Pan M., Yang J., Galanis G., Roh Y. H., Morales R. T., Kumar S. S., Heo S. J., Xu X., Guo W. (2023). Double Digital Assay for Single Extracellular Vesicle
and Single Molecule Detection. Adv. Sci. (Weinh).

[447] Molnar S. M., Kim Y., Wieczorek L., Williams A., Patil K. A., Khatkar P., Santos M. F., Mensah G., Lorico A., Polonis V. R., Kashanchi F. (2024). Extracellular
Vesicle Isolation Methods Identify Distinct Hiv-1 Particles Released
from Chronically Infected T-Cells. J. Extracell.
Vesicles.

[448] Suades R., Greco M. F., Prieto P., Padró T., Devaux Y., Domingo P., Badimon L. (2024). Cd66b+/Cd68+
Circulating
Extracellular Vesicles, Lactate Dehydrogenase and Neutrophil-to-Lymphocyte
Ratio Can Differentiate Coronavirus Disease 2019 Severity During and
after Infection. J. Extracell. Vesicles.

[449] Ning B., Youngquist B. M., Li D. D., Lyon C. J., Zelazny A., Maness N. J., Tian D., Hu T. Y. (2022). Rapid Detection
of Multiple Sars-Cov-2 Variants of Concern by Pam-Targeting Mutations. Cell Rep. Methods.

[450] Hoffmann M., Kleine-Weber H., Schroeder S., Krüger N., Herrler T., Erichsen S., Schiergens T. S., Herrler G., Wu N. H., Nitsche A., Müller M. A., Drosten C., Pöhlmann S. (2020). Sars-Cov-2 Cell Entry
Depends on Ace2 and Tmprss2 and Is Blocked by a Clinically Proven
Protease Inhibitor. Cell.

[451] Cacciottolo M., Li Y., Nice J. B., LeClaire M. J., Twaddle R., Mora C. L., Adachi S. Y., Young M., Angeles J., Elliott K. (2023). Nanograms of Sars-Cov-2 Spike Protein
Delivered by Exosomes Induce Potent Neutralization of Both Delta and
Omicron Variants. PLoS One.

[452] Tu B., Pan Z., Wang H., Qu J., Sun F., Shi M., Zhang Y., Wu H., Muhitdinov B., Huang Y. (2024). Heparin-Conjugated Ace2-Bearing Extracellular Vesicles as Engineered
Dual-Decoy for Combating the Sars-Cov-2 Omicron Variant Via Pulmonary
Delivery. ACS Materials Letters.

[453] Huang F., Bai J., Zhang J., Yang D., Fan H., Huang L., Shi T., Lu G. (2019). Identification of Potential
Diagnostic Biomarkers for Pneumonia Caused by Adenovirus Infection
in Children by Screening Serum Exosomal Micrornas. Mol. Med. Rep..

[454] Dahiya B., Khan A., Mor P., Kamra E., Singh N., Gupta K. B., Sheoran A., Sreenivas V., Mehta P. K. (2019). Detection of Mycobacterium Tuberculosis Lipoarabinomannan
and Cfp-10 (Rv3874) from Urinary Extracellular Vesicles of Tuberculosis
Patients by Immuno-Pcr. Pathogens and disease.

[455] Li L., Mao L., van der
Zalm M. M., Olivo J., Liu S., Vergara C., Palmer M., Shu Q., Demers A. M., Lyon C. J. (2025). Blood-Based Diagnosis of Pediatric Tuberculosis: A
Prospective Cohort Study in South Africa and Dominican Republic. J. Infect.

[456] Huang Z., Zhang G., Lyon C. J., Hu T. Y., Lu S. (2023). Outlook for Crispr-Based Tuberculosis Assays Now in Their Infancy. Front. Immunol..

[457] Sun D., Yang L., Lyon C. J., Hu T. (2020). Simulation-Directed
Amplifiable Nanoparticle Enhanced Quantitative Scattering Assay under
Low Magnification Dark Field Microscopy. J.
Mater. Chem. B.

[458] Li J., Sina A. A. I., Antaw F., Fielding D., Möller A., Lobb R., Wuethrich A., Trau M. (2023). Digital Decoding of
Single Extracellular Vesicle Phenotype Differentiates Early Malignant
and Benign Lung Lesions. Adv. Sci..

[459] Tong Z., Yang D., Shen C., Li C., Xu X., Li Q., Wu Z., Ma H., Chen F., Mao H. (2024). Rapid Automated Extracellular Vesicle Isolation and Mirna Preparation
on a Cost-Effective Digital Microfluidic Platform. Anal. Chim. Acta.

[460] Peng W., Sun D., Lu W., Yin S., Ye B., Wang X., Ren Y., Hong Z., Zhu W., Yu P., Xi J. J., Yao B. (2023). Comprehensive Detection of Pd-L1
Protein and Mrna in Tumor Cells and Extracellular Vesicles through
a Real-Time Qpcr Assay. Anal. Chem..

[461] Zhang Y.-H., Chen Y., Shi L., Han X., Xie J.-C., Chen Y., Xiang M., Li B.-W., Li J., Xing H. R. (2024). A Novel Lung Cancer Stem Cell Extracellular Vesicles
Lncrna Rollcsc Modulate Non-Stemness Cancer Cell Plasticity through
Mir-5623–3p and Mir-217–5p Targeting Lipid Metabolism. Int. J. Biol. Macromol..

[462] Park J., Park J. S., Huang C. H., Jo A., Cook K., Wang R., Lin H. Y., Van Deun J., Li H., Min J. (2021). An Integrated Magneto-Electrochemical Device
for the Rapid Profiling of Tumour Extracellular Vesicles from Blood
Plasma. Nat. Biomed Eng..

[463] Fan Q., Sun X.-H., Wu N., Wang Y.-H., Wang J.-H., Yang T. (2024). An Extracellular Vesicle
Microrna-Initiated 3d Dnazyme Motor for
Colorectal Cancer Diagnosis. Analyst.

[464] Lenart M., Siemińska I., Szatanek R., Mordel A., Szczepanik A., Rubinkiewicz M., Siedlar M., Baj-Krzyworzeka M. (2024). Identification
of Mirnas Present in Cell-and Plasma-Derived Extracellular Vesicles—Possible
Biomarkers of Colorectal Cancer. Cancers.

[465] Tian F., Zhang S., Liu C., Han Z., Liu Y., Deng J., Li Y., Wu X., Cai L., Qin L. (2021). Protein Analysis of Extracellular Vesicles
to Monitor
and Predict Therapeutic Response in Metastatic Breast Cancer. Nat. Commun..

[466] Zhang X.-W., Qi G.-X., Liu M.-X., Yang Y.-F., Wang J.-H., Yu Y.-L., Chen S. (2024). Deep Learning
Promotes
Profiling of Multiple Mirnas in Single Extracellular Vesicles for
Cancer Diagnosis. ACS sensors.

[467] Wang X., Jian Q., Zhang Z., Gu J., Wang X., Wang Y. (2024). Effect of Tumor-Derived Extracellular
Vesicle-Shuttled Lncrna Malat1 on Proliferation, Invasion and Metastasis
of Triple-Negative Breast Cancer by Regulating Macrophage M2 Polarization
Via the Postn/Hippo/Yap Axis. Translational
Oncology.

[468] Han Z., Wan F., Deng J., Zhao J., Li Y., Yang Y., Jiang Q., Ding B., Liu C., Dai B. (2021). Ultrasensitive Detection of Mrna in Extracellular Vesicles
Using DNA Tetrahedron-Based Thermophoretic Assay. Nano Today.

[469] Li Z., Li L. X., Diao Y. J., Wang J., Ye Y., Hao X. K. (2021). Identification
of Urinary Exosomal Mirnas for the Non-Invasive
Diagnosis of Prostate Cancer. Cancer Manag Res..

[470] Guo X., Peng Y., Song Q., Wei J., Wang X., Ru Y., Xu S., Cheng X., Li X., Wu D. (2023). A Liquid Biopsy Signature for the Early Detection
of Gastric Cancer
in Patients. Gastroenterology.

[471] Serratì S., Guida M., Di Fonte R., De Summa S., Strippoli S., Iacobazzi R. M., Quarta A., De Risi I., Guida G., Paradiso A., Porcelli L., Azzariti A. (2022). Circulating
Extracellular Vesicles Expressing Pd1 and Pd-L1 Predict Response and
Mediate Resistance to Checkpoint Inhibitors Immunotherapy in Metastatic
Melanoma. Mol. Cancer.

[472] Nogueras-Ortiz C. J., Eren E., Yao P., Calzada E., Dunn C., Volpert O., Delgado-Peraza F., Mustapic M., Lyashkov A., Rubio F. J. (2024). Single-Extracellular
Vesicle (Ev) Analyses Validate the Use of L1 Cell Adhesion Molecule
(L1cam) as a Reliable Biomarker of Neuron-Derived Evs. J. Extracell. Vesicles.

[473] Kumagai M., Tsuchiya A., Yang Y., Takeda N., Natsui K., Natusi Y., Tomiyoshi K., Yamazaki F., Koseki Y., Shinchi H. (2024). Fibulin-4 as a Potential
Extracellular Vesicle Marker of Fibrosis in Patients with Cirrhosis. FEBS Open Bio..

[474] Cairoli V., Valle-Millares D., Terrón-Orellano M. C., Luque D., Ryan P., Dominguez L., Martín-Carbonero L., De los Santos I., De Matteo E., Ameigeiras B. (2023). Microrna Signature
from Extracellular
Vesicles of Hcv/Hiv Co-Infected Individuals Differs from Hcv Mono-Infected. J. Mol. Med..

[475] Li L., Zhang L., Montgomery K. C., Jiang L., Lyon C. J., Hu T. Y. (2023). Advanced
Technologies for Molecular Diagnosis of Cancer: State of
Pre-Clinical Tumor-Derived Exosome Liquid Biopsies. Mater. Today Bio.

[476] Shama A., Soni T., Jawanda I. K., Upadhyay G., Sharma A., Prabha V. (2023). The Latest Developments in Using
Proteomic Biomarkers from Urine and Serum for Non-Invasive Disease
Diagnosis and Prognosis. Biomarker Insights.

[477] Jiao Y., Gao L., Zhang T., He Z., Zheng S.-Y., Liu W. (2024). Profiling DNA Cargos in Single Extracellular
Vesicles Via Hydrogel-Based Droplet Digital Multiple Displacement
Amplification. Anal. Chem..

[478] Cutshaw G., Uthaman S., Hassan N., Kothadiya S., Wen X., Bardhan R. (2023). The Emerging Role of
Raman Spectroscopy as an Omics
Approach for Metabolic Profiling and Biomarker Detection toward Precision
Medicine. Chem. Rev..

[479] Liu Y., Zhang Y., Li H., Hu T. Y. (2025). Recent Advances
in the Bench-to-Bedside Translation of Cancer Nanomedicines. Acta Pharm. Sin B.

[480] Crescitelli R., Lässer C., Lötvall J. (2021). Isolation
and Characterization of Extracellular Vesicle Subpopulations from
Tissues. Nature protocols.

[481] Saigusa D., Honda T., Iwasaki Y., Ueda K., Hishinuma E., Matsukawa N., Togashi A., Matsutani N., Seki N. (2022). Lipidomic and Metabolic
Profiling of Plasma and Plasma-Derived Extracellular
Vesicles by Uhplc-Ms/Ms. Med. Mass Spectrom..

[482] Špilak A., Brachner A., Kegler U., Neuhaus W., Noehammer C. (2021). Implications and Pitfalls for Cancer
Diagnostics Exploiting
Extracellular Vesicles. Advanced drug delivery
reviews.

[483] Patel B., Gaikwad S., Prasad S. (2024). Exploring
the Significance
of Extracellular Vesicles: Key Players in Advancing Cancer and Possible
Theranostic Tools. Cancer Pathog. Ther..

[484] Xavier C. P. R., Caires H. R., Barbosa M. A. G., Bergantim R., Guimarães J. E., Vasconcelos M. H. (2020). The Role
of Extracellular Vesicles
in the Hallmarks of Cancer and Drug Resistance. Cells.

[485] Zhang J., Wu J., Wang G., He L., Zheng Z., Wu M., Zhang Y. (2023). Extracellular Vesicles:
Techniques and Biomedical Applications Related to Single Vesicle Analysis. ACS Nano.

[486] Hu T., Brinker C. J., Chan W. C. W., Chen C., Chen X., Ho D., Kataoka K., Kotov N. A., Liz-Marzán L. M., Nel A. E. (2022). Publishing Translational Research of Nanomedicine in
Acs Nano. ACS Nano.

[487] Cheng K., Kalluri R. (2023). Guidelines for Clinical
Translation
and Commercialization of Extracellular Vesicles and Exosomes Based
Therapeutics. Extracellular Vesicle.

[488] Van der Pol E., Böing A., Gool E., Nieuwland R. (2016). Recent Developments
in the Nomenclature, Presence, Isolation, Detection and Clinical Impact
of Extracellular Vesicles. Journal of thrombosis
and haemostasis.

[489] Ansari F. J., Tafti H. A., Amanzadeh A., Rabbani S., Shokrgozar M. A., Heidari R., Behroozi J., Eyni H., Uversky V. N., Ghanbari H. (2024). Comparison of the Efficiency
of Ultrafiltration, Precipitation, and Ultracentrifugation Methods
for Exosome Isolation. Biochemistry and biophysics
reports.

[490] Yakubovich E., Polischouk A., Evtushenko V. (2022). Principles
and Problems of Exosome Isolation from Biological Fluids. Biochemistry (Moscow), Supplement Series A: Membrane and Cell
Biology.

[491] Rufo J., Zhang P., Wang Z., Gu Y., Yang K., Rich J., Chen C., Zhong R., Jin K., He Y., Xia J., Li K., Wu J., Ouyang Y., Sadovsky Y., Lee L. P., Huang T. J. (2024). High-Yield
and Rapid Isolation of Extracellular Vesicles by Flocculation
Via Orbital Acoustic Trapping: Float. Microsyst.
Nanoeng..

[492] Bagi M., Amjad F., Ghoreishian S. M., Sohrabi Shahsavari S., Huh Y. S., Moraveji M. K., Shimpalee S. (2024). Advances in
Technical Assessment of Spiral Inertial Microfluidic Devices toward
Bioparticle Separation and Profiling: A Critical Review. BioChip Journal.

[493] Ridolfi A., Conti L., Brucale M., Frigerio R., Cardellini J., Musicò A., Romano M., Zendrini A., Polito L., Bergamaschi G., Gori A., Montis C., Panella S., Barile L., Berti D., Radeghieri A., Bergese P., Cretich M., Valle F. (2023). Particle Profiling
of Ev-Lipoprotein Mixtures by Afm Nanomechanical Imaging. J. Extracell. Vesicles.

[494] Cimorelli M., Nieuwland R., Varga Z., van der
Pol E. (2021). Standardized Procedure to Measure the Size Distribution of Extracellular
Vesicles Together with Other Particles in Biofluids with Microfluidic
Resistive Pulse Sensing. PLoS One.

[495] Teles, R. H. G. ; Engelmayr, D. ; Meybohm, P. ; Burek, M. ; Isolation of Extracellular Vesicles Using Formulas to Adapt Centrifugation to Different Centrifuges. In Neuroprotection: Method and Protocols; Springer, 2024; pp 39–48. 10.1007/978-1-0716-3662-6_3 38427227

[496] Erdbrügger U., Lannigan J. (2016). Analytical Challenges of Extracellular
Vesicle Detection: A Comparison of Different Techniques. Cytometry Part A.

[497] Tiwari S., Kumar V., Randhawa S., Verma S. K. (2021). Preparation
and Characterization of Extracellular Vesicles. Am. J. Reprod. Immunol..

[498] Gul B., Syed F., Khan S., Iqbal A., Ahmad I. (2022). Characterization
of Extracellular Vesicles by Flow Cytometry: Challenges and Promises. Micron.

[499] Nguyen P. H. D., Le A. H., Pek J. S. Q., Pham T. T., Jayasinghe M. K., Do D. V., Phung C. D., Le M. T. N. (2022). Extracellular
Vesicles and Lipoproteins-Smart Messengers of Blood Cells in the Circulation. J. Extracell. Biol..

[500] Stranska R., Gysbrechts L., Wouters J., Vermeersch P., Bloch K., Dierickx D., Andrei G., Snoeck R. (2018). Comparison
of Membrane Affinity-Based Method with Size-Exclusion Chromatography
for Isolation of Exosome-Like Vesicles from Human Plasma. J. Transl. Med..

[501] Visan K. S., Wu L. Y., Voss S., Wuethrich A., Möller A. (2023). Status Quo of Extracellular Vesicle
Isolation and Detection
Methods for Clinical Utility. Semin Cancer Biol..

[502] Su Y., He W., Zheng L., Fan X., Hu T. Y. (2025). Toward
Clarity in Single Extracellular Vesicle Research: Defining the Field
and Correcting Missteps. ACS Nano.

[503] Visan K. S., Lobb R. J., Ham S., Lima L. G., Palma C., Edna C. P. Z., Wu L. Y., Gowda H., Datta K. K., Hartel G. (2022). Comparative
Analysis
of Tangential Flow Filtration and Ultracentrifugation, Both Combined
with Subsequent Size Exclusion Chromatography, for the Isolation of
Small Extracellular Vesicles. J. Extracell.
Vesicles.

[504] Qiu L., Liu X., Zhu L., Luo L., Sun N., Pei R. (2023). Current Advances in Technologies
for Single Extracellular Vesicle
Analysis and Its Clinical Applications in Cancer Diagnosis. Biosensors.

[505] Ji Y., Qi D., Li L., Su H., Li X., Luo Y., Sun B., Zhang F., Lin B., Liu T. (2019). Multiplexed Profiling of Single-Cell Extracellular
Vesicles Secretion. Proc. Natl. Acad. Sci. U.
S. A..

[506] Nikoloff J. M., Saucedo-Espinosa M. A., Kling A., Dittrich P. S. (2021). Identifying
Extracellular Vesicle Populations from Single Cells. Proc. Natl. Acad. Sci. U. S. A..

[507] Fathi M., Joseph R., Adolacion J. R. T., Martinez-Paniagua M., An X., Gabrusiewicz K., Mani S. A., Varadarajan N. (2021). Single-Cell Cloning of Breast Cancer
Cells Secreting Specific Subsets of Extracellular Vesicles. Cancers (Basel).

[508] Zhou B., Guo W., Guo L., Li Y., Zheng Z., Huai Q., Tan F., Li Y., Xue Q., Ying J. (2023). Single-Cell Rna-Sequencing Data Reveals the
Genetic Source of Extracellular Vesicles in Esophageal Squamous Cell
Carcinoma. Pharmacol. Res..

[509] Forte D., Maltoni F., Bruno S., Garcia-Gonzalez P., Cristiano G., Sartor C., Soverini S., Catani L., Argüello R. J., Cavo M. (2023). Single-Cell
Metabolic
Profiling Integrated with Extracellular Vesicle Analysis Reveals Novel
Metabolic Vulnerabilities and Prognostic Biomarkers in Acute Myeloid
Leukemia. Blood.

[510] Koo D., Cheng X., Udani S., Baghdasarian S., Zhu D., Li J., Hall B., Tsubamoto N., Hu S., Ko J. (2024). Optimizing Cell Therapy by Sorting Cells with
High Extracellular Vesicle Secretion. Nat. Commun..

[511] Jiang X., Yang J., Lin Y., Liu F., Tao J., Zhang W., Xu J., Zhang M. (2023). Extracellular Vesicles
Derived from Human Esc-Mscs Target Macrophage and Promote Anti-Inflammation
Process, Angiogenesis, and Functional Recovery in Acs-Induced Severe
Skeletal Muscle Injury. Stem Cell Res. Ther..

[512] Ko J., Wang Y., Sheng K., Weitz D. A., Weissleder R. (2021). Sequencing-Based
Protein Analysis of Single Extracellular Vesicles. ACS Nano.

[513] Rayamajhi S., Gibbs B. K., Sipes J., Pathak H. B., Bossmann S. H., Godwin A. K. (2024). Tracking Small Extracellular Vesicles
Using a Minimally Invasive Picogreen Labeling Strategy. ACS Appl. Bio Mater..

[514] Spitzberg J. D., Ferguson S., Yang K. S., Peterson H. M., Carlson J. C., Weissleder R. (2023). Multiplexed Analysis of Ev Reveals
Specific Biomarker Composition with Diagnostic Impact. Nat. Commun..

[515] Muratori M., Tarozzi N., Carpentiero F., Danti S., Perrone F. M., Cambi M., Casini A., Azzari C., Boni L., Maggi M. (2019). Sperm
Selection with Density Gradient Centrifugation and Swim Up: Effect
on DNA Fragmentation in Viable Spermatozoa. Sci. Rep.

[516] Pösel C., Möller K., Fröhlich W., Schulz I., Boltze J., Wagner D.-C. (2012). Density
Gradient
Centrifugation Compromises Bone Marrow Mononuclear Cell Yield. PloS one.

[517] Zhang Q., Jeppesen D. K., Higginbotham J. N., Franklin J. L., Coffey R. J. (2023). Comprehensive Isolation of Extracellular
Vesicles and Nanoparticles. Nature protocols.

[518] Wang W., Sun H., Duan H., Sheng G., Tian N., Liu D., Sun Z. (2024). Isolation
and Usage
of Exosomes in Central Nervous System Diseases. CNS Neurosci. Therap..

[519] Gao Z., Li Z., Hutchins Z., Zhang Q., Zhong W. (2023). Enhancing
Extracellular Vesicle Analysis by Integration of Large-Volume Sample
Stacking in Capillary Electrophoresis with Asymmetrical Flow Field-Flow
Fractionation. Anal. Chem..

[520] Monguió-Tortajada M., Gálvez-Montón C., Bayes-Genis A., Roura S., Borràs F. E. (2019). Extracellular
Vesicle Isolation Methods: Rising Impact of Size-Exclusion Chromatography. Cell. Mol. Life Sci..

[521] Merij L. B., da Silva L. R., Palhinha L., Gomes M. T., Dib P. R. B., Martins-Gonçalves R., Toledo-Quiroga K., Raposo-Nunes M. A., Andrade F. B., de Toledo Martins S. (2024). Density-Based Lipoprotein Depletion Improves Extracellular Vesicle
Isolation and Functional Analysis. J. Thromb
Haemost.

[522] Skoczylas Ł., Gawin M., Fochtman D., Widłak P., Whiteside T. L., Pietrowska M. (2023). Immune Capture and Protein Profiling
of Small Extracellular Vesicles from Human Plasma. Proteomics.

[523] Normak K., Papp M., Ullmann M., Paganini C., Manno M., Bongiovanni A., Bergese P., Arosio P. (2023). Multiparametric
Orthogonal Characterization of Extracellular Vesicles by Liquid Chromatography
Combined with in-Line Light Scattering and Fluorescence Detection. Anal. Chem..

[524] Leong S. Y., Ong H. B., Tay H. M., Kong F., Upadya M., Gong L., Dao M., Dalan R., Hou H. W. (2022). Microfluidic Size Exclusion Chromatography (Μsec)
for Extracellular Vesicles and Plasma Protein Separation. Small.

[525] Bazaz S. R., Zhand S., Salomon R., Beheshti E. H., Jin D., Warkiani M. E. (2023). Immunoinertial Microfluidics:
A Novel Strategy for
Isolation of Small Ev Subpopulations. Appl.
Mater. Today.

[526] He N., Thippabhotla S., Zhong C., Greenberg Z., Xu L., Pessetto Z., Godwin A. K., Zeng Y., He M. (2022). Nano Pom-Poms
Prepared Exosomes Enable Highly Specific Cancer Biomarker Detection. Commun. Biol..

[527] Zhao W., Han M., Huang X., Xiao T., Xie D., Zhao Y., Tan M., Zhu B., Chen Y., Tang B. Z. (2025). Weight Differences-Based Multi-Level
Signal Profiling
for Homogeneous and Ultrasensitive Intelligent Bioassays. ACS Nano.

[528] Yang J., Pan B., Zeng F., He B., Gao Y., Liu X., Song Y. (2021). Magnetic Colloid Antibodies Accelerate
Small Extracellular Vesicles Isolation for Point-of-Care Diagnostics. Nano Lett..

[529] Jang Y. O., Roh Y., Shin W., Jo S., Koo B., Liu H., Kim M. G., Lee H. J., Qiao Z., Lee E. Y. (2024). Transferrin-Conjugated Magnetic Nanoparticles for the
Isolation of Brain-Derived Blood Exosomal Micrornas: A Novel Approach
for Parkinson’s Disease Diagnosis. Anal.
Chim. Acta.

[530] Ströhle G., Gan J., Li H. (2022). Affinity-Based Isolation
of Extracellular Vesicles and the Effects on Downstream Molecular
Analysis. Anal. Bioanal. Chem..

[531] Mao Y., Li J., Li J., Su C., Long K., Li D., Ding Z., Guo S. (2024). Enhanced Immune
Capture of Extracellular
Vesicles with Gelatin Nanoparticles and Acoustic Mixing. Analyst.

[532] Geng Y., Yu J. (2024). Progress in Constructing Functional
Coacervate Systems Using Microfluidics. BMEMat.

[533] Bai J., Wei X., Zhang X., Wu C., Wang Z., Chen M., Wang J. (2023). Microfluidic Strategies for the Isolation
and Profiling of Exosomes. TrAC Trends in Analytical
Chemistry.

[534] Yu H., Kim J., Yu J., Hyun K.-A., Lim J.-Y., Yoon Y.-J., Park S., Jung H.-I. (2023). Continuous Isolation
of Stem-Cell-Derived Extracellular Vesicles (Sc-Evs) by Recycled Magnetic
Beads in Microfluidic Channels. BioChip Journal.

[535] Kimiz-Gebologlu I., Oncel S. S. (2022). Exosomes: Large-Scale
Production,
Isolation, Drug Loading Efficiency, and Biodistribution and Uptake. J. Controlled Release.

[536] Lai J. J., Chau Z. L., Chen S. Y., Hill J. J., Korpany K. V., Liang N. W., Lin L. H., Lin Y. H., Liu J. K., Liu Y. C. (2022). Exosome Processing and Characterization
Approaches for Research and Technology Development. Adv. Sci..

[537] Chen G. Y., Cheng J. C., Chen Y. F., Yang J. C., Hsu F. M. (2021). Circulating Exosomal Integrin Β3
Is Associated
with Intracranial Failure and Survival in Lung Cancer Patients Receiving
Cranial Irradiation for Brain Metastases: A Prospective Observational
Study. Cancers (Basel).

[538] Zhang N., Chen H., Yang C., Hu X., Sun N., Deng C. (2022). Functionalized Nanomaterials in Separation and Analysis
of Extracellular Vesicles and Their Contents. TrAC Trends in Analytical Chemistry.

[539] Djeungoue Petga M. A., Taylor C., Macpherson A., Dhadi S. R., Rollin T., Roy J. W., Ghosh A., Lewis S. M., Ouellette R. J. (2024). A Simple Scalable Extracellular Vesicle
Isolation Method Using Polyethylenimine Polymers for Use in Cellular
Delivery. Extracell. Vesicle.

[540] Chandrasekera D., Shah R., van Hout I., De Jonge W., Bunton R., Parry D., Davis P., Katare R. (2023). Combination
of Precipitation and Size Exclusion Chromatography as an Effective
Method for Exosome Like Extracellular Vesicle Isolation from Pericardial
Fluids. Nanotheranostics.

[541] Ukkola J., Pratiwi F. W., Kankaanpää S., Abdorahimzadeh S., KarzarJeddi M., Singh P., Zhyvolozhnyi A., Makieieva O., Viitala S., Samoylenko A. (2022). Enrichment of Bovine Milk-Derived Extracellular Vesicles Using Surface-Functionalized
Cellulose Nanofibers. Carbohydr. Polym..

[542] He M., Crow J., Roth M., Zeng Y., Godwin A. K. (2014). Integrated
Immunoisolation and Protein Analysis of Circulating Exosomes Using
Microfluidic Technology. Lab Chip.

[543] Zhang P., Zhou X., He M., Shang Y., Tetlow A. L., Godwin A. K., Zeng Y. (2019). Ultrasensitive
Detection
of Circulating Exosomes with a 3d-Nanopatterned Microfluidic Chip. Nat. Biomed Eng..

[544] Dinh M. T., Mukhamedshin A., Abhishek K., Lam F. W., Gifford S. C., Shevkoplyas S. S. (2024). Separation of Platelets by Size in
a Microfluidic Device Based on Controlled Incremental Filtration. Lab Chip.

[545] Morani M., Taverna M., Krupova Z., Alexandre L., Defrenaix P., Mai T. D. (2022). Development of a Microfluidic Droplet
Platform with an Antibody-Free Magnetic-Bead-Based Strategy for High
through-Put and Efficient Evs Isolation. Talanta.

[546] Meggiolaro A., Moccia V., Sammarco A., Brun P., Damanti C. C., Crestani B., Mussolin L., Pierno M., Mistura G., Zappulli V. (2024). Droplet Microfluidic
Platform for
Extracellular Vesicle Isolation Based on Magnetic Bead Handling. Sens. Actuators, B.

[547] Meng Y., Zhang Y., Bühler M., Wang S., Asghari M., Stürchler A., Mateescu B., Weiss T., Stavrakis S., deMello A. J. (2023). Direct Isolation of Small Extracellular Vesicles from
Human Blood Using Viscoelastic Microfluidics. Sci. Adv..

[548] Kim S., Song J., Roh S. M., Kim H. J., Kim H., Lee S., Yoshie A., Ha T., Kim Y., Lee S.-H. (2024). Efficient Exosome Separation
Utilizing Dielectrophoretic Force in
Conductive Spiral Microfluidic Chips and Validation Via a Reduced
Graphene Oxide (Rgo)-Based Biosensor. Sens.
Actuators, B.

[549] Zhang J., Chen C., Becker R., Rufo J., Yang S., Mai J., Zhang P., Gu Y., Wang Z., Ma Z. (2022). A Solution to the Biophysical
Fractionation of Extracellular Vesicles: Acoustic Nanoscale Separation
Via Wave-Pillar Excitation Resonance (Answer). Sci. Adv..

[550] Kim D., Woo H. K., Lee C., Min Y., Kumar S., Sunkara V., Jo H. G., Lee Y. J., Kim J., Ha H. K. (2020). Ev-Ident: Identifying Tumor-Specific Extracellular
Vesicles by Size Fractionation and Single-Vesicle Analysis. Anal. Chem..

[551] Sharma M., Sheth M., Poling H. M., Kuhnell D., Langevin S. M., Esfandiari L. (2023). Rapid Purification and Multiparametric
Characterization of Circulating Small Extracellular Vesicles Utilizing
a Label-Free Lab-on-a-Chip Device. Sci. Rep..

[552] Qiu J., Guo Q., Chu Y., Wang C., Xue H., Zhang Y., Liu H., Li G., Han L. (2024). Efficient
Evs Separation and Detection by an Alumina-Nanochannel-Array-Membrane
Integrated Microfluidic Chip and an Antibody Barcode Biochip. Anal. Chim. Acta.

[553] Fernández-Rhodes M., Adlou B., Williams S., Lees R., Peacock B., Aubert D., Jalal A. R., Lewis M. P., Davies O. G. (2023). Defining the Influence of Size-Exclusion
Chromatography Fraction Window and Ultrafiltration Column Choice on
Extracellular Vesicle Recovery in a Skeletal Muscle Model. J. Extracell. Biol..

[554] Chernyshev V. S., Yashchenok A., Ivanov M., Silachev D. N. (2023). Filtration-Based
Technologies for Isolation, Purification and Analysis of Extracellular
Vesicles. Phys. Chem. Chem. Phys..

[555] Ko M., Kim H. J., Park J., Lee H., Lee K. N., Kim K., Lee J., Yoon S. J., Kim T., Jeong S. (2023). Isolation of Bovine Milk Exosome Using Electrophoretic
Oscillation
Assisted Tangential Flow Filtration with Antifouling of Micro-Ultrafiltration
Membrane Filters. ACS Appl. Mater. Interfaces.

[556] Yuan, R. ; Zhou, Y. ; Arias, G. F. ; Dittmer, D. P. Extracellular Vesicle Isolation by a Tangential-Flow Filtration-Based Large-Scale Purification Method. In Cell-Secreted Vesicles: Methods and Protocols; Springer, 2023; pp 45–55. 10.1007/978-1-0716-3203-1_5 37140789

[557] Lee C.-L., Vu C.-A., Vu V.-T., Cheng C.-M., Chang Y., Chen W.-Y. (2024). Integrating Sulfobetaine Methacrylate
Hydrogel-Modified Cellulose Triacetate Filter into a Zwitterionized
Tandem Membrane System for Exosome Isolation. ACS Appl. Polym. Mater..

[558] Sorrells J. E., Martin E. M., Aksamitiene E., Mukherjee P., Alex A., Chaney E. J., Marjanovic M., Boppart S. A. (2021). Label-Free Characterization of Single Extracellular
Vesicles Using Two-Photon Fluorescence Lifetime Imaging Microscopy
of Nad (P) H. Sci. Rep..

[559] Walker S. N., Lucas K., Dewey M. J., Badylak S. F., Hussey G. S., Flax J., McGrath J. L. (2025). Rapid Assessment
of Biomarkers on Single Extracellular Vesicles Using “Catch
and Display” on Ultrathin Nanoporous Silicon Nitride Membranes. Small.

[560] Kanabekova P., Dauletkanov B., Bekezhankyzy Z., Toktarkan S., Martin A., Pham T. T., Kostas K., Kulsharova G. (2024). A Hybrid Fluorescent Nanofiber Membrane
Integrated
with Microfluidic Chips Towards Lung-on-a-Chip Applications. Lab Chip.

[561] Sausset R., Krupova Z., Guédon E., Peron S., Grangier A., Petit M. A., De Sordi L., De Paepe M. (2023). Comparison of Interferometric
Light Microscopy with
Nanoparticle Tracking Analysis for the Study of Extracellular Vesicles
and Bacteriophages. J. Extracell. Biol..

[562] Corona M. L., Hurbain I., Raposo G., van Niel G. (2023). Characterization
of Extracellular Vesicles by Transmission Electron Microscopy and
Immunolabeling Electron Microscopy. Methods
Mol. Biol..

[563] Zelinger E., Brumfeld V., Rechav K., Waiger D., Kossovsky T., Heifetz Y. (2024). Three-Dimensional Correlative Microscopy
of the Drosophila Female Reproductive Tract Reveals Modes of Communication
in Seminal Receptacle Sperm Storage. Commun.
Biol..

[564] Morandi M. I., Busko P., Ozer-Partuk E., Khan S., Zarfati G., Elbaz-Alon Y., Abou Karam P., Napso Shogan T., Ginini L., Gil Z. (2022). Extracellular Vesicle Fusion Visualized by Cryo-Electron Microscopy. PNAS Nexus.

[565] Doyle N., Simpson J., Hawes P. C., Maier H. J. (2024). A Novel
Optimized Pre-Embedding Antibody-Labelling Correlative Light Electron
Microscopy Technique. Access Microbiol..

[566] Olofsson Bagge R., Berndtsson J., Urzì O., Lötvall J., Micaroni M., Crescitelli R. (2023). Three-Dimensional
Reconstruction of Interstitial Extracellular Vesicles in Human Liver
as Determined by Electron Tomography. J. Extracell.
Vesicles.

[567] Feng Y., Liu M., Li X., Li M., Xing X., Liu L. (2023). Nanomechanical Signatures of Extracellular
Vesicles from Hematologic Cancer Patients Unraveled by Atomic Force
Microscopy for Liquid Biopsy. Nano Lett..

[568] Kim S. Y., Khanal D., Kalionis B., Chrzanowski W. (2019). High-Fidelity
Probing of the Structure and Heterogeneity of Extracellular Vesicles
by Resonance-Enhanced Atomic Force Microscopy Infrared Spectroscopy. Nat. Protoc..

[569] Puthukodan S., Hofmann M., Mairhofer M., Janout H., Schurr J., Hauser F., Naderer C., Preiner J., Winkler S., Sivun D. (2023). Purification
Analysis, Intracellular Tracking, and Colocalization of Extracellular
Vesicles Using Atomic Force and 3d Single-Molecule Localization Microscopy. Anal. Chem..

[570] Saftics A., Abuelreich S., Romano E., Ghaeli I., Jiang N., Spanos M., Lennon K. M., Singh G., Das S., Van Keuren-Jensen K. (2023). Single Extracellular
Vesicle Nanoscopy. J. Extracell. Vesicles.

[571] Han C., Kang M., Kang H., Yi J., Lim M., Kwon Y., Park J. (2023). Characterization of Extracellular
Vesicle and Virus-Like Particles by Single Vesicle Tetraspanin Analysis. Sens. Actuators, B.

[572] Yang Z., Atiyas Y., Shen H., Siedlik M. J., Wu J., Beard K., Fonar G., Dolle J. P., Smith D. H., Eberwine J. H. (2022). Ultrasensitive Single Extracellular Vesicle
Detection Using High Throughput Droplet Digital Enzyme-Linked Immunosorbent
Assay. Nano Lett..

[573] Alexandre L., Dubrova A., Kunduru A., Surply E., Ribes C., Boucenna I., Gazeau F., Silva A. K. A., Mangenot S., Aubertin K. (2025). Investigating Extracellular
Vesicles
in Viscous Formulations: Interplay of Nanoparticle Tracking and Nanorheology
Via Interferometric Light Microscopy. Small
Sci..

[574] Hendrix A., Lippens L., Pinheiro C., Théry C., Martin-Jaular L., Lötvall J., Lässer C., Hill A. F., Witwer K. W. (2023). Extracellular Vesicle Analysis. Nat. Rev. Methods Primers.

[575] Thane K. E., Davis A. M., Hoffman A. M. (2019). Improved
Methods
for Fluorescent Labeling and Detection of Single Extracellular Vesicles
Using Nanoparticle Tracking Analysis. Sci. Rep..

[576] Kashkanova A. D., Blessing M., Gemeinhardt A., Soulat D., Sandoghdar V. (2022). Precision Size and Refractive Index
Analysis of Weakly Scattering Nanoparticles in Polydispersions. Nat. Methods.

[577] Welsh J. A., Killingsworth B., Kepley J., Traynor T., McKinnon K., Savage J., Appel D., Aldape K., Camphausen K., Berzofsky J. A. (2021). A Simple, High-Throughput
Method of Protein and Label Removal from Extracellular Vesicle Samples. Nanoscale.

[578] Erdbrügger U., Rudy C. K., Etter M. E., Dryden K. A., Yeager M., Klibanov A. L., Lannigan J. (2014). Imaging Flow Cytometry
Elucidates Limitations of Microparticle Analysis by Conventional Flow
Cytometry. Cytometry, Part A.

[579] Liu H., Tian Y., Xue C., Niu Q., Chen C., Yan X. (2022). Analysis of Extracellular Vesicle DNA at the Single-Vesicle Level
by Nano-Flow Cytometry. J. Extracell. Vesicles.

[580] Paul N., Maiti K., Sultana Z., Fisher J. J., Zhang H., Cole N., Morgan T., Smith R. (2024). Human Placenta
Releases Extracellular Vesicles Carrying Corticotrophin Releasing
Hormone Mrna into the Maternal Blood. Placenta.

[581] Driedonks T. A., Ressel S., Tran Ngoc
Minh T., Buck A. H., Nolte-‘t Hoen E. N. (2024). Intracellular Localisation
and Extracellular Release of Y Rna and Y Rna Binding Proteins. J. Extracell. Biol..

[582] Kim J., Xu S., Jung S. R., Nguyen A., Cheng Y., Zhao M., Fujimoto B. S., Nelson W., Schiro P., Franklin J. L. (2024). Comparison
of Ev Characterization by Commercial
High-Sensitivity Flow Cytometers and a Custom Single-Molecule Flow
Cytometer. J. Extracell. Vesicles.

[583] Chen K., Duong B. T. V., Ahmed S. U., Dhavarasa P., Wang Z., Labib M., Flynn C., Xu J., Zhang Y. Y., Wang H. (2023). A Magneto-Activated
Nanoscale Cytometry Platform for Molecular Profiling of Small Extracellular
Vesicles. Nat. Commun..

[584] Welsh J. A., Arkesteijn G. J. A., Bremer M., Cimorelli M., Dignat-George F., Giebel B., Görgens A., Hendrix A., Kuiper M., Lacroix R. (2023). A Compendium
of Single Extracellular Vesicle Flow Cytometry. J. Extracell Vesicles.

[585] Young T. W., Kappler M. P., Hockaden N. M., Carpenter R. L., Jacobson S. C. (2023). Characterization of Extracellular
Vesicles by Resistive-Pulse
Sensing on in-Plane Multipore Nanofluidic Devices. Anal. Chem..

[586] Anderson W., Lane R., Korbie D., Trau M. (2015). Observations
of Tunable Resistive Pulse Sensing for Exosome Analysis: Improving
System Sensitivity and Stability. Langmuir.

[587] Jia R., Rotenberg S. A., Mirkin M. V. (2022). Electrochemical Resistive-Pulse Sensing
of Extracellular Vesicles. Anal. Chem..

[588] Kwon Y., Park J. (2022). Methods to Analyze
Extracellular
Vesicles at Single Particle Level. Micro Nano
Syst. Lett..

[589] Vaidyanathan S., Wijerathne H., Gamage S. S. T., Shiri F., Zhao Z., Choi J., Park S., Witek M. A., McKinney C., Verber M. (2023). High Sensitivity Extended
Nano-Coulter Counter for Detection of Viral Particles and Extracellular
Vesicles. Anal. Chem..

[590] Zou R., Cao W., Chong L., Hua W., Xu H., Mao Y., Page J., Shi R., Xia Y., Hu T. Y. (2019). Point-of-Care Tissue Analysis Using Miniature
Mass Spectrometer. Anal. Chem..

[591] Korte A. R., Yandeau-Nelson M. D., Nikolau B. J., Lee Y. J. (2015). Subcellular-Level
Resolution Maldi-Ms Imaging of Maize Leaf Metabolites by Maldi-Linear
Ion Trap-Orbitrap Mass Spectrometer. Anal. Bioanal.
Chem..

[592] Khoo A., Liu L. Y., Nyalwidhe J. O., Semmes O. J., Vesprini D., Downes M. R., Boutros P. C., Liu S. K., Kislinger T. (2021). Proteomic
Discovery of Non-Invasive
Biomarkers of Localized Prostate Cancer Using Mass Spectrometry. Nature Reviews Urology.

[593] Wang R., Hastings W. J., Saliba J. G., Bao D., Huang Y., Maity S., Kamal Ahmad O. M., Hu L., Wang S., Fan J. (2025). Applications of Nanotechnology
for Spatial Omics: Biological Structures and Functions at Nanoscale
Resolution. ACS Nano.

[594] Waury K., Gogishvili D., Nieuwland R., Chatterjee M., Teunissen C. E., Abeln S. (2024). Proteome Encoded Determinants
of Protein Sorting into Extracellular Vesicles. J. Extracell. Biol..

[595] Wang Y., Zhang K., Huang X., Qiao L., Liu B. (2021). Mass Spectrometry Imaging of Mass
Tag Immunoassay Enables the Quantitative
Profiling of Biomarkers from Dozens of Exosomes. Anal. Chem..

[596] Ventouri I. K., Veelders S., Passamonti M., Endres P., Roemling R., Schoenmakers P. J., Somsen G. W., Haselberg R., Gargano A. F. (2023). Micro-Flow Size-Exclusion
Chromatography for Enhanced Native Mass Spectrometry of Proteins and
Protein Complexes. Anal. Chim. Acta.

[597] Chang C.-J., Huang Y.-N., Lu Y.-B., Zhang Y., Wu P.-H., Huang J.-S., Yang W., Chiang T.-Y., Hsieh H.-S., Chung W.-H. (2024). Proteomic Analysis
of Serum Extracellular
Vesicles from Biliary Tract Infection Patients to Identify Novel Biomarkers. Sci. Rep..

[598] Zhu Q., Luo J., Li H. P., Ye W., Pan R., Shi K. Q., Yang R., Xu H., Li H., Lee L. P. (2023). Robust Acute Pancreatitis Identification and
Diagnosis:
Rapidx. ACS Nano.

[599] Lokumcu T., Iskar M., Schneider M., Helm D., Klinke G., Schlicker L., Bethke F., Müller G., Richter K., Poschet G. (2024). Proteomic, Metabolomic, and Fatty Acid Profiling of Small Extracellular
Vesicles from Glioblastoma Stem-Like Cells and Their Role in Tumor
Heterogeneity. ACS Nano.

[600] Fan S., Poetsch A. (2023). Proteomic Research
of Extracellular Vesicles in Clinical
Biofluid. Proteomes.

[601] Veliz L., Cooper T. T., Grenier-Pleau I., Abraham S. A., Gomes J., Pasternak S. H., Dauber B., Postovit L. M., Lajoie G. A., Lagugné-Labarthet F. (2024). Tandem Sers
and Ms/Ms Profiling of Plasma Extracellular Vesicles for Early Ovarian
Cancer Biomarker Discovery. ACS Sens.

[602] Santiago V. F., Rosa-Fernandes L., Macedo-da-Silva J., Angeli C. B., Mule S. N., Marinho C. R. F., Torrecilhas A. C., Marie S. N. K., Palmisano G. (2024). Isolation
of Extracellular Vesicles
Using Titanium Dioxide Microspheres. Adv. Exp.
Med. Biol..

[603] Dorado E., Doria M. L., Nagelkerke A., McKenzie J. S., Maneta-Stavrakaki S., Whittaker T. E., Nicholson J. K., Coombes R. C., Stevens M. M., Takats Z. (2024). Extracellular
Vesicles as a Promising Source of Lipid Biomarkers for Breast Cancer
Detection in Blood Plasma. J. Extracell. Vesicles.

[604] Verkhoturov D. S., Crulhas B. P., Eller M. J., Han Y. D., Verkhoturov S. V., Bisrat Y., Revzin A., Schweikert E. A. (2021). Nanoprojectile
Secondary Ion Mass Spectrometry for Analysis of Extracellular Vesicles. Anal. Chem..

[605] Xing L., Zhao C. L., Mou H. Z., Pan J., Kang B., Chen H. Y., Xu J. J. (2023). Next Generation
of Mass Spectrometry Imaging: From Micrometer to Subcellular Resolution. Chem. Biomed Imaging.

[606] Martínez-García J., Villa-Vázquez A., Fernández B., González-Iglesias H., Pereiro R. (2024). Exploring
Capabilities of Elemental Mass Spectrometry for Determination of Metal
and Biomolecules in Extracellular Vesicles. Anal Bioanal Chem..

[607] Nguyen T. D. K., Rabasco S., Lork A. A., Toit A. D., Ewing A. G. (2023). Quantitative Nanoscale Secondary Ion Mass Spectrometry
(Nanosims) Imaging of Individual Vesicles to Investigate the Relation
between Fraction of Chemical Release and Vesicle Size. Angew. Chem., Int. Ed..

[608] Chen X., Gao Y., Qi Y., Li J., Hu T. Y., Chen Z., Zhu J.-J. (2025). Label-Free Raman
Probing of the Intrinsic Electric Field for High-Efficiency Screening
of Electricity-Producing Bacteria at the Single-Cell Level. Angew. Chem., Int. Ed..

[609] Buccini L., Proietti A., La Penna G., Mancini C., Mura F., Tacconi S., Dini L., Rossi M., Passeri D. (2024). Toward the
Nanoscale Chemical and Physical Probing
of Milk-Derived Extracellular Vesicles Using Raman and Tip-Enhanced
Raman Spectroscopy. Nanoscale.

[610] Penders J., Nagelkerke A., Cunnane E. M., Pedersen S. V., Pence I. J., Coombes R. C., Stevens M. M. (2021). Single Particle
Automated Raman Trapping Analysis of Breast Cancer Cell-Derived Extracellular
Vesicles as Cancer Biomarkers. ACS Nano.

[611] Li J., Li M., Wuethrich A., Guan R., Zhao L., Hu C., Trau M., Sun Y. (2024). Molecular Stratification and Treatment
Monitoring of Lung Cancer Using a Small Extracellular Vesicle-Activated
Nanocavity Architecture. Anal. Chem..

[612] Parlatan U., Ozen M. O., Kecoglu I., Koyuncu B., Torun H., Khalafkhany D., Loc I., Ogut M. G., Inci F., Akin D., Solaroglu I., Ozoren N., Unlu M. B., Demirci U. (2023). Label-Free
Identification of Exosomes Using Raman Spectroscopy and Machine Learning. Small.

[613] Sun X., Chen B., Li Z., Shan Y., Jian M., Meng X., Wang Z. (2024). Accurate Diagnosis
of Thyroid Cancer
Using a Combination of Surface-Enhanced Raman Spectroscopy of Exosome
on Mxene-Coated Gold@ Silver Core@ Shell Nanoparticle Substrate and
Deep Learning. Chemical Engineering Journal.

[614] Wan, M. ; Amrollahi, P. ; Sun, D. ; Lyon, C. ; Hu, T. Y. Using Nanoplasmon-Enhanced Scattering and Low-Magnification Microscope Imaging to Quantify Tumor-Derived Exosomes. J. Vis. Exp. 2019. 10.3791/59177 PMC8157488 31180357

[615] Ng S. S., Lee H. L., Tran H. L., Doong R.-a. (2024). Composites
of Gold Nanostars and Nitrogen- and Sulfur-Codoped Graphene Quantum
Dots as Plasmon-Enhanced Immunosensors for Cancer Prognosis. ACS Applied Nano Materials.

[616] Wang S., Zheng W., Wang R., Zhang L., Yang L., Wang T., Saliba J. G., Chandra S., Li C., Lyon C. J., Hu T. Y. (2023). Monocrystalline Labeling
Enables Stable Plasmonic Enhancement for Isolation-Free Extracellular
Vesicle Analysis. Small.

[617] Sun D., Fan J., Liu C., Liu Y., Bu Y., Lyon C. J., Hu Y. (2016). Noise Reduction Method for Quantifying
Nanoparticle Light Scattering in Low Magnification Dark-Field Microscope
Far-Field Images. Anal. Chem..

[618] Liang K., Liu F., Fan J., Sun D., Liu C., Lyon C. J., Bernard D. W., Li Y., Yokoi K., Katz M. H. (2017). Nanoplasmonic Quantification
of Tumour-Derived Extracellular
Vesicles in Plasma Microsamples for Diagnosis and Treatment Monitoring. Nat. Biomed. Eng..

[619] Sharar N., Wüstefeld K., Talukder R. M., Skolnik J., Kaufmann K., Giebel B., Börger V., Nolte F., Watzl C., Weichert F. (2023). The Employment
of the
Surface Plasmon Resonance (Spr) Microscopy Sensor for the Detection
of Individual Extracellular Vesicles and Non-Biological Nanoparticles. Biosensors.

[620] Wang X., Zeng Q., Xie F., Wang J., Yang Y., Xu Y., Li J., Yu H. (2021). Automated
Nanoparticle Analysis in Surface Plasmon Resonance Microscopy. Anal. Chem..

[621] Jeong M. H., Son T., Im H. (2023). Plasmon-Enhanced Characterization
of Single Extracellular Vesicles. Methods Mol.
Biol..

[622] Hong C., Ndukaife J. C. (2023). Scalable Trapping of Single Nanosized
Extracellular Vesicles Using Plasmonics. Nat.
Commun..

[623] Feng H., Min S., Huang Y., Gan Z., Liang C., Li W.-D., Chen Y. (2024). Concentric Gradient
Nanoplasmonic Sensors for Detecting Tumor-Derived Extracellular Vesicles. Sens. Actuators, B.

[624] Sun D., Hu T. Y. (2018). A Low Cost Mobile Phone Dark-Field
Microscope for Nanoparticle-Based
Quantitative Studies. Biosens Bioelectron.

[625] Min J., Son T., Hong J. S., Cheah P. S., Wegemann A., Murlidharan K., Weissleder R., Lee H., Im H. (2020). Plasmon-Enhanced
Biosensing for Multiplexed Profiling of Extracellular Vesicles. Adv. Biosyst..

[626] Wang Z., Feng N., Zhou Y., Cheng X., Zhou C., Ma A., Wang Q., Li Y., Chen Y. (2024). Mesophilic Argonaute-Mediated Polydisperse Droplet
Biosensor for
Amplification-Free, One-Pot, and Multiplexed Nucleic Acid Detection
Using Deep Learning. Anal. Chem..

[627] Wang Z., Ma A., Chen Y. (2024). An Amplification-Free
Digital Assay Based on Primer Exchange Reaction-Mediated Botryoidal-Like
Fluorescent Polystyrene Dots to Detect Multiple Pathogenic Bacteria. ACS Nano.

[628] Qu Y., Bai Y., Wu Z., Yang D., Liu H., Mao H. (2023). Non-Invasive Detection of Tumor Markers in Salivary Extracellular
Vesicles Based on Digital Pcr Chips. Clin. Chim.
Acta.

[629] Liu C., Lin H., Guo J., Yang C., Chen J., Pan W., Cui B., Feng J., Zhang Y., Li B. (2023). Profiling
of Single-Vesicle Surface Proteins Via Droplet Digital
Immuno-Pcr for Multi-Subpopulation Extracellular Vesicles Counting
Towards Cancer Diagnostics. Chemical Engineering
Journal.

[630] Clarissa E. M., Kumar S., Park J., Karmacharya M., Oh I. J., Kim M. H., Ryu J. S., Cho Y. K. (2025). Digital
Profiling of Tumor Extracellular Vesicle-Associated Rnas Directly
from Unprocessed Blood Plasma. ACS Nano.

[631] Feng X., Zhai C., Xu J., Yang Y., Yu H. (2023). Automatically Digital Extracellular
Vesicles Analyzer for Size-Dependent
Subpopulation Analysis in Surface Plasmon Resonance Microscopy. View.

[632] Alwarappan S., Nesakumar N., Sun D., Hu T. Y., Li C. Z. (2022). 2d Metal Carbides and Nitrides (Mxenes)
for Sensors and Biosensors. Biosens Bioelectron.

[633] Cao Y., Zhou L., Zhou G., Liu W., Cui H., Cao Y., Zuo X., Zhao J. (2024). Proximity
Labeling-Assisted Click
Conjugation for Electrochemical Analysis of Specific Subpopulations
in Circulating Extracellular Vesicles. Biosens.
Bioelectron..

[634] Liu X., Wang Y., Du Y., Zhang J., Wang Y., Xue Y., Zhao J., Ge L., Yang L., Li F. (2024). Laser-Induced
Graphene (Lig)-Based Electrochemical Microfluidic Chip for Simultaneous
Analysis of Multiplex Micrornas. Chemical Engineering
Journal.

[635] Huang J., Chen T., Zhao Y., Li D., Huang Q., Cao L., Chen J., Chen D., Hu L., Liu H. (2024). Colloidal
Quantum Dots-Modified Electrochemical Sensor
for High-Sensitive Extracellular Vesicle Detection. Chemical Engineering Journal.

[636] Cinti S., Tomassi S., Ciardiello C., Migliorino R., Pirozzi M., Leone A., Di Gennaro E., Campani V., De Rosa G., D’Amore V. M. (2024). Paper-Based Electrochemical Device for Early Detection of Integrin
Αvβ6 Expressing Tumors. Commun.
Chem..

[637] Gurudatt N., Gwak H., Hyun K.-A., Jeong S.-E., Lee K., Park S., Chung M. J., Kim S.-E., Jo J. H., Jung H.-I. (2023). Electrochemical Detection and Analysis of Tumor-Derived
Extracellular Vesicles to Evaluate Malignancy of Pancreatic Cystic
Neoplasm Using Integrated Microfluidic Device. Biosens. Bioelectron..

[638] Yang S., Zhou L., Fang Z., Wang Y., Zhou G., Jin X., Cao Y., Zhao J. (2024). Proximity-Guaranteed
DNA Machine for Accurate Identification of Breast Cancer Extracellular
Vesicles. ACS sensors.

[639] Wang Z., Cheng X., Ma A., Jiang F., Chen Y. (2025). Multiplexed Food-Borne Pathogen Detection
Using an Argonaute-Mediated
Digital Sensor Based on a Magnetic-Bead-Assisted Imaging Transcoding
System. Nat. Food.

[640] Zeng X., Wu C., Xiong Y., Zhan Z., Shen C., Lin F., Zhang J., Chen P. (2024). Target Proteins-Regulated
DNA Nanomachine-Electroactive Substance Complexes Enable Separation-Free
Electrochemical Detection of Clinical Exosome. Biosens. Bioelectron..

[641] Ramadan S., Lobo R., Zhang Y., Xu L., Shaforost O., Kwong Hong Tsang D., Feng J., Yin T., Qiao M., Rajeshirke A. (2021). Carbon-Dot-Enhanced
Graphene Field-Effect Transistors for Ultrasensitive Detection of
Exosomes. ACS Appl. Mater. Interfaces.

[642] Madhivanan K., Atchudan R., Arya S., Sundramoorthy A. K. (2024). Utilization
of Nanomaterials Functionalized Bio-Field-Effect Transistors for Detection
of Cancer Biomarkers. Oral. Oncol. Rep..

[643] Bao D., Maity S., Zhan L., Seo S., Shu Q., Lyon C. J., Ning B., Zelazny A., Hu T. Y., Fan J. (2025). Precise Mycobacterial Species and
Subspecies Identification Using
the Pep-Torch Peptidome Algorithm. EMBO Mol.
Med..

[644] Zhou Y., Zhao J., Wen J., Wu Z., Dong Y., Chen Y. (2025). Unsupervised Learning-Assisted Acoustic-Driven
Nano-Lens Holography for the Ultrasensitive and Amplification-Free
Detection of Viable Bacteria. Adv. Sci..

[645] Zhang Q., Ren T., Cao K., Xu Z. (2024). Advances of
Machine Learning-Assisted Small Extracellular Vesicles Detection Strategy. Biosens. Bioelectron..

[646] Min L., Bao H., Bu F., Li X., Guo Q., Liu M., Zhu S., Meng J., Zhang S., Wang S. (2023). Machine-Learning-Assisted
Procoagulant Extracellular Vesicle Barcode Assay toward High-Performance
Evaluation of Thrombosis-Induced Death Risk in Cancer Patients. ACS Nano.

[647] Luo M., Lan F., Yang C., Ji T., Lou Y., Zhu Y., Li W., Chen S., Gao Z., Luo S. (2024). Sensitive Small Extracellular Vesicles Associated Circrnas Analysis
Combined with Machine Learning for Precision Identification of Gastric
Cancer. Chemical Engineering Journal.

[648] Li X., Liu Y., Fan Y., Tian G., Shen B., Zhang S., Fu X., He W., Tao X., Ding X. (2024). Advanced Nanoencapsulation-Enabled
Ultrasensitive Analysis:
Unraveling Tumor Extracellular Vesicle Subpopulations for Differential
Diagnosis of Hepatocellular Carcinoma Via DNA Cascade Reactions. ACS Nano.

[649] Cheng S., Zhang C., Hu X., Zhu Y., Shi H., Tan W., Luo X., Xian Y. (2024). Ultrasensitive Determination
of Surface Proteins on Tumor-Derived Small Extracellular Vesicles
for Breast Cancer Identification Based on Lanthanide-Activated Signal
Amplification Strategy. Talanta.

[650] Guo F., Sun M., Zhang Y., Xie J., Gao Q., Duan W.-J., Chen J.-X., Chen J., Dai Z., Li M. (2023). A Dual Aptamer Recognition-Based Fluorescent Biosensor
for Extracellular
Vesicles Assays with High Sensitivity and Specificity. Sens. Actuators, B.

[651] Wu J., Lin Z., Zou Z., Liang S., Wu M., Hu T. Y., Zhang Y. (2022). Identifying the Phenotypes of Tumor-Derived
Extracellular Vesicles Using Size-Coded Affinity Microbeads. J. Am. Chem. Soc..

[652] Park C., Chung S., Kim H., Kim N., Son H. Y., Kim R., Lee S., Park G., Rho H. W., Park M. (2024). All-in-One Fusogenic
Nanoreactor for the Rapid Detection of Exosomal Micrornas for Breast
Cancer Diagnosis. ACS Nano.

[653] Al Ja’farawy M. S., Linh V. T. N., Yang J.-Y., Mun C., Lee S., Park S.-G., Han I. W., Choi S., Lee M.-Y., Kim D.-H. (2024). Whole Urine-Based Multiple
Cancer Diagnosis and Metabolite Profiling Using 3d Evolutionary Gold
Nanoarchitecture Combined with Machine Learning-Assisted Sers. Sens. Actuators, B.

[654] Heydari R., Fayazzadeh S., Shahrokh S., Shekari F., Farsad F., Meyfour A. (2024). Plasma Extracellular Vesicle Lncrna
H19 as a Potential Diagnostic Biomarker for Inflammatory Bowel Diseases. Inflammatory Bowel Diseases.

[655] Yan S., Jiang C., Janzen A., Barber T. R., Seger A., Sommerauer M., Davis J. J., Marek K., Hu M. T., Oertel W. H. (2024). Neuronally Derived Extracellular Vesicle Α-Synuclein
as a Serum Biomarker for Individuals at Risk of Developing Parkinson
Disease. JAMA Neurol.

